# Identification of genetic susceptibility loci for intestinal Behçet’s disease

**DOI:** 10.1038/srep39850

**Published:** 2017-01-03

**Authors:** Seung Won Kim, Yoon Suk Jung, Jae Bum Ahn, Eun-Soon Shin, Hui Won Jang, Hyun Jung Lee, Tae Il Kim, Do Young Kim, Dongsik Bang, Won Ho Kim, Jae Hee Cheon

**Affiliations:** 1 Department of Internal Medicine and Institute of Gastroenterology, Yonsei University College of Medicine, Seoul, Korea; 2 Severance Biomedical Science Institute, Yonsei University College of Medicine, Seoul, Korea; 3 Brain Korea 21 PLUS Project for Medical Science, Yonsei University College of Medicine, Seoul, Korea; 4 Division of Gastroenterology, Department of Internal Medicine, Kangbuk Samsung Hospital, Sungkyunkwan University School of Medicine, Seoul, Korea; 5 Department of Medicine, Yonsei University College of Medicine, Seoul, Korea; 6 Bioinformatics team, DNA Link Inc., Seoul, Korea; 7 Department of Dermatology and Cutaneous Biology Research Institute, Yonsei University College of Medicine, Seoul, Korea; 8 Department of Dermatology, Catholic Kwandong University International St. Mary’s Hospital, Seoul, Korea

## Abstract

Several recent genome-wide association studies (GWAS) identified susceptibility loci/genes for Behçet’s disease (BD). However, no study has specifically investigated the genetic susceptibility loci associated with intestinal involvement in BD. We aimed to identify distinctive genetic susceptibility loci/genes associated with intestinal involvement in BD and determine their roles in intestinal inflammation as well as their interactions with genes involved in inflammatory bowel disease (IBD). GWAS and validation studies showed intestinal BD-specific associations with an *NAALADL2* gene locus (rs3914501, *P* = 3.8 × 10^−4^) and a *YIPF7* gene locus (rs6838327, *P* = 3.5 × 10^−4^). Validation, haplotype, and pathway analyses showed distinct genetic architectures between intestinal BD and BD without intestinal involvement. Furthermore, network analysis revealed shared pathogenic pathways between intestinal BD and IBD. Gene functional analyses indicated that down-regulation of *NAALADL2* and *YIPF7* expression was associated with exacerbating intestinal inflammatory responses both *in vitro* and *in vivo*. Our results provide new insights into intestinal BD-specific genetic variations, which represents a distinct pathway from BD without intestinal involvement. Functional consequences of the intestinal BD-specific *NAALADL2* and *YIPF7* expression patterns proved a suggestive association with intestinal inflammation risk, which warrants further validation.

Behçet’s disease (BD) is a rare, chronic, inflammatory, multi-systemic disorder characterized by recurrent oral and genital ulcers, ocular lesions, skin manifestations, and arthritis, as well as vascular, neurological, and intestinal involvement^1^,^2^. Multiple factors, including undefined environmental components and host genetic changes, cooperatively interact and participate in the development of the disease^1^,^2^,^3^. Although the nature of these genetic variants remains unknown for the most part, various genetic risk factors are considered to contribute to the disease susceptibility^2^,^3^,^4^,^5^. In addition to human leukocyte antigen gene encoding B*51 (HLA-B*51) and regions encompassing major histocompatibility complex (*MHC*) class I, genome-wide association studies (GWAS) have identified several other BD susceptibility genes, including interleukin (*IL)10*, IL23 receptor (*IL23R*), IL12 receptor beta 2 (*IL12RB2*), C-C chemokine receptor 1 gene (*CCR1*), signal transducer and activator of transcription (*STAT4*), genes encoding killer cell lectin-like receptor family members (*KLRC4-KLRK1*), and endoplasmic reticulum aminopeptidase (*ERAP1*)^2^,^3^,^4^,^5^,^6^.

Intestinal BD (BD with intestinal involvement) is diagnosed when there is a typically shaped ulcer in the gastrointestinal tract, and clinical findings meet the diagnostic criteria for BD^7^. Intestinal BD can result in severe complications, including bowel perforation and extensive hemorrhage, and is therefore one of the major causes of morbidity and mortality in patients with BD^8^. Intestinal involvement is rare in Mediterranean BD patients (0 to 3%)^9^, whereas it is relatively more prevalent in East Asia, including Korea and Japan (5–25%)^10^. Given these geographic and racial differences in the incidence of BD with or without intestinal involvement, intestinal BD is likely to be associated with pathogenic pathways that are distinct from those contributed to BD without intestinal involvement. Some phenotypic overlaps (inflammation in the eyes, skin, and intestine); shared genetic associations in the MHC class I region, *IL10,* and *IL23R*; and the effectiveness of tumor necrosis factor (TNF)-α blockade also imply common pathogenic pathways between BD and inflammatory bowel disease (IBD)^11^,^12^. Given that IBD and intestinal BD share a number of clinical phenotypes in terms of intestinal manifestations, therapeutic applications, and clinical courses^12^, they may show a shared inherited susceptibility mechanism. However, intestinal BD and IBD have also been considered as two distinct diseases by some authors. Thus, there may be specific genetic markers for intestinal BD that are different from those for IBD. Although GWAS have revealed several genetic susceptibility loci associated with BD development^2^,^3^,^4^,^5^,^6^, all of these studies focused on BD itself. The specific genetic factors associated with intestinal BD have not yet been examined, which is mainly owing to the rarity of the disease.

In this study, we sought to discover specific genetic susceptibility loci associated with intestinal BD, through GWAS and replication studies in the Korean population. This study was performed with the intention of defining specific genetic markers for intestinal BD. We also investigated the functional consequences of genetic factors that influence both disease susceptibility and BD phenotypes with intestinal involvement, as well as shared pathogenic pathways related to IBD.

## Results

### GWAS of intestinal BD and single nucleotide polymorphism (SNP) selection

A GWAS was performed including 100 BD patients without intestinal involvement, 99 patients with intestinal BD, and 597 controls. The results of principal components analysis (PCA) showed no stratification resulting from the population admixture, similar to the previous Korean IBD GWAS data^13^,^14^ (Fig. S1). An overview of the clinical characteristics of the study samples is provided in Table 1. We focused on independent polygenic factors specifically related to intestinal BD, because we hypothesized that intestinal BD originates from additional polygenic factors that might be different from those related to BD without intestinal involvement. The detailed workflow of our association studies is depicted in Fig. S2.

First, intestinal BD and BD without intestinal involvement were directly compared, but none of the SNPs was significant after conservative Bonferroni correction, owing to the small sample size. Therefore, the SNPs showing the lowest *P* values in our GWAS were subjected to a validation test, including rs6838327 in *YIPF7* in a clustered genomic locus on chromosome 4, rs7941240 on chromosome 11 in the intergenic region, and rs2655653 on chromosome 6 in the intergenic region (Fig. S2a). Next, to exclude any genetic factors related to BD without intestinal involvement, only the loci that were significant in intestinal BD and were not significant in the comparison of BD without intestinal involvement to healthy controls were reanalyzed. As a result, only two SNPs in non-clustered regions met the genome-wide significance threshold, of which only one SNP with the lowest *P* value (rs4500591; near *MIR548F5* and *NBEA; P* = 8.8 × 10^−16^) was selected for the next step. Furthermore, 12 additional significant SNPs from four clustered genomic loci (*DCAF12, PLCB1, ELMO1*, and *NAALADL2*) were included for validation, even though the significance level did not reach the Bonferroni correction threshold (Fig. S2b and Supporting information 2). BD patients without intestinal involvement were also compared to the healthy control group. Among 342 significant SNPs, there were 6 clustered SNPs, including 2 genomic loci (*LOC284395, CD300LB*), that met the Bonferroni threshold. Among these, we selected one SNP in *LOC284395,* presenting the lowest *P* value (rs7245731, *P* = 1.9 × 10^−9^; Fig. S2c). The SNP in *CD300LB* (rs61730133) was identified as a susceptibility locus for BD without intestinal involvement. This SNP was also detected in the imputation analysis as an intestinal BD-specific gene (Fig. S2d and Supporting information 2). Finally, we compared the 32 BD-specific SNPs with significant *P* values between the intestinal BD and BD without intestinal involvement comparison, and selected one SNP (rs32019, CD180; Fig. S2e) for further analysis. Manhattan and regional association plots in the discovery group were constructed to provide a detailed overview of the disease-associated regions, as shown in Figs S3 and S4. Overall, 19 SNPs from our GWAS were chosen to be validated.

The combinations of multiple variants with small effects helps to explain the overall susceptibility to a multifactorial disease^15^. Therefore, a haplotype analysis was conducted with additional proxy SNPs to better evaluate the genetic associations at the haplotype level. As a result, 12 additional SNPs were selected for the validation study. Finally, 13 previously reported potential candidate SNPs were validated after literature review. Collectively, 44 selected SNPs were used in a replication study, including 196 independent patients with intestinal BD, 138 BD patients without intestinal involvement, and 391 healthy controls, to validate the GWAS screening results (Supporting information 3).

### Validation study results

A comparison of SNPs between intestinal BD and BD without intestinal involvement showed an intestinal BD-specific association of the *YIPF7* gene locus (rs6838327, odds ratio [OR] = 1.567, combined *P* = 3.5 × 10^−4^, Table 2) after Bonferroni correction (*P* < 0.05/44 SNPs). In addition, the *NAALADL2* locus on chromosome 3 (rs3914501) showed a significant association with the development of intestinal BD (OR = 1.914, *P* = 3.8 × 10^−4^, Table 3) in the combined analysis and modest evidence of an association in the replication analysis (*P* = 1.6 × 10^−2^). Notably, among the genetic loci implicated in previous GWAS^2^,^3^,^4^,^5^,^6^,^16^*, IL10* and *ERAP1* also showed modest associations with intestinal BD (rs1518111, combined *P* = 7.6 × 10^−3^, Table 3; rs2927615, combined *P* = 1.6 × 10^−2^), and *HLA-B* (near *PSORS1C1*) was significantly associated with BD without intestinal involvement compared to healthy controls (rs4959053, combined *P* = 6.6 × 10^−5^, *P* replication = 4.6 × 10^−3^; rs12525170, combined *P* = 1.3 × 10^−5^, *P* replication = 1.1 × 10^−5^, Table 4). However, *CCR1-CCR3, IL23R-IL12RB2, HLA-F-AS1–HLA-A, STAT4*, and *KLRC4-KLRK1* did not show significant associations with any BD subtypes in our study, which might be due to the limited sample size or differences in ethnic background effects on disease development. Our validation stage had sufficient power (≥0.8) to detect an OR of 2.5 for the *YIPF7* SNP with a minor allele frequency (MAF) of 0.43, and an OR of 2.2 for the *HLA-B* SNP with an MAF of 0.13 (Table S1). Data for all of the replicated SNPs are summarized in Supporting information 3.

### Haplotype analysis

We performed a haplotype analysis to further elucidate the genetic factors involved in intestinal BD pathogenesis. In the haplotype analysis (Table S2), *DCAF12* (C-A, *P* = 2.4 × 10^−3^), *IL10* (G-C-C, *P* = 7.3 × 10^−3^), *PLCB1* (C-C-T-T-G, *P* = 1.5 × 10^−2^), *SCHIP1* (G-C, *P* = 3.2 × 10^−3^), and *TGFBR3* (C-C-G, *P* = 2.2 × 10^−2^) showed associations with intestinal BD development. These results indicate that these genes are potential causal variants that contribute to intestinal BD development. In contrast, *HLA-B* showed associations with BD without intestinal involvement (G-A-A, *P* = 2.4 × 10^−5^). The associations of *NAALADL2* (C-G, *P* = 1.2 × 10^−2^) and loci near *HLA-B* (G-A-A, *P* = 9.7 × 10^−3^) were significant in the comparison between intestinal BD and BD without intestinal involvement. These different genetic risks reflect the specificity of biological genetic markers for intestinal involvement.

### Clinical outcomes according to genotype

**Figure 1:**
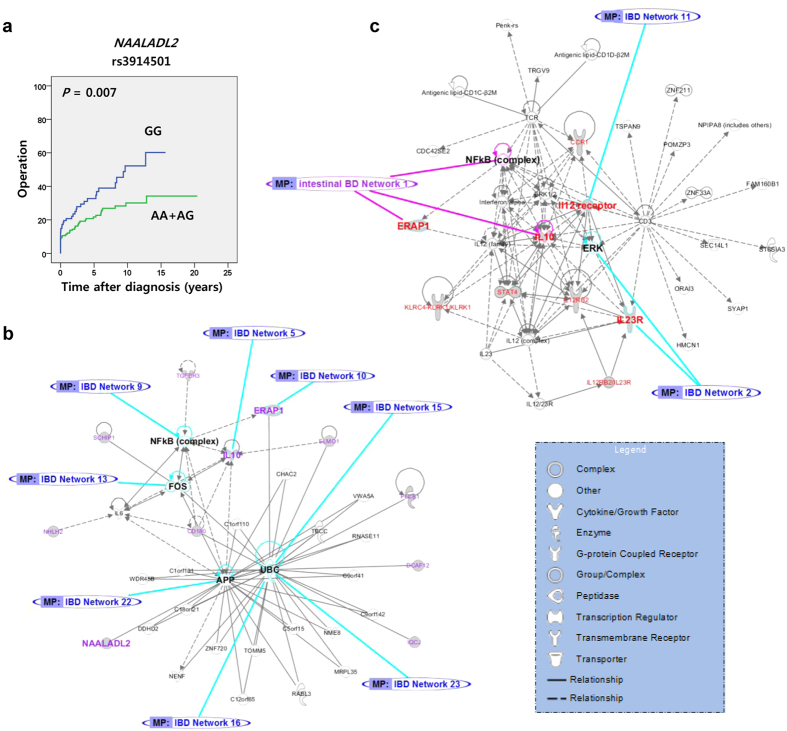
Clinical outcomes according to *NAALADL2* genotype and pathway analysis. (**a**) Cumulative probabilities of surgery according to *NAALADL2* genotype in patients with intestinal Behçet’s disease (BD). The prognosis of intestinal BD was analyzed using the Kaplan–Meier method with differences determined by the log-rank test. Blue lines show risk alleles. (**b**,**c**) Network Diagrams of BD susceptibility genes. (**b**) Networks of overlap between intestinal BD (purple) and inflammatory bowel disease (IBD, blue). Blue lines show the connection of intestinal BD pathways with those of IBD. (**c**) Networks of overlap between intestinal BD (purple) and BD without intestinal involvement (red). Pink lines show the connection of BD pathways with those of IBD.

We assessed whether the investigated SNPs could influence clinical manifestations and prognosis, including intestinal complications and the cumulative probabilities of surgery, hospitalization, corticosteroid use, and immunosuppressant use, in patients with intestinal BD. The risk allele of rs3914501 in *NAALADL2* was associated with a higher cumulative probability of surgery (Fig. 1a). In addition, rs3914501 and rs16848171 risk alleles showed modest associations with a higher cumulative probability of hospitalization and corticosteroid use, respectively (Fig. S5), suggesting their association with a poorer prognosis. *DCAF12* (rs10758242) and *TGFBR3* (rs284148) showed modest associations with intestinal fistula development and intestinal stricture, respectively (Table S3). *HLA-B* genotype showed a strong association with HLA-B*51 positivity in both intestinal BD patients (OR: 26.07, Table S3) and BD patients without intestinal involvement (OR: 56.53, Table S4). *HLA-B* genotype also showed a strong association with central nervous system lesions (OR: 23.23, Table S4) in BD patients without intestinal involvement.

### Pathway analysis

Functionally related genes collectively contribute to disease susceptibility, including loci that do not reach the genome-wide significance threshold individually^17^. Ingenuity Pathway Analysis (IPA) based on published data was used to identify the potential biological pathways of the genes responsible for disease susceptibility for the validated SNPs (*P* < 0.05). Overlap between significant regions was examined according to the published GWAS results (GWAS catalog: Supporting information 6). Notably, the pathway analyses showed that intestinal BD genes (13 genes) comprise a single functional network, including 8 focus molecules that overlapped with eight networks (IL10, NFκB, ERAP1, FOS, UBC, UBC, APP, UBC) for the genes identified in the IBD GWAS (Fig. 1b). These results indicate that the phenotype of intestinal BD shares some common pathogenic risk factors with IBD (Fig. S6a). Intestinal BD-related genes have only one network (ERAP1, IL10) with 14 genes derived from the previous BD GWAS catalog, which may also indirectly connect the intestinal BD pathway to the IBD networks (IL23R, IL12, Figs 1c and S6c). NAALADL2 was included in the pathway analysis with the IBD genes, but YIPF7 was not included because the exact function of this protein remains unknown. The functional network showed potential functions in common for PI3K signaling in B lymphocytes (PLCB1, CD180) and CXCR4 (PLCB1, ELMO1) signaling with two focus molecules (CD180, ELMO1) related to five genes for BD without intestinal involvement (Fig. S6b). This indicates that some intestinal BD genes contribute to BD phenotypes through interactions with genes for BD without intestinal involvement.

### Gene functional analysis of *NAALADL2* and *YIPF7*

**Figure 2:**
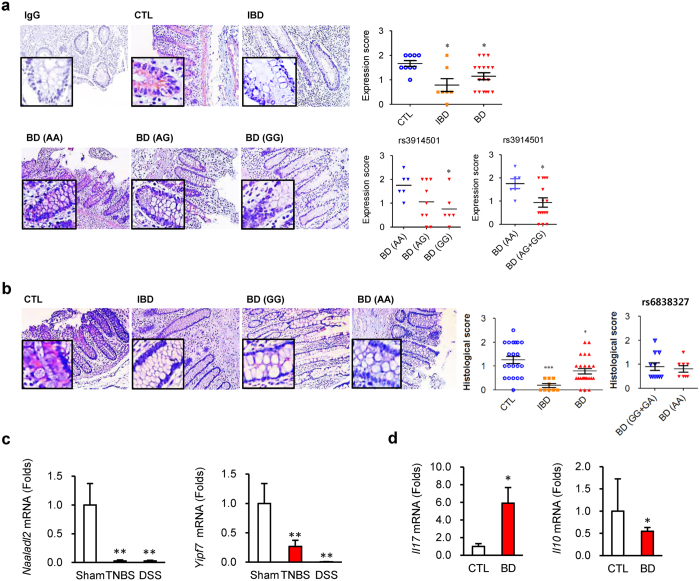
Gene expression analyses in the colons of patients with Behçet’s disease and inflammatory bowel disease and the inflamed colons of mice. (**a,b**) Immunohistochemistry of NAALADL2 (**a**) and YIPF7 (**b**). Right panels show expression scores according to diseases and SNP genotypes (*NAALADL2*, rs3914501; *YIPF7*: rs6838327). Pink and blue show NAALADL2 and the nucleus, respectively. The genotype is indicated in parentheses. (**c,d**) mRNA levels in inflamed colon tissues. (**c**) Transcript levels of *NAALADL2* from control, TNBS-, and DSS-treated mouse colon tissues. Data represent mean ± SEM. (**d**) Transcript levels of *IL17* and *IL10* in colon tissues from control and intestinal BD patients (controls, n = 7; intestinal BD, n = 8). mRNA levels were quantified by qRT-PCR. *P < 0.05 vs. CTL, **P < 0.005 *vs.* CTL or Sham. Data represent mean ± SD. BD, intestinal Behçet’s diseases; CTL, control; IBD, inflammatory bowel disease; TNBS, 2,4 6-trinitrobenzenesulfonic acid; DSS, dextran sulfate sodium.

Next, we validated these associations using experimental functional studies to complement our genetic study results. Immunohistochemical analyses for NAALADL2 and YIPF7 were performed using inflamed colon tissues obtained from patients with intestinal BD or IBD and normal colon tissues obtained from patients with colorectal cancer after intestinal surgery. The protein expression levels of NAALADL2 and YIPF7 were significantly decreased in the tissues of patients with IBD and intestinal BD compared to those in control tissues. The difference was more dramatic in patients with risk alleles than in those without risk alleles (Fig. 2a,b).

**Figure 3:**
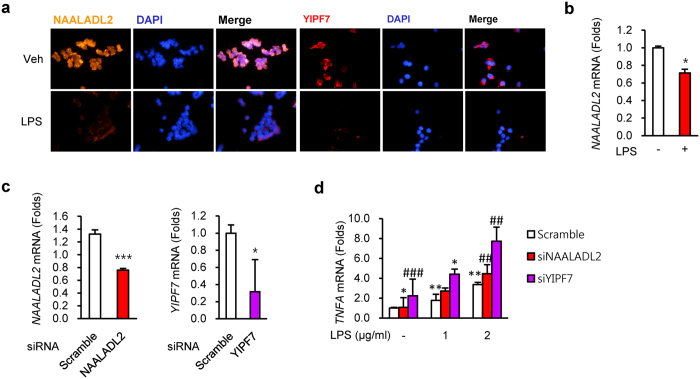
Functional analyses of *NAALADL2* and *YIPF7* in human intestinal epithelial cells. (**a**) Immunofluorescent staining for NAALADL2 and YIPF7 in HT-29 cells after 1 μg/mL lipopolysaccharide (LPS) treatment for 24 h. Orange, NAALADL2; red, YIPF7; blue, DAPI. (**b–d**) mRNA quantification. mRNA levels of *NAALADL2* and *YIPF7* in HT-29 cells after LPS treatment for 4 h (**b**) and after short interfering RNA (siRNA) treatment for 24 h (**c**). (**d**) *TNFA* mRNA levels in gene-knockdown cells (si*NAALADL2* and *siYIPF7,* respectively). Data represent mean ± SEM (n = 3). mRNA levels were quantified by qRT-PCR.

Concordantly, the mRNA levels of *NAALADL2* and *YIPF7* in the inflamed colon tissues of mice were lower than control mice, respectively (Fig. 2c). In addition, the colon tissues of patients with intestinal BD showed increased *IL17* mRNA expression levels, along with decreased *IL10* mRNA expression levels (Fig. 2d). Previous studies indicate that perturbed homeostasis between commensal bacteria and mucosal immunity serves as a critical determinant in the development of gut inflammation in IBD for a genetically susceptible individual^18^,^19^. In addition, aberrant Toll-like receptor (TLR) modulation, such as TLR4, by lipopolysaccharide (LPS), an important stimulator of cytokines such as TNF-α, may contribute to the development of IBD^20^. Thus, additional functional studies were conducted in intestinal epithelial cells using LPS stimulation. LPS reduced NAALADL2 and YIPF7 protein and mRNA levels in HT-29 cells (Fig. 3a,b). Furthermore, knockdown of *NAALADL2* and *YIPF7* by short interfering RNAs (siRNAs) resulted in higher *TNF* mRNA levels than controls (Figs 3c,d and S7).

## Discussion

Variations of clinical phenotypes in patients with BD are well known, but the underlying contribution of genetic variations to these phenotypes has not yet been explored. Here lies the novel contribution of our GWAS. We identified intestinal BD-specific associations of loci near *NAALADL2* and *YIPF7*. An SNP in *NAALADL2* (rs62285902) was also previously identified as a candidate gene in the Crohn’s disease genome-wide imputation analysis of the Japanese population^21^. In addition, the *NAALADL2*-associated rs3914501 risk allele was reported to be linked to the development of Kawasaki disease and Cornelia de Lange syndrome, a rare developmental malformation syndrome^22^. Since Kawasaki disease affects blood vessels and BD is also a form of vasculitis, the fact that *NAALADL2* showed a novel association with intestinal BD in both our replication and subsequent haplotype analyses is intriguing. Moreover, the *NAALADL2*-associated rs3914501 risk allele was associated with poor prognosis of intestinal BD. Dysfunction of YIP1 family members may deregulate intestinal homeostasis, leading to a pathogenic state^23^. Mice with null mutated *Yipf6* were extremely sensitive to dextran sodium sulfate (DSS)-induced colitis. Consistently, we newly identified more frequent variations of *YIPF7*, another YIP1 family member, in patients with intestinal BD than in those without intestinal BD.

Although IHC and mRNA results cannot explain the causal relationships between the candidate genes and disease, they do support the hypothesis of an association between genetic variation and alteration of gene expression, which can be indirectly be represented by altered IHC and mRNA expression levels of *NAALADL2* and *YIPF7*. Supporting these results, SNPs in *NAALADL2* and *YIPF7* presented transcriptional regulatory activities to directly influence their expression (Supporting information 4, RegulomeDB and Haploreg). Moreover, we showed that the expression levels of two genes were reduced by LPS, which consequently upregulated TNF-α expression. We think that these two genes are the mediators of LPS signaling, and the downregulation of the expression of these two genes from genetic variations due to mRNA or protein instability might upregulate TNF-α expression and aggravate colitis. Perturbed homeostasis between commensal bacteria and mucosal immunity serves as a critical determinant in the development of gut inflammation in inflammatory bowel disease (IBD) for genetically susceptible individuals^18^. Aberrant modulation of TLRs (e.g., TLR4) by LPS, an important stimulator of cytokines such as TNF-α, may contribute to the development of IBD^20^. We used LPS as a microbial triggering factor of intestinal bacteria for the initiation of homeostasis disruption. TNF-α is a key cytokine in the pathogenesis of IBD and BD, and its blockade is now commonly used as a standard therapy for IBD and BD clinical practice. In fact, both genes showed higher expression in crypts than in the lamina propria (data not shown), suggesting that the genes function in epithelial cells rather than in immune cells and may affect TNF-α expression. Thus, we suggest that TNF-α expression will affect the downstream signaling of immune cells. Furthermore, since an expression quantitative loci (eQTL) analysis provided suggestive evidence that rs3914501 in *NAALADL2* and rs6838327 in *YIPF7* upregulate the expression of *IL13, IL22*, and *IL12RB2* in lymphoid cells (Fig. S9 and Supporting information 4, eQTL), we thought that its effects on IL22 would also be feasible in epithelial cells. Consistently, our data showed increased *IL17* mRNA expression levels in the colon tissues of intestinal BD patients along with decreased *IL10* mRNA expression levels, which was well correlated with the decreased protein levels of NAALADL2 and YIPF7 in inflamed cells or tissues, including colonic enterocytes from patients with intestinal BD and IBD, the inflamed mouse colon, and LPS-treated HT-29 cells. Taken together, our results suggest that SNPs of *NAALADL2* and *YIPF7* are candidates as causal variants of intestinal BD development.

Notably, GWAS cannot provide supporting evidence based on more complex patterns of associations with other polymorphisms in the same gene^24^. In addition, other unknown variants in regions showing high linkage disequilibrium of the investigated loci may alter protein expression, translation, or degradation^25^,^26^. Hence, we performed haplotype and pathway analyses to further elucidate the genetic factors involved in intestinal BD pathogenesis. In particular, pathways consisting of combinations of multiple variants with small effects could explain the overall susceptibility to multifactorial diseases that cause the same disease phenotype in complex diseases^15^. It has been reported that HLA-B*51, the genetic risk factor most strongly associated with BD in several populations^2^,^4^,^27^, is associated with a moderately higher prevalence of genital ulcers, ocular manifestations, and skin manifestations, although it showed a decreased prevalence of gastrointestinal involvement in BD^28^ and no relationship with IBD^29^. Similarly, our results showed that the rate of HLA-B51 positivity was significantly higher in BD patients without intestinal involvement than that in intestinal BD patients (36.7% vs. 22.5%, *P* = 0.011, Table 1) in concordance with the genetic results (Table 2). In addition, *HLA-B* genotype showed a stronger correlation with HLA-B*51 positivity in BD patients without intestinal involvement (OR: 56.53, Table S4) than in intestinal BD (OR: 26.07, Table S3). Based on our results and a previous meta-analysis^28^, genotyping of this allele seems to be less associated with intestinal BD and more strongly associated with BD patients without intestinal involvement.

The *IL10* knockout model is one of the best knockout animal models of IBD^30^. *IL10* protects against colonic inflammation^31^,^32^. Concordantly, *IL10* was found to be more responsible for intestinal BD than for BD without intestinal involvement in our genetic association study, including the haplotype analyses. Moreover, the pathway analysis revealed connection networks between the intestinal BD and IBD pathways through *IL10*, and the gene expression analysis showed that the *IL10* expression level was reduced in the inflamed BD colon. In this context, there is strong evidence supporting a potential regulatory function of SNPs in *IL10* by affecting the CREB binding protein (Supporting information 4, RegulomeDB). These results support that *IL10* is an example of a shared genetic pathogenesis factor between IBD and intestinal BD^33^,^34^. The risk alleles in *IL10* and *HLA-B* up-regulate the expression of pro-inflammatory cytokines (Fig. S9 and Supporting information 4), suggesting that many risk variants have multiple regulatory and functional features at their loci and interact with each other. Collectively, our findings strongly suggest both differences and similarities between the pathways of BD with and without intestinal involvement and those of IBD, suggesting overlapping yet distinct genetic architectures for these two diseases.

Haplotype analysis suggested phospholipase C beta 1 (*PLCB1*), transforming growth factor, beta receptor 3 (*TGFBR3*), DDB1 and CUL4 associated factor 12 (*DCAF12*), and schwannomin interacting protein 1 (*SCHIP1*) as potential causal variants of intestinal BD, although they failed to reach significance in the Bonferroni test in a replication study (Supplementary information 4). PLCB1 is the major nuclear PLC-β isozyme, and its expression was reported to be increased in the proliferating crypt compartments of the mouse intestine^35^. Interactions between gut microbiota and intestinal epithelial cells trigger increased DUOX enzymatic activity via PLC-β-dependent production of inositol-1,4,5-trisphosphate^36^. Regulatory and eQTL SNPs of *TGFBR3* (rs1805110 and rs17882828, respectively) were identified in this study (Supporting information 4). Although other groups have reported BD polymorphisms^37^,^38^, *TGFBR3* is also known to function in the development of colon diseases such as cancer^39^,^40^. *DCAF12* was revealed to significantly increase the expression level of the pro-inflammatory genes *CCR1, CCR3, TNF, IL5, IL13, ERAP1,* and *NUDT2*, while decreasing the expression level of *IL10* in eQTL analyses (Figs S8, S9, and Supplementary information 4).

In conclusion, intestinal BD shares common pathogenic pathways with IBD and BD without intestinal involvement, but in a different manner, with distinct genetic architectures. Several loci previously suggested by GWAS^4^,^5^ did not show a significant association with any BD subtypes in this study, which might be due to the multiple factors involved in disease development and the limited sample size. Thus, large multi-center replication studies and additional functional experiments are warranted to validate our findings.

This is the first study elucidating specific genetic polymorphisms contributing to intestinal involvement in BD. Our study provides the first notable evidence that there are specific genetic susceptibility loci associated with intestinal involvement in patients with BD, and that *NAALADL2* and *YIPF7* are strong candidates as causal variants of intestinal BD development. The GWAS results genetically separated intestinal BD and BD without intestinal involvement. We also identified other independent genetic variants associated with the development of intestinal BD, which showed a partial overlap with IBD-associated genetic variants. This study provides new insights into the pathogenic mechanisms of intestinal BD and IBD, which should prove useful for establishing new diagnostic and therapeutic strategies.

## Methods

### Study subjects and DNA extraction

A total of 533 BD patients of Korean descent, including 238 cases of BD without intestinal involvement and 295 cases of intestinal BD, were enrolled from the Behçet’s Disease Clinic of Yonsei University College of Medicine, Severance Hospital, Seoul, Korea between June 2006 and August 2013. Intestinal BD was diagnosed according to established criteria based on colonoscopic features and clinical manifestations^7^. Only patients who were finally classified as “definite” or “probable” types were included in this study. The Institutional Review Board of Severance Hospital, Yonsei University approved this study (IRB approval number: 4-2013-0805). All patients and controls provided written informed consent and all methods were performed in accordance with the relevant guidelines and regulations. Genomic DNA was extracted from whole blood samples, using the DNA blood maxi kit from Qiagen (Santa Clara, CA, USA).

### GWAS

Genotyping was performed on specimens from 199 patients with BD (100 BD patients without intestinal involvement and 99 patients with intestinal BD), using the Affymetrix Whole-Genome-Wide Human SNP Array 6.0 (Affymetrix, Santa Clara, CA, USA). All samples showed call rates of >95% and were finally included in the case–control analysis. After excluding controls with <95% call rates or mismatched sex and subjects who were potential relatives, a total of 597 control samples out of 600 Korean individuals were entered into the case–control analysis. Quality control methods for the GWAS, PCA, and imputation analysis are described in the Supporting Methods, Supporting information 1.

### SNP selection and validation study

Forty-four SNPs, including those derived from our GWAS and those previously reported as significant elsewhere, were validated on the same platform, using an independent cohort of samples from 138 BD patients without intestinal involvement and 196 BD patients with intestinal involvement. The details of SNP selection, validation, and haplotype analysis are described in the Supporting Methods, Supporting Information 1.

### *In vitro* and *in vivo* experiments

The HT-29 cell line (Korean Cell Line Bank, Seoul, Korea) was maintained at 37 °C in Dulbecco’s modified Eagle’s medium supplemented with 10% heat-inactivated fetal bovine serum and 1% antibiotics in a humidified atmosphere of 5% CO_2_.

Knockdown of a specific gene was achieved by 24-h transfection of siRNA or a non-targeting control (AccuTarget, Bioneer, Daejeon, Korea) into HT-29 cells. To assess inflammatory responses, the cell culture medium was replaced with medium containing LPS (1–2 μg/mL) at 24 h post-transfection. Cells were harvested at 3 h for quantitative reverse-transcription polymerase chain reaction (qRT-PCR) analysis and at 24 h for immunostaining after LPS treatment.

Methods for immunohistochemical staining, qRT-PCR, and colitis mouse models are provided in the Supporting methods, Supporting Information 1. All experiments using animals were reviewed and approved by the Institutional Animal Care and Use Committee of Yonsei University Severance Hospital, Seoul, Korea (IACUC Approval No: 2013-0166) and all methods were performed in accordance with the relevant guidelines and regulations.

### *In silico* analyses of SNP functions, networks, and pathways

*In silico* analyses to explore SNP functions are described in the Supporting Methods, Supporting Information 1. To establish the biological relevance of the selected SNPs in disease pathogenesis, the possible functional consequences of the 44 selected SNPs were explored. Biological pathways were analyzed using IPA (ver. 23814503, http://www.ingenuity.com/) to evaluate whether these sub-networks are biologically meaningful by comparing the functional relationships between constituent genes identified from the GWAS. Gene lists from our study and ‘Reported Gene’ lists of BD and IBD from a GWAS catalog (http://www.genome.gov/gwastudies) were entered into the IPA database. Enrichment of focus genes and functional categories were also evaluated in the IPA Knowledge Base. The network score or *P* value represents the significance of focus gene enrichment.

### Statistical analysis

Statistical significance of the association of selected SNPs with a disease or disease subset was determined by the chi-square test. Logistic regression analysis was used to obtain the OR, 95% confidence interval for the OR, and corresponding *P* values between cases and controls regarding the selected SNPs. Significant *P* values in association analysis for combined samples were computed by chi-square and Cochran-Mantel-Haenszel tests. The prognosis of intestinal BD was analyzed by the Kaplan–Meier method with differences determined by the log-rank test. Details are described in the Supporting Methods, Supporting Information 1.

## Additional Information

**How to cite this article**: Kim, S. W. *et al*. Identification of genetic susceptibility loci for intestinal Behçet's disease. *Sci. Rep.* **7**, 39850; doi: 10.1038/srep39850 (2017).

**Publisher's note:** Springer Nature remains neutral with regard to jurisdictional claims in published maps and institutional affiliations.

## Supplementary Material

Supporting Information

## Figures and Tables

**Table 1:** Clinical and demographic characteristics of Behçet’s disease without and with intestinal involvement.

	BD without intestinal involvement (n = 238)	Intestinal BD (n = 295)
Sex, male (%)	62 (26.1)	135 (45.8)
Mean age (years)	41.8 ± 11.8	44.3 ± 12.3
Mean age at diagnosis (years)	35.5 ± 10.8	40.1 ± 11.8
Mean disease duration (years)	5.9 ± 6.9	7.5 ± 5.7
HLA-B51 positivity (%)^A^	83/226 (36.7)	23/102 (22.5)*
Clinical manifestations (%)		
Oral ulcers	238 (100)	273 (92.5)
Genital ulcers	212 (89.1)	154 (52.2)
Eye lesions	76 (31.9)	52 (17.6)
Skin lesions	219 (92.0)	192 (65.1)
Arthritis/arthralgia	134 (56.3)	158 (53.6)
Vascular lesions	10 (4.2)	11 (3.7)
Central nervous system lesions	5 (2.1)	5 (1.7)
Epididymitis	3 (1.3)	0
Intestinal complications (%)		
Perforation	0 (0.0)	28 (9.5)
Fistula	0 (0.0)	26 (8.8)
Stricture	0 (0.0)	25 (8.5)
Abscess	0 (0.0)	13 (4.4)
Medication use (%)		
5-aminosalicylic acid/ sulfasalazine	0 (0.0)	288 (97.6)
Corticosteroids	124 (52.1)	136 (46.1)
Azathiopurine/6-mercaptopurine	24 (10.1)	107 (36.3)
Anti-TNF agent	0 (0.0)	8 (2.7)

BD, Behcet’s disease; HLA, human leukocyte antigen; TNF, tumor necrosis factor; NA, not applicable. *P < 0.05 vs. BD without intestinal involvement.

^A^Data are available for 226 BD patients without intestinal involvement and 102 intestinal BD patients.

**Table 2:** Association results of comparison between patients with intestinal Behçet’s disease and patients with Behçet’s disease without intestinal involvement.

SNP	MA	Locus	Nearby genes†	GWAS	Replicated			Combined			
				*P* Model	a*P* value Model	MAF: BD without intestinal involvement	MAF: Intestinal BD	a*P* value Model	OR (95% CI)	MAF: BD without intestinal involvement	MAF: Intestinal BD
rs284148	T	Chr.1: 92277843	*TGFBR3*	6.4 × 10^−4^ Recessive	6.0 × 10^−1^ Recessive	0.417	0.420	1.7 × 10^−2^ Dominant	0.638 (0.442–0.921)	0.446	0.381
rs16848171	C	Chr. 3: 175181067	*NAALADL2*	4.9 × 10^−3^ Recessive	5.4 × 10^−2^ Recessive	0.195	0.227	7.4 × 10^−3^ Recessive	3.208 (1.367–7.529)	0.217	0.248
rs6838327	A	Chr. 4: 44626846	*YIPF7*	6.2 × 10^−6^ Allelic	4.8 × 10^−1^ Allelic	0.456	0.482	**3.5** × **10**^**−4**^ Allelic	1.567 (1.225–2.005)	0.408	0.519
rs2927615*	A	Chr. 5: 96198202	*ERAP1-ERAP2*	—	5.9 × 10^−2^ Allelic	0.010	0.033	2.0 × 10^−2^ Allelic	2.744 (1.172–6.427)	0.016	0.041
rs12525170	A	Chr. 6: 31099761	*HLA-B*	—	1.5 × 10^−1^ Dominant	0.195	0.161	6.0 × 10^−3^ Dominant	0.595 (0.410–0.862)	0.212	0.151
rs4959053*	A	Chr. 6: 31099577	*HLA-B*	8.2 × 10^−3^ Allelic	3.0 × 10^−1^ Allelic	0.185	0.156	1.8 × 10^−2^ Allelic	0.619 (0.425–0.9)	0.204	0.148
rs7245731	A	Chr. 19: 29975118	*LOC284395*	9.2 × 10^−2^ Recessive	2.6 × 10^−1^ Recessive	0.109	0.125	4.2 × 10^−2^ Recessive	0.113 (0.014–0.925)	0.139	0.127
rs6086653	G	Chr.20: 8838343	*PLCB1*	7.8 × 10^−2^ Recessive	4.7 × 10^−2^ Recessive	0.734	0.668	1.7 × 10^−2^ Recessive	0.467 (0.250–0.874)	0.729	0.707

The combined analysis were performed 238 BD cases without intestinal involvement including 100 samples used in GWAS and 295 intestinal BD samples including 99 samples used in GWAS. Bonferroni-corrected significance level is calculated as 0.05/44 tests (bold). SNP, single nucleotide polymorphism; Chr., chromosome; MA, minor allele; GWAS, genome-wide association study; OR, odds ratio; 95% CI, 95% confidence interval; MAF, minor allele frequency. a*P* value: *P* value from logistic regression analysis adjusted for sex and age.

^*^Discovered loci described previously in BD GWAS. Allele frequencies are presented for the discovery sample.

^†^Nearby genes are defined as the closest genes to the SNP within signal boundary or the closest genes within a 200-kb window.

**Table 3:** Association results of comparison between healthy controls and patients with intestinal Behçet’s disease.

SNP	MA	Locus	Nearby genes†	GWAS	Replicated		Combined			
				*P* Model	a*P* value Model	MAF: Intestinal BD	a*P* value Model	OR (95% CI)	MAF: Intestinal BD	MAF: Healthy control
rs1554286*	G	Chr. 1: 206944233	*IL10*	7.6 × 10^−1^ Codominant	4.0 × 10^−3^ Codominant	0.237	7.6 × 10^−3^ Codominant	0.717 (0.562–0.915)	0.252	0.318
rs1800871*	C	Chr. 1: 206946634	*IL10*	—	5.8 × 10^−3^ Codominant	0.232	9.1 × 10^−3^ Codominant	0.722 (0.564–0.922)	0.245	0.309
rs1518111*	C	Chr. 1: 206944645	*IL10*	—	5.4 × 10^−3^ Allelic	0.234	7.6 × 10^−3^ Allelic	0.717 (0.562–0.916)	0.247	0.313
rs7556581	A	Chr. 1: 116386105	*NHLH2*	7.1 × 10^−5^ Recessive	3.7 × 10^−1^ Recessive	0.379	1.1 × 10^−2^ Recessive	1.731 (1.131–2.649)	0.402	0.350
rs284148	T	Chr.1: 92277843	*TGFBR3*	8.8 × 10^−5^ Dominant	3.5 × 10^−1^ Dominant	0.420	1.2 × 10^−2^ Dominant	0.667 (0.486–0.916)	0.381	0.425
rs16830589	C	Chr.3: 159365432	SCHIP1	2.5 × 10^−5^ Dominant	2.0 × 10^−1^ Dominant	0.204	4.7 × 10^−3^ Dominant	1.579 (1.15–2.167)	0.231	0.182
rs16830581	G	Chr.3: 159362915	SCHIP1	1.5 × 10^−5^ Dominant	3.4 × 10^−1^ Dominant	0.194	7.6 × 10^−3^ Dominant	1.544 (1.122–2.126)	0.225	0.178
rs3914501	G	Chr. 3: 174564668	*NAALADL2*	5.7 × 10^−5^ Recessive	1.6 × 10^−2^ Recessive	0.505	**3.8** × **10**^**−4**^Recessive	1.914 (1.338–2.738)	0.537	0.459
rs16848171	C	Chr. 3: 174564668	*NAALADL2*	4.0 × 10^−5^ Recessive	9.3 × 10^−2^ Recessive	0.227	7.0 × 10^−3^ Recessive	2.462 (1.279–4.739)	0.248	0.219
rs6838327	A	Chr. 4: 44626846	*YIPF7*	4.6 × 10^−3^ Recessive	1.2 × 10^−1^ Recessive	0.482	6.4 × 10^−3^ Recessive	1.654 (1.152–2.376)	0.519	0.468
rs32019	C	Chr. 5: 66702373	*CD180*	–	4.4 × 10^−2^ Codominant	0.551	4.0 × 10^−3^ Codominant	1.265 (1.023–1.563)	0.548	0.487
rs10259514	G	Chr. 7: 36829705	*ELMO1*	4.3 × 10^−5^ Allelic	1.4 × 10^−1^ Allelic	0.277	4.8 × 10^−3^ Allelic	0.706 (0.554–0.899)	0.249	0.320
rs10441723	C	Chr. 9: 34082144	*DCAF12*	4.2 × 10^−5^ Codominant	3.5 × 10^−1^ Codominant	0.295	9.5 × 10^−3^ Codominant	0.725 (0.568–0.924)	0.256	0.321
rs10758242	A	Chr. 9: 34146776	*DCAF12*	2.6 × 10^−5^ Allelic	2.1 × 10^−1^ Allelic	0.283	2.8 × 10^−3^ Allelic	0.689 (0.54–0.88)	0.245	0.320
rs12624809	C	Chr.20: 8822431	*PLCB1*	8.5 × 10^−6^ Dominant	6.5 × 10^−1^ Dominant	0.653	3.0 × 10^−2^ Dominant	1.403 (1.034–1.905)	0.696	0.671
rs6086632	C	Chr.20: 8822931	*PLCB1*	7.8 × 10^−6^ Dominant	7.9 × 10^−1^ Dominant	0.649	3.7 × 10^−2^ Dominant	1.385 (1.019–1.880)	0.694	0.671
rs6086633	T	Chr.20: 8823064	*PLCB1*	6.8 × 10^−6^ Dominant	7.4 × 10^−1^ Dominant	0.652	3.7 × 10^−2^ Dominant	1.383 (1.019–1.876)	0.695	0.671
rs6039302	T	Chr.20: 8831137	*PLCB1*	1.1 × 10^−5^ Dominant	7.1 × 10^−1^ Dominant	0658	3.3 × 10^−2^ Dominant	1.397 (1.028–1.898)	0.701	0.675
rs6086653	G	Chr.20: 8838343	*PLCB1*	3.5 × 10^−5^ Dominant	6.2 × 10^−1^ Dominant	0.668	4.3 × 10^−2^ Dominant	1.371 (1.010–1.862)	0.707	0.688

The combined analysis were performed 391 healthy controls and 295 intestinal BD samples including 99 samples used in GWAS. Bonferroni-corrected significance level is calculated as 0.05/44 tests (bold). SNP, single nucleotide polymorphism; Chr., chromosome; MA, minor allele; GWAS, genome-wide association study; OR, odds ratio; 95% CI, 95% confidence interval; MAF, minor allele frequency. a*P* value: *P* value from logistic regression analysis adjusted for sex and age.

^*^Discovered loci described previously. Allele frequencies are presented for the discovery sample.

^†^Nearby genes are defined as the closest genes to the SNP within signal boundary or the closest genes within a 200-kb window.

**Table 4:** Association results of comparison between healthy controls and patients with Behçet’s disease without intestinal involvement.

SNP	MA	Locus	Nearby genes†	GWAS	Replicated		Combined			
				*P* Model	a*P* value Model	MAF: BD without intestinal involvement	a *P* value Model	OR (95% CI)	MAF: BD without intestinal involvement	MAF: Healthy Control
rs6838327	A	Chr. 4: 44626846	*YIPF7*	4.4 × 10^−4^ Dominant	3.6 × 10^−1^ Dominant	0.415	3.1 × 10^−2^ Dominant	0.630 (0.442–0.897)	0.456	0.468
rs32019	C	Chr. 5: 66702373	*CD180*	4.0 × 10^−1^ Recessive	1.2 × 10^−2^ Recessive	0.474	4.0 × 10^−2^ Recessive	0.658 (0.442–0.98)	0.461	0.487
rs4959053*	A	Chr. 6: 31099577	*HLA-B*	2.2 × 10^−5^ Allelic	4.6 × 10^−3^ Allelic	0.207	**6.6** × **10**^**−5**^Allelic	1.892 (1.383–2.589)	0.185	0.119
rs12525170*	A	Chr. 6: 31099761	*HLA-B, PSORS1C1*	—	**1.1** × **10**^**−5**^Dominant	0.213	**1.3** × **10**^**−5**^Dominant	2.210 (1.546–3.157)	0.195	0.119
rs10259514	G	Chr. 7: 36829705	*ELMO1*	1.9 × 10^−2^ Codominant	1.7 × 10^−2^ Codominant	0.257	1.7 × 10^−2^ Codominant	0.724 (0.556–0.944)	0.245	0.320

The combined analysis was performed on 391 healthy controls and 238 BD cases without intestinal involvement, including 100 samples used in GWAS. SNP, single nucleotide polymorphism; Chr., chromosome; MA, minor allele; GWAS, genome-wide association study; OR, odds ratio; 95% CI, 95% confidence interval; MAF, minor allele frequency. a*P* value: *P* value from logistic regression analysis adjusted for sex and age.

^*^Discovered loci described previously. Allele frequencies are presented for the discovery sample.

^†^Nearby genes are defined as the closest genes to the SNP within signal boundary or the closest genes within a 200-kb window. Bonferroni-corrected significance level is calculated as 0.05/44 tests (bold).
