# Supplementary material for: Identification of genetic susceptibility loci for intestinal Behçet’s disease
Source: Sci Rep. 2017 Jan 3;7:39850. doi: 10.1038/srep39850 (PMC5206652; doi:10.1038/srep39850)
Supplement: Supporting Information [file srep39850-s1.pdf]

Supporting Information

Identification of genetic susceptibility loci for intestinal Behçet's disease

Seung Won Kim[1,2,3#], Yoon Suk Jung[4#], Jae Bum Ahn[5], Eun-Soon Shin[6], Hui Won Jang[1], Hyun Jung Lee[1], Tae Il Kim[1], Do Young Kim[7], Dongsik Bang[8], Won Ho Kim[1*], and Jae Hee Cheon[1,2,3*]

[1]Department of Internal Medicine and Institute of Gastroenterology, Yonsei University College of Medicine, Seoul, Korea

[2]Severance Biomedical Science Institute, Yonsei University College of Medicine, Seoul, Korea

[3]Brain Korea 21 PLUS Project for Medical Science, Yonsei University College of Medicine, Seoul, Korea

[4] Division of Gastroenterology, Department of Internal Medicine, Kangbuk Samsung Hospital, Sungkyunkwan University School of Medicine, Seoul, Korea

[5]Department of Medicine, Yonsei University College of Medicine, Seoul, Korea

[6]Bioinformatics team, DNA Link Inc., Seoul, Korea

[7]Department of Dermatology and Cutaneous Biology Research Institute, Yonsei University College of Medicine, Seoul, Korea

[8]Department of Dermatology, Catholic Kwandong University International St. Mary's Hospital, Seoul, Korea

Supporting Information 1: Supplementary Tables, Supplementary Figures, Supporting Methods, Supporting results, and References

Table S1. Statistical power calculation

MAF	Minimum odds ratio
0.05	1.71
0.10	1.50
0.15	1.42
0.20	1.37
0.25	1.35
0.30	1.33
0.35	1.31
0.40	1.30
0.45	1.30
0.50	1.30

We calculated the statistical power to detect a given allelic disease association (for carriership of the rarer SNP allele) in the validation (559 cases and 391 controls) with Quanto v1.2.4. Calculations were performed for different allele frequencies. The power is given as a function of the odds ratio.

Table S2. Haplotype analysis of susceptible genes for intestinal Behçet's disease and Behçet's disease without intestinal involvement

Closest genes	Haplotype	*P* value	Model	OR (95% CI)	MAF	
iBD (n = 295) *vs.* sBD (n = 264)					iBD	sBD
NAALADL2:						
rs16848171, rs9866564	C-G	1.7×10^{-2}	Recessive	3.401 (1.245–9.296)	0.214	0.160
HLA-B :						
rs7742033, rs4959053, rs12525170	G-A-A	9.7×10^{-3}	Dominant	0.617 (0.428–0.889)	0.149	0.206
iBD (n = 295) *vs.* HC (n = 391)					iBD	HC
DCAF12:						
rs10441723, rs10758242	C-A	2.4×10^{-3}	Dominant	0.619 (0.454–0.843)	0.241	0.317
IL10:						
rs1554286, rs1518111, rs1800871	G-C-C	7.3×10^{-3}	Codominant	0.712 (0.555–0.913)	0.243	0.310
PLCB1						
rs12624809, rs6086632, rs6086633, rs6039302, rs6086653	C-C-T-T-G	1.5×10^{-2}	Recessive	1.465 (1.077–1.993)	0.691	0.661
SCHIP1:						
rs16830581, rs16830589	G-C	3.2×10^{-3}	Dominant	1.613 (1.174–2.217)	0.228	0.177
TGFBR3:						
rs17882828, rs284148, rs1805110	C-C-G	2.2×10^{-2}	Recessive	1.481 (1.059–2.070)	0.562	0.526
sBD (n = 264) vs. HC (n = 391)					sBD	HC
Loci nearby *HLA-B:*						
rs7742033, rs4959053, rs12525170	G-A-A	2.4×10^{-5}	Allelic	1.935 (1.424–2.628)	0.206	0.118

SNPs were excluded if they had a minor allele frequency (MAF) <0.05 and SNP distances >100 kb. Risk alleles are underlined. HC, healthy control; iBD, intestinal Behçet's disease; OR, odds ratio; sBD, Behçet's disease without intestinal involvement; 95% CI, 95% confidence interval. Underline shows risk allele.

Table S3. Intestinal complications according to SNP genotypes in patients with intestinal Behçet's disease

Phenotype	Nearby gene<br>rs number		Genotype<br>or allele	Patients with<br>phenotype (%)	Patients without<br>phenotype (%)	OR<br>(95% CI)	*P* value
HLA-B51 positivity	*HLA-B*	Dominant	GG	5 (22.7)	69 (88.5)	26.07	<0.001
	rs4959053		AG/AA	17 (77.3)	9 (11.5)	(7.73-87.87)	
Fistula	*DCAF12*	Dominant	GG	19 (79.2)	142 (55.7)	0.33	0.033
	rs10758242		AG/AA	5 (20.8)	113 (44.3)	(0.12–0.91)	
Stricture	*TGFBR3*	Allele	T	26 (59.1)	192 (36.5)	2.51	0.004
	rs284148		C	18 (40.9)	334 (63.5)	(1.34–4.70)	

OR, odds ratio; CI, confidence interval. Underline shows risk allele.

Table S4. Clinical manifestations according to SNP genotypes in Behçet's disease patients without intestinal involvement

Phenotype	Nearby gene rs number		Genotype or allele	Patients with phenotype (%)	Patients without phenotype (%)	OR (95% CI)	*P* value
HLA-B51 positivity	*HLA-B*	Dominant	GG	11 (11.9)	128 (90.8)	59.53	<0.001
	rs4959053		AG/AA	68 (86.1)	13 (9.2)	(25.26-140.30)	
Central nervous	*HLA-B*	Recessive	GG/AG	3 (60.0)	218 (96.5)	22.91	0.002
system lesions	rs4959053		AA	2 (40.0)	8 (3.5)	(3.02-174.09)	

OR, odds ratio; 95% CI, 95% confidence interval. Underline shows risk allele.

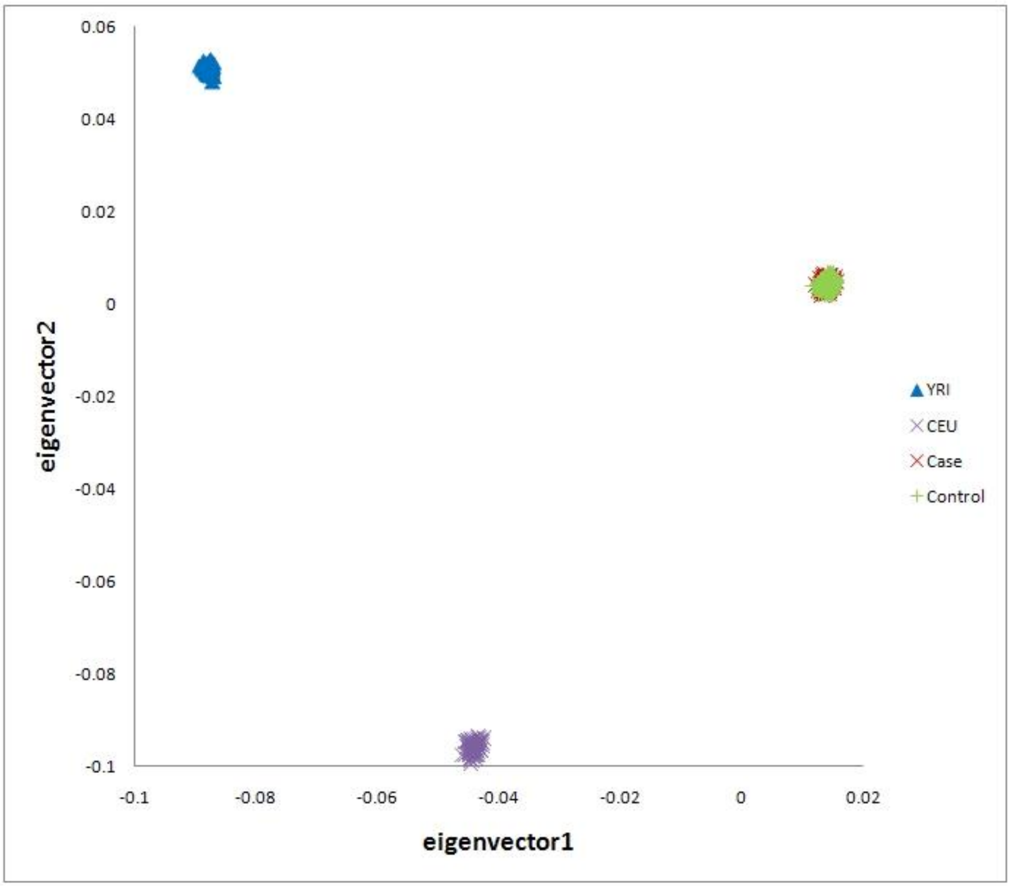

Figure S1. Principal Component Analysis (PCA). PCA of samples from all participants (199 BD cases and 597 controls) along with reference samples from 1000 Genomes Project. Behçet's disease (BD) cases are shown in red, controls are shown in green, Utah residents with ancestry from northern and western Europe (CEU) are shown in purple, and Yoruba from Ibadan, Nigeria (YRI) are shown in dark blue.

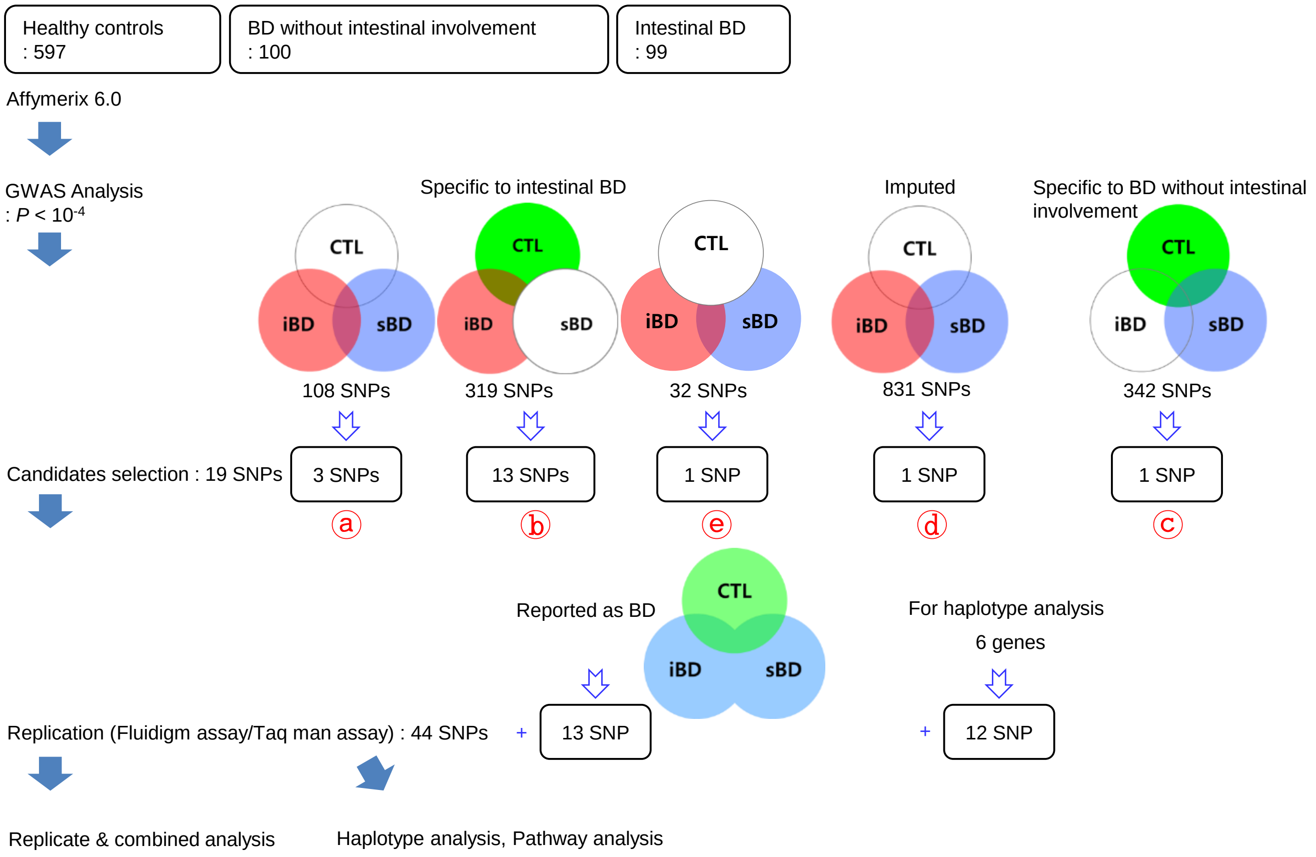

Figure S2. Workflow of this study. ⓐ intestinal BD vs. BD without intestinal involvement, ⓑ intestinal BD-specific loci (not included in the significant regions of BD without intestinal involvement) vs. healthy controls, ⓒ BD patients without intestinal involvement vs. healthy control group, ⓓ imputated intestinal BD vs. BD without intestinal involvement, ⓔ intestinal BD vs. BD without intestinal involvement among BD-specific SNPs. CTL, healthy control; iBD, intestinal Behçet's disease; sBD, systemic BD without intestinal involvement.

a

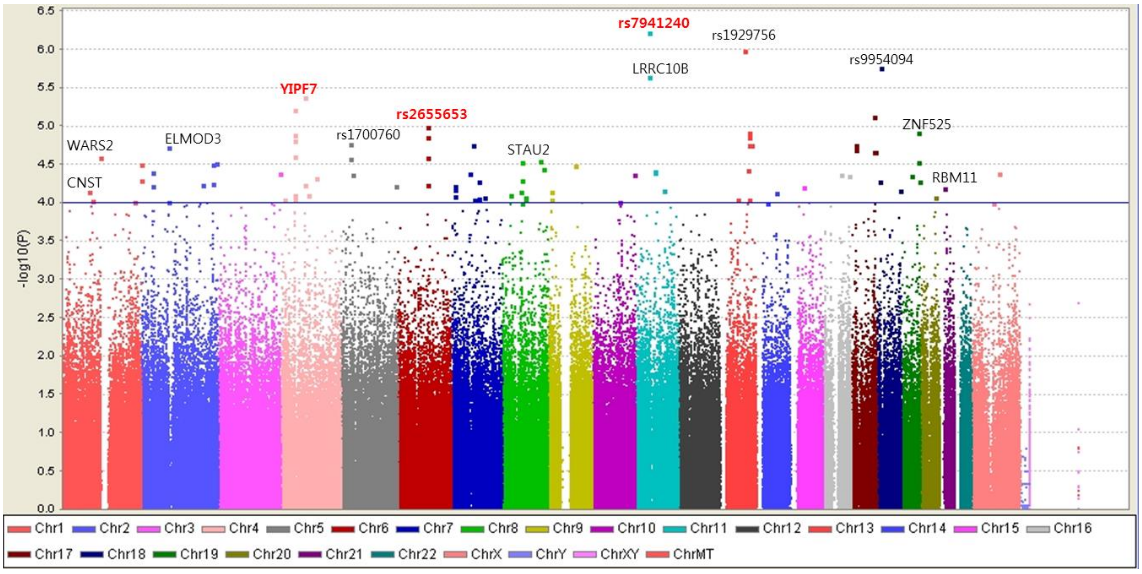

rs7941240
rs1929756
rs9954094
LRRC10B
YIPF7
rs2655653
ZNF525
rs1700760
ELMOD3
WARS2
STAU2
RBM11
CNST
-log10(P)
Chr1 Chr2 Chr3 Chr4 Chr5 Chr6 Chr7 Chr8 Chr9 Chr10 Chr11 Chr12 Chr13 Chr14 Chr15 Chr16
Chr17 Chr18 Chr19 Chr20 Chr21 Chr22 ChrX ChrY ChrXY ChrMT

b

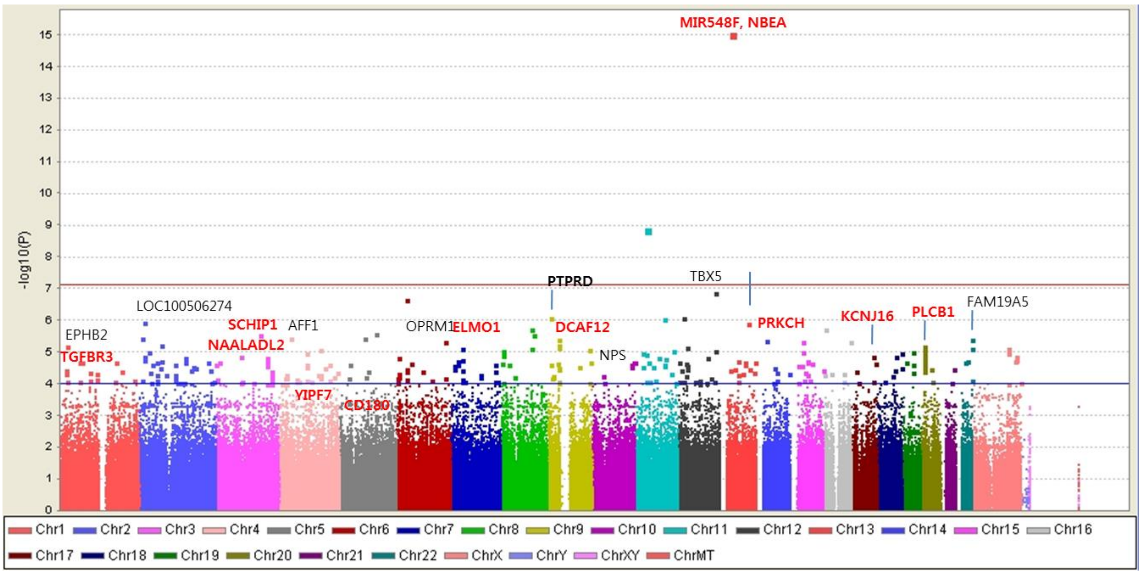

MIR548F, NBEA
PTPRD
TBX5
LOC100506274
FAM19A5
KCNJ16
PLCB1
PRKCH
EPHB2
SCHIP1
AFF1
OPRM1
ELMO1
DCAF12
NAALADL2
TGFBR3
NPS
YIPF7
CD180
-log10(P)
Chr1 Chr2 Chr3 Chr4 Chr5 Chr6 Chr7 Chr8 Chr9 Chr10 Chr11 Chr12 Chr13 Chr14 Chr15 Chr16
Chr17 Chr18 Chr19 Chr20 Chr21 Chr22 ChrX ChrY ChrXY ChrMT

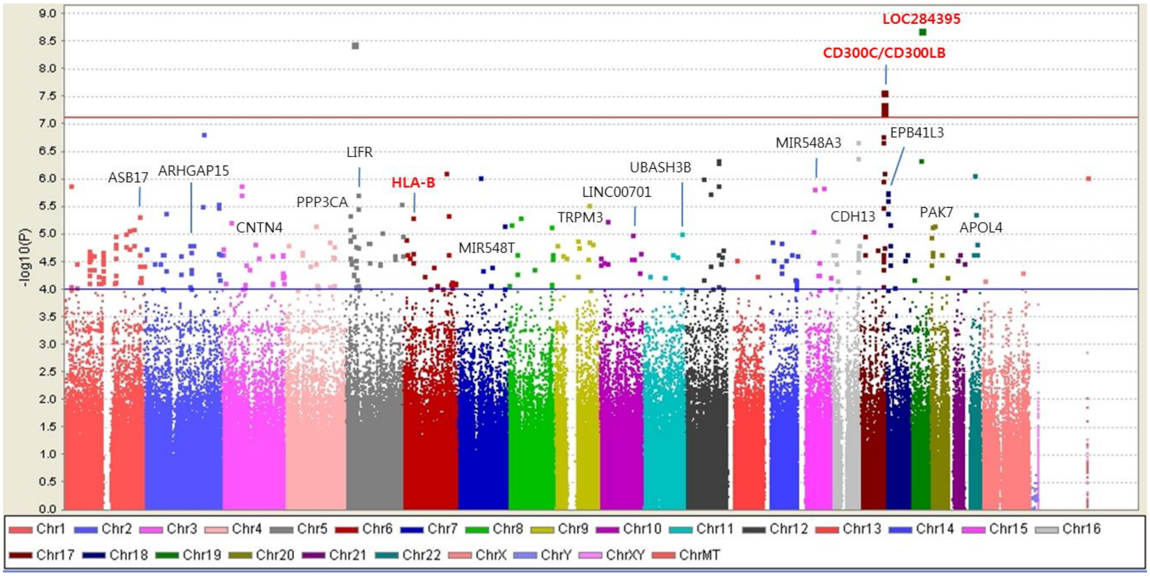

d

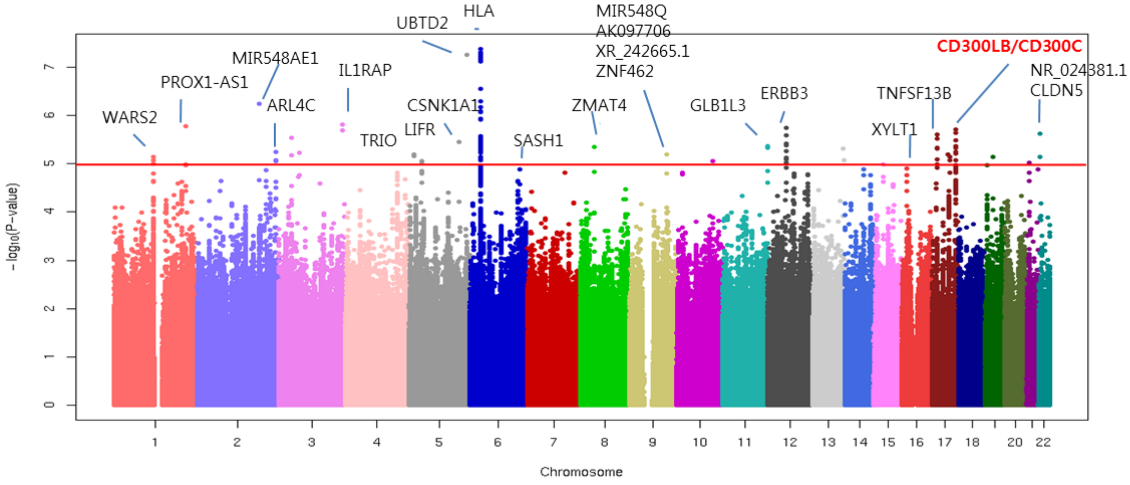

Figure S3. Manhattan plots. Manhattan plot of the comparison between patients with intestinal BD *vs*. without intestinal involvement (**a**), comparison between patients with intestinal BD *vs.* healthy controls (**b**), comparison between BD patients without intestinal involvement *vs*. healthy controls (**c**), and imputed comparison between patients with intestinal BD *vs*. healthy controls (**d**). Blue line indicates $P < 10^{-4}$, brown line indicates Bonferroni corrected P value, and red letter indicates validated

SNPs in this study. BD, Behçet’s disease.

a

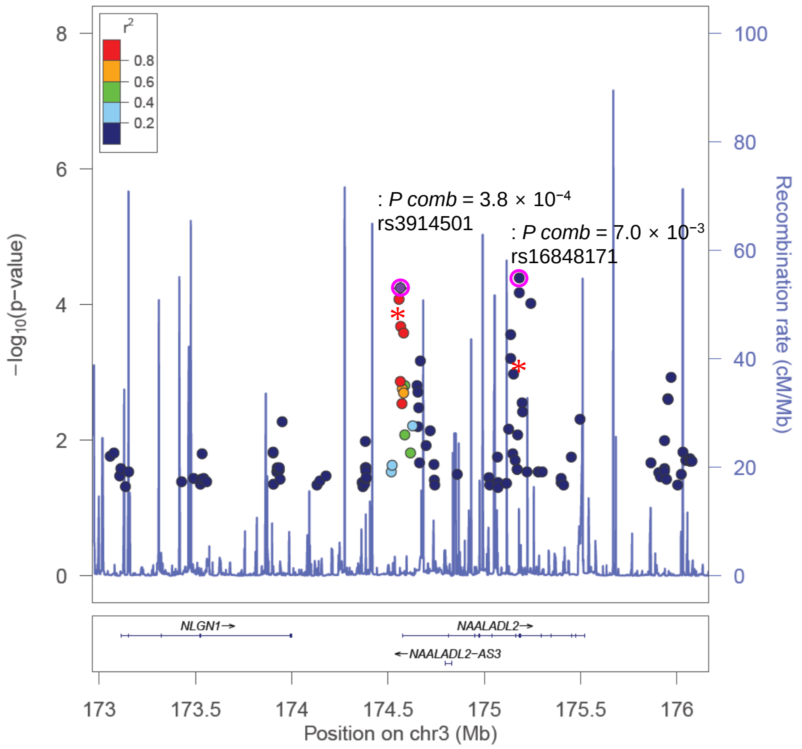

r2
0.8
0.6
0.4
0.2
: P comb = 3.8 × 10−4
rs3914501
: P comb = 7.0 × 10−3
rs16848171
−log10(p−value)
Recombination rate (cM/Mb)
NLGN1
NAALADL2
NAALADL2−AS3
Position on chr3 (Mb)

b

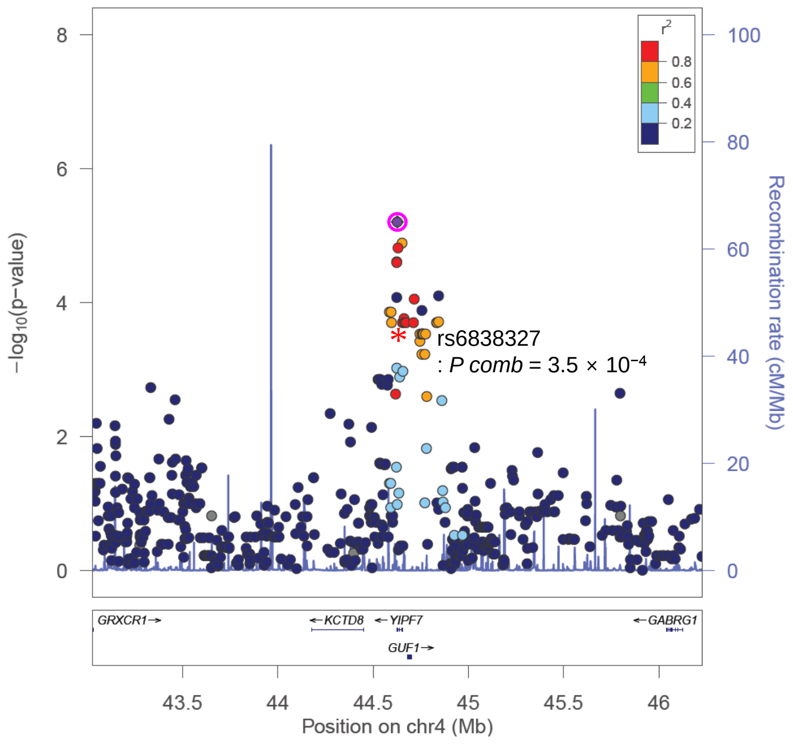

r2
0.8
0.6
0.4
0.2
rs6838327
: P comb = 3.5 × 10−4
−log10(p−value)
Recombination rate (cM/Mb)
GRXCR1
KCTD8
YIPF7
GUF1
GABRG1
Position on chr4 (Mb)

c

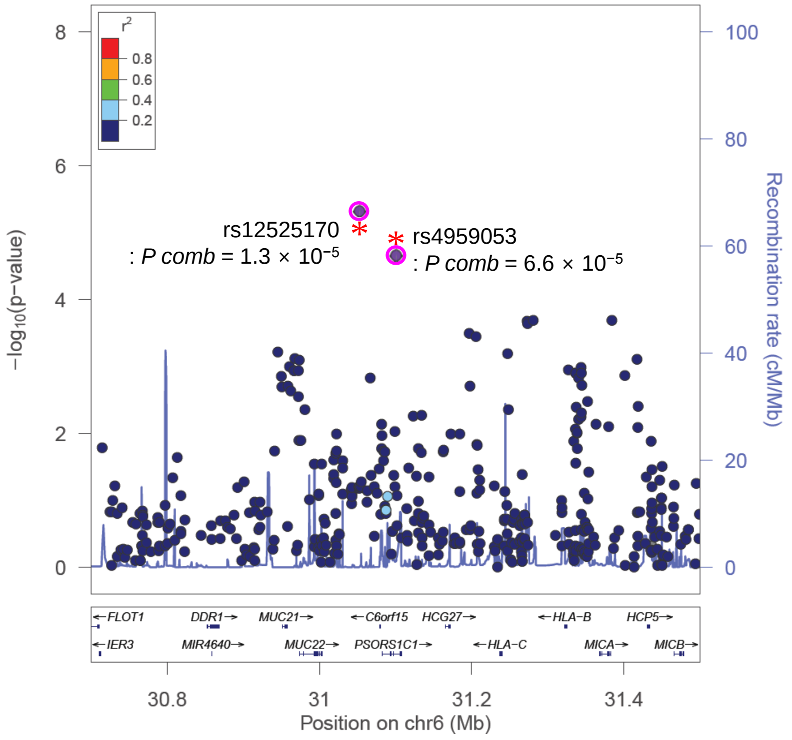

Figure S4. Regional association plots. Regional association plots from GWAS ($-\log_{10}$ *P* values; top) and gene loci (bottom). (**a**) rs3914501 (intestinal BD *vs*. healthy controls), (**b**) rs6838327 (BD without intestinal involvement *vs*. intestinal BD), (**c**) rs4959053 (BD without intestinal involvement *vs*. healthy controls). Single nucleotide polymorphisms (SNPs) are plotted using the estimated recombination rates from hg19/1000 Genomes Asian groups (December 2012). The validated SNPs and $-\log_{10}$ *P* values are shown by pink circles and red asterisks, respectively. Plots were generated using LocusZoom. BD, Behçet's disease.

a

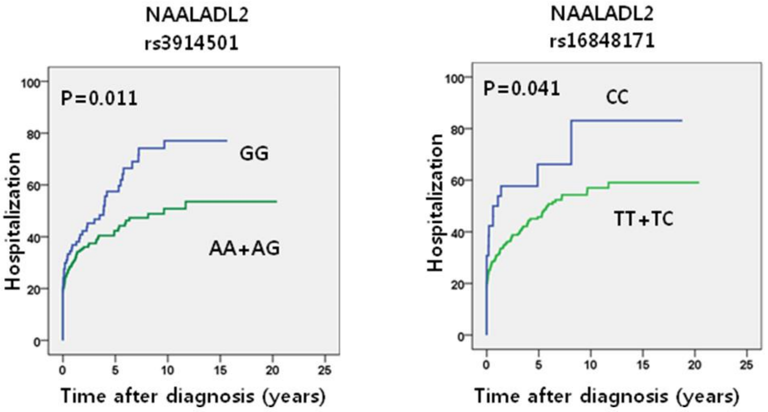

b

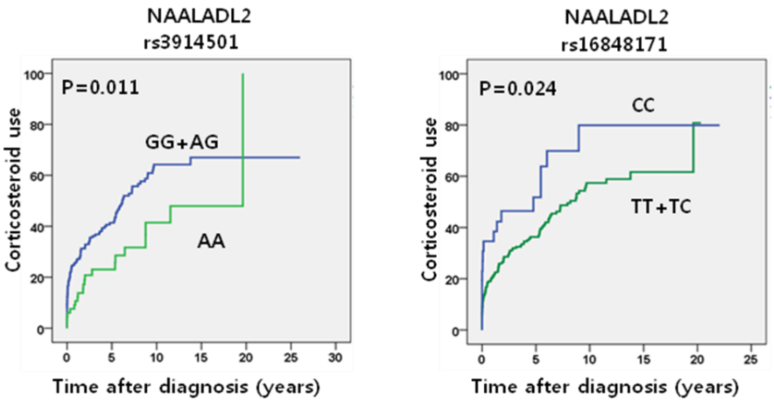

Figure S5. Cumulative probabilities of hospitalization. (a) and corticosteroid use (b) according to SNP genotype for patients with intestinal Behçet’s disease. Blue lines show risk alleles.

a

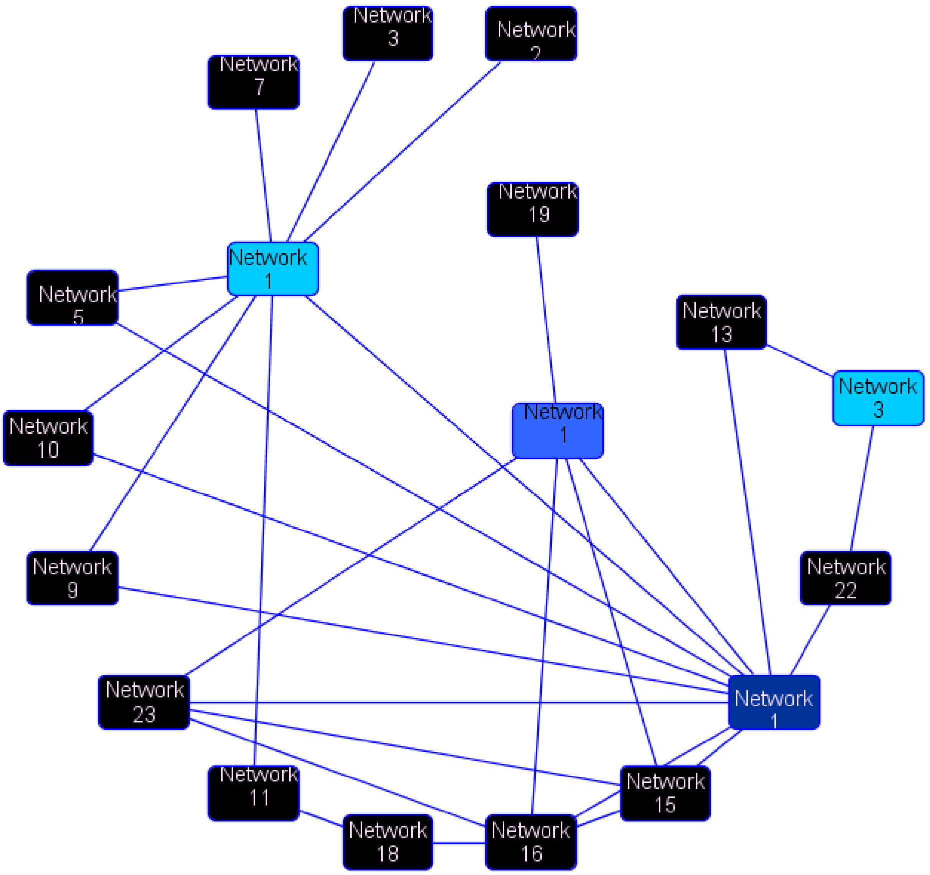

Network 3
Network 2
Network 7
Network 19
Network 1
Network 5
Network 13
Network 3
Network 1
Network 10
Network 9
Network 22
Network 23
Network 1
Network 11
Network 15
Network 18
Network 16

b

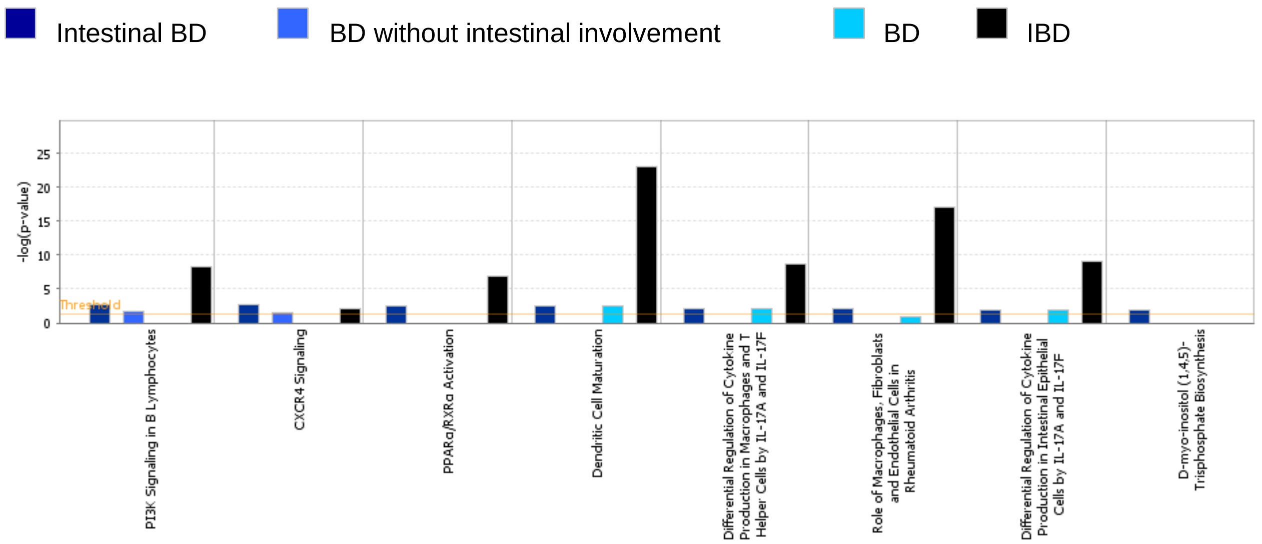

Intestinal BD
BD without intestinal involvement
BD
IBD
-log(p-value)
25
20
15
10
5
0
Threshold
PI3K Signaling in B Lymphocytes
CXCR4 Signaling
PPARα/RXRα Activation
Dendritic Cell Maturation
Differential Regulation of Cytokine Production in Macrophages and T Helper Cells by IL-17A and IL-17F
Role of Macrophages, Fibroblasts and Endothelial Cells in Rheumatoid Arthritis
Differential Regulation of Cytokine Production in Intestinal Epithelial Cells by IL-17A and IL-17F
D-myo-inositol (1,4,5)-Trisphosphate Biosynthesis

c

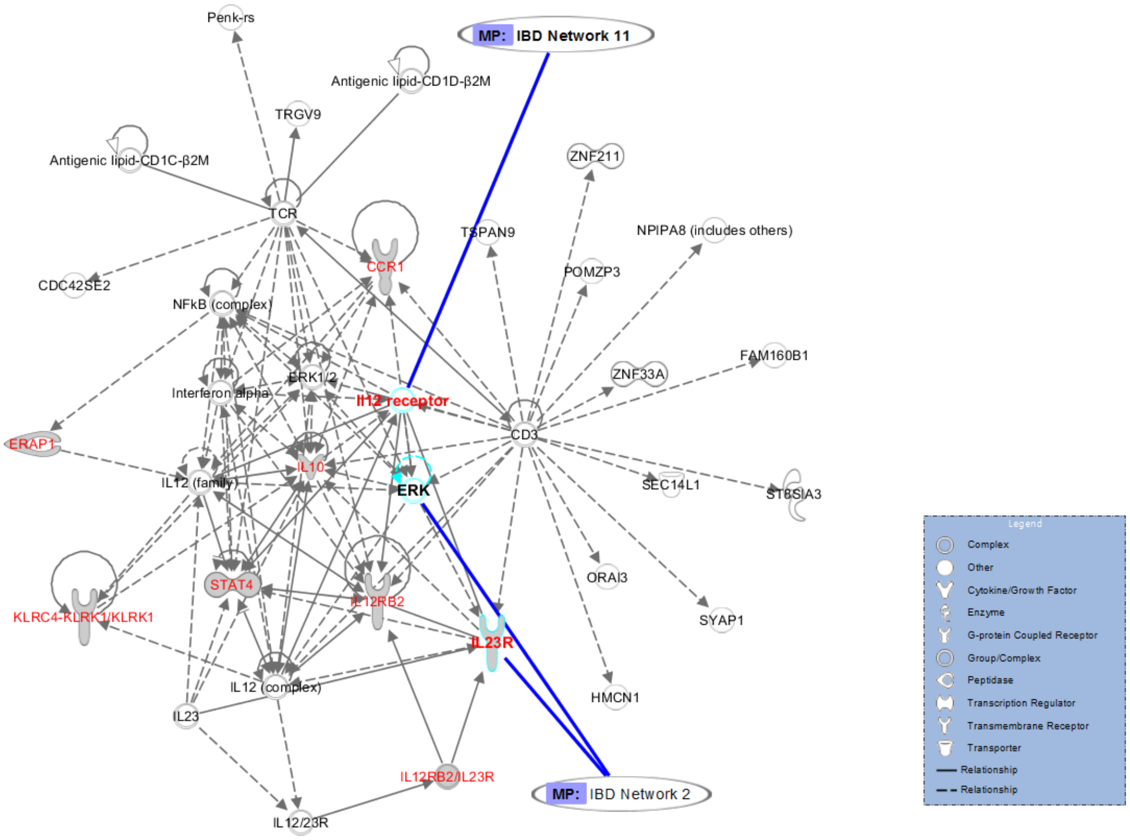

Figure S6. Network Diagram of Behçet's disease susceptible genes. (**a**) Networks of overlap among intestinal BD (dark blue), BD without intestinal involvement (blue), and inflammatory bowel disease (IBD, black). (**b**) Comparison of pathways for intestinal BD, BD without intestinal involvement, and inflammatory bowel disease. BD, Behçet's disease; IBD, inflammatory bowel disease. Orange color shows threshold. (**c**) Networks of overlap between BD without intestinal involvement and IBD. Network 1 of BD is linked to Network 2 and Network 11 of IBD networks.

a

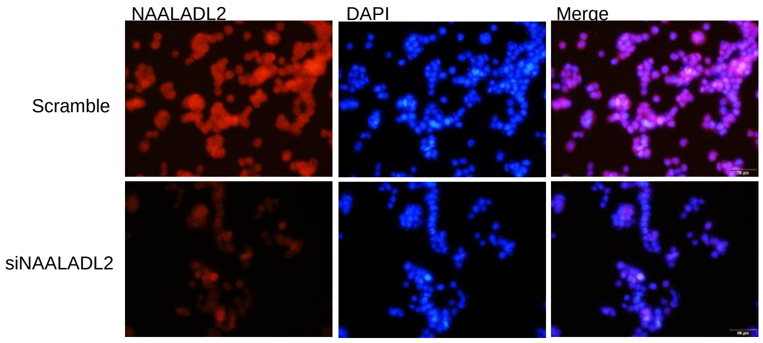
NAALADL2
DAPI
Merge
Scramble
siNAALADL2

b

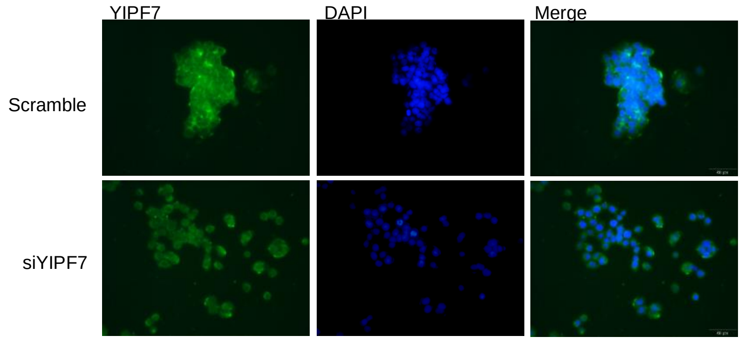
YIPF7
DAPI
Merge
Scramble
siYIPF7

c

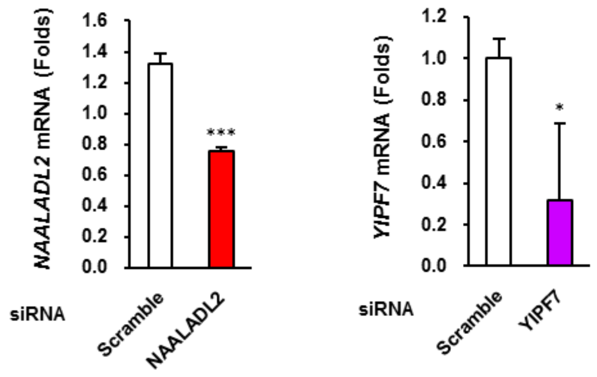
NAALADL2 mRNA (Folds)
1.6
1.4
1.2
1.0
0.8
0.6
0.4
0.2
0.0
***
siRNA
Scramble
NAALADL2
YIPF7 mRNA (Folds)
1.2
1.0
0.8
0.6
0.4
0.2
0.0
*
siRNA
Scramble
YIPF7

Figure S7. ***NAALADL2*** **and YIPF7 silencing was performed using short interfering RNA in HT-29 cells.** Expression analysis was performed at 24 h post transfection with small interfering (siRNA) against *NAALADL2* and *YIPF7*. The protein (**a, b**) and mRNA (**b**) levels were evaluated by immunofluorescent staining and quantitative reverse transcription polymerase chain reaction, respectively. Blue, DAPI; red, NAALADL2; green, YIPF7. Data represent mean ± SEM. (n = 3).

(**a**) rs2927615 in *ERAP1*

ERAP1

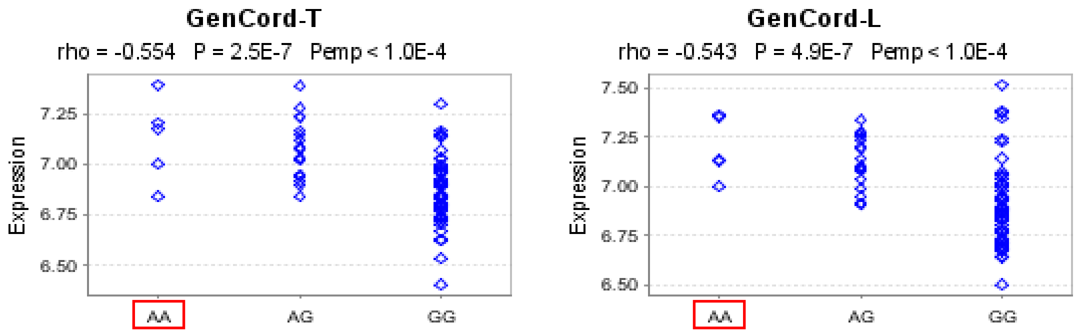

(**b**) rs10758242 in *DCAF12*

NUDT2 ***ERAP1****

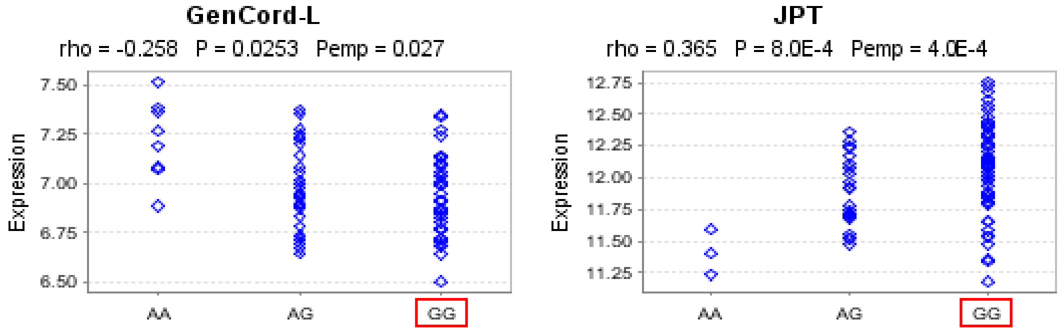

Figure S8. Expression quantitative trait loci (eQTL) analysis. (**a**) rs2927615 in *ERAP1*. (**b**) rs10758242 in *DCAF12*. Results were plotted from probes located at each SNP of HapMap3 (CEU, CHB, GIH, JPT, LWK, MEX, MKK, YRI; Stranger *et al.*, 2012) and Geneva Gencord (Dimas *et al.*, 2009) studies. A: adipose tissue; F, fibroblast; T, T-cell; S, skin; F, fat; L, lymphoblastoid cell line. Asterisks and red boxes show significant eQTLs among candidate SNPs for BD after 10,000

permutations and risk alleles, respectively. Red boxes indicate risk alleles.

(**a**) *IL22*

rs3914501 (*NAALADL2*)

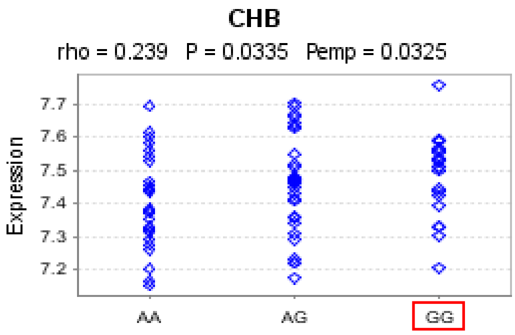

rs4959053 (*HLA-B*)

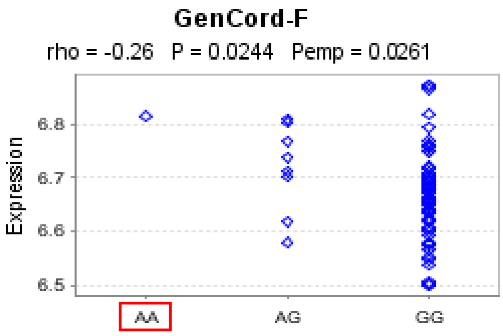

rs2927615 (*ERAP1-ERAP2*)

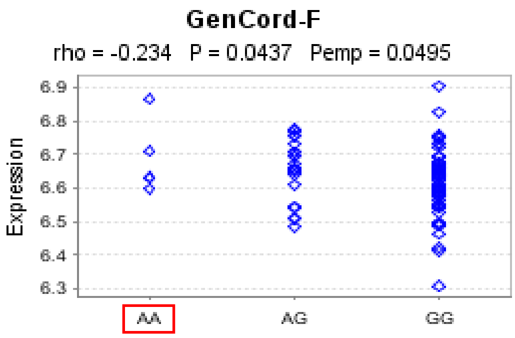

(**b**) *IL5*

rs4959053 (*HLA-B*)

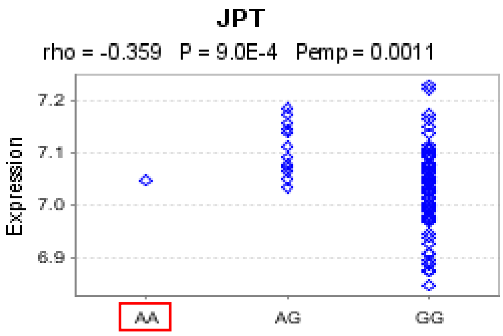

rs10758242 (*DCAF12*)

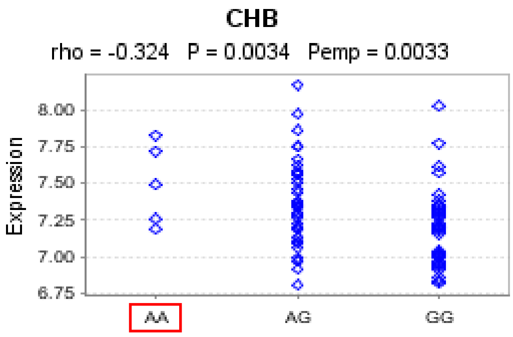

(**c**) *NOD2*

rs1518111 (*IL10*)

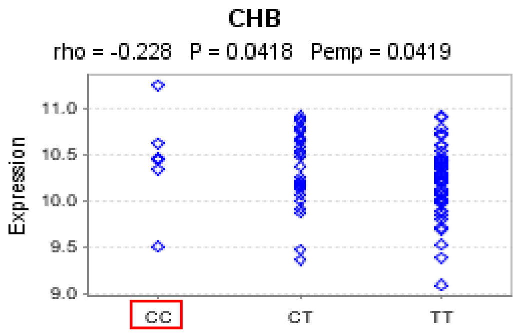

rs1554286 (*IL10*)

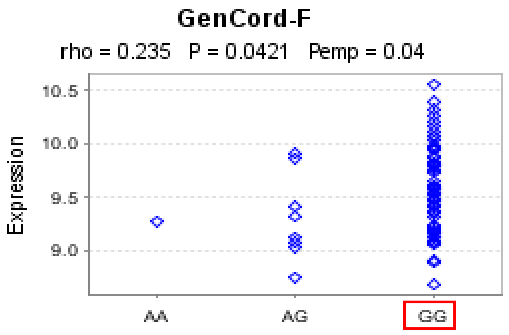

rs1805110 (*TGFBR3*)

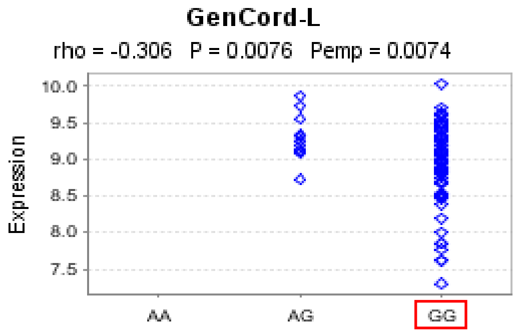

rs4959053 (*HLA-B*)

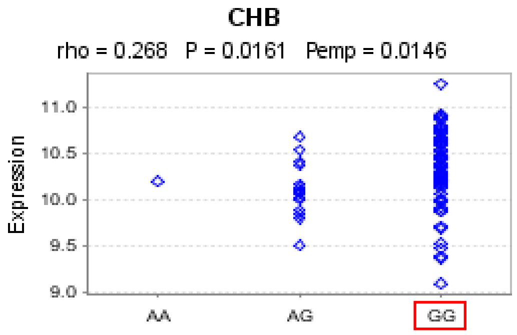

(**d**) *YIPF7*

rs16830581 (*SCHIP1*)

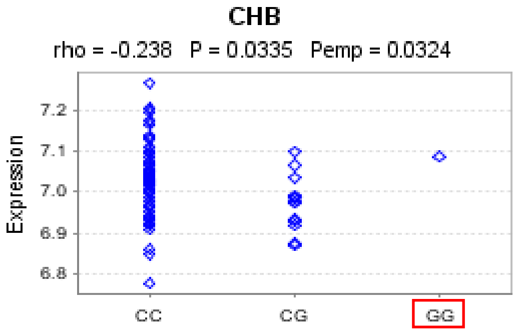

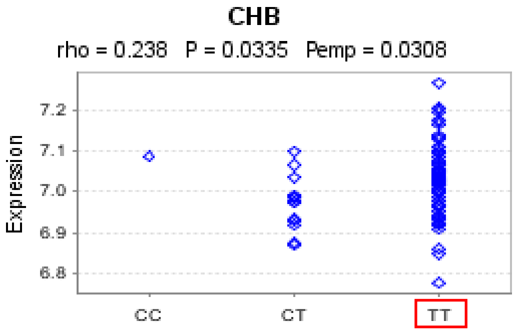

(**e**) *TNF*

rs10441723 (*DCAF12*)

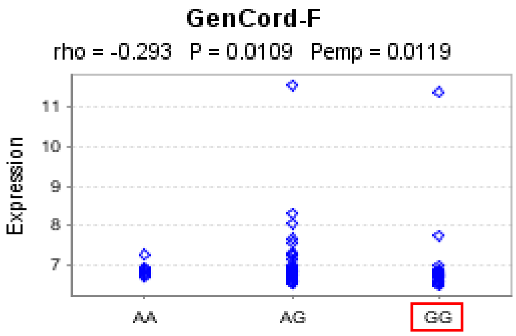

(**f**) *IL23R*

rs1805110 (*TGFBR3*)

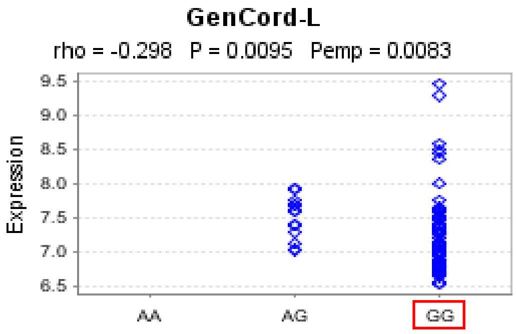

rs4959053 (*HLA-B*)

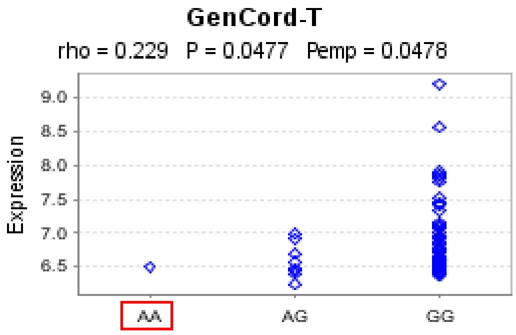

(**g**) *IL12B*

rs1805110 (*TGFBR3*)

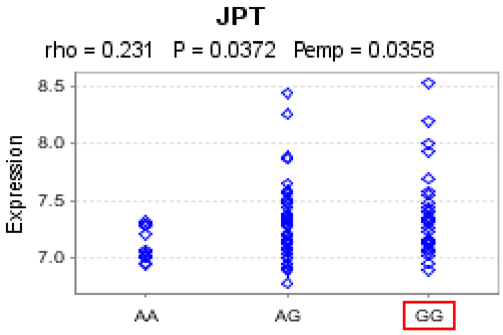

rs284148 (*TGFBR3*)

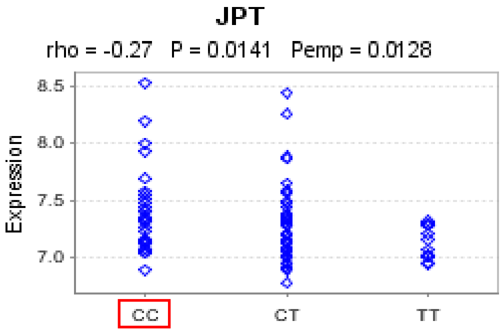

rs2927615 (*ERAP1-ERAP2*)

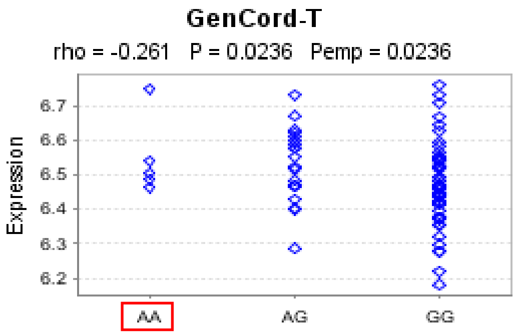

(**h**) *IL10*

rs1518111 (*IL10*)

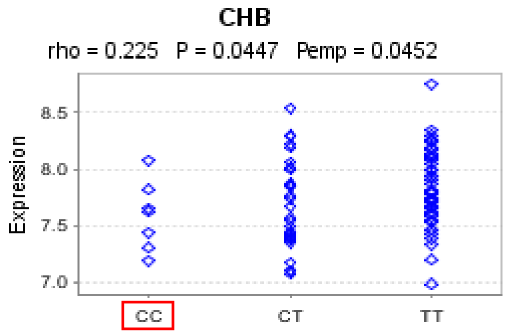

rs1554286 (*IL10*)

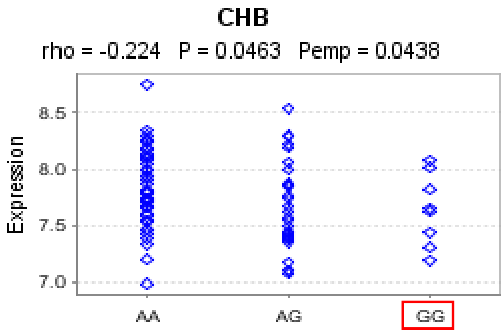

rs284148 (*TGFBR3*)

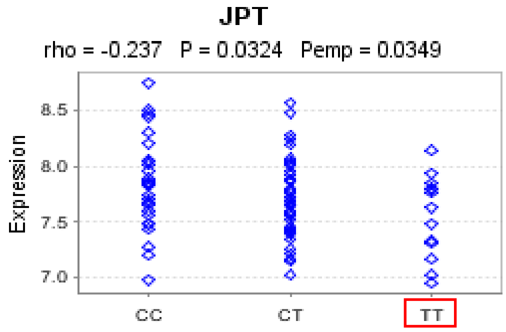

rs10441723 (*DCAF12*)

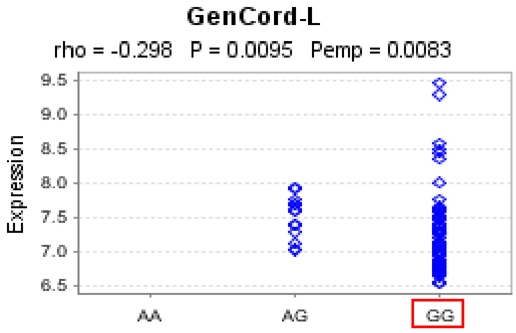

rs1554286 (*IL10*)

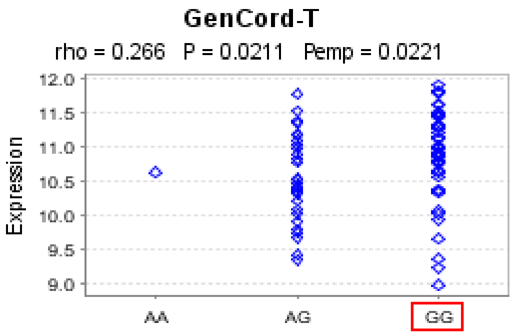

rs2927615 (*ERAP1-ERAP2*)

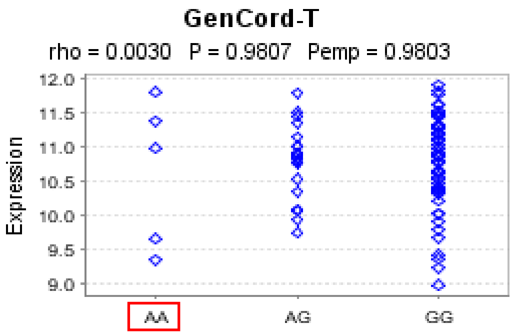

(**i**) *IL13*

rs3914501 (*NAALADL2*)

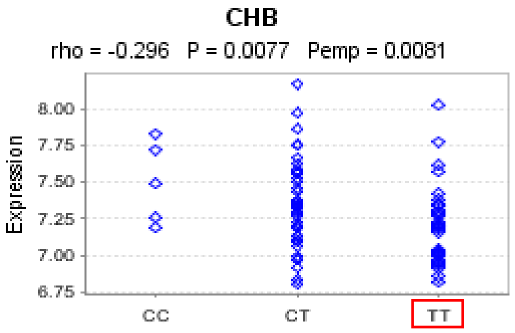

rs10758242 (*DCAF12*)

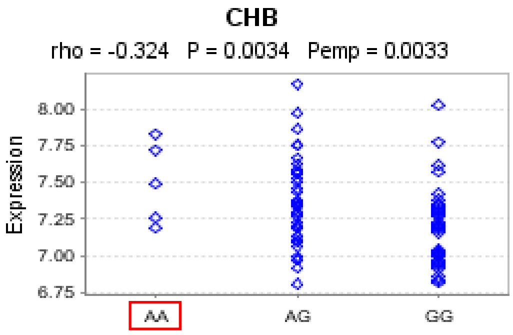

(**j**) *TGFB1*

rs284148 (*TGFBR3*)

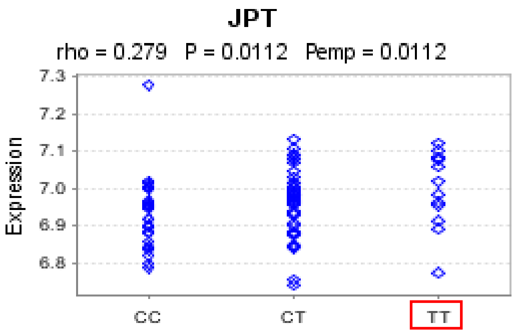

(**p**) *PSORS1C1*

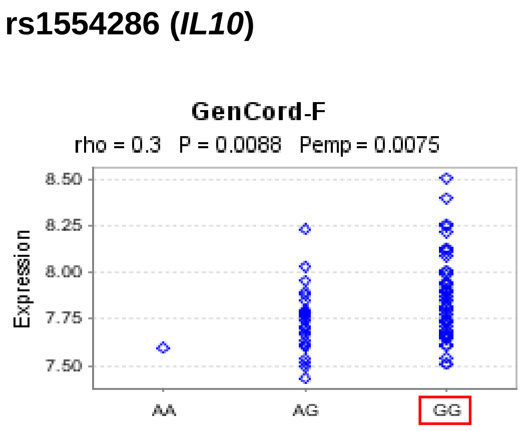

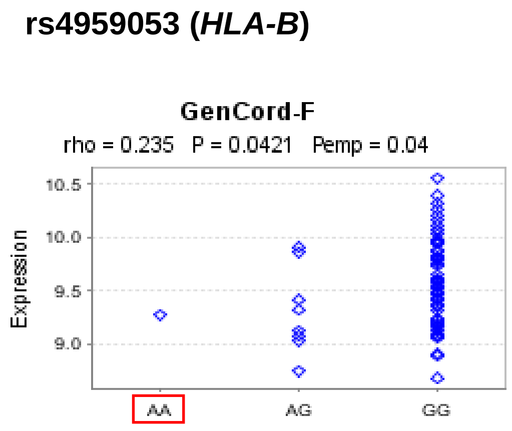

Figure S9. SNP-gene association analysis of associated SNPs. (**a**) *IL22*. (**b**) *IL5*. (**c**) *NOD2*. (**d**) *YIPF7*. (**e**) *TNF*. (**f**) *IL23R*. (**g**) *IL12B*. (**h**) *IL10*. (**i**) *IL13*, (**j**) *TGFB1*, (**k**) *PSORS1C1*. Results were obtained from cis-probes located ± 1,000 kb from significant SNPs of HapMap3 (CHB, JPT) (Stranger *et al*., 2012) and Geneva Gencord (Dimas *et al*., 2009) studies. Chr., chromosome; eQTL, expression quantitative trait loci; LD, linkage disequilibrium; Position, chromosome position (hg19); rSNP, regulatory SNP; F, fibroblast; T, T-cell; S, skin; F, fat; L, lymphoblastoid cell line. *P* values were obtained by 10,000 permutations. Red boxes indicate risk alleles.

Supporting Methods

Quality control in GWAS, imputation, and Principal component analysis (PCA)

For quality control, SNPs with *P* values $>1 \times 10^{-4}$, those that were not mapped to autosomal chromosomes in cases or controls, those with <1% minor allele frequency, or those with *P* values $<1 \times 10^{-7}$ by Hardy-Weinberg equilibrium (HWE) in controls were excluded from the analysis. A quantile–quantile plot (Q–Q plot) was obtained to evaluate the overall significance of genome-wide associations and the potential impact of population stratification. After quality control and filtering processes, the genotype data for 1) 637,816 SNPs between patients with intestinal BD *vs.* BD patients without intestinal involvement, 2) 646,355 SNPs between BD patients without intestinal involvement *vs.* healthy controls, and 3) 60,137 SNPs between patients with intestinal BD *vs.* healthy controls, excluding SNPs significant in BD patients without intestinal involvement were used for the final GWAS analysis (Fig. S2). A Manhattan plot of $-\log_{10} P$ was created using Haploview (V.4.2, http://www.broadinstitute.org/haploview. Fig. S3).

To increase the coverage of common variants and capture additional association signals in our study, the missing genotypes of autosomal SNPs in our GWAS collection of 99 intestinal BD cases and 597 controls were imputed in the 1000 Genomes Project data released March 2012 (Phase I, version 3) as a reference using IMPUTE (v.2.0, https://mathgen.stats.ox.ac.uk/impute/impute_v2.html,

Supporting information 2). Reference haplotypes included data from 246 African, 181 American, 286 Asian, and 379 European individuals because panel combinations might improve imputation performance [1]. A total of 5,418,765 SNPs with an estimated imputation accuracy of >0.9 and minor allele frequency (MAF) of >0.01 were included in the association analysis.

PCA was performed to assess population structure stratification using the software package EIGENSTRAT v.5.0.1 [2] with default parameters. For initial PCA, Korean samples (199 cases and 597 controls) were analyzed along with unrelated European (CEU) and African (YRI) individuals obtained from the 1000 Genomes Project (phase1_release_v3.20101123). A total of 391,001 markers were used, showing >95% call rates, >5% MAF, and >0.05 HWE *P* values. PCA results in Fig. S1 showed a negligible false-positive association resulting from the population admixture, similar to previous Korean IBD GWAS data [3, 4].

SNP selection and validation study

First, intestinal BD and BD without intestinal involvement were directly compared, but none of the SNPs, of SNPs with $P < 1.0 \times 10^{-4}$, was significant after conservative Bonferroni correction (Supporting information 2). Therefore, rs6838327 in *YIPF7* with the lowest *P* value in a clustered genomic locus on chromosome 4, rs7941240 on chromosome 11, and rs2655653 on chromosome 6 in two intergenic regions with the lowest *P* value were subjected to a validation test. Next, SNPs not

included in the significant regions of BD without intestinal involvement were compared with healthy controls to exclude specific factors related to BD without intestinal involvement. Only 2 SNPs in non-clustered regions met the Bonferroni threshold. We selected a SNP out of these SNPs in a genomic locus (rs4500591, near *MIR548F5* and *NBEA*) and 12 SNPs from 4 genomic loci (*DCAF12, PLCB1*, *ELMO1*, and *NAALADL2*) with more than 4 significant clustered SNPs by chromosome for the validation study. BD groups without intestinal involvement were also compared again to the healthy control group to explore factors that further affect BD without intestinal involvement. Among 342 significant SNPs, there were 6 clustered SNPs, including 2 genomic loci (*LOC284395*, *CD300LB*) after a multiple test correction. We selected one SNP in LOC284395 with the lowest P value (rs7245731, $P = 1.9 \times 10^{-9}$) and the previously reported SNP in CD300LB (rs61730133) as susceptible loci against BD without intestinal involvement, which were also detected in imputation analysis as an intestinal BD-specific gene. To assess more specific intestinal BD-associated SNPs, 60,137 SNPs not included in the significant regions of BD without intestinal involvement were compared between patients with intestinal BD and healthy controls to exclude factors related to BD without intestinal involvement. This analysis identified rs32019 in *CD180*.

Following the genome-wide discovery analysis, a total of 44 SNPs from regions containing more than 4 SNPs with *P* values $<1 \times 10^{-4}$ within 100 kb, SNPs with the lowest *P* values in GWAS, 13 SNPs (rs12119179 and rs1495965 in *IL23R-IL12RB2*; rs1554286, rs1518111, and rs1800871 in *IL10*;

rs5742906 in *CCR1-CCR3*; rs17482078, rs2927615 in *ERAP1*; rs4713242 in *HLA-F-AS1–HLA-A*; rs2255336 in *KLRC4-KLRK1*; rs12525170 and rs4959053 nearby *HLA-B*; rs7574070 in *STAT4*) previously reported in association with BD, additional loci for haplotype analysis, and one locus (rs61730133 in *CD300C/CD300LB*, $P < 2.0 \times 10^{-6}$) from our imputed data were selected for a replication study (Supporting information 3). This study was performed in a non-overlapping set of 196 patients with intestinal BD, 138 BD patients without intestinal involvement, and 391 healthy controls with available DNA.

Validation of rs17856199, rs1800871, and rs4500591 was performed on the same platform by TaqMan genotyping assays (Applied Biosystems, Foster City, CA, USA). Validation of others was performed using the BioMark TM HD system (Fluidigm, South San Francisco, CA, USA) according to the manufacturer's protocol and recommended quality control measures. The concordance rate in 44 SNPs among duplicated samples between Affymetrix Whole-Genome-Wide Human SNP Array 6.0 and TaqMan genotyping assays or the BioMark TM HD system was >99.9%, indicating a negligible possibility of genotype error in this study.

Haplotypic analysis

To clarify the susceptibility regions and to narrow down to the candidate regions, haplotypic associations were analyzed by a method developed by Lake *et al.* [5]. The method, implemented in R

(http://www.r-project.org), estimates the significance of the relative effects of haplotypes on a trait compared to the baseline haplotype. In this study, a haplotype consisting of a combination of the major alleles from each locus was used as a baseline (Table S2 in Supporting information 1).

In silico analyses of SNP functions

For Cis-expression Quantitative Trait Locus (cis-eQTL) analysis, to reveal the genetic loci that control specific changes in gene expression, publicly available sources from eQTL Browser were investigated (http://www.sanger.ac.uk/ resources/software/genevar/, Fig. S8 and S9 in Supporting information 1 and Supporting information 4). The cis associations of each SNP and its proxies with expression of nearby genes were searched in fibroblasts, lymphoblastoid cells, T-cells as well as in adipose and skin tissues [6, 7].

Regulatory SNPs among a total of 44 SNPs were surveyed using RegulomeDB [8] (http://regulomedb.org) and HaploReg v2 resources (http://compbio.mit.edu/HaploReg), which are summarized in Supporting information 4. Variants in RegulomeDB analysis were classified into one of 6 RegulomeDB categories with scores ranging from 1 to 6 indicating putative functions. HaploReg analysis was used to identify biological features in sequences containing BD risk variants and SNPs in high LD ($r^2 = 0.8$ in Europeans from the 1000 Genomes Project) as well as evolutionarily conserved regions based on SIte-specific PHYlogenetic (SiPhy) analysis [9, 10]. Functional elements located in

the same regions as the index and correlated SNPs were identified using ENCODE [11]. These elements were annotated with potential effects on regulatory motifs based on existing databases such as TRANSFAC, JASPAR, and PBMC [10]. In HapoReg analysis, ASN (CHB+JPT) was selected as the population for LD calculation. All SNPs in the ASN population were chosen as the background for enhancer enrichment analysis.

Immunohistochemical staining

For immunohistochemical analysis, formalin-fixed paraffin-embedded (FFPE) tissue sections (21 normal tissues obtained from cancer patients and 29 inflamed tissues from patients with intestinal BD after surgical resection) were deparaffinized, washed with distilled water, and heated in 10 mM citrate buffer (pH 6.0) for 10 min to allow antigen retrieval. Sections were then washed with distilled water and treated with 3% H_2O_2 for 5 min. After washing with Tris-buffered saline containing 0.1% Tween-20 (TBS-T), sections were blocked with 2.5% normal horse serum in TBS-T for 1 h and then incubated with NAALADL2 antibody (1:100, Santa Cruz Biotechnology, Santa Cruz, CA, USA) and YIPF7 (1:100, Novus Biologicals, Littleton, CO, USA) diluted in 1% normal horse serum at 4℃ overnight. After washing, sections were incubated with biotin-conjugated IgG (1:500, Vector Laboratories, Burlingame, CA, USA) and then treated with reagents from a Vecta-Elite streptavidin-peroxidase kit (Vector Laboratories) with benzidine substrate for color development. Sections were counterstained

with diluted hematoxylin and examined by light microscopy (Olympus BX41; Olympus Optical, Tokyo, Japan). To quantify gene expression, randomly selected fields were studied for each sample at 100× magnification and scored from 0 to 3.

Quantitative real-time reverse-transcription polymerase chain reaction (qRT-PCR)

Total RNA extraction and reverse-transcription were performed as previously described [12]. PCR was performed using primers for *NAALADL2*, *YIPF7*, *TNFA*, *IL17*, and *IL10* (AccuTarget qRT-PCR primer, Bioneer). Samples were amplified in a StepOne Plus real-time PCR system (Applied Biosystems) for 40–45 cycles using the following PCR variables: 95℃ for 30 sec, 60–63℃ for 30 sec, and 72℃ for 40 sec. Finally, quantitative analysis was performed using the relative standard curve method. The results were reported as a relative expression or fold change compared to the calibrator after normalization of the transcript level to the endogenous control, glyceraldehyde-3-phosphate dehydrogenase (*GAPDH*) or β-actin.

Trinitrobenzenesulfonic acid (TNBS) and dextran sulfate sodium (DSS)-induced colitis in mice

Gene function in colon inflammation was investigated using a mouse colitis model; although there is no currently established murine colitis models resembling human intestinal BD. Eight-week-old, male C57BL/6 mice (Orient, Seongnam, Korea) were used. The mice were maintained on 12:12 h light:dark

cycles under specific pathogen-free conditions. Mice were slightly anesthetized with an i.p. injection of zoletil (20 mg/kg) and xylazine (10 mg/kg). A 5% (w/v) TNBS solution (100 μL) in 50% ethanol was then administered using a thin round-tip needle equipped with a 1-mL syringe inserted into the colon 4 cm proximal to the anus. Animals were maintained in a head-down vertical position for 2 min to distribute the agents within the entire colon and caecum (day 0). Animals were euthanized at day 3. DSS-induced colitis was accomplished using 3% DSS as previously described [13]. Throughout the experiment, mice were monitored for body weight loss and overall mortality. After euthanasia, colons were quickly removed from proximal rectums, opened longitudinally, and gently cleared of stool by PBS. The colon length was measured between the ileocecal junction and proximal rectum. Colon tissue was then used for qRT-PCR analyses. All animal experiments were performed according to all applicable Korean laws and were reviewed and approved by the Institutional Animal Care and Use Committee of Yonsei University Severance Hospital (Seoul, Korea, IACUC Approval No: 2013-0166).

Statistical analysis

The association of selected SNPs with the disease or a subset of diseases was analyzed by comparing minor allele frequencies between case and control groups. Statistical significance was determined by chi-square test, while the Cochran-Armitage Trend test was performed as a parametric method and the Jonckheere-Terpstra test was performed as a non-parametric method, using SAS 9.1.3 version (SAS

institute Inc. Cary, NC, USA). Logistic regression analysis was also performed to obtain the odds ratio (OR), 95% confidence interval (CI) for OR, and corresponding *P* values between cases and controls regarding the selected SNPs under four alternative models (dominant, recessive, codominant, and allelic). Significant *P* values in association analysis for combined samples were computed using chi-square and the Cochran-Mantel-Haenszel test (Bonferroni correction was applied with a statistical significance level $P < 1.1 \times 10^{-3}$ for 44 SNPs). For minor allele frequency of 0.4, the study achieved 80% power to detect OR as small as 1.56, as estimated by Quanto (version 1.2.4). For each reported SNP, the power for detection at a nominal *P* value of 0.05 was calculated based on the reported OR and allele frequency of the Korean population.

The prognosis of intestinal BD (cumulative probabilities of operation, admission, corticosteroid use, and immunosuppressant use after diagnosis) was analyzed using the Kaplan–Meier method with differences determined by log-rank test. Student's t-test and Kruskal-Wallis test were performed to test for significant differences. $P < 0.05$ was considered significant between samples in experiments.

- **Supporting Results**

Pathway analysis

Overlap between significant regions was examined according to published GWAS results (GWAS catalog: Supporting information 6). One functional network of 3 identified networks from the BD GWAS catalog (14 genes) included 9 focus molecules with potential functions of T helper cell differentiation ($P = 2.83 \times 10^{-8}$), IL12 signaling and production in macrophages ($P = 3.79 \times 10^{-7}$), IL10 signaling ($P = 4.88 \times 10^{-4}$), and dendritic cell maturation ($P = 3.31 \times 10^{-3}$). The top functional network of 25 networks from the IBD GWAS catalog (536 genes) included 26 focus molecules with potential functions, including T helper cell differentiation ($P = 2.97 \times 10^{-29}$), dendritic cell maturation ($P = 8.27 \times 10^{-24}$), and communication between dendritic cells and natural killer cells ($P = 5.7 \times 10^{-22}$). Notably, the pathway analyses showed that intestinal BD genes (13 genes) presented one functional network including 11 focus molecules that overlapped with 8 networks (Networks 5, 9, 10, 13, 15, 16, 22, and 23) of IBD GWAS genes. These results suggest that the phenotype of intestinal BD shares some common pathogenic risk factors with IBD (Fig. 1b and Fig. S6). Intestinal BD related genes have only one network (Network 1) derived from previous BD GWAS, which may also indirectly connect the intestinal BD pathway with the IBD pathway (Networks 2 and 11; Fig. 1c). The functional network shares common potential functions with PI3K signaling in B lymphocytes (PLCB1, CD180) and

CXCR4 (PLCB1, ELMO1) signaling with 2 focus molecules (CD180, ELMO1) from 5 BD without intestinal involvement-associated genes (Fig. S6). This suggests that some intestinal BD genes result in BD phenotypes through interactions with genes associated with BD with and without intestinal involvement. Comparisons between BD genes and IBD genes from the GWAS catalog also demonstrated that 2 BD networks (Networks 1 and 3) overlapped with IBD networks (Fig. S6a).

References

1 Marchini J, Howie B. Genotype imputation for genome-wide association studies. Nat Rev Genet 2010;11(7):499-511.

2 Patterson N, Price AL, Reich D. Population structure and eigenanalysis. PLoS genetics 2006;2(12):e190.

3 Yang SK, Hong M, Zhao W, et al. Genome-wide association study of ulcerative colitis in Koreans suggests extensive overlapping of genetic susceptibility with Caucasians. Inflammatory bowel diseases 2013;19(5):954-66.

4 Yang SK, Hong M, Zhao W, et al. Genome-wide association study of Crohn's disease in Koreans revealed three new susceptibility loci and common attributes of genetic susceptibility across ethnic populations. Gut 2014;63(1):80-7.

5 Lake SL, Lyon H, Tantisira K, et al. Estimation and tests of haplotype-environment interaction when linkage phase is ambiguous. Human heredity 2003;55(1):56-65.

6 Dimas AS, Deutsch S, Stranger BE, et al. Common regulatory variation impacts gene expression in a cell type-dependent manner. Science 2009;325(5945):1246-50.

7 Stranger BE, Montgomery SB, Dimas AS, et al. Patterns of cis regulatory variation in diverse human populations. PLoS Genet 2012;8(4):e1002639.

8 Boyle AP, Hong EL, Hariharan M, et al. Annotation of functional variation in personal

genomes using RegulomeDB. Genome Res 2012;22(9):1790-7.

9 Garber M, Guttman M, Clamp M, Zody MC, Friedman N, Xie X. Identifying novel constrained elements by exploiting biased substitution patterns. Bioinformatics 2009;25(12):i54-62.

10 Ward LD, Kellis M. HaploReg: a resource for exploring chromatin states, conservation, and regulatory motif alterations within sets of genetically linked variants. Nucleic Acids Res 2012;40(Database issue):D930-4.

11 Consortium EP. An integrated encyclopedia of DNA elements in the human genome. Nature 2012;489(7414):57-74.

12 Kim SW, Kim ES, Moon CM, et al. Genetic polymorphisms of IL-23R and IL-17A and novel insights into their associations with inflammatory bowel disease. Gut 2011;60(11):1527-36.

13 Kim SW, Kim HM, Yang KM, et al. Bifidobacterium lactis inhibits NF-kappaB in intestinal epithelial cells and prevents acute colitis and colitis-associated colon cancer in mice. Inflamm Bowel Dis 2010;16(9):1514-25.

Supporting Information 2: BD GWAS results

ⓐ Intestinal BD (iBD, n = 99) vs. BD without intestinal involvement (sBD, n = 100)

rs number	*P* value	Chromosome	Position	Cluster[†]	Selected nearby gene
rs7941240	5.91399E-07	11	43141144	14	Intergenic
rs10838023	5.91399E-07	11	43156912	14	
rs1929756	1.03457E-06	13	76804369		
rs16755	1.70990E-06	18	11118427		
rs2018445	2.30444E-06	11	43160775	14	
rs1472201	4.23158E-06	4	77793375	7	
rs6838327	6.20427E-06	4	44626846	6	YIPF7
rs4969044	7.47806E-06	17	70980366		
rs2655653	1.02848E-05	6	92037600	9	Intergenic
rs2224062	1.02848E-05	6	92020516	9	
rs2655634	1.02848E-05	6	92021630	9	
rs7318897	1.22749E-05	13	90541300	16	
rs1341651	1.22749E-05	13	90557832	16	
rs12610602	1.23176E-05	19	53896633	18	
rs1113098	1.29386E-05	4	44652187	6	
rs2655628	1.40835E-05	6	92026044	9	
rs9560493	1.41217E-05	13	90545655	16	
rs1898864	1.53811E-05	4	44630548	6	
rs644349	1.71060E-05	5	29506366	8	
rs10250491	1.77410E-05	7	67227867		
rs7328136	1.77864E-05	13	98709941		
rs9301634	1.78302E-05	13	90574498	16	
rs4430786	1.78875E-05	17	15060607	17	
rs1877954	1.90559E-05	2	85654299	4	
rs9914552	2.00533E-05	17	15060382	17	
rs12601604	2.15964E-05	17	75527000		
rs11077753	2.17168E-05	17	72556010		
rs1595658	2.45467E-05	4	44622835	6	
rs990356	2.50908E-05	4	44624156	6	
rs7754081	2.53329E-05	6	92054292	9	
rs16880053	2.53329E-05	6	92066037	9	
rs10485328	2.53329E-05	6	92067726	9	
rs2207613	2.55599E-05	6	92020610	9	
rs2811662	2.55599E-05	6	92029854	9	
rs12031585	2.56622E-05	1	119657938	1	
rs1341583	2.56622E-05	1	119677250	1	
rs1700760	2.66155E-05	5	29506587	8	Intergenic
rs7838050	2.82960E-05	8	118518101		
rs2617650	2.90720E-05	19	53893835	18	
rs8111882	2.90720E-05	19	53894068	18	
rs2102927	2.95511E-05	8	61066300	12	
rs2271153	3.10015E-05	2	235006892		
rs12758590	3.13682E-05	1	246800022	2	
rs12741150	3.13682E-05	1	246800329	2	
rs6659356	3.13682E-05	1	246802868	2	
rs11899060	3.17947E-05	2	226775988	5	
rs11139326	3.28196E-05	9	84194607		
rs10505559	3.61339E-05	8	131721961		
rs9519096	3.79743E-05	13	86927664		
rs7125196	3.94126E-05	11	61272565	15	
rs10204222	3.95850E-05	2	36020504	3	
rs12712466	3.95850E-05	2	36020591	3	
rs11230723	3.96762E-05	11	61272309	15	
rs10232967	4.15692E-05	7	57749918		
rs2720896	4.18522E-05	3	194764403		
rs916352	4.21670E-05	X	86757780		

rs4254878	4.24088E-05	5	35226579		
rs6499988	4.31279E-05	16	58902521		
rs893509	4.33233E-05	10	133013442		
rs16962841	4.40323E-05	19	29939023		
rs2278021	4.43926E-05	16	81076202		
rs965013	4.83694E-05	4	113310981		
rs1520269	5.17186E-05	8	61090641	12	
rs4129249	5.17626E-05	1	246790756	2	
rs12971579	5.25055E-05	19	57512386		
rs6951365	5.26285E-05	7	85012428	11	
rs9954094	5.29767E-05	18	7427516		
rs13022222	5.72742E-05	2	226747677	5	
rs7772106	5.81102E-05	6	92054323	9	
rs4853631	5.81664E-05	2	192581534		
rs6532678	5.86625E-05	4	77794236	7	
rs7557606	6.00344E-05	2	36014002	3	
rs10264013	6.09965E-05	7	10852392	10	
rs1979925	6.13473E-05	5	174413991		
rs16972584	6.21375E-05	15	42355706		
rs118511	6.41316E-05	21	15580754	19	
rs371195	6.41316E-05	21	15583629	19	
rs11974904	6.58048E-05	7	10771349	10	
rs17088909	6.98731E-05	18	71844236		
rs10830909	7.03660E-05	11	88175768		
rs1606076	7.12101E-05	8	60685788		
rs4650353	7.15789E-05	1	81329765		
rs7047812	7.26343E-05	9	9726090		
rs4899050	7.34824E-05	14	61943886		
rs17012552	7.81533E-05	4	88126218		
rs891401	7.83533E-05	8	27381194		
rs17601544	8.01306E-05	4	44843173	6	
rs4720910	8.28409E-05	7	10855494	10	
rs1557664	8.37355E-05	7	103883264		
rs7004663	8.42395E-05	8	74530392	13	
rs12678504	8.42395E-05	8	74639179	13	
rs3213898	8.46651E-05	4	44624177	6	
rs6013079	8.56830E-05	20	49688726		
rs1403786	8.68131E-05	7	85014355	11	
rs7670601	8.71982E-05	4	44715341	6	
rs833456	9.01102E-05	9	10547886		
rs11839457	9.10564E-05	13	56345359		Intergenic
rs11730600	9.15386E-05	4	11623160		
rs10240570	9.16037E-05	7	71954779		
rs1935187	9.16197E-05	13	90530237	16	
rs16938713	9.20829E-05	8	74581464	13	
rs2479872	9.55618E-05	1	92353706		
rs1135604	9.64768E-05	2	85615012	4	
rs7524430	9.69359E-05	1	226693542		
rs7069727	9.82411E-05	10	83798469		
rs6625561	9.94290E-05	X	69225199		
rs7156337	9.94675E-05	14	35160860		
rs4631456	9.94979E-05	8	61104747	12	

‡: Regions containing more than 2 SNPs with *P* value <0.0001 within 100 kb

Blue shows validated SNPs in this study.

Chromosome locations are based on NCBI 37 Build.

ⓑ Intestinal BD (iBD, n = 99) vs. healthy control (healthy CTL, n = 597) within SNPs with *P* value>0.0001 from comparison between BD without intestinal involvement (sBD, n = 100) and healthy control (n = 597)

rs number	*P* value	Chromosome	Position	Cluster[†]	Selected nearby gene
rs6687487	6.36384E-06	1	23061551	1	EPHB2
rs3885883	2.04908E-05	1	59266115		
rs6691740	2.08197E-05	1	66852802		
rs12065732	2.14933E-05	1	175566717		
rs10917438	3.73530E-05	1	19711749		
rs339569	3.99569E-05	1	194940699		
rs284148	4.58611E-05	1	92277843	2	TGFBR3
rs6670890	4.77194E-05	1	116375318	3	NHLH2
rs4532860	4.79068E-05	1	18349821		
rs7556581	7.11401E-05	1	116386105	3	NHLH2
rs503633	7.57919E-05	1	231389097		
rs901913	7.99133E-05	1	92293162	2	TGFBR3
rs2489186	8.03685E-05	1	92297055	2	TGFBR3
rs10753541	8.34596E-05	1	23061992	1	EPHB2
rs1337740	8.53138E-05	1	60544418		
rs4916197	8.53138E-05	1	172831286		
rs17532799	1.20249E-06	2	15773339	5	
rs8179776	3.71590E-06	2	7564293	4	LOC100506274
rs6731427	6.31279E-06	2	68884537	7	PROKR1
rs12619049	9.67097E-06	2	29446701		
rs2287637	1.35043E-05	2	15783051	5	
rs6723640	1.51171E-05	2	144169816		
rs12463669	1.82657E-05	2	15790091	5	
rs6546423	1.96849E-05	2	68883233	7	PROKR1
rs4280455	2.04908E-05	2	216103283		
rs2111724	2.08339E-05	2	57091538	6	
rs17412908	2.11538E-05	2	146794223		
rs17024202	2.41193E-05	2	39934498		
rs12615741	2.41628E-05	2	111935939	8	ACOXL,BCL2L11
rs6728947	3.02868E-05	2	169059511		
rs16865576	3.21503E-05	2	178535465		
rs17032991	3.26751E-05	2	67303965		
rs4674839	3.64240E-05	2	224879207		
rs12471221	3.69653E-05	2	163401685		
rs1463143	4.28534E-05	2	33903260		
rs2289321	5.15922E-05	2	111870220	8	ACOXL,BCL2L11
rs6742537	5.22945E-05	2	83337797		
rs6741957	5.34620E-05	2	57077745	6	
rs3789088	7.02699E-05	2	111790804	8	ACOXL,BCL2L11
rs12617726	7.05472E-05	2	111969151	8	ACOXL,BCL2L11
rs6732885	7.79386E-05	2	7564768	4	LOC100506274
rs6545601	7.91221E-05	2	57077993	6	
rs4341884	8.49533E-05	2	235918504		
rs2080674	8.96430E-05	2	57088328	6	
rs10199928	8.96667E-05	2	141491320		
rs6750111	9.02791E-05	2	228857940	9	SPHKAP
rs11883583	9.02791E-05	2	228860909	9	SPHKAP
rs3732071	9.11667E-05	2	36970016		
rs2048819	9.25523E-05	2	118399142		
rs11680053	9.37140E-05	2	228864662	9	SPHKAP
rs2439568	9.66538E-05	2	135209142		
rs7612622	2.83022E-06	3	137979007		
rs10511129	1.43913E-05	3	78434526	11	
rs16830581*	1.50702E-05	3	159362915	12	IQCJ-SCHIP1
rs9879792	1.79209E-05	3	159359429	12	IQCJ-SCHIP1
rs6772245	1.99090E-05	3	159372107	12	IQCJ-SCHIP1
rs9853191	2.08197E-05	3	9170534		

rs11926288	2.41193E-05	3	316405	10	CHL1
rs11928134	2.41193E-05	3	353520	10	CHL1
rs16830589*	2.51889E-05	3	159365432	12	IQCJ-SCHIP1
rs9290046	2.65308E-05	3	159364557	12	IQCJ-SCHIP1
rs16848171	4.03846E-05	3	175181067	14	NAALADL2
rs3914501	5.71420E-05	3	174564668	13	NAALADL2
rs16825731	6.65590E-05	3	175180991	14	NAALADL2
rs13091513	7.15375E-05	3	930567		
rs3849516	8.24078E-05	3	174558124	13	NAALADL2
rs7611902	8.36520E-05	3	81921467		
rs1401072	9.02791E-05	3	116281707		
rs17016015	9.07079E-05	3	78427899	11	
rs10513733	9.71840E-05	3	175242041	14	NAALADL2
rs1437520	9.86225E-05	3	159347475	12	IQCJ-SCHIP1
rs7674779	3.63594E-06	4	37257069		
rs1489439	8.18229E-06	4	131614612	17	
rs4536897	1.11039E-05	4	87877156	16	AFF1
rs340652	2.42433E-05	4	87935730	16	AFF1
rs4330315	2.49524E-05	4	159697699		
rs4693155	2.84294E-05	4	87920020	16	AFF1
rs3775696	3.17583E-05	4	143228914		
rs6849842	4.35308E-05	4	185436255		
rs6826722	5.03997E-05	4	24706382	15	
rs17005650	5.08661E-05	4	123239788		
rs7674705	5.39861E-05	4	131611683	17	
rs4308347	6.05977E-05	4	174933413		
rs1489442	6.55081E-05	4	131614929	17	
rs17624591	6.72354E-05	4	20787077		
rs10004694	6.76561E-05	4	24723788	15	
rs10026819	7.25964E-05	4	56121821		
rs6531944	7.80352E-05	4	87921241	16	AFF1
rs9990937	8.08300E-05	4	157483467		
rs6831193	8.27957E-05	4	100908585		
rs7698068	8.81080E-05	4	63907850		
rs12500507	9.70633E-05	4	24705742	15	
rs11733360	9.71840E-05	4	7427831		
rs185566	2.60581E-06	5	114646029		
rs264986	3.76976E-06	5	79170424		
rs3935489	2.52047E-05	5	32293735		
rs7727198	3.98001E-05	5	88889955		
rs6876218	6.24603E-05	5	82193633		
rs620508	6.87085E-05	5	29507295		
rs9348841	2.25983E-07	6	30577873		
rs1086494	4.60330E-06	6	154302366	20	OPRM1
rs6918658	1.50260E-05	6	5717397	18	FARS2
rs4715005	2.25497E-05	6	47364957		
rs9378150	3.33332E-05	6	30869693		
rs9348863	3.99683E-05	6	31262461		
rs16892285	4.23260E-05	6	81536002		
rs7741082	4.85390E-05	6	6778912	19	
rs4960242	5.60917E-05	6	6779888	19	
rs4960245	5.60917E-05	6	6780524	19	
rs1938167	5.60917E-05	6	6783594	19	
rs4464841	5.60917E-05	6	6783708	19	
rs11757961	5.74443E-05	6	5708735	18	FARS2
rs1938166	6.37770E-05	6	6783617	19	
rs282091	6.88083E-05	6	154261427	20	OPRM1
rs2479003	6.88279E-05	6	2318449		
rs3828913	7.00185E-05	6	31465795		
rs11754928	7.60678E-05	6	111316619		
rs12197082	9.11667E-05	6	3809510		

rs10259514*	7.86476E-06	7	36829705	23	ELMO1
rs10271212	1.72092E-05	7	36814860	23	ELMO1
rs4722071	1.95506E-05	7	21995315	22	
rs801089	2.41193E-05	7	140651523	25	TMEM178B,MRPS33
rs10239770	2.41193E-05	7	140721368	25	TMEM178B,MRPS33
rs512509	2.41193E-05	7	140758147	25	TMEM178B,MRPS33
rs1174940	2.45323E-05	7	22001693	22	
rs488795	2.58145E-05	7	140757886	25	TMEM178B,MRPS33
rs2017812	2.65443E-05	7	12336093	21	VWDE
rs11980906	3.02819E-05	7	12333929	21	VWDE
rs10950385	3.52434E-05	7	12155047		
rs10238019	3.81181E-05	7	36858203	23	ELMO1
rs12672764	4.00066E-05	7	140698551	25	TMEM178B,MRPS33
rs38808	5.01263E-05	7	92145950	24	PEX1
rs4727276	5.99560E-05	7	92107752	24	PEX1
rs4723598	6.96848E-05	7	36953196	23	ELMO1
rs17171876	8.22480E-05	7	41515005		
rs2074714	8.28741E-05	7	147259438		
rs491554	8.81080E-05	7	140776614	25	TMEM178B,MRPS33
rs534348	8.81080E-05	7	140782235	25	TMEM178B,MRPS33
rs4440598	1.93644E-06	8	95068543		
rs1346897	2.83022E-06	8	102299833		
rs880642	7.71818E-06	8	96221214		
rs6558989	9.17274E-06	8	5118515	26	
rs4128412	1.11986E-05	8	5110469	26	
rs10089783	1.26189E-05	8	5085053	26	
rs189317	2.26189E-05	8	2733176		
rs4872196	2.41193E-05	8	23692067		
rs10097563	6.01889E-05	8	40580638		
rs7816936	9.07461E-05	8	5071412	26	
rs12003392	8.56776E-07	9	9151640	27	PTPRD
rs10758242	4.00239E-06	9	34146776	30	DCAF12
rs501873	6.01623E-06	9	34816235	31	FAM205B
rs10441723	7.28828E-06	9	34082144	30	DCAF12
rs10758240	7.28828E-06	9	34098110	30	DCAF12
rs10511914	7.28828E-06	9	34107232	30	DCAF12
rs1220673	8.30685E-06	9	132342281	32	
rs11243818	2.04908E-05	9	135406494		
rs7858279	2.21138E-05	9	30531968		
rs7043302	2.41193E-05	9	9139951	27	PTPRD
rs1335049	2.86982E-05	9	98447883		
rs10971709	3.05259E-05	9	33804813	29	PRSS3,UBE2R2
rs10971711	3.50093E-05	9	33815842	29	PRSS3,UBE2R2
rs10810544	5.46280E-05	9	16286870	28	
rs7861593	6.02090E-05	9	16290697	28	
rs7875776	6.60639E-05	9	14552979		
rs10733474	8.67406E-05	9	34795097	31	FAM205B
rs2178824	9.40594E-05	9	33765513	29	PRSS3,UBE2R2
rs1231463	9.47356E-05	9	132339129	32	
rs7910988	2.07743E-05	10	134966362		
rs7910780	2.14933E-05	10	129346305	34	NPS
rs11018189	2.18383E-05	10	129345788	34	NPS
rs10886852	2.37864E-05	10	122868596	33	
rs11199770	2.94572E-05	10	122868170	33	
rs12220562	5.80667E-05	10	32029437		
rs2796789	8.98526E-05	10	36626633		
rs2785155	1.37654E-09	11	35068606		
rs4753390	9.31828E-07	11	91846180	36	
rs2078216	8.96319E-06	11	122204076		
rs2042683	1.08144E-05	11	25264523	35	
rs11033293	1.20197E-05	11	35856943		

rs674503	1.45629E-05	11	75268337		
rs16921968	1.66251E-05	11	95194085		
rs16934303	2.08197E-05	11	40085949		
rs4515927	2.41193E-05	11	91866812	36	
rs16916983	2.45323E-05	11	91834020	36	
rs3996554	2.45323E-05	11	91868956	36	
rs2398136	2.67064E-05	11	91872001	36	
rs7927031	3.02868E-05	11	25247977	35	
rs4584535	3.02868E-05	11	25255960	35	
rs7129491	3.02868E-05	11	57095082		
rs7131623	4.98377E-05	11	113500513		
rs1791785	7.92759E-05	11	61442813		
rs661993	8.25816E-05	11	35558748		
rs11038039	8.53138E-05	11	44530645		
rs12291500	8.99510E-05	11	126414087		
rs11068952	1.40911E-07	12	118887856		
rs2970839	8.11056E-07	12	19254829		
rs1398376	6.92877E-06	12	28752435	38	
rs2103204	9.07990E-06	12	119391981		
rs17019943	1.54498E-05	12	92427190		
rs7967959	2.08197E-05	12	2500314		
rs11831715	2.36036E-05	12	24433480	37	SOX5
rs16933100	2.64412E-05	12	28765347	38	
rs16933103	3.29000E-05	12	28766116	38	
rs1512571	3.40307E-05	12	28756488	38	
rs7315674	3.61683E-05	12	12146359		
rs526058	5.52315E-05	12	24435421	37	SOX5
rs2257156	6.60639E-05	12	105591083		
rs1882033	7.55483E-05	12	67819376	40	
rs2280080	8.53138E-05	12	54801204	39	ITGA5
rs7302345	8.53138E-05	12	54802872	39	ITGA5
rs2551390	8.53138E-05	12	114857358	42	TBX5, TBX5-AS1
rs2551378	8.53138E-05	12	114874902	42	TBX5, TBX5-AS1
rs2859670	8.53138E-05	12	114879632	42	TBX5, TBX5-AS1
rs12422543	8.56129E-05	12	4464430		
rs1248063	8.67406E-05	12	114862921	42	TBX5, TBX5-AS1
rs11043982	8.67406E-05	12	18409542		
rs2161792	8.81915E-05	12	114875198	42	TBX5, TBX5-AS1
rs1798011	9.07079E-05	12	88956625	41	KITLG
rs1703081	9.26699E-05	12	88951387	41	KITLG
rs2870937	9.75100E-05	12	67819682	40	
rs4500591	8.88178E-16	13	36081260		MIR548F5, NBEA
rs9519096	1.26109E-06	13	86927664		
rs2997108	1.95646E-05	13	56383613	43	intergenic between RPL13AP25 and HNF4GP1
rs9559174	2.11538E-05	13	108503398		
rs11842196	2.16910E-05	13	76493793	44	
rs1323569	2.41193E-05	13	76467127	44	
rs9600593	2.41193E-05	13	76486342	44	
rs7989987	3.45659E-05	13	46865392		
rs17267214	3.46881E-05	13	93243648		
rs9315032	3.86676E-05	13	31280212		
rs1513041	4.45472E-05	13	104806056		
rs9599714	4.67567E-05	13	71277340		
rs9597267	5.02431E-05	13	56370860	43	intergenic between RPL13AP25 and HNF4GP1
rs3015418	5.25858E-05	13	56392267	43	intergenic between RPL13AP25 and HNF4GP1
rs11839457	8.83910E-05	13	56345359	43	intergenic between RPL13AP25 and HNF4GP1
rs17097879	4.55049E-06	14	31895974		
rs17091965	3.22012E-05	14	57157830	45	
rs12433533	4.34098E-05	14	61960906	46	PRKCH
rs12437374	4.55121E-05	14	61961173	46	PRKCH
rs12147287	4.74216E-05	14	101554839		

rs17098514	7.90696E-05	14	61952839	46	PRKCH
rs8020299	9.11049E-05	14	52645780		
rs17091976	9.12209E-05	14	57170111	45	
rs4923804	4.86204E-06	15	39019716	47	
rs16967283	9.67097E-06	15	39015636	47	
rs473249	1.99562E-05	15	53493208	48	
rs538505	2.06032E-05	15	53487760	48	
rs338364	2.19451E-05	15	68173921		
rs4778334	2.74697E-05	15	23040856		
rs1564328	2.78079E-05	15	33282844		
rs3021420	3.59218E-05	15	101917664		
rs8029187	4.57005E-05	15	44338950		
rs690013	5.37286E-05	15	53492361	48	
rs8042219	8.00274E-05	15	42352153		
rs2970364	8.04096E-05	15	60140209		
rs11633486	9.16319E-05	15	24088722		
rs8044765	1.87856E-06	16	7958906		
rs247825	4.86231E-06	16	84583727		
rs2189290	4.84167E-05	16	24356533		
rs2967192	4.97312E-05	16	64290094	49	
rs3760083	5.35712E-05	16	3450052		
rs2926134	8.66237E-05	16	64281926	49	
rs1013996	1.35043E-05	17	68063631	50	KCNJ16
rs2108440	1.37615E-05	17	68077926	50	KCNJ16
rs9910533	1.37615E-05	17	68049240	50	KCNJ16
rs2574861	2.36330E-05	17	75507296	51	
rs449988	2.36330E-05	17	75507679	51	
rs2996026	4.00902E-05	17	13345571		
rs2108439	8.53138E-05	17	68077738	50	KCNJ16
rs4141238	8.67406E-05	17	68067881	50	KCNJ16
rs3930821	9.31925E-05	17	20697797		
rs10747215	1.11986E-05	18	75230448	52	
rs12607506	1.37615E-05	18	56865533		
rs11661532	3.19704E-05	18	75234255	52	
rs2277719	3.69653E-05	18	56199529		
rs1489169	5.50195E-05	18	45875660		
rs1652366	8.67406E-05	18	21192261		
rs11880125	1.01923E-05	19	32083223	53	
rs17616661	2.10693E-05	19	11303554		
rs7252301	2.38219E-05	19	32107103	53	
rs7250299	4.59282E-05	19	32103145	53	
rs6086633	6.81593E-06	20	8823064	54	PLCB1
rs6086632	7.80707E-06	20	8822931	54	PLCB1
rs6140755	7.81319E-06	20	8824117	54	PLCB1
rs12624809	8.54910E-06	20	8822431	54	PLCB1
rs6140740	8.54910E-06	20	8818700	54	PLCB1
rs6140742	8.54910E-06	20	8819112	54	PLCB1
rs2179475	8.54910E-06	20	8820280	54	PLCB1
rs6039302	1.14285E-05	20	8831137	54	PLCB1
rs6140753	1.30841E-05	20	8822723	54	PLCB1
rs3848829	1.83137E-05	20	8848724	54	PLCB1
rs3848831	2.29837E-05	20	8851321	54	PLCB1
rs2761272	2.49524E-05	20	10749074		
rs6086653	3.48964E-05	20	8838343	54	PLCB1
rs6140779	3.48964E-05	20	8843845	54	PLCB1
rs6059286	3.51998E-05	20	31957992		
rs6086654	4.23561E-05	20	8838376	54	PLCB1
rs2425046	8.53138E-05	20	33871661		
rs1571717	3.41885E-05	21	41698322		
rs8130926	8.97104E-05	21	33333331		
rs713923	4.17787E-06	22	48891519	56	FAM19A5

rs6007870	7.50029E-06	22	48894318	56	FAM19A5
rs4645805	1.92413E-05	22	35815063		
rs17480137	2.04908E-05	22	28279688	55	PITPNB,TTC28-AS1,TTC28,MIR548AM
rs7286237	2.04908E-05	22	28371305	55	PITPNB,TTC28-AS1,TTC28,MIR548AM
rs135113	2.04908E-05	22	28488450		
rs5767172	8.04430E-05	22	48892314	56	FAM19A5
rs1575119	7.97455E-06	X	114350460	57	LRCH2
rs2396925	7.97455E-06	X	114503236	58	
rs5988209	1.06421E-05	X	114291450	57	LRCH2
rs7886303	1.37887E-05	X	136870739	59	
rs6528478	1.37887E-05	X	136880403	59	
rs6635478	1.37887E-05	X	136882025	59	
rs1342044	1.37887E-05	X	136909946	59	
rs17331107	1.72635E-05	X	136854221	59	
rs11796573	1.72635E-05	X	136858447	59	
rs2859251	1.97124E-05	X	136598435		
rs6627785	9.57966E-05	X	152616701		
rs2064652	9.99659E-05	X	114502990	58	

‡: Regions containing more than 2 SNPs with *P* value <0.0001 within 100 kb

*Reported SNPs

Yellow shows significant *P* value < 8.5×10^{-8} (Bonferroni corrected).

Blue shows validated SNPs in this study.

Chromosome locations are based on NCBI 37 Build.

ⓒ **BD without intestinal involvement (sBD, n = 100) vs. healthy control (healthy CTL, n = 597)**

rs number	*P* value	Chromosome	Position	Cluster[†]	Selected nearby gene
rs7245731	1.89459E-09	19	29975118	61	LOC284395
rs12515056	3.52677E-09	5	25594997		
rs10852753	2.55548E-08	17	72536009	56	CD300LB,CD300C
rs2242462	4.24379E-08	17	72517824	56	CD300LB,CD300C
rs11654726	5.63191E-08	17	72534875	56	CD300LB,CD300C
rs4788844	5.85493E-08	17	72534133	56	CD300LB,CD300C
rs10497642	1.49561E-07	2	184828432		
rs4788843	1.64325E-07	17	72528091	56	CD300LB,CD300C
rs8060711	2.06154E-07	16	83678513	54	CDH13
rs10512596	2.07300E-07	17	72523224	56	CD300LB,CD300C
rs8060190	4.16920E-07	16	83678303	54	CDH13
rs2116878	4.45829E-07	19	29968357	61	LOC284395
rs10860907	4.53673E-07	12	103130062	48	
rs2866068	4.57275E-07	19	29946763	61	LOC284395
rs7296994	4.95619E-07	12	103128049	48	
rs4796796	7.61571E-07	17	76614120	58	
rs17722916	7.67893E-07	6	136732406		
rs2073326	8.55748E-07	22	29105527		
rs28359178	9.13588E-07	26	13708		
rs2960877	9.13588E-07	7	70862756		
rs13020	9.92123E-07	12	56120523		
rs1532800	1.09075E-06	17	72537179	56	CD300LB,CD300C
rs12300112	1.27257E-06	12	103147575	48	
rs12089039	1.31279E-06	1	18263510	1	
rs242195	1.31279E-06	3	60399154		
rs2469195	1.41220E-06	15	77259486		
rs1918638	1.50366E-06	15	47150142	51	MIR548A3
rs9950450	1.75742E-06	18	5471765	59	EPB41L3
rs9952940	1.81009E-06	18	5472021	59	EPB41L3
rs1726423	1.81815E-06	12	78621072	47	
rs1899940	1.93095E-06	5	38561303	30	LIFR,LIFR-AS1
rs6804425	1.93383E-06	3	62000476		
rs7244835	2.45451E-06	18	5461414	59	EPB41L3
rs13176854	2.80065E-06	5	176010156		
rs2114591	2.82456E-06	2	231050569	14	SP110
rs10816460	2.94256E-06	9	109780666	43	ZNF462
rs10497618	3.02998E-06	2	183870146		
rs1699567	3.13859E-06	17	72546965	56	CD300LB,CD300C
rs4542839	3.19583E-06	2	231046602	14	SP110
rs3097247	3.38109E-06	5	38491937	30	LIFR,LIFR-AS1
rs1963340	3.99318E-06	18	5452292	59	EPB41L3
rs6740565	4.10136E-06	2	67515512		
rs11703031	4.29974E-06	22	35036264		
rs17232796	4.49051E-06	5	14149763	26	TRIO
rs17178527	4.51725E-06	6	141906080		
rs4649436	4.67911E-06	1	232453777	9	
rs9380217	4.83644E-06	6	31051553	36	PSORS1C1 (HLA-B)
rs10089590	5.01099E-06	8	40821132		
rs11014632	5.68456E-06	10	25877651		
rs17029872	6.11181E-06	3	30770287		
rs11787443	6.68747E-06	8	9641811		
rs2584732	6.70584E-06	18	11025325		
rs13151212	6.82820E-06	4	97685140	21	
rs11944591	6.82820E-06	4	97694898	21	
rs1859537	6.97190E-06	7	147880163	40	MIR548T,CNTNAP2
rs10485544	6.97190E-06	20	16019252		
rs7004093	7.13407E-06	8	134462291	41	
rs6133736	7.32906E-06	20	9653962	62	PAK7
rs10513118	7.90010E-06	5	12962546		
rs340835	7.90791E-06	1	214163675	8	PROX1
rs10920357	8.37653E-06	1	202151566	7	PTPRVP

rs12442708	8.95043E-06	15	47144386	51	MIR548A3
rs10070910	9.28300E-06	5	116013795		
rs10494629	9.53004E-06	1	189997930		
rs10750219	9.86209E-06	11	122558850	45	UBASH3B
rs7129189	9.86209E-06	11	122562045	45	UBASH3B
rs911547	1.01045E-05	10	105639421		
rs1886904	1.04080E-05	1	157835595	6	
rs193714	1.04318E-05	5	179315794	34	TBC1D9B
rs4430320	1.07206E-05	1	157845221	6	
rs13162439	1.07804E-05	5	25812208	28	
rs933310	1.08318E-05	17	13270658	55	
rs151558	1.13625E-05	20	3464300		
rs645297	1.24748E-05	6	10469826		
rs7724830	1.25029E-05	5	14160447	26	TRIO
rs12597308	1.27050E-05	16	16969951		
rs17298767	1.27955E-05	5	14157376	26	TRIO
rs17055833	1.28116E-05	9	73412162	42	TRPM3
rs17055851	1.28116E-05	9	73416273	42	TRPM3
rs7695171	1.33529E-05	4	140700785	23	MAML3
rs2293708	1.34917E-05	14	22331151		
rs10816459	1.37938E-05	9	109778256	43	ZNF462
rs10140657	1.38186E-05	14	52892612	49	
rs1217744	1.39139E-05	5	71245810		
rs7711157	1.41103E-05	5	74239494	31	
rs4450009	1.46360E-05	1	157879145	6	
rs132700	1.51231E-05	22	36587704	64	APOL4
rs13062734	1.51276E-05	3	105513689		
rs7851469	1.51276E-05	9	118540789		
rs340882	1.52662E-05	1	214145731	8	PROX1
rs2381553	1.54129E-05	2	144445980	12	ARHGAP15
rs1941101	1.54129E-05	18	13987707	60	ZNF519
rs12003225	1.54129E-05	9	8182214		
rs13431051	1.54129E-05	2	155206097		
rs11725136	1.57036E-05	4	11933565		
rs12597271	1.57950E-05	16	87776213		
rs17312167	1.59366E-05	4	156344492	25	
rs524646	1.60783E-05	1	191401978		
rs2042960	1.64173E-05	5	31819330	29	
rs6885954	1.64173E-05	5	31824213	29	
rs10458627	1.67693E-05	1	157848231	6	
rs4077596	1.67693E-05	17	76618696	58	
rs3756419	1.68943E-05	5	38491403	30	LIFR,LIFR-AS1
rs7416590	1.72600E-05	1	157848031	6	
rs1930069	1.72807E-05	9	77830922		
rs7528295	1.76308E-05	1	202140228	7	PTPRVP
rs6808240	1.77167E-05	3	2422321	16	CNTN4
rs1027219	1.78988E-05	2	7611683		
rs7309163	1.83904E-05	12	117729274		
rs1689271	1.97416E-05	1	76392862	2	ASB17
rs1796812	1.97416E-05	1	76401831	2	ASB17
rs7201416	2.02417E-05	16	83740068	54	CDH13
rs12564953	2.03046E-05	1	119665129	4	WARS2
rs12567198	2.03279E-05	1	119610764	4	WARS2
rs2423446	2.06599E-05	20	9711219	62	PAK7
rs1427310	2.07035E-05	2	144912521	13	GTDC1
rs6857264	2.08071E-05	4	54953324	20	
rs7084288	2.14933E-05	10	128059654		
rs4959053*	2.15868E-05	6	31099577	36	HLA-B
rs2233375	2.20811E-05	2	233896498		
rs3930962	2.24737E-05	18	13999049	60	ZNF519
rs6733552	2.25405E-05	2	238578536	15	LRRFIP1
rs6723512	2.25405E-05	2	238578549	15	LRRFIP1
rs4627424	2.25405E-05	4	54949995	20	

rs9403454	2.25405E-05	6	143484200	37	AIG1
rs17072288	2.25405E-05	6	143508908	37	AIG1
rs41372750	2.25405E-05	17	13271274	55	
rs930297	2.25405E-05	17	73404537	57	GRB2
rs4789193	2.25405E-05	17	73404796	57	GRB2
rs7223674	2.25405E-05	17	73413449	57	GRB2
rs871295	2.25405E-05	16	5216718		
rs134049	2.25405E-05	22	28058812		
rs2833679	2.25405E-05	21	33515924		
rs6024258	2.25405E-05	20	36872818		
rs545982	2.25405E-05	18	66257569		
rs17706970	2.25405E-05	14	84765126		
rs7951538	2.25405E-05	11	95867689		
rs7462840	2.25405E-05	8	140135202		
rs6787085	2.28987E-05	3	2436234	16	CNTN4
rs6762390	2.28987E-05	3	191404361	19	
rs132688	2.28987E-05	22	36576940	64	APOL4
rs7839487	2.28987E-05	8	28465696		
rs11577394	2.28987E-05	1	79106967		
rs7205784	2.30445E-05	16	83740167	54	CDH13
rs2865625	2.30445E-05	16	12850671		
rs7601625	2.31461E-05	2	55251374	11	RTN4
rs10797472	2.31599E-05	1	232464505	9	
rs11125770	2.32627E-05	2	59328816		
rs1727090	2.32627E-05	12	102031538		
rs7341250	2.33084E-05	6	13001274		
rs7633696	2.36324E-05	3	191395972	19	
rs4789189	2.36324E-05	17	73397985	57	GRB2
rs10967075	2.36324E-05	9	25788198		
rs6882589	2.36324E-05	5	152778847		
rs6705487	2.37439E-05	2	113431092		
rs10220700	2.37522E-05	14	101835482		
rs12122986	2.40081E-05	1	90568148		
rs340258	2.40081E-05	3	158634099		
rs2498500	2.40250E-05	1	119673093	4	WARS2
rs1298	2.43016E-05	5	179289895	34	TBC1D9B
rs2423445	2.43807E-05	20	9711104	62	PAK7
rs1251552	2.44399E-05	1	76444108	2	ASB17
rs930296	2.47776E-05	17	73404168	57	GRB2
rs35346	2.47776E-05	12	115611773		
rs10890817	2.51715E-05	11	108006047		
rs2358471	2.55718E-05	4	149597093	24	
rs17377880	2.58591E-05	4	156375974	25	
rs12036768	2.65934E-05	1	157846165	6	
rs17158135	2.67064E-05	10	2360250	44	LINC00701
rs7674926	2.67073E-05	4	149619238	24	
rs931798	2.67073E-05	5	155810823	33	SGCD
rs6880816	2.67073E-05	5	155864868	33	SGCD
rs12652669	2.67073E-05	5	155876204	33	SGCD
rs7751661	2.67073E-05	6	21621850	35	
rs1744864	2.67073E-05	6	21630684	35	
rs35162519	2.67073E-05	5	13181231		
rs2039142	2.67073E-05	9	36963222		
rs17681451	2.67073E-05	3	114399296		
rs1341583	2.71146E-05	1	119677250	4	WARS2
rs7203252	2.71244E-05	16	83685999	54	CDH13
rs4704801	2.71602E-05	5	155824027	33	SGCD
rs17762396	2.76209E-05	9	103902955		
rs1889752	2.76294E-05	1	80511059		
rs2496009	2.79722E-05	10	106604706		
rs16908987	2.80822E-05	8	139384701		
rs11813699	2.80894E-05	10	98810892		
rs1251272	2.83905E-05	1	76289004		

rs2753591	2.85658E-05	14	26462208		
rs17836763	2.85658E-05	14	67908233		
rs13189082	2.90500E-05	5	31329533		
rs154894	2.90504E-05	18	58874142		
rs2827617	2.95433E-05	21	23954629		
rs9511501	2.96581E-05	13	25460242		
rs1251539	2.98976E-05	1	76436260	2	ASB17
rs11077753	3.03219E-05	17	72556010	56	CD300LB,CD300C
rs4702133	3.08669E-05	5	16261195	27	
rs9293634	3.11339E-05	5	74239231	31	
rs12055568	3.12557E-05	6	32192083		
rs3814165	3.13703E-05	10	15256194		
rs2414717	3.22503E-05	15	61861985	52	
rs11114053	3.33363E-05	12	109168515		
rs12042797	3.37172E-05	1	119680090	4	WARS2
rs17025903	3.39605E-05	4	97742156	21	
rs11743167	3.39605E-05	5	16232215	27	
rs12519388	3.39605E-05	5	107195737	32	FBXL17
rs2505449	3.39605E-05	10	26019605		
rs551356	3.39605E-05	2	30984850		
rs493243	3.39605E-05	1	37489221		
rs7560377	3.39605E-05	2	49739575		
rs2839081	3.41725E-05	21	47441315	63	
rs1794225	3.41725E-05	16	14109740		
rs17619153	3.44142E-05	18	14071522	60	ZNF519
rs2422907	3.44142E-05	20	3583666		
rs12522552	3.46047E-05	5	107195846	32	FBXL17
rs7631341	3.52524E-05	3	74498821		
rs17158139	3.52611E-05	10	2362113	44	LINC00701
rs10910219	3.61358E-05	1	232463759	9	
rs1472201	3.61930E-05	4	77793375		
rs10754539	3.63027E-05	1	247365430		
rs1763148	3.66302E-05	14	52891060	49	
rs7312612	3.69814E-05	12	78640351	47	
rs2042962	3.87985E-05	5	31820098	29	
rs2072209	3.91006E-05	7	107592198		
rs6932267	3.94706E-05	6	96014686		
rs9889888	4.15967E-05	17	71499923		
rs2048957	4.16608E-05	2	144483781	12	ARHGAP15
rs10189240	4.16608E-05	2	144484493	12	ARHGAP15
rs12745451	4.16608E-05	1	88620063		
rs1417403	4.23108E-05	1	76454816	2	ASB17
rs6763449	4.29443E-05	3	86190433		
rs1349576	4.33801E-05	8	82890913		
rs11687496	4.38022E-05	2	68012667		
rs12741328	4.54852E-05	1	119537464	4	WARS2
rs10245130	4.55244E-05	7	78002024		
rs16961182	4.67867E-05	16	83688077	54	CDH13
rs17689520	4.67867E-05	16	83695851	54	CDH13
rs3891799	4.89769E-05	3	189161056	18	
rs10140562	4.90359E-05	14	52892530	49	
rs1864245	4.97682E-05	2	144778518	13	GTDC1
rs5977273	4.99561E-05	23	129552909		
rs705169	5.01669E-05	10	125285443		
rs16826860	5.08454E-05	2	231047624	14	SP110
rs10942140	5.08758E-05	5	25821882	28	
rs3846203	5.10572E-05	3	189155465	18	
rs12617864	5.18764E-05	2	113714976		
rs12547428	5.25650E-05	8	29741748		
rs2792664	5.45904E-05	1	76459445	2	ASB17
rs11264728	5.48266E-05	1	157401706	5	
rs12439707	5.50659E-05	15	59996367		
rs388248	5.51284E-05	5	25824908	28	

rs6416472	5.51292E-05	15	61863233	52	
rs12665113	5.54865E-05	6	67556820		
rs994239	5.60017E-05	1	105505952	3	MIR548H3
rs6771859	5.63348E-05	3	189159352	18	
rs9812880	5.63638E-05	3	193012791		
rs2298826	5.66040E-05	11	20659757		
rs16944501	5.68264E-05	13	91044545		
rs7027234	5.76606E-05	9	73712141		
rs895217	5.85556E-05	1	236496215	10	
rs6026227	5.87925E-05	20	56957174		
rs1561048	5.87925E-05	15	100415574		
rs3891798	5.97799E-05	3	189161117	18	
rs10802074	6.04737E-05	1	119624996	4	WARS2
rs1017639	6.05321E-05	11	68598534		
rs8100206	6.38543E-05	19	11764496		
rs10181734	6.39786E-05	2	235326046		
rs750678	6.53382E-05	14	93400879		
rs12652472	6.61714E-05	5	36374029		
rs1689512	6.64708E-05	12	56510637		
rs2362147	6.81370E-05	1	119764787	4	WARS2
rs7155768	6.85238E-05	14	98235200	50	
rs9935840	6.95044E-05	16	20225443		
rs1020104	6.98311E-05	23	12551001		
rs2236016	7.05907E-05	6	153313460	38	MTRF1L,RGS17
rs7523656	7.08989E-05	1	157402121	5	
rs1109214	7.12310E-05	1	119768928	4	WARS2
rs16856809	7.27016E-05	1	232455417	9	
rs4659446	7.33217E-05	1	236489984	10	
rs6796285	7.45336E-05	3	10998663		
rs12410373	7.50023E-05	1	76398249	2	ASB17
rs6685657	7.50023E-05	1	76417440	2	ASB17
rs7550524	7.50023E-05	1	105507848	3	MIR548H3
rs13098886	7.51213E-05	3	157613783	17	
rs1744494	7.63341E-05	6	165654440	39	
rs881249	7.63341E-05	1	112286948		
rs9291042	7.65130E-05	3	192790018		
rs1347251	7.68576E-05	1	153269184		
rs6455995	7.81357E-05	6	165646265	39	
rs1018356	7.81357E-05	8	134435107	41	
rs2056122	7.88626E-05	3	157591991	17	
rs4122014	7.92235E-05	6	148189157		
rs7147798	7.99075E-05	14	98230632	50	
rs12630040	8.04778E-05	3	71974395		
rs6882351	8.10092E-05	5	40481654		
rs1037955	8.10218E-05	6	107005165		
rs13274797	8.10946E-05	8	134462992	41	ZNF462
rs4690966	8.10981E-05	4	156353245	25	
rs7804535	8.20282E-05	7	103813522		
rs17030727	8.23587E-05	4	101963249	22	PPP3CA
rs10026319	8.23587E-05	4	101975635	22	PPP3CA
rs10003855	8.23587E-05	4	101975750	22	PPP3CA
rs10020845	8.23587E-05	4	101989580	22	PPP3CA
rs9371268	8.27188E-05	6	153153293		
rs17319422	8.36435E-05	8	3066608		
rs10025768	8.40770E-05	4	101965295	22	PPP3CA
rs12092756	8.53138E-05	1	18249743	1	
rs6708276	8.53138E-05	2	144442150	12	ARHGAP15
rs6812244	8.57588E-05	4	140705510	23	MAML3
rs4867084	8.75000E-05	5	31818948	29	
rs3862478	8.81017E-05	17	73568925		
rs11111712	8.85711E-05	12	104081723		
rs12030047	8.91483E-05	1	35160305		
rs11937871	8.96667E-05	4	104792456		

rs9884061	8.99000E-05	3	73689380		
rs8096880	9.03656E-05	18	28097568		
rs17243500	9.06158E-05	16	82578047		
rs9397578	9.19833E-05	6	153329508	38	MTRF1L,RGS17
rs503366	9.21022E-05	6	153333550	38	MTRF1L,RGS17
rs2042959	9.30065E-05	5	31819180	29	
rs6807028	9.39097E-05	3	65650920		
rs260090	9.42741E-05	15	99667524	53	SYNM,TTC23
rs637307	9.42741E-05	12	118139859		
rs1572576	9.42741E-05	6	125013995		
rs7930792	9.47075E-05	11	122581725	45	UBASH3B
rs16823373	9.54146E-05	2	144718006	13	GTDC1
rs2972102	9.58356E-05	7	147881158	40	MIR548T,CNTNAP2
rs1015931	9.58356E-05	7	147888914	40	MIR548T,CNTNAP2
rs1462266	9.58356E-05	14	98249402	50	
rs285969	9.58356E-05	6	83354055		
rs7966105	9.58356E-05	12	85577001		
rs7584354	9.61456E-05	2	55271875	11	RTN4
rs1384449	9.68541E-05	5	44377060		
rs883136	9.74231E-05	1	18253743	1	
rs2602027	9.74231E-05	15	99703772	53	SYNM,TTC23
rs610327	9.79457E-05	16	26811282		
rs6818783	9.90370E-05	4	40323397		
rs10771640	9.92397E-05	12	30299556	46	
rs901077	9.92397E-05	12	30300105	46	
rs2322101	9.92397E-05	18	5477881	59	EPB41L3
rs882630	9.92397E-05	21	47476248	63	
rs10817030	9.98792E-05	9	113245758		
rs2414719	9.98882E-05	15	61869851	52	

‡:Regions containing more than 2 SNPs with *P* value <0.0001 within 100 kb

*Reported SNPs

Yellow shows significant *P* value < 8.5×10^{-7} (Bonferroni corrected).

Blue shows validated SNPs in this study.

Chromosome locations are based on NCBI 37 Build.

ⓓ Imputation analysis

Chromosome	rs number	Position	A1	F_A	F_U	A2	CHISQ	*P* value	OR
11	rs36056670	70387145	G	0.457	0.3124	A	15.14	9.98E-05	1.852
6	rs114781599	31414082	T	0.2287	0.1233	G	15.14	9.97E-05	2.109
4	rs142875846	183346928	T	0.1968	0.09983	C	15.15	9.95E-05	2.21
4	rs76929118	183347530	G	0.1968	0.09983	A	15.15	9.95E-05	2.21
12	rs4620779	17886898	T	0.55	0.3956	G	15.15	9.94E-05	1.867
16	rs34927517	84377076	G	0.1209	0.04816	A	15.15	9.90E-05	2.717
6	rs115562658	32196864	G	0.3587	0.226	A	15.16	9.86E-05	1.915
11	rs12271322	70388251	C	0.4574	0.3133	T	15.17	9.85E-05	1.848
12	rs12302160	75088138	C	0.05435	0.0128	T	15.17	9.82E-05	4.433
4	rs1113098	44652187	C	0.2819	0.4324	G	15.17	9.80E-05	0.5153
4	rs1491315	44656980	T	0.2819	0.4324	C	15.17	9.80E-05	0.5153
4	rs1907489	44657191	C	0.2819	0.4324	A	15.17	9.80E-05	0.5153
4	rs10805130	44660570	C	0.2819	0.4324	T	15.17	9.80E-05	0.5153
1	rs12129670	201237806	T	0.07447	0.02245	C	15.18	9.79E-05	3.503
15	rs2404971	76602346	A	0.1277	0.05295	G	15.18	9.75E-05	2.617
20	rs8183394	8254575	T	0.2967	0.1745	A	15.19	9.74E-05	1.996
1	rs12042797	119680090	C	0.1436	0.06334	T	15.19	9.72E-05	2.48
9	rs17807662	113207450	T	0.1436	0.06334	C	15.19	9.72E-05	2.48
2	rs67143479	220171765	T	0.08065	0.02544	C	15.19	9.71E-05	3.361
21	rs56132061	20764084	T	0.233	0.1236	C	15.2	9.69E-05	2.153
16	rs4401054	20213695	T	0.3172	0.1918	C	15.21	9.62E-05	1.957
12	rs146364103	30325611	T	0.04787	0.01019	C	15.21	9.61E-05	4.885
12	rs10879824	75089621	A	0.05435	0.01278	G	15.21	9.61E-05	4.441
1	rs68126797	119701578	C	0.2151	0.1127	T	15.21	9.60E-05	2.157
13	rs57475038	23488737	C	0.3352	0.2057	A	15.22	9.59E-05	1.947
5	rs2973650	44392131	C	0.4239	0.2821	T	15.22	9.58E-05	1.873
2	rs56034474	235321386	A	0.1596	0.07376	C	15.22	9.56E-05	2.384
2	rs10165420	235321635	A	0.1596	0.07376	G	15.22	9.56E-05	2.384
2	rs7424598	235321769	A	0.1596	0.07376	G	15.22	9.56E-05	2.384
17	rs8072582	50826390	A	0.2872	0.1684	G	15.22	9.55E-05	1.991
17	rs139223735	50828814	A	0.2872	0.1684	C	15.22	9.55E-05	1.991
17	rs8067043	50854001	C	0.2849	0.1661	T	15.22	9.54E-05	2.001
17	rs8078281	50936108	A	0.2926	0.1726	T	15.23	9.54E-05	1.983
15	rs56372945	76603164	G	0.1277	0.05286	A	15.25	9.44E-05	2.622
15	rs57217518	76603169	C	0.1277	0.05286	T	15.25	9.44E-05	2.622
20	rs6140576	8248770	G	0.3032	0.181	C	15.25	9.42E-05	1.968
20	rs6133562	8249076	A	0.3032	0.181	G	15.25	9.42E-05	1.968
20	rs6133563	8249811	T	0.3032	0.181	C	15.25	9.42E-05	1.968
6	rs73004963	155443733	A	0.3667	0.231	G	15.26	9.39E-05	1.927
6	rs73004964	155443773	G	0.3667	0.231	A	15.26	9.39E-05	1.927
9	rs7873215	71971003	A	0.1022	0.03717	T	15.26	9.38E-05	2.947
5	rs4075367	2125330	T	0.3298	0.2027	C	15.26	9.35E-05	1.935
7	rs76493470	22214172	C	0.04839	0.01027	T	15.26	9.35E-05	4.898
2	rs74430915	143637397	C	0.1183	0.04693	T	15.28	9.27E-05	2.724
19	rs138569814	23729249	A	0.04787	0.01015	G	15.29	9.22E-05	4.902
6	rs115045353	31417159	A	0.2287	0.1229	G	15.29	9.22E-05	2.117
19	rs148229215	6171143	A	0.05435	0.01273	T	15.3	9.20E-05	4.456
17	rs56226662	50841857	C	0.2872	0.1681	T	15.3	9.17E-05	1.995
6	rs3818127	153323896	T	0.5	0.3514	C	15.3	9.15E-05	1.846
6	rs3818130	153324156	G	0.5	0.3514	A	15.3	9.15E-05	1.846
6	rs7752197	153326846	C	0.5	0.3514	T	15.3	9.15E-05	1.846
4	rs1979662	44650561	C	0.2819	0.4332	T	15.31	9.11E-05	0.5137
17	rs10515026	50850749	T	0.2872	0.1681	C	15.32	9.09E-05	1.995
14	rs1951642	38279008	T	0.1543	0.0701	C	15.33	9.05E-05	2.419
1	rs11264731	157416467	A	0.1517	0.06696	T	15.33	9.02E-05	2.492
17	rs55766844	13272398	A	0.1236	0.04912	G	15.34	9.00E-05	2.73
4	rs2218111	44634458	G	0.2819	0.4333	A	15.34	8.99E-05	0.5135
6	rs9347903	165114404	C	0.3533	0.5085	T	15.35	8.95E-05	0.5279
1	rs7525555	202139703	G	0.234	0.1267	C	15.35	8.94E-05	2.106
8	rs76250284	18650036	T	0.2204	0.1162	C	15.35	8.93E-05	2.15
4	rs6840406	140702650	T	0.1337	0.2735	G	15.35	8.92E-05	0.41
6	rs183785627	165127807	C	0.619	0.4567	T	15.36	8.87E-05	1.933
14	rs11626420	98228930	T	0.1755	0.08446	C	15.37	8.83E-05	2.308
14	rs7146763	98230310	G	0.1755	0.08446	A	15.37	8.83E-05	2.308
14	rs7147798	98230632	A	0.1755	0.08446	G	15.37	8.83E-05	2.308
14	rs7155566	98247629	C	0.1755	0.08446	T	15.37	8.83E-05	2.308

14	rs6575633	98247653	T	0.1755	0.08446	A	15.37	8.83E-05	2.308
14	rs7155086	98247914	A	0.1755	0.08446	G	15.37	8.83E-05	2.308
14	rs7159273	98248090	G	0.1755	0.08446	A	15.37	8.83E-05	2.308
14	rs7160424	98248676	G	0.1755	0.08446	A	15.37	8.83E-05	2.308
14	rs6575634	98248954	C	0.1755	0.08446	T	15.37	8.83E-05	2.308
14	rs6575635	98249039	A	0.1755	0.08446	G	15.37	8.83E-05	2.308
14	rs8006984	98249156	A	0.1755	0.08446	G	15.37	8.83E-05	2.308
19	rs11670722	49724946	A	0.08152	0.2026	G	15.37	8.83E-05	0.3494
2	rs114760725	206467565	G	0.1576	0.07167	T	15.37	8.82E-05	2.423
20	rs115766182	495229	T	0.133	0.05612	C	15.38	8.81E-05	2.579
17	rs73288064	32670789	A	0.5769	0.4211	T	15.38	8.80E-05	1.875
17	rs58786854	50855393	T	0.2903	0.1698	C	15.38	8.78E-05	2
2	rs10933339	231420072	G	0.2841	0.1622	C	15.4	8.72E-05	2.05
19	rs181413239	6152326	A	0.06915	0.01962	G	15.42	8.61E-05	3.711
5	rs59636641	20711994	A	0.5914	0.4366	G	15.42	8.60E-05	1.868
6	rs79556279	31329846	T	0.2012	0.09828	G	15.43	8.57E-05	2.311
19	rs73048802	49719419	G	0.08152	0.203	T	15.43	8.54E-05	0.3486
2	rs77675125	218186819	A	0.06452	0.01724	G	15.44	8.54E-05	3.931
5	rs66774244	20729096	T	0.587	0.4315	C	15.46	8.45E-05	1.873
1	rs67184413	157419845	T	0.15	0.06579	G	15.46	8.42E-05	2.506
6	rs78201106	141896930	A	0.1596	0.2981	G	15.46	8.41E-05	0.447
14	rs7160746	98249964	T	0.1755	0.08418	C	15.47	8.37E-05	2.316
14	rs6575637	98250269	A	0.1755	0.08418	G	15.47	8.37E-05	2.316
7	rs112853446	12728489	G	0.04891	0.01029	A	15.48	8.34E-05	4.946
15	rs6494316	62505352	C	0.2368	0.4038	T	15.48	8.32E-05	0.4582
17	rs5020101	50925745	A	0.2926	0.1717	G	15.49	8.31E-05	1.994
13	rs12583415	23492031	T	0.3297	0.2002	C	15.5	8.26E-05	1.965
9	rs10816461	109793425	A	0.2791	0.4388	G	15.51	8.20E-05	0.495
5	rs7706358	20730790	G	0.5924	0.4364	A	15.51	8.19E-05	1.877
20	rs67220553	8250530	G	0.3065	0.1823	A	15.51	8.19E-05	1.982
1	rs118129417	179070449	T	0.05851	0.01453	G	15.52	8.18E-05	4.215
14	rs10873485	98252737	T	0.1722	0.08062	C	15.53	8.11E-05	2.373
1	rs115342628	6102748	A	0.06383	0.01698	G	15.56	8.01E-05	3.948
1	rs2864705	24004099	C	0.06383	0.01698	T	15.56	8.01E-05	3.948
1	rs9427280	157405144	C	0.1383	0.05922	T	15.56	7.97E-05	2.55
1	rs2151164	157407528	T	0.1383	0.05922	A	15.56	7.97E-05	2.55
17	rs59762135	13264314	A	0.1237	0.04983	G	15.57	7.96E-05	2.691
17	rs7218413	13265478	T	0.1237	0.04983	C	15.57	7.96E-05	2.691
17	rs28602833	13271981	G	0.1237	0.04983	A	15.57	7.96E-05	2.691
17	rs10515027	50870949	T	0.2926	0.1715	C	15.58	7.90E-05	1.998
17	rs16952234	50871126	G	0.2926	0.1715	A	15.58	7.90E-05	1.998
17	rs60423449	50871731	C	0.2926	0.1715	T	15.58	7.90E-05	1.998
17	rs59962110	50872137	T	0.2926	0.1715	C	15.58	7.90E-05	1.998
17	rs73987837	50873271	A	0.2926	0.1715	G	15.58	7.90E-05	1.998
17	rs77690759	50873306	C	0.2926	0.1715	T	15.58	7.90E-05	1.998
17	rs57878695	50873734	A	0.2926	0.1715	G	15.58	7.90E-05	1.998
17	rs73987841	50874165	C	0.2926	0.1715	T	15.58	7.90E-05	1.998
17	rs73987843	50874296	G	0.2926	0.1715	T	15.58	7.90E-05	1.998
17	rs73987845	50874429	G	0.2926	0.1715	T	15.58	7.90E-05	1.998
17	rs73987846	50874597	T	0.2926	0.1715	C	15.58	7.90E-05	1.998
17	rs76910519	50874759	A	0.2926	0.1715	T	15.58	7.90E-05	1.998
17	rs55674028	50878378	A	0.2926	0.1715	G	15.58	7.90E-05	1.998
17	rs115063333	50878524	C	0.2926	0.1715	T	15.58	7.90E-05	1.998
17	rs76834193	50878843	T	0.2926	0.1715	G	15.58	7.90E-05	1.998
17	rs66538736	50879131	A	0.2926	0.1715	C	15.58	7.90E-05	1.998
17	rs59607819	50883246	G	0.2926	0.1715	A	15.58	7.90E-05	1.998
17	rs77647109	50883953	A	0.2926	0.1715	G	15.58	7.90E-05	1.998
17	rs115229770	50883970	G	0.2926	0.1715	A	15.58	7.90E-05	1.998
17	rs66726712	50886320	A	0.2926	0.1715	C	15.58	7.90E-05	1.998
17	rs76908161	50886334	T	0.2926	0.1715	G	15.58	7.90E-05	1.998
17	rs76836985	50886538	C	0.2926	0.1715	A	15.58	7.90E-05	1.998
17	rs117789675	50894614	A	0.2926	0.1715	G	15.58	7.90E-05	1.998
17	rs16952300	50896241	G	0.2926	0.1715	A	15.58	7.90E-05	1.998
17	rs16952310	50896403	C	0.2926	0.1715	T	15.58	7.90E-05	1.998
17	rs16952312	50896683	T	0.2926	0.1715	C	15.58	7.90E-05	1.998
17	rs80292875	50897610	T	0.2926	0.1715	A	15.58	7.90E-05	1.998
17	rs55944866	50898180	C	0.2926	0.1715	G	15.58	7.90E-05	1.998
17	rs2320096	50898467	C	0.2926	0.1715	G	15.58	7.90E-05	1.998
17	rs75002724	50899493	G	0.2926	0.1715	C	15.58	7.90E-05	1.998

17	rs16952328	50900820	C	0.2926	0.1715	T	15.58	7.90E-05	1.998
17	rs9807080	50906341	T	0.2926	0.1715	C	15.58	7.90E-05	1.998
17	rs8080294	50908543	A	0.2926	0.1715	G	15.58	7.90E-05	1.998
17	rs6504793	50909244	C	0.2926	0.1715	T	15.58	7.90E-05	1.998
17	rs8074354	50914295	T	0.2926	0.1715	A	15.58	7.90E-05	1.998
17	rs78467697	50914532	G	0.2926	0.1715	C	15.58	7.90E-05	1.998
17	rs74327153	50916697	A	0.2926	0.1715	G	15.58	7.90E-05	1.998
17	rs8081542	50917779	G	0.2926	0.1715	A	15.58	7.90E-05	1.998
17	rs12450164	50919575	C	0.2926	0.1715	G	15.58	7.90E-05	1.998
17	rs9906652	50919635	T	0.2926	0.1715	C	15.58	7.90E-05	1.998
17	rs4258667	50920459	C	0.2926	0.1715	T	15.58	7.90E-05	1.998
17	rs8073239	50923040	G	0.2926	0.1715	A	15.58	7.90E-05	1.998
17	rs79135083	50923559	C	0.2926	0.1715	T	15.58	7.90E-05	1.998
17	rs59844681	50924057	A	0.2926	0.1715	G	15.58	7.90E-05	1.998
20	rs6133557	8233121	G	0.2979	0.1757	A	15.59	7.87E-05	1.991
20	rs6140563	8233442	A	0.2979	0.1757	G	15.59	7.87E-05	1.991
20	rs111747627	8233843	A	0.2979	0.1757	C	15.59	7.87E-05	1.991
20	rs66510567	8234026	T	0.2979	0.1757	C	15.59	7.87E-05	1.991
20	rs66623426	8234945	C	0.2979	0.1757	T	15.59	7.87E-05	1.991
20	rs1997653	8235597	C	0.2979	0.1757	A	15.59	7.87E-05	1.991
20	rs67750658	8237193	G	0.2979	0.1757	C	15.59	7.87E-05	1.991
20	rs6140566	8237322	A	0.2979	0.1757	G	15.59	7.87E-05	1.991
20	rs2743159	8239108	C	0.2979	0.1757	G	15.59	7.87E-05	1.991
20	rs6140568	8239984	T	0.2979	0.1757	C	15.59	7.87E-05	1.991
20	rs6133558	8240297	A	0.2979	0.1757	G	15.59	7.87E-05	1.991
20	rs67066054	8240880	A	0.2979	0.1757	G	15.59	7.87E-05	1.991
20	rs67990733	8240983	C	0.2979	0.1757	G	15.59	7.87E-05	1.991
20	rs67818593	8241396	A	0.2979	0.1757	G	15.59	7.87E-05	1.991
20	rs6133559	8241522	T	0.2979	0.1757	C	15.59	7.87E-05	1.991
20	rs6133560	8241637	T	0.2979	0.1757	G	15.59	7.87E-05	1.991
20	rs67432256	8241990	A	0.2979	0.1757	G	15.59	7.87E-05	1.991
20	rs66936452	8242014	A	0.2979	0.1757	T	15.59	7.87E-05	1.991
20	rs6140570	8242639	A	0.2979	0.1757	T	15.59	7.87E-05	1.991
2	rs76179840	143710480	T	0.1183	0.04639	C	15.6	7.83E-05	2.757
2	rs80081139	218164323	G	0.05851	0.01448	T	15.6	7.82E-05	4.23
2	rs75651488	218170503	C	0.05851	0.01448	T	15.6	7.82E-05	4.23
19	rs76493785	6149349	T	0.06915	0.01949	A	15.61	7.80E-05	3.737
19	rs189804896	6152387	T	0.06915	0.01949	C	15.61	7.80E-05	3.737
19	rs12104257	6152992	T	0.06915	0.01949	C	15.61	7.80E-05	3.737
19	rs77751982	6155767	A	0.06915	0.01949	G	15.61	7.80E-05	3.737
5	rs28410044	20724069	T	0.5989	0.4412	G	15.62	7.73E-05	1.891
1	rs1889012	157401174	T	0.1383	0.05912	C	15.63	7.71E-05	2.554
1	rs11264728	157401706	G	0.1383	0.05912	A	15.63	7.71E-05	2.554
1	rs7523656	157402121	A	0.1383	0.05912	G	15.63	7.71E-05	2.554
1	rs11585245	157403321	A	0.1383	0.05912	G	15.63	7.71E-05	2.554
3	rs17022021	28878688	C	0.1702	0.08024	T	15.64	7.68E-05	2.351
2	rs78881937	218158778	A	0.05851	0.01446	T	15.65	7.64E-05	4.237
2	rs17797102	218159072	C	0.05851	0.01446	T	15.65	7.64E-05	4.237
2	rs79985817	218161917	C	0.05851	0.01446	T	15.65	7.64E-05	4.237
2	rs142573705	218162884	C	0.05851	0.01446	A	15.65	7.64E-05	4.237
2	rs74551905	218166144	T	0.05851	0.01446	A	15.65	7.64E-05	4.237
2	rs79348021	218169160	C	0.05851	0.01446	T	15.65	7.64E-05	4.237
2	rs116369935	218170037	T	0.05851	0.01446	C	15.65	7.64E-05	4.237
2	rs76697141	218170517	A	0.05851	0.01446	G	15.65	7.64E-05	4.237
2	rs78437976	218171909	G	0.05851	0.01446	C	15.65	7.64E-05	4.237
16	rs12932349	16975466	A	0.266	0.1503	G	15.65	7.61E-05	2.048
19	rs57830242	6149042	T	0.06915	0.01946	C	15.65	7.61E-05	3.743
19	rs79899501	6151479	T	0.06915	0.01946	C	15.65	7.61E-05	3.743
19	rs115943792	6156797	G	0.06915	0.01946	A	15.65	7.61E-05	3.743
17	rs8077546	50929438	C	0.2926	0.1712	G	15.66	7.60E-05	2.002
20	rs2246899	8246956	G	0.3032	0.1797	A	15.66	7.56E-05	1.987
5	rs55716018	2122640	C	0.3333	0.2036	A	15.69	7.48E-05	1.956
6	rs7751799	7503022	T	0.03723	0.005932	C	15.69	7.48E-05	6.481
2	rs74556304	218168900	G	0.05851	0.01443	A	15.69	7.46E-05	4.244
14	rs6575636	98249575	A	0.1755	0.08376	G	15.69	7.46E-05	2.329
14	rs4901284	38277999	T	0.1543	0.06937	C	15.7	7.44E-05	2.447
14	rs4901285	38278611	C	0.1543	0.06937	T	15.7	7.44E-05	2.447
14	rs34550574	38280442	T	0.1543	0.06937	C	15.7	7.44E-05	2.447
19	rs10421054	6158235	C	0.07447	0.02203	T	15.71	7.39E-05	3.571

2	rs78028445	101667239	A	0.03723	0.005922	G	15.72	7.33E-05	6.492
2	rs149925100	101667488	A	0.03723	0.005922	G	15.72	7.33E-05	6.492
2	rs138786122	101668299	T	0.03723	0.005922	G	15.72	7.33E-05	6.492
2	rs189099047	101668993	A	0.03723	0.005922	C	15.72	7.33E-05	6.492
2	rs116058758	101669127	T	0.03723	0.005922	C	15.72	7.33E-05	6.492
2	rs181787080	101674082	A	0.03723	0.005922	G	15.72	7.33E-05	6.492
2	rs189973920	101674967	G	0.03723	0.005922	C	15.72	7.33E-05	6.492
2	rs139224819	101675585	C	0.03723	0.005922	G	15.72	7.33E-05	6.492
2	rs186608537	101676478	A	0.03723	0.005922	G	15.72	7.33E-05	6.492
2	rs139898765	101676581	T	0.03723	0.005922	C	15.72	7.33E-05	6.492
2	rs190389470	101677604	T	0.03723	0.005922	C	15.72	7.33E-05	6.492
2	rs188283473	101681175	A	0.03723	0.005922	G	15.72	7.33E-05	6.492
2	rs184780259	101681484	A	0.03723	0.005922	G	15.72	7.33E-05	6.492
2	rs188822583	101690360	G	0.03723	0.005922	C	15.72	7.33E-05	6.492
2	rs147459701	101692051	C	0.03723	0.005922	T	15.72	7.33E-05	6.492
2	rs191355441	101693509	A	0.03723	0.005922	G	15.72	7.33E-05	6.492
2	rs187040766	101693671	C	0.03723	0.005922	G	15.72	7.33E-05	6.492
2	rs76969563	101695630	T	0.03723	0.005922	A	15.72	7.33E-05	6.492
12	rs11614670	118940840	G	0.07979	0.0247	T	15.73	7.31E-05	3.423
2	rs74876733	218158701	T	0.05851	0.01441	G	15.73	7.29E-05	4.252
2	rs76208585	218162171	T	0.05851	0.01441	A	15.73	7.29E-05	4.252
2	rs115887468	218163450	C	0.05851	0.01441	G	15.73	7.29E-05	4.252
2	rs77068672	218164589	A	0.05851	0.01441	G	15.73	7.29E-05	4.252
2	rs116507753	218165263	G	0.05851	0.01441	A	15.73	7.29E-05	4.252
2	rs116368533	218165515	C	0.05851	0.01441	T	15.73	7.29E-05	4.252
2	rs114471903	218165867	T	0.05851	0.01441	C	15.73	7.29E-05	4.252
2	rs75180113	218166735	A	0.05851	0.01441	G	15.73	7.29E-05	4.252
2	rs115020924	218167090	A	0.05851	0.01441	G	15.73	7.29E-05	4.252
2	rs79333697	218167517	C	0.05851	0.01441	A	15.73	7.29E-05	4.252
2	rs115933559	218172162	A	0.05851	0.01441	G	15.73	7.29E-05	4.252
2	rs55771914	218172370	C	0.05851	0.01441	T	15.73	7.29E-05	4.252
2	rs75134266	218173583	G	0.05851	0.01441	A	15.73	7.29E-05	4.252
2	rs115452488	218173855	T	0.05851	0.01441	G	15.73	7.29E-05	4.252
14	rs34603503	98253800	A	0.1685	0.07818	G	15.74	7.27E-05	2.389
14	rs36019608	98232142	C	0.172	0.08092	A	15.75	7.24E-05	2.36
17	rs142795777	50871755	G	0.2926	0.1709	A	15.75	7.22E-05	2.006
17	rs67354784	50885642	A	0.2926	0.1709	T	15.75	7.22E-05	2.006
20	rs2743169	8244884	G	0.2979	0.1751	A	15.75	7.21E-05	1.998
20	rs2743171	8245177	G	0.2979	0.1751	C	15.75	7.21E-05	1.998
20	rs2743172	8245254	G	0.2979	0.1751	A	15.75	7.21E-05	1.998
20	rs6140573	8245274	G	0.2979	0.1751	A	15.75	7.21E-05	1.998
2	rs112125503	101699040	G	0.03723	0.005912	A	15.76	7.19E-05	6.503
2	rs80100952	101699127	C	0.03723	0.005912	T	15.76	7.19E-05	6.503
2	rs189307225	101699785	A	0.03723	0.005912	T	15.76	7.19E-05	6.503
2	rs113386984	101704796	A	0.03723	0.005912	G	15.76	7.19E-05	6.503
2	rs146535503	101707043	G	0.03723	0.005912	C	15.76	7.19E-05	6.503
2	rs188841187	101707702	A	0.03723	0.005912	G	15.76	7.19E-05	6.503
2	rs141538962	101707797	C	0.03723	0.005912	A	15.76	7.19E-05	6.503
2	rs183857817	101710512	T	0.03723	0.005912	C	15.76	7.19E-05	6.503
2	rs148740470	101714388	A	0.03723	0.005912	G	15.76	7.19E-05	6.503
2	rs184987683	101716316	C	0.03723	0.005912	A	15.76	7.19E-05	6.503
2	rs188288314	101716535	T	0.03723	0.005912	C	15.76	7.19E-05	6.503
2	rs188796686	101720133	T	0.03723	0.005912	C	15.76	7.19E-05	6.503
2	rs181569394	101720391	T	0.03723	0.005912	C	15.76	7.19E-05	6.503
2	rs149308652	101725248	T	0.03723	0.005912	C	15.76	7.19E-05	6.503
2	rs143549500	101725304	C	0.03723	0.005912	G	15.76	7.19E-05	6.503
2	rs148114586	101725699	C	0.03723	0.005912	T	15.76	7.19E-05	6.503
2	rs185645417	101726961	A	0.03723	0.005912	G	15.76	7.19E-05	6.503
15	rs150324558	79539697	C	0.03723	0.005912	T	15.76	7.19E-05	6.503
14	rs7156945	98235660	A	0.1755	0.08361	G	15.76	7.18E-05	2.333
1	rs72994370	119900942	G	0.05319	0.01201	A	15.77	7.15E-05	4.623
1	rs58301273	119901641	C	0.05319	0.01201	G	15.77	7.15E-05	4.623
1	rs72994373	119902005	T	0.05319	0.01201	G	15.77	7.15E-05	4.623
1	rs10923765	119719666	T	0.2222	0.1153	G	15.78	7.10E-05	2.192
3	rs11129817	39289725	C	0.3298	0.2008	G	15.79	7.10E-05	1.958
5	rs7723872	20714239	G	0.587	0.4298	A	15.79	7.06E-05	1.885
1	rs117467628	191330540	A	0.04891	0.01015	G	15.8	7.03E-05	5.014
3	rs111608980	194480258	A	0.07447	0.02196	G	15.8	7.03E-05	3.584
1	rs2993426	202165814	A	0.2337	0.1243	G	15.81	7.02E-05	2.148

2	rs13417123	101672623	A	0.03763	0.005963	G	15.81	7.01E-05	6.52
2	rs10195965	101677626	A	0.03763	0.005963	G	15.81	7.01E-05	6.52
2	rs72937659	206462143	T	0.1576	0.07082	G	15.81	7.01E-05	2.455
12	rs1617905	78378699	C	0.3987	0.248	T	15.82	6.97E-05	2.011
1	rs2225573	157419719	T	0.1517	0.06591	C	15.84	6.89E-05	2.534
17	rs28886065	50840942	A	0.2903	0.1684	G	15.84	6.89E-05	2.021
4	rs79022743	188288238	T	0.03763	0.005952	G	15.85	6.87E-05	6.531
4	rs186140479	188288543	C	0.03763	0.005952	A	15.85	6.87E-05	6.531
9	rs192803058	34283351	C	0.03763	0.005952	T	15.85	6.87E-05	6.531
19	rs149957579	6157206	A	0.06989	0.01952	G	15.86	6.82E-05	3.774
19	rs77855792	6157538	T	0.06989	0.01952	C	15.86	6.82E-05	3.774
1	rs72704594	157419863	T	0.15	0.06503	C	15.86	6.82E-05	2.537
14	rs7140759	98250165	C	0.1755	0.08333	T	15.87	6.78E-05	2.342
22	rs117889307	24232856	G	0.05914	0.01448	T	15.88	6.75E-05	4.278
22	rs117761922	24233605	A	0.05914	0.01448	C	15.88	6.75E-05	4.278
2	rs142636442	101719225	T	0.03763	0.005942	C	15.88	6.74E-05	6.542
6	rs149293861	7502561	A	0.03763	0.005942	G	15.88	6.74E-05	6.542
7	rs117317993	137073962	A	0.2303	0.1203	C	15.89	6.73E-05	2.188
3	rs6599017	39287342	C	0.3298	0.2005	T	15.89	6.72E-05	1.962
3	rs3953248	39288676	T	0.3298	0.2005	C	15.89	6.72E-05	1.962
3	rs9835206	39289205	G	0.3298	0.2005	A	15.89	6.72E-05	1.962
3	rs9835335	39289218	G	0.3298	0.2005	A	15.89	6.72E-05	1.962
3	rs9821993	39289603	C	0.3298	0.2005	T	15.89	6.72E-05	1.962
3	rs9877538	39289664	A	0.3298	0.2005	C	15.89	6.72E-05	1.962
3	rs9839675	39289693	G	0.3298	0.2005	A	15.89	6.72E-05	1.962
3	rs9822146	39289697	C	0.3298	0.2005	T	15.89	6.72E-05	1.962
3	rs9822477	39289829	C	0.3298	0.2005	T	15.89	6.72E-05	1.962
3	rs9878455	39290215	T	0.3298	0.2005	C	15.89	6.72E-05	1.962
3	rs28391240	39290294	C	0.3298	0.2005	G	15.89	6.72E-05	1.962
3	rs9844952	39290524	G	0.3298	0.2005	A	15.89	6.72E-05	1.962
3	rs9882968	39290544	T	0.3298	0.2005	G	15.89	6.72E-05	1.962
3	rs9827570	39290709	C	0.3298	0.2005	T	15.89	6.72E-05	1.962
3	rs9845278	39290716	G	0.3298	0.2005	A	15.89	6.72E-05	1.962
3	rs9827771	39290859	C	0.3298	0.2005	T	15.89	6.72E-05	1.962
3	rs9883489	39290902	A	0.3298	0.2005	G	15.89	6.72E-05	1.962
3	rs9883618	39290982	T	0.3298	0.2005	G	15.89	6.72E-05	1.962
1	rs664443	191389398	G	0.06452	0.01695	A	15.89	6.71E-05	4
4	rs8180313	156346082	G	0.4892	0.3382	A	15.89	6.70E-05	1.874
14	rs1272513	21069321	A	0.1117	0.2424	G	15.92	6.61E-05	0.393
2	rs191941010	101709524	T	0.03763	0.005932	C	15.92	6.61E-05	6.553
17	rs16952221	50868681	G	0.2926	0.1703	C	15.92	6.60E-05	2.014
17	rs9709437	50869297	C	0.2926	0.1703	T	15.92	6.60E-05	2.014
17	rs16952227	50869835	G	0.2926	0.1703	A	15.92	6.60E-05	2.014
17	rs16952231	50870335	C	0.2926	0.1703	T	15.92	6.60E-05	2.014
17	rs6416843	72532447	C	0.3495	0.2161	T	15.93	6.57E-05	1.949
14	rs17179532	38276846	A	0.1559	0.06973	T	15.94	6.55E-05	2.464
14	rs12897031	38277316	G	0.1559	0.06973	A	15.94	6.55E-05	2.464
17	rs75981961	50820671	T	0.2903	0.1681	A	15.94	6.55E-05	2.025
3	rs9850385	39291616	G	0.3298	0.2002	A	15.94	6.54E-05	1.966
20	rs12625429	464877	C	0.129	0.05241	T	15.96	6.47E-05	2.679
5	rs72736816	35287615	G	0.09677	0.03327	T	15.97	6.44E-05	3.113
5	rs10070910	116013795	C	0.1862	0.09052	T	15.97	6.43E-05	2.298
7	rs76244705	137063161	T	0.2303	0.1201	C	15.97	6.43E-05	2.192
2	rs2292601	220172731	G	0.08065	0.0246	A	15.98	6.41E-05	3.477
2	rs36001784	220173035	A	0.08065	0.0246	G	15.98	6.41E-05	3.477
8	rs78263509	18655506	C	0.2181	0.1132	T	15.98	6.41E-05	2.185
8	rs79768913	18655545	T	0.2181	0.1132	C	15.98	6.41E-05	2.185
12	rs795950	78632036	G	0.07979	0.02449	A	15.98	6.40E-05	3.453
12	rs79848518	78634533	T	0.07979	0.02449	C	15.98	6.40E-05	3.453
12	rs74482041	78635281	C	0.07979	0.02449	T	15.98	6.40E-05	3.453
12	rs77848659	78635436	A	0.07979	0.02449	G	15.98	6.40E-05	3.453
12	rs74678292	78637779	C	0.07979	0.02449	A	15.98	6.40E-05	3.453
6	rs1572605	141995944	G	0.1505	0.291	C	15.99	6.35E-05	0.4319
6	rs57437386	141997575	G	0.1505	0.291	C	15.99	6.35E-05	0.4319
6	rs11155199	141998379	A	0.1505	0.291	G	15.99	6.35E-05	0.4319
6	rs12198661	141998749	T	0.1505	0.291	C	15.99	6.35E-05	0.4319
4	rs6536073	156404399	T	0.5	0.3476	A	16.01	6.29E-05	1.877
17	rs76957178	50798788	C	0.2872	0.1658	T	16.01	6.29E-05	2.027
5	rs12651945	148865624	G	0.07447	0.02163	A	16.02	6.25E-05	3.64

1	rs35404519	119669262	A	0.2181	0.1132	C	16.05	6.18E-05	2.186
1	rs2498500	119673093	T	0.2181	0.1132	C	16.05	6.18E-05	2.186
5	rs10062900	116013764	T	0.1862	0.09037	C	16.05	6.18E-05	2.303
21	rs77067815	23041067	A	0.5291	0.3673	C	16.06	6.12E-05	1.935
1	rs112289021	231523347	C	0.04255	0.00764	T	16.08	6.07E-05	5.773
6	rs116617589	31238815	G	0.4494	0.2899	A	16.09	6.05E-05	1.999
5	rs11954337	116014884	A	0.1828	0.08759	G	16.09	6.03E-05	2.33
5	rs76906740	116015130	A	0.1828	0.08759	G	16.09	6.03E-05	2.33
20	rs6133736	9653962	C	0.5798	0.4231	T	16.12	5.94E-05	1.881
12	rs7975464	118915101	A	0.08602	0.0274	G	16.13	5.92E-05	3.341
17	rs6504792	50908074	C	0.2957	0.1717	A	16.14	5.89E-05	2.025
1	rs56130333	119599808	T	0.2074	0.1051	C	16.14	5.88E-05	2.228
6	rs1265047	31081464	G	0.4731	0.3225	A	16.15	5.85E-05	1.887
13	rs7320661	102125387	A	0.09677	0.03322	G	16.15	5.85E-05	3.118
13	rs6491623	102125408	A	0.09677	0.03322	G	16.15	5.85E-05	3.118
2	rs56291674	36041356	A	0.6312	0.4603	C	16.16	5.84E-05	2.007
12	rs76945581	77454963	T	0.04255	0.007614	C	16.16	5.83E-05	5.793
6	rs117418064	148962414	A	0.0989	0.03407	G	16.17	5.78E-05	3.112
6	rs9403971	148962709	A	0.0989	0.03407	G	16.17	5.78E-05	3.112
3	rs9883690	39291200	T	0.3298	0.1995	C	16.18	5.77E-05	1.975
12	rs7295514	77465171	C	0.04255	0.007601	G	16.2	5.71E-05	5.802
12	rs75722470	77465601	G	0.04255	0.007601	A	16.2	5.71E-05	5.802
12	rs17042843	77466341	G	0.04255	0.007601	A	16.2	5.71E-05	5.802
17	rs8077578	72533640	C	0.3478	0.2132	T	16.2	5.71E-05	1.968
1	rs35608803	157420307	C	0.15	0.06437	T	16.2	5.70E-05	2.565
1	rs2077566	202164063	T	0.2389	0.1263	C	16.2	5.70E-05	2.171
12	rs751353	52583101	T	0.4091	0.2617	G	16.2	5.69E-05	1.953
6	rs73390248	31329834	T	0.2099	0.1019	C	16.23	5.62E-05	2.341
4	rs2341602	156407800	C	0.5	0.3466	T	16.23	5.60E-05	1.886
6	rs78075329	141948415	G	0.1489	0.2895	A	16.24	5.58E-05	0.4295
6	rs77340238	141955813	A	0.1489	0.2895	G	16.24	5.58E-05	0.4295
12	rs75772217	111732782	A	0.06915	0.01887	G	16.25	5.54E-05	3.863
4	rs6827464	156350128	T	0.5	0.347	C	16.26	5.51E-05	1.882
2	rs4527192	235321998	C	0.117	0.04492	T	16.27	5.49E-05	2.818
19	rs114962168	6162047	G	0.06044	0.01458	A	16.28	5.47E-05	4.348
19	rs149504864	6162401	A	0.06044	0.01458	C	16.28	5.47E-05	4.348
19	rs76769540	6165578	T	0.06044	0.01458	A	16.28	5.47E-05	4.348
19	rs75463840	6165837	A	0.06044	0.01458	G	16.28	5.47E-05	4.348
19	rs61707093	6166815	T	0.06044	0.01458	A	16.28	5.47E-05	4.348
6	rs9403968	148941986	A	0.1022	0.03602	G	16.28	5.46E-05	3.045
8	rs34857327	127938456	G	0.2093	0.3666	A	16.28	5.46E-05	0.4573
12	rs11608836	118922468	A	0.08602	0.02726	G	16.28	5.45E-05	3.359
12	rs11615103	118923079	C	0.08602	0.02726	A	16.28	5.45E-05	3.359
12	rs7485910	118923239	T	0.08602	0.02726	C	16.28	5.45E-05	3.359
12	rs7485870	118923775	A	0.08602	0.02726	G	16.28	5.45E-05	3.359
5	rs78957287	84726769	C	0.04301	0.007653	T	16.29	5.44E-05	5.828
5	rs78469872	84727204	A	0.04301	0.007653	G	16.29	5.44E-05	5.828
5	rs7709193	20709756	A	0.5914	0.4325	C	16.29	5.43E-05	1.899
4	rs4690966	156353245	G	0.5	0.3471	A	16.3	5.40E-05	1.881
17	rs6416844	72532515	A	0.3548	0.2191	T	16.31	5.38E-05	1.961
8	rs72718252	134470533	C	0.05435	0.01201	A	16.32	5.36E-05	4.729
19	rs191015007	23751704	G	0.04301	0.00764	A	16.33	5.33E-05	5.838
4	rs138714883	179435377	C	0.1935	0.09429	G	16.38	5.19E-05	2.305
1	rs12564953	119665129	G	0.2181	0.1123	T	16.39	5.17E-05	2.204
1	rs7528295	202140228	G	0.2181	0.1123	A	16.39	5.17E-05	2.204
17	rs189735752	50846897	A	0.2849	0.1624	C	16.39	5.16E-05	2.055
17	rs9903132	50835641	T	0.2926	0.1689	C	16.39	5.16E-05	2.035
17	rs16952207	50866087	C	0.2926	0.1689	T	16.39	5.16E-05	2.035
2	rs10181734	235326046	T	0.117	0.04476	A	16.39	5.15E-05	2.828
2	rs116572694	218216163	G	0.1596	0.07143	T	16.39	5.15E-05	2.468
5	rs7444742	20718866	G	0.5978	0.4373	C	16.42	5.06E-05	1.913
1	rs10802077	119710342	C	0.2356	0.1222	T	16.45	4.98E-05	2.215
4	rs7677803	156409087	A	0.5053	0.3514	G	16.46	4.98E-05	1.886
1	rs2362144	119727725	T	0.2222	0.1136	C	16.46	4.97E-05	2.229
1	rs2362145	119728582	A	0.2222	0.1136	T	16.46	4.97E-05	2.229
4	rs1599232	156411343	G	0.5165	0.3595	A	16.48	4.91E-05	1.903
1	rs3120747	202163621	A	0.2366	0.125	G	16.5	4.86E-05	2.169
4	rs10011941	156398437	G	0.5	0.3455	A	16.51	4.83E-05	1.894
17	rs80024245	50863644	A	0.2872	0.1641	G	16.56	4.73E-05	2.053

11	rs117167825	58961131	A	0.04839	0.009483	C	16.57	4.68E-05	5.311
12	rs76594900	29360223	G	0.06548	0.1945	A	16.58	4.67E-05	0.2902
20	rs2235812	49902060	C	0.06915	0.01864	A	16.59	4.64E-05	3.91
12	rs12827133	118926804	A	0.08696	0.02726	T	16.63	4.55E-05	3.399
19	rs78940112	6135231	A	0.06915	0.01861	C	16.64	4.52E-05	3.917
1	rs4950773	202137090	T	0.2181	0.1117	C	16.65	4.50E-05	2.219
1	rs4950831	202137318	A	0.2181	0.1117	G	16.65	4.50E-05	2.219
6	rs7770810	141992625	G	0.1489	0.2916	A	16.65	4.50E-05	0.4251
6	rs1577948	141994355	G	0.1489	0.2916	A	16.65	4.50E-05	0.4251
1	rs184047596	179154213	A	0.05851	0.0137	G	16.66	4.46E-05	4.475
4	rs146800989	179434085	T	0.1935	0.09359	C	16.69	4.41E-05	2.324
4	rs188329653	179434353	A	0.1935	0.09359	C	16.69	4.41E-05	2.324
4	rs75843115	179434505	G	0.1935	0.09359	A	16.69	4.41E-05	2.324
14	rs12891366	98231278	A	0.1755	0.08163	T	16.69	4.40E-05	2.395
14	rs7144057	98233489	T	0.1755	0.08163	C	16.69	4.40E-05	2.395
6	rs2233965	31080899	G	0.4096	0.2648	T	16.69	4.39E-05	1.926
1	rs7550457	202140315	A	0.2181	0.1115	C	16.73	4.31E-05	2.223
1	rs7550551	202140400	A	0.2181	0.1115	G	16.73	4.31E-05	2.223
4	rs6536074	156408000	G	0.4688	0.3059	A	16.74	4.28E-05	2.002
6	rs11155198	141994070	A	0.1489	0.2921	G	16.75	4.28E-05	0.4241
4	rs10032772	156345224	G	0.5	0.3452	A	16.75	4.27E-05	1.897
1	rs3010090	202164837	T	0.2366	0.1244	C	16.77	4.22E-05	2.182
2	rs116629128	218212108	C	0.1596	0.07069	G	16.78	4.20E-05	2.496
12	rs79748929	78624800	G	0.07979	0.02369	C	16.79	4.17E-05	3.573
12	rs79053063	112550965	G	0.05851	0.01363	A	16.8	4.15E-05	4.498
6	chr6:31080828	31080828	G	0.4096	0.2644	C	16.82	4.12E-05	1.93
17	rs10852753	72536009	T	0.3407	0.2046	C	16.82	4.11E-05	2.008
12	rs77260502	78612649	A	0.07979	0.02365	G	16.84	4.06E-05	3.58
12	rs1726423	78621072	T	0.07979	0.02365	G	16.84	4.06E-05	3.58
4	rs7442314	156399621	T	0.5	0.3446	C	16.89	3.97E-05	1.902
5	rs13164311	116018296	C	0.1828	0.08588	T	16.89	3.96E-05	2.381
17	rs72852232	72513553	T	0.3424	0.2063	A	16.93	3.89E-05	2.003
12	rs73205612	112251362	T	0.05851	0.01356	C	16.94	3.87E-05	4.521
12	rs73205625	112335205	C	0.05851	0.01356	T	16.94	3.87E-05	4.521
12	rs73205697	112392527	G	0.05851	0.01356	A	16.94	3.87E-05	4.521
7	rs79178345	12726754	A	0.04891	0.009434	G	16.97	3.80E-05	5.4
14	rs7155768	98235200	T	0.1755	0.08108	C	16.99	3.76E-05	2.413
1	rs3100127	202160970	A	0.2366	0.1237	C	16.99	3.75E-05	2.195
12	rs11068956	118904273	T	0.08602	0.0265	G	17	3.75E-05	3.458
12	rs113642960	118914598	T	0.08602	0.0265	G	17	3.75E-05	3.458
12	rs7978865	118915548	A	0.08602	0.0265	G	17	3.75E-05	3.458
17	rs73370729	50885064	T	0.3125	0.1793	C	17.01	3.72E-05	2.081
1	rs2361422	202158670	T	0.2366	0.1237	G	17.02	3.71E-05	2.195
1	rs4511180	202149439	A	0.234	0.1222	G	17.02	3.70E-05	2.195
12	rs1689512	56510637	G	0.08511	0.02618	A	17.03	3.69E-05	3.46
6	rs77991326	142007819	C	0.1413	0.2861	T	17.04	3.65E-05	0.4107
6	rs79207474	142017086	A	0.1413	0.2861	G	17.04	3.65E-05	0.4107
6	rs76583605	142018339	A	0.1413	0.2861	G	17.04	3.65E-05	0.4107
12	rs11068965	118925265	G	0.08602	0.02645	A	17.05	3.64E-05	3.464
16	rs12597430	16970563	G	0.2418	0.1269	T	17.05	3.63E-05	2.194
1	rs7529073	214147889	T	0.2957	0.4573	C	17.06	3.62E-05	0.4982
17	rs2134849	72515689	T	0.3424	0.2058	C	17.07	3.61E-05	2.009
2	rs76242095	67499689	C	0.2079	0.1011	T	17.07	3.60E-05	2.334
1	rs4397705	202140638	T	0.2253	0.1144	C	17.07	3.60E-05	2.252
12	rs7316206	75074154	C	0.05914	0.01363	T	17.09	3.56E-05	4.549
12	rs11180157	75077344	C	0.05914	0.01363	T	17.09	3.56E-05	4.549
12	rs71444511	118919098	A	0.08602	0.02641	T	17.1	3.54E-05	3.47
13	rs4624035	36759154	T	0.08602	0.02641	C	17.1	3.54E-05	3.47
4	rs6447363	44648797	G	0.2717	0.4332	A	17.13	3.49E-05	0.4883
12	rs12308319	75057931	T	0.05914	0.01361	A	17.14	3.48E-05	4.557
12	rs7966232	75061760	T	0.05914	0.01361	A	17.14	3.48E-05	4.557
12	rs11836636	75063110	G	0.05914	0.01361	A	17.14	3.48E-05	4.557
12	rs73357962	75064402	T	0.05914	0.01361	C	17.14	3.48E-05	4.557
8	rs7004093	134462291	A	0.05978	0.01375	G	17.16	3.44E-05	4.562
17	rs4789078	72554661	G	0.3916	0.2393	A	17.2	3.36E-05	2.046
14	rs60999667	76559083	A	0.1613	0.07082	G	17.22	3.32E-05	2.523
12	rs79953109	78595873	A	0.07065	0.01861	C	17.27	3.24E-05	4.009
12	rs80320430	78596881	A	0.07065	0.01861	G	17.27	3.24E-05	4.009
12	rs78376609	78599458	A	0.07065	0.01861	T	17.27	3.24E-05	4.009

12	rs75592089	78599646	G	0.07065	0.01861	A	17.27	3.24E-05	4.009
12	rs80157427	78601154	C	0.07065	0.01861	T	17.27	3.24E-05	4.009
17	rs9892631	13262589	T	0.1277	0.04983	A	17.29	3.21E-05	2.79
17	rs16947730	13267668	A	0.1277	0.04983	G	17.29	3.21E-05	2.79
17	rs9912964	13268953	A	0.1277	0.04983	C	17.29	3.21E-05	2.79
17	rs9906582	13269816	G	0.1277	0.04983	A	17.29	3.21E-05	2.79
17	rs8081684	13269942	G	0.1277	0.04983	A	17.29	3.21E-05	2.79
17	rs8064586	13269991	C	0.1277	0.04983	T	17.29	3.21E-05	2.79
17	rs8081985	13270064	G	0.1277	0.04983	A	17.29	3.21E-05	2.79
17	rs76729416	13270065	C	0.1277	0.04983	A	17.29	3.21E-05	2.79
17	rs933309	13270532	C	0.1277	0.04983	T	17.29	3.21E-05	2.79
17	rs933310	13270658	G	0.1277	0.04983	C	17.29	3.21E-05	2.79
17	rs41372750	13271274	C	0.1277	0.04983	G	17.29	3.21E-05	2.79
17	rs9896094	13271616	C	0.1277	0.04983	T	17.29	3.21E-05	2.79
17	rs9894432	13271712	C	0.1277	0.04983	A	17.29	3.21E-05	2.79
2	rs4663406	235322699	A	0.1398	0.05672	C	17.35	3.11E-05	2.703
17	rs72852298	72529956	T	0.3564	0.2173	G	17.37	3.08E-05	1.994
15	rs59467462	76605942	A	0.1277	0.04948	G	17.39	3.04E-05	2.811
12	rs12816015	118926138	G	0.08696	0.02645	A	17.4	3.03E-05	3.505
14	rs11625364	98236137	A	0.1755	0.08024	T	17.42	3.00E-05	2.441
2	rs72937683	206485041	C	0.1576	0.06775	A	17.43	2.98E-05	2.574
2	rs60864133	206486376	T	0.1576	0.06775	A	17.43	2.98E-05	2.574
17	rs72852234	72518243	C	0.3564	0.2171	A	17.46	2.94E-05	1.997
17	rs1472726	72519798	T	0.3564	0.2171	C	17.46	2.94E-05	1.997
17	rs10512596	72523224	C	0.3564	0.2171	T	17.46	2.94E-05	1.997
17	rs4789072	72526863	A	0.3564	0.2171	G	17.46	2.94E-05	1.997
17	rs4788843	72528091	T	0.3564	0.2171	C	17.46	2.94E-05	1.997
17	rs2134848	72529262	C	0.3564	0.2171	T	17.46	2.94E-05	1.997
17	rs2382846	72530447	C	0.3564	0.2171	T	17.46	2.94E-05	1.997
17	rs8081411	72531523	G	0.3564	0.2171	A	17.46	2.94E-05	1.997
17	rs4789074	72532409	G	0.3564	0.2171	A	17.46	2.94E-05	1.997
17	rs8067152	72532940	A	0.3564	0.2171	G	17.46	2.94E-05	1.997
17	rs11652446	72533076	A	0.3564	0.2171	C	17.46	2.94E-05	1.997
17	rs8073796	72533728	G	0.3564	0.2171	A	17.46	2.94E-05	1.997
17	rs6501722	72533893	C	0.3564	0.2171	T	17.46	2.94E-05	1.997
1	rs340882	214145731	G	0.2766	0.4383	C	17.47	2.92E-05	0.4899
6	rs3132558	31105466	C	0.3036	0.168	G	17.47	2.91E-05	2.159
3	rs9822525	39289906	C	0.3261	0.191	T	17.49	2.89E-05	2.049
17	rs8079734	72531557	A	0.3602	0.2193	G	17.55	2.80E-05	2.005
4	rs12651596	156389851	C	0.4881	0.3228	T	17.57	2.77E-05	2
6	rs145662636	31169549	A	0.1778	0.0802	G	17.58	2.76E-05	2.48
17	rs55792225	72531334	A	0.3564	0.2166	G	17.59	2.73E-05	2.003
17	rs113598994	72529750	A	0.3478	0.2085	C	17.6	2.73E-05	2.025
14	rs12884354	98237577	T	0.1755	0.0798	C	17.62	2.70E-05	2.455
14	rs1462268	98237766	T	0.1755	0.0798	C	17.62	2.70E-05	2.455
14	rs35267087	98240814	T	0.1755	0.0798	C	17.62	2.70E-05	2.455
14	rs34551620	98241523	T	0.1755	0.0798	C	17.62	2.70E-05	2.455
6	rs9403966	148929217	G	0.1087	0.0378	C	17.66	2.64E-05	3.104
15	rs2469195	77259486	C	0.3511	0.212	T	17.66	2.64E-05	2.011
12	rs11615133	118901915	T	0.08602	0.02577	C	17.69	2.60E-05	3.558
17	rs9897032	13266853	G	0.129	0.04983	A	17.72	2.56E-05	2.825
17	rs8081983	13270020	T	0.129	0.04983	C	17.72	2.56E-05	2.825
6	rs118157886	31076084	T	0.3602	0.2188	A	17.74	2.53E-05	2.011
1	rs483130	191380527	T	0.06383	0.01531	C	17.74	2.53E-05	4.386
3	rs75868439	123077062	A	0.08602	0.02573	G	17.74	2.53E-05	3.564
1	rs535553	191395497	C	0.06915	0.01774	T	17.74	2.53E-05	4.114
1	rs61820464	191397295	A	0.06915	0.01774	G	17.74	2.53E-05	4.114
1	rs545082	191399054	C	0.06915	0.01774	T	17.74	2.53E-05	4.114
1	rs524646	191401978	G	0.06915	0.01774	A	17.74	2.53E-05	4.114
5	rs3822426	38476528	T	0.07979	0.0228	A	17.75	2.52E-05	3.715
12	rs2640569	56508821	T	0.08511	0.02542	C	17.78	2.49E-05	3.566
12	rs11171745	56512671	A	0.08511	0.02542	C	17.78	2.49E-05	3.566
17	rs2382843	72516336	G	0.3602	0.2185	A	17.78	2.48E-05	2.013
11	rs73604928	134182970	T	0.1209	0.04444	C	17.78	2.47E-05	2.956
1	rs10923742	119610270	C	0.2074	0.1014	T	17.82	2.42E-05	2.321
1	rs12567198	119610764	A	0.2074	0.1014	G	17.82	2.42E-05	2.321
1	rs12569117	119610849	G	0.2074	0.1014	C	17.82	2.42E-05	2.321
1	rs28606391	119612001	T	0.2074	0.1014	C	17.82	2.42E-05	2.321
1	rs3804553	119618680	G	0.2074	0.1014	T	17.82	2.42E-05	2.321

1	rs12024271	119619654	A	0.2074	0.1014	C	17.82	2.42E-05	2.321
1	rs34851338	119620207	T	0.2074	0.1014	C	17.82	2.42E-05	2.321
1	rs6704201	119621341	T	0.2074	0.1014	C	17.82	2.42E-05	2.321
1	rs34182963	119621792	A	0.2074	0.1014	G	17.82	2.42E-05	2.321
1	rs12741640	119623921	G	0.2074	0.1014	A	17.82	2.42E-05	2.321
1	rs12044562	119627148	G	0.2074	0.1014	T	17.82	2.42E-05	2.321
15	rs4143322	47150907	T	0.2283	0.1154	C	17.83	2.42E-05	2.266
6	rs77734311	142010460	C	0.1374	0.2861	T	17.83	2.42E-05	0.3974
2	rs7592209	235329548	C	0.1374	0.05401	T	17.85	2.39E-05	2.789
6	rs113570031	142014150	C	0.1163	0.2652	T	17.86	2.38E-05	0.3645
1	rs10920357	202151566	A	0.2366	0.1215	G	17.87	2.37E-05	2.24
1	rs2993427	202153583	C	0.2366	0.1215	T	17.87	2.37E-05	2.24
1	rs2924110	202154241	A	0.2366	0.1215	G	17.87	2.37E-05	2.24
6	rs115662870	31045113	T	0.3602	0.2183	C	17.88	2.36E-05	2.016
16	rs35930004	16986849	T	0.2419	0.1256	C	17.89	2.35E-05	2.221
6	rs17054002	142024340	T	0.1374	0.2864	C	17.91	2.32E-05	0.3967
2	rs77647021	206465910	T	0.1576	0.0669	A	17.92	2.30E-05	2.61
21	rs75313264	20783974	A	0.2473	0.1285	G	17.92	2.30E-05	2.228
1	rs12738768	119627954	C	0.2074	0.101	T	17.95	2.26E-05	2.329
2	rs6713097	69401105	T	0.08152	0.02316	A	17.96	2.26E-05	3.744
2	rs1349827	184592205	A	0.4651	0.302	T	17.99	2.22E-05	2.009
17	rs4789076	72546392	T	0.3913	0.2432	C	18	2.21E-05	2
4	rs1517293	156393197	G	0.489	0.3276	A	18.02	2.18E-05	1.965
6	rs139407918	32193434	A	0.2556	0.134	G	18.03	2.17E-05	2.218
4	rs191176756	179434548	T	0.1935	0.09059	C	18.06	2.14E-05	2.409
6	rs143554049	31075718	C	0.3533	0.211	T	18.15	2.05E-05	2.043
6	rs112598507	31235662	T	0.1828	0.08333	C	18.15	2.04E-05	2.461
6	rs115606363	31074128	T	0.3617	0.2189	C	18.19	2.00E-05	2.022
6	rs114218527	31049655	A	0.3617	0.2188	G	18.26	1.92E-05	2.024
6	rs114288990	31051008	A	0.3617	0.2188	G	18.26	1.92E-05	2.024
6	chr6:31051553	31051553	T	0.3617	0.2188	C	18.26	1.92E-05	2.024
6	rs116550881	31055018	A	0.3617	0.2188	G	18.26	1.92E-05	2.024
6	rs115265711	31058178	C	0.3617	0.2188	T	18.26	1.92E-05	2.024
6	rs115430745	31058975	A	0.3617	0.2188	G	18.26	1.92E-05	2.024
6	rs191153530	31061630	C	0.3617	0.2188	T	18.26	1.92E-05	2.024
6	rs114435253	31064710	C	0.3617	0.2188	T	18.26	1.92E-05	2.024
6	rs114247930	31066671	A	0.3617	0.2188	C	18.26	1.92E-05	2.024
6	rs115116245	31066677	T	0.3617	0.2188	G	18.26	1.92E-05	2.024
6	rs145705679	31069749	A	0.3617	0.2188	C	18.26	1.92E-05	2.024
6	rs115361770	31069937	G	0.3617	0.2188	A	18.26	1.92E-05	2.024
6	rs114478090	31070522	A	0.3617	0.2188	G	18.26	1.92E-05	2.024
6	rs115131922	31070524	T	0.3617	0.2188	C	18.26	1.92E-05	2.024
6	rs116029578	31070548	T	0.3617	0.2188	C	18.26	1.92E-05	2.024
6	rs116463898	31070960	T	0.3617	0.2188	A	18.26	1.92E-05	2.024
6	rs114215544	31071453	A	0.3617	0.2188	G	18.26	1.92E-05	2.024
6	rs151255529	31072685	T	0.3617	0.2188	C	18.26	1.92E-05	2.024
6	rs116547042	31073337	G	0.3617	0.2188	T	18.26	1.92E-05	2.024
6	rs150324115	31073369	T	0.3617	0.2188	G	18.26	1.92E-05	2.024
6	rs149333983	31073537	A	0.3617	0.2188	T	18.26	1.92E-05	2.024
6	rs151033586	31073772	A	0.3617	0.2188	G	18.26	1.92E-05	2.024
6	rs114812812	31074148	T	0.3617	0.2188	G	18.26	1.92E-05	2.024
6	rs145381480	31074455	T	0.3617	0.2188	C	18.26	1.92E-05	2.024
6	rs140429023	31076199	T	0.3617	0.2188	A	18.26	1.92E-05	2.024
6	rs151087001	31077046	T	0.3617	0.2188	C	18.26	1.92E-05	2.024
6	rs115299882	31077239	T	0.3617	0.2188	C	18.26	1.92E-05	2.024
6	rs78032704	31078160	G	0.3617	0.2188	A	18.26	1.92E-05	2.024
6	rs3734854	31078836	A	0.3617	0.2188	G	18.26	1.92E-05	2.024
6	rs2233985	31079242	G	0.3617	0.2188	A	18.26	1.92E-05	2.024
6	rs2233984	31079264	T	0.3617	0.2188	C	18.26	1.92E-05	2.024
6	rs2233983	31079371	G	0.3617	0.2188	C	18.26	1.92E-05	2.024
6	rs2233973	31080099	C	0.3617	0.2188	G	18.26	1.92E-05	2.024
6	rs4495304	31080718	C	0.3617	0.2188	T	18.26	1.92E-05	2.024
6	rs115124330	31077405	C	0.3602	0.2169	G	18.27	1.92E-05	2.032
3	rs1982513	59696522	A	0.1705	0.07319	T	18.29	1.90E-05	2.602
12	rs76896409	78589563	T	0.07065	0.0178	C	18.3	1.89E-05	4.196
12	rs77493961	78590190	T	0.07065	0.0178	C	18.3	1.89E-05	4.196
12	rs74705494	78590264	C	0.07065	0.0178	T	18.3	1.89E-05	4.196
15	rs8028738	47151605	C	0.2312	0.1168	T	18.33	1.86E-05	2.275
4	rs2125880	156410338	C	0.5165	0.3514	G	18.35	1.84E-05	1.971

4	rs10013797	156410972	A	0.5056	0.3405	G	18.36	1.83E-05	1.98
6	rs114664593	31052913	A	0.3617	0.2183	G	18.4	1.79E-05	2.029
6	rs114356017	32205922	T	0.2472	0.1263	C	18.43	1.77E-05	2.271
14	rs10431723	76522379	G	0.08696	0.02531	T	18.43	1.76E-05	3.668
6	chr6:31099577	31099577	A	0.2447	0.1267	G	18.45	1.75E-05	2.233
6	rs12525170	31099761	A	0.2447	0.1267	G	18.45	1.75E-05	2.233
16	rs12597308	16969951	C	0.2447	0.1267	T	18.45	1.75E-05	2.233
14	rs7155898	98247762	A	0.1828	0.08262	T	18.47	1.73E-05	2.484
12	rs11610144	118902615	A	0.08602	0.025	T	18.47	1.73E-05	3.671
3	rs148176219	62148552	A	0.04839	0.008532	G	18.48	1.72E-05	5.908
6	rs115285758	31213644	A	0.1862	0.0853	C	18.48	1.71E-05	2.453
6	rs115919076	31391599	T	0.2581	0.1359	C	18.52	1.68E-05	2.212
12	rs75987555	118902616	A	0.08602	0.02496	G	18.53	1.67E-05	3.677
10	rs111970024	16365943	C	0.07979	0.02207	T	18.55	1.66E-05	3.842
12	rs300451	78606896	G	0.07222	0.01804	A	18.56	1.65E-05	4.237
12	rs78916118	78609257	T	0.07222	0.01801	C	18.61	1.60E-05	4.244
1	rs66839660	119643774	G	0.2181	0.1069	A	18.62	1.60E-05	2.33
9	rs10816459	109778256	T	0.3085	0.4772	C	18.62	1.59E-05	0.4888
5	rs3110971	38490271	G	0.08065	0.02226	A	18.63	1.59E-05	3.853
21	rs79763533	20775007	C	0.2778	0.1487	T	18.66	1.57E-05	2.202
17	rs4788845	72536901	A	0.3967	0.2453	G	18.68	1.55E-05	2.023
4	rs4691643	156378568	T	0.4894	0.3274	C	18.7	1.53E-05	1.969
4	rs17008137	156382707	A	0.4894	0.3274	T	18.7	1.53E-05	1.969
17	rs1106351	53324637	A	0.212	0.1016	G	18.7	1.53E-05	2.379
5	rs3110234	38486065	T	0.07979	0.02196	C	18.71	1.52E-05	3.862
5	rs3110235	38486412	T	0.07979	0.02196	G	18.71	1.52E-05	3.862
5	rs3756419	38491403	T	0.07979	0.02196	C	18.71	1.52E-05	3.862
5	rs3099126	38511265	A	0.07979	0.02196	G	18.71	1.52E-05	3.862
5	rs35389603	38513060	G	0.07979	0.02196	A	18.71	1.52E-05	3.862
5	rs2731959	38513561	T	0.07979	0.02196	C	18.71	1.52E-05	3.862
10	rs73589739	16370090	A	0.07979	0.02196	G	18.71	1.52E-05	3.862
7	rs12705815	111818296	C	0.1968	0.09206	T	18.72	1.51E-05	2.417
8	rs76140844	40840177	A	0.4011	0.2478	C	18.74	1.50E-05	2.033
6	rs115379475	32200681	A	0.2444	0.1239	G	18.77	1.48E-05	2.287
17	rs62084827	72549802	T	0.4011	0.2478	C	18.78	1.47E-05	2.033
11	rs11223753	134180874	A	0.1277	0.04762	G	18.81	1.45E-05	2.927
17	rs7215318	72538705	C	0.4032	0.2509	A	18.82	1.44E-05	2.018
6	rs115620472	31217376	T	0.1862	0.0846	C	18.84	1.42E-05	2.475
5	rs6872543	38545260	C	0.07527	0.01956	T	18.86	1.41E-05	4.08
2	rs72937685	206485332	G	0.1613	0.06775	A	18.93	1.35E-05	2.646
21	rs2014457	45669344	T	0.1333	0.04974	C	18.95	1.34E-05	2.939
17	rs2140253	72537894	G	0.4032	0.2504	A	18.96	1.33E-05	2.022
6	rs9390587	148929456	G	0.1087	0.03621	A	18.96	1.33E-05	3.246
6	rs142252318	31102273	A	0.1882	0.08532	T	18.97	1.33E-05	2.485
14	rs4903373	76511798	G	0.1613	0.06759	A	18.98	1.32E-05	2.653
6	rs114547683	31045801	A	0.3587	0.2127	C	18.98	1.32E-05	2.07
6	rs115000746	31045802	T	0.3587	0.2127	C	18.98	1.32E-05	2.07
6	rs137861710	31159139	T	0.1882	0.0853	C	19.02	1.29E-05	2.485
6	rs116041372	31159455	T	0.1882	0.0853	C	19.02	1.29E-05	2.485
6	rs117503829	31161387	T	0.1882	0.0853	C	19.02	1.29E-05	2.485
6	rs146053952	31164664	C	0.1882	0.0853	A	19.02	1.29E-05	2.485
6	rs116758762	31172874	T	0.1882	0.0853	C	19.02	1.29E-05	2.485
6	rs147702490	31175520	T	0.1882	0.0853	C	19.02	1.29E-05	2.485
6	rs114581621	31184418	T	0.1882	0.0853	C	19.02	1.29E-05	2.485
6	rs116529510	31185770	A	0.1882	0.0853	G	19.02	1.29E-05	2.485
6	rs149729882	31187523	T	0.1882	0.0853	G	19.02	1.29E-05	2.485
6	rs117658308	31190737	G	0.1882	0.0853	C	19.02	1.29E-05	2.485
16	rs7191788	16986702	T	0.2473	0.1267	C	19.08	1.26E-05	2.265
6	rs10947137	31094971	C	0.2447	0.125	A	19.11	1.23E-05	2.268
12	rs10747761	56523057	G	0.06452	0.01453	A	19.16	1.20E-05	4.677
17	rs34694042	16845288	G	0.1383	0.05336	A	19.16	1.20E-05	2.847
17	rs783236	72545857	G	0.3956	0.2419	A	19.22	1.16E-05	2.052
6	rs116789740	31382831	T	0.1793	0.07863	C	19.25	1.15E-05	2.561
6	rs117207796	31248736	A	0.1862	0.08376	G	19.28	1.13E-05	2.502
6	rs150150304	31363331	T	0.1793	0.0785	C	19.33	1.10E-05	2.566
1	rs340874	214159256	C	0.2191	0.3903	T	19.36	1.08E-05	0.4383
17	rs11655165	72535321	A	0.3533	0.2071	C	19.37	1.07E-05	2.091
19	rs78159307	6159782	A	0.06522	0.01458	G	19.39	1.07E-05	4.715
19	rs79864873	6160522	T	0.06522	0.01458	G	19.39	1.07E-05	4.715

19	rs58594133	6161060	A	0.06522	0.01458	G	19.39	1.07E-05	4.715
19	rs78713134	6161219	T	0.06522	0.01458	C	19.39	1.07E-05	4.715
1	rs340835	214163675	A	0.2181	0.3843	G	19.42	1.05E-05	0.4469
15	rs1918638	47150142	G	0.234	0.1166	A	19.46	1.03E-05	2.316
17	rs3829584	16838218	T	0.1196	0.04145	C	19.47	1.02E-05	3.141
1	rs55708420	119643546	A	0.2181	0.105	C	19.5	1.01E-05	2.378
1	rs67024035	119640339	A	0.234	0.1162	G	19.55	9.82E-06	2.324
12	rs3809133	56520092	C	0.08511	0.02377	G	19.57	9.72E-06	3.821
21	rs76279832	20782846	A	0.2228	0.1072	G	19.59	9.61E-06	2.388
12	rs11171746	56516156	G	0.08511	0.02373	A	19.62	9.42E-06	3.827
5	rs10071384	38542595	G	0.07979	0.02115	T	19.68	9.18E-06	4.013
5	rs3097247	38491937	C	0.07979	0.02111	T	19.73	8.91E-06	4.02
5	rs10473100	38554731	C	0.07979	0.02111	G	19.73	8.91E-06	4.02
5	rs1899940	38561303	C	0.07979	0.02111	T	19.73	8.91E-06	4.02
2	rs10166207	235322449	A	0.1436	0.05575	G	19.74	8.88E-06	2.841
10	rs911547	105639421	G	0.05851	0.01182	A	19.77	8.75E-06	5.194
6	rs114936582	31034331	T	0.1793	0.07765	C	19.8	8.60E-06	2.596
6	rs115707568	31365050	T	0.2688	0.1404	C	19.8	8.60E-06	2.252
17	rs884802	72517043	C	0.3564	0.2095	T	19.8	8.60E-06	2.09
17	rs2242462	72517824	C	0.3564	0.2095	G	19.8	8.60E-06	2.09
17	rs17553512	72521897	A	0.3564	0.2095	G	19.8	8.60E-06	2.09
17	rs1879968	72525508	T	0.3564	0.2095	C	19.8	8.60E-06	2.09
17	rs4789073	72532378	T	0.3564	0.2095	C	19.8	8.60E-06	2.09
17	rs11657358	72533144	G	0.3564	0.2095	A	19.8	8.60E-06	2.09
17	rs4788844	72534133	A	0.3564	0.2095	C	19.8	8.60E-06	2.09
17	rs11654726	72534875	A	0.3564	0.2095	G	19.8	8.60E-06	2.09
17	rs11654815	72535121	C	0.3564	0.2095	G	19.8	8.60E-06	2.09
2	rs10165529	235321776	A	0.1436	0.05565	G	19.81	8.54E-06	2.846
2	rs6713036	235326552	T	0.1436	0.05565	C	19.81	8.54E-06	2.846
17	rs7214829	53336590	A	0.2181	0.1044	G	19.83	8.47E-06	2.392
17	rs62079899	53337105	T	0.2181	0.1044	G	19.83	8.47E-06	2.392
1	rs12739100	119637891	A	0.234	0.1155	G	19.83	8.47E-06	2.34
13	rs1163623	112221631	C	0.07447	0.01858	T	19.83	8.45E-06	4.25
17	rs73291180	16859249	A	0.1277	0.04615	G	19.88	8.27E-06	3.024
6	rs115696800	31146276	A	0.1882	0.08361	G	19.91	8.10E-06	2.54
12	rs17118317	56520375	C	0.06915	0.01613	T	19.92	8.07E-06	4.531
6	rs117516622	31264415	A	0.1862	0.08248	C	19.94	8.01E-06	2.545
6	rs140561297	31249637	A	0.1429	0.05225	T	19.95	7.95E-06	3.023
6	rs144199269	31249644	A	0.1429	0.05225	G	19.95	7.95E-06	3.023
12	rs76079275	56491740	C	0.06989	0.01624	G	20.05	7.55E-06	4.552
22	rs111826052	19562373	A	0.04255	0.005922	G	20.1	7.36E-06	7.46
22	rs5748305	19563842	T	0.04255	0.005922	C	20.1	7.36E-06	7.46
22	rs5746782	19564442	C	0.04255	0.005922	G	20.1	7.36E-06	7.46
22	rs11703660	19565221	A	0.04255	0.005922	G	20.1	7.36E-06	7.46
22	rs5746785	19565427	C	0.04255	0.005922	T	20.1	7.36E-06	7.46
19	rs118027090	23580864	T	0.07609	0.01884	C	20.1	7.34E-06	4.29
17	rs4627424	53332044	A	0.2181	0.1039	G	20.1	7.33E-06	2.406
6	rs114754524	31040538	A	0.1862	0.0822	C	20.11	7.33E-06	2.554
6	rs117255982	31041453	G	0.1862	0.0822	A	20.11	7.33E-06	2.554
1	rs12724443	119629821	A	0.234	0.1149	G	20.12	7.28E-06	2.355
1	rs28572465	119632815	C	0.234	0.1149	T	20.12	7.28E-06	2.355
1	rs35497565	119637134	G	0.234	0.1149	T	20.12	7.28E-06	2.355
5	rs17232390	14125733	T	0.04787	0.007627	C	20.23	6.88E-06	6.542
5	rs72740380	14139913	C	0.04787	0.007614	T	20.27	6.71E-06	6.553
3	rs77260709	39286999	T	0.157	0.06071	C	20.28	6.68E-06	2.881
5	rs72740385	14146873	T	0.04787	0.007601	C	20.32	6.55E-06	6.564
5	rs17232796	14149763	A	0.04787	0.007601	G	20.32	6.55E-06	6.564
5	rs72740391	14156525	T	0.04787	0.007601	C	20.32	6.55E-06	6.564
5	rs17298767	14157376	T	0.04787	0.007601	C	20.32	6.55E-06	6.564
5	rs7724830	14160447	G	0.04787	0.007601	C	20.32	6.55E-06	6.564
5	rs72740400	14168911	T	0.04839	0.007679	C	20.34	6.49E-06	6.571
17	rs76665464	48936975	A	0.04301	0.005932	G	20.34	6.48E-06	7.531
9	rs10816460	109780666	T	0.6383	0.4611	C	20.38	6.34E-06	2.062
17	rs1699567	72546965	G	0.4149	0.2559	A	20.44	6.14E-06	2.062
6	chr6:32192083	32192083	A	0.2606	0.1334	G	20.49	6.00E-06	2.289
3	rs139874943	62158556	A	0.05319	0.00937	G	20.5	5.97E-06	5.94
2	rs4663408	235330136	A	0.1398	0.05199	G	20.56	5.79E-06	2.963
17	rs11653299	72539555	A	0.4032	0.2448	G	20.57	5.75E-06	2.084
6	rs74375290	31097025	C	0.1862	0.08136	T	20.57	5.74E-06	2.583

6	rs117862827	31140909	G	0.1882	0.0822	T	20.67	5.46E-06	2.588
12	rs12309692	56522945	T	0.06452	0.01361	G	20.67	5.45E-06	5
6	rs117761175	31133982	T	0.1882	0.08206	C	20.75	5.22E-06	2.593
6	rs116196750	31134833	T	0.1882	0.08206	C	20.75	5.22E-06	2.593
13	rs139196431	109321206	A	0.1067	0.03246	C	20.88	4.88E-06	3.562
17	rs9891547	16860257	T	0.1304	0.04593	C	20.9	4.85E-06	3.116
17	rs11486481	16861203	T	0.1304	0.04593	C	20.9	4.85E-06	3.116
11	rs11600362	134196132	T	0.1277	0.04475	C	20.94	4.75E-06	3.124
11	rs11606054	134196311	T	0.1277	0.04475	C	20.94	4.75E-06	3.124
11	rs11606067	134196321	T	0.1277	0.04475	C	20.94	4.75E-06	3.124
11	rs11223756	134196784	T	0.1277	0.04475	C	20.94	4.75E-06	3.124
17	rs1976492	72539845	A	0.4032	0.2436	G	20.97	4.67E-06	2.098
17	rs1566836	72539925	A	0.4032	0.2436	G	20.97	4.67E-06	2.098
17	rs73291188	16864041	G	0.1304	0.04585	A	20.97	4.67E-06	3.122
6	rs146843932	31375482	A	0.2634	0.1339	G	20.97	4.66E-06	2.313
6	rs115201713	31375955	T	0.2634	0.1339	A	20.97	4.66E-06	2.313
6	rs114324866	31376488	C	0.2634	0.1339	T	20.97	4.66E-06	2.313
11	rs68192549	134194345	A	0.1277	0.04467	G	21.01	4.57E-06	3.129
8	rs10089590	40821132	T	0.4043	0.2449	C	21.05	4.46E-06	2.092
17	rs4789077	72546905	G	0.4066	0.2449	T	21.07	4.43E-06	2.113
11	rs7924934	134179321	G	0.133	0.04762	A	21.14	4.26E-06	3.067
6	rs74499212	31108110	T	0.1882	0.08136	C	21.14	4.26E-06	2.617
6	rs115675486	31384479	A	0.266	0.1357	G	21.15	4.26E-06	2.308
6	rs115482356	31386328	A	0.266	0.1357	G	21.15	4.26E-06	2.308
6	rs115684011	31387101	C	0.266	0.1357	T	21.15	4.26E-06	2.308
12	rs78948754	56499715	T	0.06915	0.01528	C	21.21	4.11E-06	4.787
6	rs114291624	31384337	C	0.266	0.1354	T	21.25	4.03E-06	2.313
6	rs115205672	31027569	A	0.1828	0.07751	G	21.32	3.88E-06	2.662
6	rs116297521	31027951	A	0.1828	0.07751	G	21.32	3.88E-06	2.662
12	rs11504023	56505709	T	0.06915	0.01523	C	21.32	3.88E-06	4.804
12	rs78441112	56509229	C	0.06915	0.01523	T	21.32	3.88E-06	4.804
12	rs139404346	56512095	A	0.06915	0.01523	G	21.32	3.88E-06	4.804
12	rs12298136	56516770	G	0.06915	0.01523	C	21.32	3.88E-06	4.804
12	rs79642227	56516978	T	0.06915	0.01523	C	21.32	3.88E-06	4.804
6	rs114790025	31384279	C	0.266	0.1352	T	21.36	3.81E-06	2.317
6	rs116107645	31367636	C	0.2634	0.1329	G	21.38	3.77E-06	2.334
6	rs115804084	31370663	T	0.2634	0.1327	C	21.49	3.57E-06	2.339
5	rs181149712	148917806	C	0.08065	0.0199	A	21.49	3.56E-06	4.321
6	rs112988678	31342208	A	0.2216	0.1007	G	21.55	3.45E-06	2.542
17	rs7215669	72538917	G	0.4032	0.2414	A	21.63	3.30E-06	2.123
6	rs115919349	31378772	T	0.266	0.1345	C	21.68	3.23E-06	2.331
6	rs115009589	31381223	T	0.266	0.1345	C	21.68	3.23E-06	2.331
17	rs113927696	16839712	A	0.1277	0.04374	G	21.79	3.04E-06	3.199
3	rs60193982	39272753	A	0.05978	0.01104	G	21.85	2.96E-06	5.698
6	rs115014250	31368438	A	0.2717	0.137	C	21.9	2.87E-06	2.351
6	rs149837246	31202189	T	0.1512	0.05476	C	21.95	2.79E-06	3.074
6	rs112792404	31346979	C	0.3226	0.1753	T	22.05	2.66E-06	2.24
12	rs12296387	56523354	G	0.06452	0.01278	A	22.09	2.60E-06	5.329
17	rs73289274	16839182	T	0.1237	0.0408	A	22.18	2.49E-06	3.317
22	rs5748306	19565341	T	0.04787	0.006791	C	22.26	2.38E-06	7.353
17	rs11653281	72537245	G	0.3989	0.2365	A	22.32	2.30E-06	2.143
3	rs145093043	190362356	G	0.04255	0.005076	T	22.52	2.08E-06	8.711
3	rs79660256	190364516	G	0.04255	0.005076	A	22.52	2.08E-06	8.711
17	rs1532800	72537179	A	0.3989	0.2356	C	22.6	2.00E-06	2.153
12	rs190516343	56485931	G	0.06989	0.01453	T	22.74	1.86E-06	5.097
1	rs340883	214145706	T	0.2056	0.3904	C	22.9	1.71E-06	0.4041
3	rs150356187	190364357	T	0.04348	0.005093	C	23.05	1.58E-06	8.879
6	rs116918020	31275113	T	0.1793	0.0713	C	23.51	1.25E-06	2.846
6	rs145469430	31109119	A	0.1966	0.08134	G	23.53	1.23E-06	2.764
6	rs189100632	31021266	T	0.1868	0.0756	G	23.62	1.18E-06	2.809
6	rs115904395	31363510	T	0.3085	0.1598	C	24.19	8.72E-07	2.347
6	rs114798610	31016493	T	0.1902	0.07705	C	24.26	8.42E-07	2.814
6	rs115643617	31360934	T	0.3085	0.1586	C	24.68	6.78E-07	2.367
6	rs115335666	31013726	T	0.1902	0.07633	A	24.71	6.66E-07	2.843
2	rs10497642	184828432	C	0.2181	0.09459	T	24.97	5.82E-07	2.67
6	rs143171284	31355557	T	0.2253	0.09737	C	25.23	5.08E-07	2.696
6	rs114108685	31008439	T	0.1944	0.07573	C	26.38	2.80E-07	2.946
6	rs114337491	31346621	C	0.25	0.1068	G	28.88	7.71E-08	2.787
6	rs116799036	31349148	A	0.2394	0.1014	G	29.14	6.74E-08	2.79

6	rs111835655	31347046	A	0.2419	0.1024	G	29.21	6.50E-08	2.797
6	rs116454427	31345484	A	0.2444	0.1025	G	29.31	6.16E-08	2.834
5	rs115338680	171704469	A	0.05319	0.005932	G	29.51	5.56E-08	9.414
6	rs140104413	31361118	C	0.2394	0.1005	T	29.59	5.35E-08	2.816
6	rs115312047	31353019	G	0.2394	0.1005	C	29.72	4.99E-08	2.816
6	rs115515778	31360671	T	0.2394	0.09983	C	30.06	4.19E-08	2.838
6	rs144280416	31361090	A	0.2394	0.09983	G	30.06	4.19E-08	2.838

Blue shows validated SNPs in this study.

ⓔ Intestinal BD (iBD, n = 99) vs. BD without intestinal involvement (sBD, n = 100) within SNPs with *P* value < 0.0001 from comparison between BD (n = 199) and healthy control (healthy CTL, n = 597)

rs number[‡]	Chromosome	Position	Cluster	*P* value		Selected nearby gene[†]
				Intestinal BD vs BD without intestinal involvement	Healthy control vs. intestinal+systemic	
rs1681595	14	23102439		1.88098E-23	8.57067E-27	
rs4500591	13	36081260		1.30274E-12	5.81645E-07	MIR548F5, NBEA
rs2785155	11	35068606		1.28514E-06	1.19974E-06	
rs32019	5	66702373		1.54409E-06	3.95241E-06	CD180
rs7590554	2	40715880		0.000119914	1.66874E-06	SLC8A1
rs1433584	8	130522508		0.00136393	4.86889E-05	LOC728724,GSDMC
rs4674839	2	224879207		0.002426201	9.46697E-05	SERPINE2
rs12049333	1	231314575	1	0.003579872	1.98945E-05	TRIM67
rs2242462	17	72517824	4	0.003935061	8.59114E-05	CD300LB
rs11654726	17	72534875	4	0.004279631	9.61275E-05	
rs7153058	14	47631898		0.005801574	3.73997E-05	MDGA2
rs17178527	6	141906080		0.006442459	8.15344E-05	
rs10497642	2	184828432		0.008842849	3.05418E-05	
rs917365	12	2152171		0.009001497	9.82925E-05	DCP1B,CACNA1C
rs9348841	6	30577873		0.009891566	0.000057493	PPP1R10
rs10816460	9	109780666		0.011686556	5.73717E-05	MIR548Q
rs7245731	19	29975118		0.013565042	2.08074E-05	LOC284395
rs1918638	15	47150142		0.013784378	6.92281E-05	
rs10089590	8	40821132		0.015749247	8.92487E-05	
rs8060190	16	83678303	3	0.023106756	6.5683E-06	CDH13
rs8060711	16	83678513	3	0.023106756	3.62423E-06	CDH13
rs750688	3	159358778		0.023438553	4.03899E-05	IQCJ-SCHIP1,SCHIP1,IQCJ
rs10881048	12	47527599		0.024416929	8.79967E-05	AMIGO2,LOC100233209,FAM113B
rs4796796	17	76614120		0.024512801	8.51488E-05	
rs10271212	7	36814860		0.030244206	9.93291E-05	
rs1357329	1	77409490		0.036712374	8.65218E-05	ST6GALNAC5
rs12048739	1	231316174	1	0.039569753	4.72545E-05	TRIM67
rs9324513	8	140725042		0.040951361	5.82008E-05	
rs375237	16	23462639	2	0.046653144	6.63122E-05	COG7
rs394908	16	23463305	2	0.046653144	5.54729E-05	COG7
rs7204714	16	23483353	2	0.046653144	3.93079E-05	GGA2
rs430208	16	23458485	2	0.048732024	6.71611E-05	COG7

‡:Regions with *P* value <0.05 in intestinal BD vs BD without intestinal involvement among 342 SNPs with *P* value <0.0001 in healthy control vs. intestinal+systemic

Blue shows validated SNPs in this study.

Chromosome locations are based on NCBI 37 Build.

Supporting Information 3: Summary of replication study

					Replication (196 intestinal BD, 138 BD without intestinal involvement, 391 healthy control)									Combined (295 intestinal BD, 238 BD without intestinal involvement, 597 healthy control)											
	Locus				aOR(95%CI)				aP value†				Cochran-Armitage Trend Test	aOR(95%CI)				aP value				Cochran-Mantel-Haenszel test			
Comparison groups	Nearby Gene	SNP	Chr.	Position	Dominant	Recessive	Codominant	Allele	Dominant	Recessive	Codominant	Allele		Dominant	Recessive	Codominant	Allele	Dominant	Recessive	Codominant	Allele	Dominant	Recessive	Codominant	Allele
Intestinal BD vs. control	IL10	rs1554286	1	206944233	0.632(0.447,0.892)	0.493(0.24,1.011)	0.662(0.5,0.877)	0.668(0.507,0.88)	9.E-03	5.E-02	4.E-03	4.E-03	4.E-03	0.687(0.506,0.932)	0.577(0.319,1.044)	0.717(0.562,0.915)	0.722(0.567,0.918)	2.E-02	7.E-02	8.E-03	8.E-03	2.E-02	7.E-02	7.E-03	8.E-03
Intestinal BD vs. control	IL10	rs1518111	1	206944645	0.618(0.435,0.876)	0.577(0.287,1.158)	0.67(0.505,0.89)	0.671(0.507,0.889)	7.E-03	1.E-01	6.E-03	5.E-03	5.E-03	0.665(0.489,0.905)	0.642(0.358,1.153)	0.717(0.561,0.916)	0.717(0.562,0.916)	1.E-02	1.E-01	8.E-03	8.E-03	9.E-03	1.E-01	8.E-03	8.E-03
Intestinal BD vs. control	IL10	rs1800871	1	206946634	0.618(0.437,0.874)	0.594(0.295,1.196)	0.672(0.507,0.891)	0.676(0.512,0.893)	6.E-03	1.E-01	6.E-03	6.E-03	5.E-03	0.667(0.491,0.906)	0.667(0.37,1.204)	0.722(0.564,0.922)	0.724(0.568,0.923)	1.E-02	2.E-01	9.E-03	9.E-03	9.E-03	2.E-01	9.E-03	9.E-03
Intestinal BD vs. control	IL23R-IL12RB2	rs12119179	1	67747415	0.85(0.577,1.251)	1.014(0.665,1.547)	0.939(0.731,1.207)	0.942(0.737,1.204)	4.E-01	9.E-01	6.E-01	6.E-01	6.E-01	0.953(0.673,1.35)	1.196(0.832,1.722)	1.047(0.84,1.305)	1.046(0.842,1.298)	8.E-01	3.E-01	7.E-01	7.E-01	8.E-01	3.E-01	7.E-01	7.E-01
Intestinal BD vs. control	IL23R-IL12RB2	rs1495965	1	67753508	0.85(0.568,1.27)	1.186(0.8,1.76)	1.006(0.784,1.289)	1.005(0.788,1.283)	4.E-01	4.E-01	1.E+00	1.E+00	1.E+00	0.971(0.679,1.389)	1.283(0.901,1.828)	1.089(0.873,1.357)	1.085(0.874,1.346)	9.E-01	2.E-01	4.E-01	5.E-01	9.E-01	2.E-01	5.E-01	5.E-01
Intestinal BD vs. control	NHLH2	rs7556581	1	116386105	1.143(0.802,1.628)	1.258(0.76,2.082)	1.135(0.88,1.464)	1.135(0.88,1.464)	5.E-01	4.E-01	3.E-01	3.E-01	3.E-01	1.15(0.841,1.573)	1.731(1.131,2.649)	1.238(0.994,1.54)	1.251(1,1.565)	4.E-01	1.E-02	6.E-02	5.E-02	4.E-01	1.E-02	6.E-02	5.E-02
Intestinal BD vs. control	TGFBR3	rs17882828	1	92163682	0.967(0.534,1.751)	0(0,∞)	0.941(0.527,1.68)	0.942(0.529,1.676)	9.E-01	1.E+00	8.E-01	8.E-01		0.877(0.511,1.506)	0(0,∞)	0.856(0.504,1.452)	0.858(0.507,1.45)	6.E-01	1.E+00	6.E-01	6.E-01	6.E-01	4.E-01	6.E-01	6.E-01
Intestinal BD vs. control	TGFBR3	rs284148	1	92277843	0.842(0.587,1.209)	1.217(0.785,1.887)	0.982(0.768,1.255)	0.982(0.767,1.256)	4.E-01	4.E-01	9.E-01	9.E-01	9.E-01	0.667(0.486,0.916)	1.05(0.703,1.567)	0.84(0.676,1.043)	0.835(0.67,1.041)	1.E-02	8.E-01	1.E-01	1.E-01	1.E-02	8.E-01	1.E-01	1.E-01
Intestinal BD vs. control	TGFBR3	rs1805110	1	92327045	0.964(0.667,1.392)	1.167(0.765,1.78)	1.035(0.812,1.319)	1.035(0.813,1.317)	8.E-01	5.E-01	8.E-01	8.E-01	8.E-01	0.774(0.561,1.067)	1.063(0.724,1.56)	0.91(0.734,1.127)	0.908(0.731,1.127)	1.E-01	8.E-01	4.E-01	4.E-01	1.E-01	8.E-01	4.E-01	4.E-01
Intestinal BD vs. control	STAT4	rs7574070	2	192010488	1.422(0.958,2.111)	1.039(0.69,1.564)	1.161(0.912,1.478)	1.166(0.913,1.489)	8.E-02	9.E-01	2.E-01	2.E-01	2.E-01	1.37(0.971,1.934)	1.007(0.7,1.449)	1.135(0.916,1.407)	1.138(0.916,1.412)	7.E-02	1.E+00	2.E-01	2.E-01	7.E-02	1.E+00	2.E-01	2.E-01
Intestinal BD vs. control	CCR1-CCR3	rs5742906	3	46306765																					
Intestinal BD vs. control	IQCJ-SCHIP1	rs16830589	3	159365432	1.265(0.882,1.814)	0.833(0.358,1.939)	1.145(0.852,1.54)	1.156(0.851,1.569)	2.E-01	7.E-01	4.E-01	4.E-01	4.E-01	1.579(1.15,2.167)	0.851(0.406,1.783)	1.343(1.032,1.748)	1.353(1.037,1.765)	5.E-03	7.E-01	3.E-02	3.E-02	5.E-03	7.E-01	3.E-02	3.E-02
Intestinal BD vs. control	IQCJ-SCHIP1,SCHIP1	rs16830581	3	159362915	1.196(0.83,1.725)	0.844(0.363,1.965)	1.103(0.818,1.488)	1.112(0.814,1.519)	3.E-01	7.E-01	5.E-01	5.E-01	5.E-01	1.544(1.122,2.126)	0.858(0.41,1.798)	1.321(1.014,1.721)	1.335(1.021,1.748)	8.E-03	7.E-01	4.E-02	4.E-02	8.E-03	7.E-01	4.E-02	3.E-02
Intestinal BD vs. control	NAALADL2	rs3914501	3	174564668	1.022(0.692,1.51)	1.649(1.099,2.474)	1.212(0.944,1.556)	1.203(0.942,1.536)	9.E-01	2.E-02	1.E-01	1.E-01	1.E-01	1.218(0.854,1.737)	1.914(1.338,2.738)	1.378(1.105,1.718)	1.366(1.1,1.696)	3.E-01	4.E-04	4.E-03	5.E-03	3.E-01	3.E-04	4.E-03	5.E-03
Intestinal BD vs. control	NAALADL2	rs16848171	3	175181067	0.934(0.658,1.327)	1.902(0.899,4.025)	1.048(0.784,1.4)	1.048(0.784,1.4)	7.E-01	9.E-02	8.E-01	8.E-01	8.E-01	1.029(0.755,1.403)	2.462(1.279,4.739)	1.17(0.912,1.501)	1.176(0.913,1.516)	9.E-01	7.E-03	2.E-01	2.E-01	9.E-01	6.E-03	2.E-01	2.E-01
Intestinal BD vs. control	NAALADL2	rs9866564	3	175345143	1.164(0.725,1.871)	0.639(0.066,6.179)	1.12(0.719,1.746)	1.122(0.718,1.754)	5.E-01	7.E-01	6.E-01	6.E-01		1.179(0.772,1.802)	0.439(0.046,4.243)	1.123(0.753,1.676)	1.123(0.753,1.675)	4.E-01	5.E-01	6.E-01	6.E-01	4.E-01	5.E-01	6.E-01	6.E-01
Intestinal BD vs. control	NAALADL2	rs183513200	3	175473134																					
Intestinal BD vs. control	YIPF7	rs6838327	4	44626846	0.873(0.593,1.284)	1.39(0.92,2.1)	1.064(0.828,1.368)	1.059(0.832,1.35)	5.E-01	1.E-01	6.E-01	6.E-01	6.E-01	1.075(0.754,1.531)	1.654(1.152,2.376)	1.243(0.995,1.554)	1.227(0.989,1.521)	7.E-01	6.E-03	6.E-02	6.E-02	7.E-01	6.E-03	6.E-02	6.E-02
Intestinal BD vs. control	YIPF7	rs201334946	4	44624550																					
Intestinal BD vs. control	YIPF7	rs192398919	4	44638017																					
Intestinal BD vs. control	YIPF7	rs374634994	4	44653617																					
Intestinal BD vs. control	CD180	rs32019	5	66702373	1.97(1.282,3.027)	1.045(0.711,1.537)	1.28(1.007,1.627)	1.291(1.012,1.647)	2.E-03	8.E-01	4.E-02	4.E-02	4.E-02	1.71(1.187,2.462)	1.134(0.807,1.592)	1.265(1.023,1.563)	1.279(1.03,1.587)	4.E-03	5.E-01	3.E-02	3.E-02	4.E-03	5.E-01	3.E-02	3.E-02
Intestinal BD vs. control	ERAP1	rs17482078	5	96118866	0.853(0.461,1.578)	–	0.914(0.506,1.653)	0.915(0.507,1.65)	6.E-01	1.E+00	8.E-01	8.E-01		1.001(0.593,1.69)	–	1.041(0.625,1.733)	1.04(0.627,1.725)	1.E+00	1.E+00	9.E-01	9.E-01	1.E+00	2.E-01	9.E-01	9.E-01
Intestinal BD vs. control	ERAP1-ERAP2	rs2927615	5	96198202	0.945(0.464,1.923)	–	1.027(0.523,2.016)	1.028(0.52,2.032)	9.E-01	1.E+00	9.E-01	9.E-01		1.261(0.701,2.27)	–	1.306(0.738,2.308)	1.307(0.739,2.313)	4.E-01	1.E+00	4.E-01	4.E-01	4.E-01	2.E-01	4.E-01	4.E-01
Intestinal BD vs. control	HLA-F-AS1–HLA-A, IFITM4P	rs4713242	6	29718220	0.88(0.62,1.249)	0.976(0.607,1.571)	0.933(0.729,1.194)	0.932(0.727,1.195)	5.E-01	9.E-01	6.E-01	6.E-01	6.E-01	0.988(0.722,1.353)	1.141(0.757,1.72)	1.032(0.83,1.281)	1.032(0.829,1.286)	9.E-01	5.E-01	8.E-01	8.E-01	9.E-01	5.E-01	8.E-01	8.E-01
Intestinal BD vs. control	HLA-B, PSORS1C1	rs12525170	6	31099761	1.404(0.947,2.081)	2.037(0.753,5.512)	1.382(0.99,1.928)	1.419(1.004,2.005)	9.E-02	2.E-01	6.E-02	5.E-02	6.E-02	1.314(0.921,1.873)	1.722(0.671,4.419)	1.293(0.954,1.752)	1.318(0.962,1.805)	1.E-01	3.E-01	1.E-01	9.E-02	1.E-01	3.E-01	1.E-01	8.E-02
Intestinal BD vs. control	HLA-B, PSORS1C1	rs121917820	6	31084794																					
Intestinal BD vs. control	HLA-B	rs4959053	6	31099577	1.341(0.904,1.988)	2.005(0.741,5.425)	1.334(0.956,1.862)	1.367(0.966,1.934)	1.E-01	2.E-01	9.E-02	8.E-02	9.E-02	1.273(0.892,1.815)	1.704(0.664,4.372)	1.261(0.931,1.709)	1.285(0.937,1.76)	2.E-01	3.E-01	1.E-01	1.E-01	2.E-01	3.E-01	1.E-01	1.E-01
Intestinal BD vs. control	PSORS1C1, CDSN	rs7742033	6	31085226	1.926(0.12,30.948)		1.926(0.12,30.948)	1.923(0.12,30.831)	6.E-01		6.E-01	6.E-01		1.323(0.082,21.241)		1.323(0.082,21.241)	1.327(0.083,21.266)	8.E-01		8.E-01	8.E-01	8.E-01		8.E-01	8.E-01
Intestinal BD vs. control	Intergenic	rs2655653	6	92037600	1.211(0.813,1.805)	1.23(0.824,1.837)	1.162(0.909,1.484)	1.161(0.909,1.482)	3.E-01	3.E-01	2.E-01	2.E-01	2.E-01	0.99(0.704,1.393)	1.037(0.72,1.494)	1.009(0.813,1.252)	1.009(0.813,1.253)	1.E+00	8.E-01	9.E-01	9.E-01	1.E+00	8.E-01	9.E-01	9.E-01
Intestinal BD vs. control	ELMO1	rs10259514	7	36829705	0.81(0.573,1.144)	0.652(0.339,1.252)	0.812(0.619,1.067)	0.816(0.624,1.068)	2.E-01	2.E-01	1.E-01	1.E-01	1.E-01	0.648(0.476,0.881)	0.645(0.364,1.141)	0.707(0.554,0.901)	0.706(0.554,0.899)	6.E-03	1.E-01	5.E-03	5.E-03	6.E-03	1.E-01	5.E-03	5.E-03
Intestinal BD vs. control	DCAF12	rs10441723	9	34082144	0.898(0.636,1.267)	0.719(0.379,1.361)	0.88(0.672,1.153)	0.885(0.679,1.152)	5.E-01	3.E-01	4.E-01	4.E-01	4.E-01	0.673(0.495,0.914)	0.659(0.371,1.168)	0.725(0.568,0.924)	0.728(0.573,0.926)	1.E-02	2.E-01	9.E-03	1.E-02	1.E-02	2.E-01	9.E-03	1.E-02
Intestinal BD vs. control	DCAF12	rs10758242	9	34146776	0.835(0.59,1.182)	0.702(0.372,1.325)	0.839(0.64,1.1)	0.84(0.642,1.1)	3.E-01	3.E-01	2.E-01	2.E-01	2.E-01	0.628(0.461,0.856)	0.639(0.362,1.129)	0.693(0.543,0.885)	0.689(0.54,0.88)	3.E-03	1.E-01	3.E-03	3.E-03	3.E-03	1.E-01	3.E-03	3.E-03
Intestinal BD vs. control	DCAF12	rs150474359	9	34107356																					
Intestinal BD vs. control	Intergenic	rs7941240	11	43141144	0.69(0.485,0.981)	0.742(0.323,1.706)	0.738(0.547,0.997)	0.745(0.555,1.001)	4.E-02	5.E-01	5.E-02	5.E-02	5.E-02	0.889(0.654,1.209)	0.965(0.488,1.905)	0.917(0.71,1.185)	0.92(0.716,1.183)	5.E-01	9.E-01	5.E-01	5.E-01	5.E-01	9.E-01	5.E-01	5.E-01
Intestinal BD vs. control	KLRC4-KLRK1, KLRK1	rs2255336	12	10532326	0.846(0.592,1.21)	0.646(0.252,1.654)	0.842(0.62,1.145)	0.844(0.622,1.145)	4.E-01	4.E-01	3.E-01	3.E-01	3.E-01	0.838(0.61,1.151)	0.586(0.251,1.368)	0.828(0.63,1.088)	0.83(0.633,1.089)	3.E-01	2.E-01	2.E-01	2.E-01	3.E-01	2.E-01	2.E-01	2.E-01
Intestinal BD vs. control	MIR548F5, NBEA	rs4500591	13	36081260	0.82(0.582,1.155)	0.621(0.316,1.219)	0.813(0.619,1.068)	0.816(0.623,1.068)	3.E-01	2.E-01	1.E-01	1.E-01	1.E-01	0.885(0.653,1.2)	0.673(0.379,1.197)	0.864(0.679,1.098)	0.867(0.684,1.098)	4.E-01	2.E-01	2.E-01	2.E-01	4.E-01	2.E-01	2.E-01	2.E-01
Intestinal BD vs. control	CD300C/CD300LB	rs61730133	17	72539040																					
Intestinal BD vs. control	LOC284395	rs7245731	19	29975118	0.896(0.605,1.326)	–	0.921(0.625,1.357)	0.931(0.649,1.336)	6.E-01	1.E+00	7.E-01	7.E-01		0.92(0.65,1.302)	–	0.938(0.665,1.322)	0.946(0.687,1.302)	6.E-01	1.E+00	7.E-01	7.E-01	6.E-01	2.E-01	7.E-01	7.E-01
Intestinal BD vs. control	PLCB1	rs12624809	20	8822431	1.081(0.766,1.527)	0.596(0.357,0.995)	0.923(0.716,1.189)	1.085(0.713,1.190)	7.E-01	5.E-02	5.E-01	5.E-01	5.E-01	1.403(1.034,1.905)	0.708(0.439,1.142)	1.116(0.890,1.401)	1.22(0.890,1.414)	3.E-02	2.E-01	3.E-01	3.E-01	3.E-02	2.E-01	3.E-01	3.E-01
Intestinal BD vs. control	PLCB1	rs6086632	20	8822931	1.047(0.741,1.479)	0.592(0.354,0.989)	0.905(0.702,1.167)	0.904(0.700,1.168)	8.E-01	5.E-02	4.E-01	4.E-01	4.E-01	1.385(1.019,1.880)	0.703(1.134,2.296)	1.105(0.881,11.387)	1.111(0.880,1.401)	4.E-02	1.E-01	4.E-01	4.E-01	4.E-02	1.E-01	4.E-01	4.E-01
Intestinal BD vs. control	PLCB1	rs6086633	20	8823064	1.062(0.752,1.497)	0.603(0.361,1.007)	0.917(0.711,1.181)	0.915(0.709,1.181)	7.E-01	5.E-02	5.E-01	5.E-01	5.E-01	1.383(1.019, 1.876)	0.714(1.151,2.259)	1.110(0.885,1.391)	1.115(0.884,1.404)	4.E-02	2.E-01	4.E-01	4.E-01	4.E-02	2.E-01	4.E-01	4.E-01
Intestinal BD vs. control	PLCB1	rs6039302	20	8831137	1.067(0.745,1.508)	0.616(0.365,1.041)	0.926(0.716,1.198)	0.926(0.715,1.198)	7.E-01	7.E-02	6.E-01	6.E-01	6.E-01	1.397(1.028,1.898)	0.720(1.171,2.26)	1.121(0.891,1.408)	1.126(0.891,1.422)	3.E-02	2.E-01	3.E-01	3.E-01	3.E-02	2.E-01	3.E-01	3.E-01
Intestinal BD vs. control	PLCB1	rs6086653	20	8838343	1.092(0.773,1.538)	0.549(0.323,0.930)	0.917(0.705,1.183)	0.914(0.705,1.185)	6.E-01	3.E-02	5.E-01	5.E-01	5.E-01	1.372(1.010,1.862)	0.646(1.057,2.534)	1.087(0.865,1.366)	1.092(0.864,1.381)	4.E-02	8.E-02	5.E-01	5.E-01	4.E-02	8.E-02	5.E-01	5.E-01
BD without intestinal involvement vs. control	IL10	rs1554286	1	206944233	0.847(0.588,1.219)	1.064(0.582,1.945)	0.92(0.696,1.216)	0.919(0.695,1.216)	4.E-01	8.E-01	6.E-01	6.E-01	6.E-01	0.755(0.546,1.044)	0.761(0.424,1.367)	0.801(0.621,1.032)	0.802(0.623,1.032)	9.E-02	4.E-01	9.E-02	9.E-02	2.E-01	8.E-01	2.E-01	2.E-01
BD without intestinal involvement vs. control	IL10	rs1518111	1	206944645	0.826(0.566,1.206)	0.984(0.516,1.877)	0.89(0.665,1.193)	0.89(0.664,1.193)	3.E-01	1.E+00	4.E-01	4.E-01	4.E-01	0.758(0.545,1.055)	0.783(0.43,1.426)	0.807(0.623,1.046)	0.807(0.623,1.046)	1.E-01	4.E-01	1.E-01	1.E-01	9.E-02	4.E-01	9.E-02	9.E-02
BD without intestinal involvement vs. control	IL10	rs1800871	1	206946634	0.871(0.602,1.259)	0.967(0.505,1.851)	0.914(0.686,1.218)	0.915(0.688,1.217)	5.E-01	9.E-01	5.E-01	5.E-01	5.E-01	0.771(0.557,1.068)	0.744(0.402,1.376)	0.806(0.622,1.044)	0.81(0.627,1.046)	1.E-01	3.E-01	1.E-01	1.E-01	1.E-01	3.E-01	1.E-01	1.E-01
BD without intestinal involvement vs. control	IL23R-IL12RB2	rs12119179	1	67747415	1.005(0.657,1.537)	1.312(0.853,2.018)	1.109(0.849,1.449)	1.105(0.85,1.436)	1.E+00	2.E-01	4.E-01	5.E-01	4.E-01	1.093(0.749,1.597)	1.369(0.938,2)	1.166(0.922,1.475)	1.159(0.921,1.46)	6.E-01	1.E-01	2.E-01	2.E-01	6.E-01	1.E-01	2.E-01	2.E-01
BD without intestinal involvement vs. control	IL23R-IL12RB2	rs1495965	1	67753508	0.827(0.541,1.264)	0.951(0.616,1.469)	0.911(0.697,1.191)	0.917(0.708,1.187)	4.E-01	8.E-01	5.E-01	5.E-01	5.E-01	1.086(0.738,1.599)	1.276(0.877,1.855)	1.135(0.896,1.438)	1.127(0.896,1.417)	7.E-01	2.E-01	3.E-01	3.E-01	7.E-01	2.E-01	3.E-01	3.E-01
BD without intestinal involvement vs. control	NHLH2	rs7556581	1	116386105	1.194(0.813,1.752)	0.733(0.391,1.374)	1.031(0.776,1.369)	1.029(0.781,1.357)	4.E-01	3.E-01	8.E-01	8.E-01	8.E-01	1.151(0.824,1.608)	1.151(0.708,1.872)	1.115(0.875,1.42)	1.114(0.876,1.416)	4.E-01	6.E-01	4.E-01	4.E-01	4.E-01	6.E-01	4.E-01	4.E-01
BD without intestinal involvement vs. control	TGFBR3	rs17882828	1	92163682	0.776(0.393,1.533)	0(0,∞)	0.762(0.391,1.483)	0.763(0.392,1.482)	5.E-01	1.E+00	4.E-01	4.E-01		0.812(0.45,1.465)	0(0,∞)	0.795(0.446,1.416)	0.796(0.448,1.415)	5.E-01	1.E+00	4.E-01	4.E-01	5.E-01	4.E-01	4.E-01	4.E-01
BD without intestinal involvement vs. control	TGFBR3	rs284148	1	92277843	0.905(0.609,1.346)	1.053(0.644,1.723)	0.97(0.739,1.273)	0.97(0.741,1.27)	6.E-01	8.E-01	8.E-01	8.E-01	8.E-01	1.046(0.735,1.489)	1.25(0.823,1.897)	1.094(0.864,1.386)	1.093(0.865,1.381)	8.E-01	3.E-01	5.E-01	5.E-01	8.E-01	3.E-01	5.E-01	5.E-01
BD without intestinal involvement vs. control	TGFBR3	rs1805110	1	92327045	1.048(0.703,1.563)	1.178(0.75,1.851)	1.077(0.829,1.4)	1.076(0.83,1.394)	8.E-01	5.E-01	6.E-01	6.E-01	6.E-01	1.094(0.766,1.562)	1.197(0.802,1.788)	1.103(0.875,1.392)	1.101(0.875,1.385)	6.E-01	4.E-01	4.E-01	4.E-01	6.E-01	4.E-01	4.E-01	4.E-01
BD without intestinal involvement vs. control	STAT4	rs7574070	2	192010488	1.456(0.947,2.24)	1.201(0.78,1.849)	1.23(0.949,1.593)	1.242(0.953,1.618)	9.E-02	4.E-01	1.E-01	1.E-01	1.E-01	1.337(0.924,1.933)	0.983(0.665,1.454)	1.115(0.886,1.401)	1.118(0.887,1.409)	1.E-01	9.E-01	4.E-01	3.E-01	1.E-01	9.E-01	4.E-01	3.E-01
BD without intestinal involvement vs. control	CCR1-CCR3	rs5742906	3	46306765																					
BD without intestinal involvement vs. control	IQCJ-SCHIP1	rs16830589	3	159365432	1.307(0.884,1.934)	0.261(0.06,1.135)	1.083(0.776,1.51)	1.085(0.775,1.521)	2.E-01	7.E-02	6.E-01	6.E-01	6.E-01	1.329(0.943,1.872)	0.623(0.258,1.506)	1.155(0.867,1.538)	1.161(0.867,1.554)	1.E-01	3.E-01	3.E-01	3.E-01	1.E-01	3.E-01	3.E-01	3.E-01
BD without intestinal involvement vs. control	IQCJ-SCHIP1,SCHIP1	rs16830581	3	159362915	1.309(0.885,1.935)	0.385(0.112,1.321)	1.102(0.793,1.53)	1.107(0.791,1.549)	2.E-01	1.E-01	6.E-01	6.E-01	6.E-01	1.322(0.936,1.866)	0.713(0.307,1.657)	1.162(0.873,1.546)	1.172(0.874,1.571)	1.E-01	4.E-01	3.E-01	3.E-01	1.E-01	4.E-01	3.E-01	3.E-01
BD without intestinal involvement vs. control	NAALADL2	rs183513200	3	175473134																					
BD without intestinal involvement vs. control	NAALADL2	rs3914501	3	174564668	1.238(0.798,1.919)	1.542(0.992,2.398)	1.282(0.972,1.689)	1.258(0.965,1.639)	3.E-01	5.E-02	8.E-02	9.E-02	8.E-02	0.95(0.658,1.371)	1.469(0.991,2.178)	1.122(0.886,1.422)	1.117(0.886,1.409)	8.E-01	6.E-02	3.E-01	3.E-01	8.E-01	5.E-02	3.E-01	3.E-01
BD without intestinal involvement vs. control	NAALADL2	rs16848171	3	175181067	0.865(0.594,1.261)	0.625(0.204,1.913)	0.855(0.613,1.192)	0.863(0.626,1.191)	5.E-01	4.E-01	4.E-01	4.E-01	4.E-01	1.018(0.732,1.416)	0.768(0.308,1.911)	0.986(0.739,1.315)	0.987(0.748,1.303)	9.E-01	6.E-01	9.E-01	9.E-01	9.E-01	6.E-01	9.E-01	9.E-01
BD without intestinal involvement vs. control	NAALADL2	rs9866564	3	175345143	1.615(1.005,2.595)	0.787(0.081,7.618)	1.504(0.966,2.343)	1.505(0.967,2.342)	5.E-02	8.E-01	7.E-02	7.E-02		1.379(0.89,2.137)	0.547(0.057,5.287)	1.299(0.86,1.961)	1.298(0.86,1.958)	2.E-01	6.E-01	2.E-01	2.E-01	1.E-01	6.E-01	2.E-01	2.E-01
BD without intestinal involvement vs. control	YIPF7	rs201334946	4	44624550																					
BD without intestinal involvement vs. control	YIPF7	rs6838327	4	44626846	0.824(0.546,1.245)	1.094(0.688,1.74)	0.948(0.72,1.249)	0.953(0.734,1.238)	4.E-01	7.E-01	7.E-01	7.E-01	7.E-01	0.63(0.442,0.897)	0.846(0.55,1.302)	0.765(0.599,0.976)	0.783(0.621,0.987)	1.E-02	4.E-01	3.E-02	4.E-02	1.E-02	4.E-01	3.E-02	4.E-02
BD without intestinal involvement vs. control	YIPF7	rs192398919	4	44638017																					
BD without intestinal involvement vs. control	YIPF7	rs374634994	4	44653617																					
BD without intestinal involvement vs. control	CD180	rs32019	5	66702373	1.299(0.846,1.997)	0.536(0.331,0.87)	0.903(0.695,1.173)	0.901(0.691,1.173)	2.E-01	1.E-02	4.E-01	4.E-01	4.E-01	1.399(0.958,2.043)	0.658(0.442,0.98)	0.982(0.78,1.236)	0.982(0.779,1.237)	8.E-02	4.E-02	9.E-01	9.E-01	8.E-02	4.E-02	9.E-01	9.E-01
BD without intestinal involvement vs. control	ERAP1	rs17482078	5	96118866	0.44(0.192,1.009)		0.44(0.192,1.009)	0.452(0.199,1.026)	5.E-02		5.E-02	6.E-02		0.669(0.358,1.251)		0.669(0.358,1.251)	0.68(0.368,1.256)	2.E-01		2.E-01	2.E-01	2.E-01		2.E-01	2.E-01
BD without intestinal involvement vs. control	ERAP1-ERAP2	rs2927615	5	96198202	0.297(0.088,0.998)		0.297(0.088,0.998)	0.304(0.091,1.014)	5.E-02		5.E-02	5.E-02		0.468(0.199,1.1)		0.468(0.199,1.1)	0.476(0.204,1.11)	8.E-02		8.E-02	9.E-02	8.E-02		8.E-02	8.E-02
BD without intestinal involvement vs. control	HLA-F-AS1–HLA-A, IFITM4P	rs4713242	6	29718220	0.915(0.629,1.333)	0.726(0.418,1.26)	0.882(0.673,1.155)	0.884(0.676,1.155)	6.E-01	3.E-01	4.E-01	4.E-01	4.E-01	1.104(0.788,1.548)	0.93(0.59,1.468)	1.03(0.813,1.304)	1.029(0.814,1.301)	6.E-01	8.E-01	8.E-01	8.E-01	6.E-01	8.E-01	8.E-01	8.E-01
BD without intestinal involvement vs. control	HLA-B, PSORS1C1	rs121917820	6	31084794																					
BD without intestinal involvement vs. control	HLA-B, PSORS1C1	rs12525170	6	31099761	1.958(1.306,2.934)	1.878(0.641,5.502)	1.756(1.239,2.489)	1.795(1.262,2.552)	1.E-03	3.E-01	2.E-03	1.E-03	1.E-03	2.21(1.546,3.157)	2.137(0.831,5.494)	1.955(1.432,2.668)	1.988(1.458,2.71)	1.E-05	1.E-01	2.E-05	1.E-05	1.E-05	1.E-01	2.E-05	1.E-05
BD without intestinal involvement vs. control	HLA-B	rs4959053	6	31099577	1.732(1.148,2.614)	2.235(0.796,6.27)	1.626(1.147,2.304)	1.679(1.174,2.401)	9.E-03	1.E-01	6.E-03	5.E-03	6.E-03	2.057(1.435,2.949)	2.166(0.842,5.569)	1.85(1.354,2.526)	1.892(1.383,2.589)	9.E-05	1.E-01	1.E-04	7.E-05	8.E-05	1.E-01	8.E-05	6.E-05
BD without intestinal involvement vs. control	PSORS1C1, CDSN	rs7742033	6	31085226	4.773(0.43,53.004)		4.773(0.43,53.004)	4.75(0.429,52.565)	2.E-01		2.E-01	2.E-01		1.648(0.103,26.477)		1.648(0.103,26.477)	1.647(0.103,26.392)	7.E-01		7.E-01	7.E-01	7.E-01		7.E-01	7.E-01

BD without intestinal involvement vs. control	Intergenic	rs2655653	6	92037600	1.251(0.81,1.931)	0.953(0.606,1.499)	1.074(0.821,1.404)	1.071(0.823,1.394)	3.E-01	8.E-01	6.E-01	6.E-01	6.E-01	1.604(1.079,2.385)	1.415(0.975,2.053)	1.359(1.074,1.72)	1.35(1.071,1.702)	2.E-02	7.E-02	1.E-02	1.E-02	2.E-02	7.E-02	1.E-02	1.E-02
BD without intestinal involvement vs. control	ELMO1	rs10259514	7	36829705	0.642(0.439,0.94)	0.58(0.274,1.229)	0.689(0.507,0.935)	0.69(0.51,0.936)	2.E-02	2.E-01	2.E-02	2.E-02	2.E-02	0.719(0.517,1)	0.513(0.263,1.002)	0.724(0.556,0.944)	0.731(0.563,0.948)	5.E-02	5.E-02	2.E-02	2.E-02	5.E-02	5.E-02	2.E-02	2.E-02
BD without intestinal involvement vs. control	DCAF12	rs10441723	9	34082144	0.825(0.572,1.192)	0.955(0.51,1.789)	0.884(0.665,1.175)	0.885(0.667,1.175)	3.E-01	9.E-01	4.E-01	4.E-01	4.E-01	0.821(0.593,1.137)	0.925(0.528,1.618)	0.876(0.681,1.126)	0.877(0.683,1.126)	2.E-01	8.E-01	3.E-01	3.E-01	2.E-01	8.E-01	3.E-01	3.E-01
BD without intestinal involvement vs. control	DCAF12	rs150474359	9	34107356																		1.E-01	4.E-01	1.E-01	1.E-01
BD without intestinal involvement vs. control	DCAF12	rs10758242	9	34146776	0.795(0.544,1.161)	0.845(0.438,1.629)	0.845(0.63,1.132)	0.843(0.628,1.131)	2.E-01	6.E-01	3.E-01	3.E-01	3.E-01	0.839(0.604,1.166)	0.817(0.461,1.449)	0.866(0.672,1.117)	0.866(0.672,1.116)	3.E-01	5.E-01	3.E-01	3.E-01	3.E-01	5.E-01	3.E-01	3.E-01
BD without intestinal involvement vs. control	Intergenic	rs7941240	11	43141144	0.882(0.606,1.283)	0.953(0.413,2.199)	0.91(0.664,1.246)	0.913(0.671,1.243)	5.E-01	9.E-01	6.E-01	6.E-01	6.E-01	0.658(0.47,0.922)	1.229(0.621,2.435)	0.781(0.592,1.03)	0.778(0.589,1.027)	1.E-02	6.E-01	8.E-02	8.E-02	1.E-02	6.E-01	8.E-02	8.E-02
BD without intestinal involvement vs. control	KLRC4-KLRK1, KLRK1	rs2255336	12	10532326	0.765(0.519,1.127)	0.523(0.174,1.571)	0.765(0.547,1.072)	0.767(0.549,1.072)	2.E-01	2.E-01	1.E-01	1.E-01	1.E-01	0.84(0.599,1.179)	0.732(0.313,1.71)	0.85(0.637,1.134)	0.85(0.637,1.134)	3.E-01	5.E-01	3.E-01	3.E-01	3.E-01	5.E-01	3.E-01	3.E-01
BD without intestinal involvement vs. control	MIR548F5, NBEA	rs4500591	13	36081260	0.839(0.58,1.214)	0.924(0.485,1.76)	0.887(0.666,1.181)	0.886(0.665,1.181)	4.E-01	8.E-01	4.E-01	4.E-01	4.E-01	0.863(0.624,1.194)	0.948(0.541,1.663)	0.907(0.706,1.165)	0.906(0.705,1.165)	4.E-01	9.E-01	4.E-01	4.E-01	4.E-01	9.E-01	4.E-01	4.E-01
BD without intestinal involvement vs. control	CD300C/CD300LB	rs61730133	17	72539040																					
BD without intestinal involvement vs. control	LOC284395	rs7245731	19	29975118	0.69(0.443,1.074)	–	0.78(0.512,1.19)	0.798(0.534,1.194)	1.E-01	1.E+00	2.E-01	3.E-01		0.915(0.632,1.324)	–	1.059(0.751,1.491)	1.055(0.757,1.47)	6.E-01	1.E+00	7.E-01	8.E-01	6.E-01	–	7.E-01	8.E-01
BD without intestinal involvement vs. control	PLCB1	rs12624809	20	8822431	1.355(0.938,1.957)	1.337(0.679,2.632)	1.274(0.951,1.706)	1.262(0.949,1.678)	1.E-01	4.E-01	1.E-01	1.E-01	1.E-01	1.318(0.952,11.825)	1.152(0.653,2.033)	1.213(0.943,1.565)	1.208(0.9471,1.550)	1.E-01	6.E-01	1.E-01	1.E-01	1.E-01	6.E-01	1.E-01	1.E-01
BD without intestinal involvement vs. control	PLCB1	rs6086632	20	8822931	1.126(0.770,1.647)	1.210(0.614,2.387)	1.116(0.829,1.502)	1.110(0.831,1.481)	5.E-01	6.E-01	5.E-01	5.E-01	5.E-01	1.195(0.858,1.664)	1.082(0.613,1.908)	1.30(0.874,1.460)	1.125(0.875,1.447)	3.E-01	8.E-01	4.E-01	4.E-01	3.E-01	8.E-01	4.E-01	4.E-01
BD without intestinal involvement vs. control	PLCB1	rs6086633	20	8823064	1.305(0.904,1.883)	1.477(0.735,2.967)	1.267(0.947,1.698)	1.253(0.943,1.667)	2.E-01	3.E-01	1.E-01	1.E-01	1.E-01	1.295(0.936,1.792)	1.218(0.684,2.169)	1.215(0.943,1.567)	1.208(0.941,1.550)	1.E-01	5.E-01	1.E-01	1.E-01	1.E-01	5.E-01	1.E-01	1.E-01
BD without intestinal involvement vs. control	PLCB1	rs6039302	20	8831137	1.232(0.848,1.786)	1.245(0.631,2.457)	1.185(0.883,1.590)	1.176(0.883,1.567)	3.E-01	5.E-01	3.E-01	3.E-01	3.E-01	1.258(0.907,1.745)	1.092(0.618,1.931)	1.168(0.906,1.508)	1.163(0.905,1.495)	2.E-01	8.E-01	2.E-01	2.E-01	2.E-01	8.E-01	2.E-01	2.E-01
BD without intestinal involvement vs. control	PLCB1	rs6086653	20	8838343	1.314(0.909,1.901)	1.429(0.688,2.967)	1.267(0.940,1.706)	1.253(0.938,1.678)	1.E-01	3.E-01	1.E-01	1.E-01	1.E-01	1.319(0.953,1.828)	1.383(0.737,1.597)	1.2630.973,1.639)	1.252(0.969,1.616)	9.E-02	3.E-01	8.E-02	9.E-02	9.E-02	3.E-01	8.E-02	9.E-02
Intestinal BD vs. BD without intestinal involvement	IL10	rs1554286	1	206944233	0.746(0.492,1.13)	0.463(0.206,1.041)	0.73(0.525,1.014)	0.726(0.522,1.01)	2.E-01	6.E-02	6.E-02	6.E-02	6.E-02	0.909(0.644,1.284)	0.758(0.381,1.505)	0.899(0.681,1.186)	0.899(0.683,1.185)	6.E-01	4.E-01	5.E-01	5.E-01	6.E-01	4.E-01	5.E-01	5.E-01
Intestinal BD vs. BD without intestinal involvement	IL10	rs1518111	1	206944645	0.748(0.486,1.151)	0.586(0.258,1.331)	0.761(0.542,1.07)	0.755(0.535,1.065)	2.E-01	2.E-01	1.E-01	1.E-01	1.E-01	0.877(0.617,1.249)	0.82(0.412,1.631)	0.89(0.672,1.179)	0.889(0.669,1.18)	5.E-01	6.E-01	4.E-01	4.E-01	5.E-01	6.E-01	4.E-01	4.E-01
Intestinal BD vs. BD without intestinal involvement	IL10	rs1800871	1	206946634	0.71(0.466,1.08)	0.613(0.27,1.391)	0.741(0.53,1.036)	0.739(0.528,1.033)	1.E-01	2.E-01	8.E-02	8.E-02	8.E-02	0.865(0.611,1.223)	0.897(0.447,1.8)	0.894(0.676,1.182)	0.894(0.676,1.182)	4.E-01	8.E-01	4.E-01	4.E-01	4.E-01	8.E-01	4.E-01	4.E-01
Intestinal BD vs. BD without intestinal involvement	IL23R-IL12RB2	rs12119179	1	67747415	0.845(0.525,1.361)	0.773(0.471,1.27)	0.854(0.635,1.15)	0.852(0.632,1.15)	5.E-01	3.E-01	3.E-01	3.E-01	3.E-01	0.872(0.583,1.303)	0.874(0.587,1.301)	0.903(0.708,1.153)	0.902(0.705,1.154)	5.E-01	5.E-01	4.E-01	4.E-01	5.E-01	5.E-01	4.E-01	4.E-01
Intestinal BD vs. BD without intestinal involvement	IL23R-IL12RB2	rs1495965	1	67753508	1.027(0.637,1.656)	1.247(0.768,2.025)	1.096(0.817,1.47)	1.097(0.817,1.472)	9.E-01	4.E-01	5.E-01	5.E-01	5.E-01	0.894(0.593,1.347)	1.006(0.681,1.485)	0.963(0.754,1.229)	0.963(0.754,1.229)	6.E-01	1.E+00	8.E-01	8.E-01	6.E-01	1.E+00	8.E-01	8.E-01
Intestinal BD vs. BD without intestinal involvement	NHLH2	rs7556581	1	116386105	0.957(0.618,1.483)	1.716(0.869,3.388)	1.11(0.804,1.532)	1.103(0.807,1.507)	8.E-01	1.E-01	5.E-01	5.E-01	5.E-01	0.999(0.698,1.43)	1.504(0.93,2.434)	1.116(0.872,1.428)	1.124(0.872,1.449)	1.E+00	1.E-01	4.E-01	4.E-01	1.E+00	1.E-01	4.E-01	4.E-01
Intestinal BD vs. BD without intestinal involvement	TGFBR3	rs17882828	1	92163682	1.246(0.582,2.668)		1.246(0.582,2.668)	1.234(0.586,2.601)	6.E-01		6.E-01	6.E-01		1.08(0.571,2.042)		1.08(0.571,2.042)	1.077(0.577,2.009)	8.E-01		8.E-01	8.E-01	8.E-01		8.E-01	8.E-01
Intestinal BD vs. BD without intestinal involvement	TGFBR3	rs284148	1	92277843	0.931(0.596,1.453)	1.156(0.67,1.992)	1.011(0.753,1.359)	1.012(0.746,1.373)	8.E-01	6.E-01	9.E-01	9.E-01	9.E-01	0.638(0.442,0.921)	0.84(0.539,1.309)	0.781(0.613,0.994)	0.764(0.594,0.982)	2.E-02	4.E-01	4.E-02	4.E-02	2.E-02	4.E-01	4.E-02	4.E-02
Intestinal BD vs. BD without intestinal involvement	TGFBR3	rs1805110	1	92327045	0.92(0.587,1.441)	0.991(0.599,1.638)	0.963(0.721,1.285)	0.962(0.718,1.288)	7.E-01	1.E+00	8.E-01	8.E-01	8.E-01	0.707(0.488,1.023)	0.888(0.582,1.355)	0.832(0.655,1.056)	0.825(0.646,1.053)	7.E-02	6.E-01	1.E-01	1.E-01	7.E-02	6.E-01	1.E-01	1.E-01
Intestinal BD vs. BD without intestinal involvement	STAT4	rs7574070	2	192010488	0.977(0.593,1.609)	0.865(0.529,1.414)	0.937(0.691,1.272)	0.939(0.695,1.268)	9.E-01	6.E-01	7.E-01	7.E-01	7.E-01	1.025(0.685,1.535)	1.024(0.675,1.554)	1.019(0.791,1.312)	1.018(0.795,1.303)	9.E-01	9.E-01	9.E-01	9.E-01	9.E-01	9.E-01	9.E-01	9.E-01
Intestinal BD vs. BD without intestinal involvement	CCR1-CCR3	rs5742906	3	46306765																					
Intestinal BD vs. BD without intestinal involvement	IQCJ-SCHIP1	rs16830589	3	159365432	0.968(0.624,1.5)	3.191(0.668,15.252)	1.07(0.725,1.579)	1.065(0.731,1.55)	9.E-01	1.E-01	7.E-01	7.E-01	7.E-01	1.188(0.832,1.696)	1.366(0.529,3.529)	1.178(0.864,1.606)	1.165(0.864,1.572)	3.E-01	5.E-01	3.E-01	3.E-01	3.E-01	5.E-01	3.E-01	3.E-01
Intestinal BD vs. BD without intestinal involvement	IQCJ-SCHIP1,SCHIP1	rs16830581	3	159362915	0.914(0.588,1.421)	2.189(0.571,8.393)	1.005(0.683,1.478)	1.005(0.688,1.467)	7.E-01	3.E-01	1.E+00	1.E+00	1.E+00	1.168(0.817,1.672)	1.203(0.483,2.996)	1.146(0.842,1.56)	1.139(0.843,1.541)	4.E-01	7.E-01	4.E-01	4.E-01	4.E-01	7.E-01	4.E-01	4.E-01
Intestinal BD vs. BD without intestinal involvement	NAALADL2	rs183513200	3	175473134																					
Intestinal BD vs. BD without intestinal involvement	NAALADL2	rs3914501	3	174564668	0.826(0.504,1.354)	1.069(0.662,1.727)	0.958(0.713,1.287)	0.956(0.708,1.291)	4.E-01	8.E-01	8.E-01	8.E-01	8.E-01	1.282(0.859,1.914)	1.303(0.88,1.928)	1.207(0.95,1.535)	1.222(0.955,1.565)	2.E-01	2.E-01	1.E-01	1.E-01	2.E-01	2.E-01	1.E-01	1.E-01
Intestinal BD vs. BD without intestinal involvement	NAALADL2	rs16848171	3	175181067	1.08(0.704,1.656)	3.043(0.982,9.433)	1.208(0.845,1.725)	1.213(0.846,1.739)	7.E-01	5.E-02	3.E-01	3.E-01	3.E-01	1.011(0.712,1.435)	3.208(1.367,7.529)	1.183(0.891,1.571)	1.192(0.892,1.591)	1.E+00	7.E-03	2.E-01	2.E-01	1.E+00	5.E-03	2.E-01	2.E-01
Intestinal BD vs. BD without intestinal involvement	NAALADL2	rs9866564	3	175345143	0.721(0.423,1.229)	0.811(0.05,13.06)	0.737(0.442,1.227)	0.746(0.453,1.229)	2.E-01	9.E-01	2.E-01	2.E-01		0.855(0.543,1.347)	0.801(0.05,12.879)	0.859(0.554,1.333)	0.865(0.564,1.328)	5.E-01	9.E-01	5.E-01	5.E-01	5.E-01	9.E-01	5.E-01	5.E-01
Intestinal BD vs. BD without intestinal involvement	YIPF7	rs201334946	4	44624550																					
Intestinal BD vs. BD without intestinal involvement	YIPF7	rs6838327	4	44626846	1.058(0.666,1.681)	1.27(0.766,2.106)	1.11(0.827,1.491)	1.112(0.827,1.494)	8.E-01	4.E-01	5.E-01	5.E-01	5.E-01	1.707(1.165,2.5)	1.955(1.268,3.012)	1.561(1.218,2)	1.567(1.225,2.005)	6.E-03	2.E-03	4.E-04	4.E-04	6.E-03	2.E-03	4.E-04	3.E-04
Intestinal BD vs. BD without intestinal involvement	YIPF7	rs192398919	4	44638017																					
Intestinal BD vs. BD without intestinal involvement	YIPF7	rs374634994	4	44653617																					
Intestinal BD vs. BD without intestinal involvement	CD180	rs32019	5	66702373	1.516(0.899,2.557)	1.948(1.146,3.312)	1.532(1.102,2.131)	1.433(1.062,1.933)	1.E-01	1.E-02	1.E-02	2.E-02	1.E-02	1.222(0.799,1.87)	1.723(1.137,2.61)	1.339(1.033,1.737)	1.302(1.019,1.665)	4.E-01	1.E-02	3.E-02	4.E-02	4.E-01	1.E-02	3.E-02	4.E-02
Intestinal BD vs. BD without intestinal involvement	ERAP1	rs17482078	5	96118866	1.939(0.778,4.833)	–	1.966(0.815,4.742)	2.024(0.829,4.941)	2.E-01	1.E+00	1.E-01	1.E-01		1.495(0.776,2.882)	–	1.526(0.806,2.891)	1.529(0.807,2.897)	2.E-01	1.E+00	2.E-01	2.E-01	2.E-01	4.E-01	2.E-01	2.E-01
Intestinal BD vs. BD without intestinal involvement	ERAP1-ERAP2	rs2927615	5	96198202	3.183(0.882,11.487)	–	3.118(0.898,10.821)	3.383(0.955,11.979)	8.E-02	1.E+00	7.E-02	6.E-02		2.695(1.135,6.399)	–	2.683(1.149,6.265)	2.744(1.172,6.427)	2.E-02	1.E+00	2.E-02	2.E-02	2.E-02	4.E-01	2.E-02	2.E-02
Intestinal BD vs. BD without intestinal involvement	HLA-F-AS1–HLA-A, IFITM4P	rs4713242	6	29718220	0.962(0.63,1.468)	1.345(0.727,2.491)	1.055(0.778,1.43)	1.055(0.778,1.429)	9.E-01	3.E-01	7.E-01	7.E-01	7.E-01	0.895(0.625,1.28)	1.227(0.763,1.971)	1.003(0.783,1.285)	1.003(0.783,1.286)	5.E-01	4.E-01	1.E+00	1.E+00	5.E-01	4.E-01	1.E+00	1.E+00
Intestinal BD vs. BD without intestinal involvement	HLA-B, PSORS1C1	rs121917820	6	31084794																					
Intestinal BD vs. BD without intestinal involvement	HLA-B, PSORS1C1	rs12525170	6	31099761	0.717(0.457,1.126)	1.085(0.368,3.193)	0.801(0.549,1.167)	0.791(0.537,1.164)	1.E-01	9.E-01	2.E-01	2.E-01	2.E-01	0.595(0.41,0.862)	0.806(0.33,1.97)	0.673(0.492,0.922)	0.663(0.482,0.912)	6.E-03	6.E-01	1.E-02	1.E-02	6.E-03	6.E-01	1.E-02	1.E-02
Intestinal BD vs. BD without intestinal involvement	HLA-B	rs4959053	6	31099577	0.774(0.49,1.223)	0.897(0.318,2.529)	0.829(0.569,1.208)	0.814(0.55,1.207)	3.E-01	8.E-01	3.E-01	3.E-01	3.E-01	0.619(0.425,0.9)	0.786(0.322,1.923)	0.693(0.505,0.949)	0.679(0.492,0.937)	1.E-02	6.E-01	2.E-02	2.E-02	1.E-02	6.E-01	2.E-02	2.E-02
Intestinal BD vs. BD without intestinal involvement	PSORS1C1, CDSN	rs7742033	6	31085226	0.403(0.036,4.489)		0.403(0.036,4.489)	0.405(0.037,4.486)	5.E-01		5.E-01	5.E-01		0.801(0.05,12.879)		0.801(0.05,12.879)	0.802(0.05,12.85)	9.E-01		9.E-01	9.E-01	9.E-01		9.E-01	9.E-01
Intestinal BD vs. BD without intestinal involvement	Intergenic	rs2655653	6	92037600	0.968(0.588,1.595)	1.291(0.781,2.133)	1.088(0.8,1.48)	1.084(0.803,1.463)	9.E-01	3.E-01	6.E-01	6.E-01	6.E-01	0.617(0.406,0.938)	0.733(0.492,1.091)	0.744(0.579,0.955)	0.747(0.584,0.957)	2.E-02	1.E-01	2.E-02	2.E-02	2.E-02	1.E-01	2.E-02	2.E-02
Intestinal BD vs. BD without intestinal involvement	ELMO1	rs10259514	7	36829705	1.261(0.82,1.939)	1.123(0.467,2.703)	1.187(0.836,1.686)	1.182(0.837,1.67)	3.E-01	8.E-01	3.E-01	3.E-01	3.E-01	0.901(0.633,1.281)	1.256(0.596,2.645)	0.966(0.725,1.287)	0.966(0.726,1.285)	6.E-01	5.E-01	8.E-01	8.E-01	6.E-01	5.E-01	8.E-01	8.E-01
Intestinal BD vs. BD without intestinal involvement	DCAF12	rs10441723	9	34082144	1.088(0.717,1.652)	0.753(0.352,1.61)	0.999(0.72,1.387)	0.999(0.723,1.381)	7.E-01	5.E-01	1.E+00	1.E+00	1.E+00	0.819(0.579,1.159)	0.712(0.373,1.359)	0.832(0.633,1.093)	0.83(0.631,1.092)	3.E-01	3.E-01	2.E-01	2.E-01	3.E-01	3.E-01	2.E-01	2.E-01
Intestinal BD vs. BD without intestinal involvement	DCAF12	rs150474359	9	34107356																					
Intestinal BD vs. BD without intestinal involvement	DCAF12	rs10758242	9	34146776	1.051(0.683,1.616)	0.831(0.378,1.826)	0.996(0.711,1.397)	0.996(0.711,1.397)	8.E-01	6.E-01	1.E+00	1.E+00	1.E+00	0.748(0.525,1.066)	0.782(0.403,1.515)	0.801(0.606,1.057)	0.796(0.601,1.054)	1.E-01	5.E-01	1.E-01	1.E-01	1.E-01	5.E-01	1.E-01	1.E-01
Intestinal BD vs. BD without intestinal involvement	Intergenic	rs7941240	11	43141144	0.783(0.509,1.204)	0.779(0.286,2.124)	0.815(0.567,1.17)	0.816(0.569,1.169)	3.E-01	6.E-01	3.E-01	3.E-01	3.E-01	1.35(0.944,1.932)	0.785(0.375,1.641)	1.173(0.878,1.569)	1.182(0.879,1.59)	1.E-01	5.E-01	3.E-01	3.E-01	1.E-01	5.E-01	3.E-01	3.E-01
Intestinal BD vs. BD without intestinal involvement	KLRC4-KLRK1, KLRK1	rs2255336	12	10532326	1.106(0.711,1.72)	1.234(0.342,4.451)	1.103(0.747,1.629)	1.099(0.75,1.613)	7.E-01	7.E-01	6.E-01	6.E-01	6.E-01	0.998(0.694,1.435)	0.801(0.296,2.169)	0.976(0.711,1.34)	0.977(0.715,1.335)	1.E+00	7.E-01	9.E-01	9.E-01	1.E+00	7.E-01	9.E-01	9.E-01
Intestinal BD vs. BD without intestinal involvement	MIR548F5,NBEA	rs4500591	13	36081260	0.977(0.643,1.484)	0.672(0.302,1.497)	0.92(0.659,1.282)	0.92(0.661,1.281)	9.E-01	3.E-01	6.E-01	6.E-01	6.E-01	1.026(0.726,1.449)	0.71(0.372,1.356)	0.956(0.728,1.256)	0.957(0.73,1.254)	9.E-01	3.E-01	7.E-01	7.E-01	9.E-01	3.E-01	7.E-01	7.E-01
Intestinal BD vs. BD without intestinal involvement	CD300C/CD300LB	rs61730133	17	72539040																		7.E-01	2.E-01	3.E-01	4.E-01
Intestinal BD vs. BD without intestinal involvement	LOC284395	rs7245731	19	29975118	1.298(0.788,2.139)	0.271(0.028,2.634)	1.172(0.738,1.861)	1.167(0.74,1.84)	3.E-01	3.E-01	5.E-01	5.E-01		1.006(0.677,1.494)	0.113(0.014,0.925)	0.895(0.624,1.283)	0.897(0.628,1.281)	1.E+00	4.E-02	5.E-01	5.E-01	1.E+00	1.E-02	5.E-01	5.E-01
Intestinal BD vs. BD without intestinal involvement	PLCB1	rs12624809	20	8822431	0.798(0.525,1.212	0.446(0.220,0.902)	0.746(0.547,1.018)	0.730(0.530,11.006)	3.E-01	2.E-02	6.E-02	5.E-02	6.E-02	1.065(0.754,1.504)	0.615(0.347,1.089)	0.935(0.726,0.206)	0.929(0.711,1.214)	7.E-01	1.E-01	6.E-01	6.E-01	7.E-01	9.E-02	6.E-01	6.E-01
Intestinal BD vs. BD without intestinal involvement	PLCB1	rs6086632	20	8822931	0.929(0.605,11.429)	0.489(0.241,0.992)	0.824(0.601,1.129)	0.815(0.590,1.126)	7.E-01	5.E-02	2.E-01	2.E-01	2.E-01	1.159(0.816,1.647)	0.649(0.366,1.151)	0.988(0.764,1.277)	0.980.754,1.292)	4.E-01	1.E-01	9.E-01	9.E-01	4.E-01	1.E-01	9.E-01	9.E-01
Intestinal BD vs. BD without intestinal involvement	PLCB1	rs6086633	20	8823064	0.813(0.536,1.233)	0.408(0.198,0.842)	0.742(0.543,1.013)	0.730(0.531,1.004)	3.E-01	2.E-02	6.E-02	5.E-02	6.E-02	1.068(0.7561.508)	0.586(0.328,1.047)	0.929(0.720,1.199)	0.923(0.707,1.266)	7.E-01	7.E-02	6.E-01	6.E-01	7.E-01	7.E-02	6.E-01	6.E-01
Intestinal BD vs. BD without intestinal involvement	PLCB1	rs6039302	20	8831137	0.867(0.568,1.3252)	0.495(0.243,1.008)	0.797(0.582,1.093)	0.787(0.570,1.087)	5.E-01	5.E-02	2.E-01	1.E-01	2.E-01	1.110(0.783,1.572)	0.659(0.370,1.174)	0.997(0.751,1.256)	0.968(0.739,1.267)	6.E-01	2.E-01	8.E-01	8.E-01	6.E-01	2.E-01	8.E-01	8.E-01
Intestinal BD vs. BD without intestinal involvement	PLCB1	rs6086653	20	8838343	0.830(0.546,1.261)	0.384(0.181,0.814)	0.747(0.545,1.022)	0.729(0.527,1.009)	4.E-01	1.E-02	7.E-02	6.E-02	7.E-02	1.038(0.734,1.468)	0.467(0.250,0.874)	0.884(0.682,1.147)	0.873(0.665,1.147)	8.E-01	2.E-02	4.E-01	3.E-01	8.E-01	2.E-02	4.E-01	3.E-01

†a*P* value: *P* value from logistic regression analysis adjusted for sex and age.

Red and yellow indicate $P < 0.05$ and $P < 0.00113$ (0.05/44 SNPs), respectively.

Supporting Information 4: eQTL analysis, RegulomeDB analysis, and HaploReg analysis

eQTL analysis

Nearby Gene[¥]	SNPs	Chr.	Position	LD-proxy of rSNP (r2>0.8)	Study	Tissue type	Cis-eQTL *P*[¶]	No. of cis-probes[*]	corrected Cis-eQTL *P* [†]	Cis-eQTL probe	Cis-eQTL gene
DCAF12	rs10441723	9	34082144	yes	Geneva GenCord	GenCord-F	4.56E-02	53	2.42E+00	ILMN_1804610	NFX1
					Geneva GenCord	GenCord-F	1.29E-02	53	6.84E-01	ILMN_2349444	NUDT2
					Geneva GenCord	GenCord-F	3.43E-02	53	1.82E+00	ILMN_1778347	NUDT2
					Geneva GenCord	GenCord-F	4.10E-03	53	2.17E-01	ILMN_1794056	DNAJB5
					Geneva GenCord	GenCord-F	3.20E-03	53	1.70E-01	ILMN_1663722	DNAJB5
					Geneva GenCord	GenCord-F	1.43E-02	53	7.58E-01	ILMN_2398489	OPRS1
					Geneva GenCord	GenCord-F	4.88E-02	53	2.59E+00	ILMN_1809456	CNTFR
					Geneva GenCord	GenCord-F	3.48E-02	53	1.84E+00	ILMN_2411791	CNTFR
					Geneva GenCord	GenCord-F	2.78E-02	53	1.47E+00	ILMN_1665442	NOL6
					Geneva GenCord	GenCord-L	1.15E-02	53	6.10E-01	ILMN_1651574	AQP3
					MuTHER Pilot	Twin1-A	4.81E-02	57	2.74E+00	ILMN_1778347	NUDT2
					MuTHER Pilot	Twin2-S	3.82E-02	57	2.18E+00	ILMN_1706005	GALT
					MuTHER Pilot	Twin2-L	3.89E-02	57	2.22E+00	ILMN_1720024	IL11RA
					MuTHER Pilot	Twin2-A	3.00E-04	57	1.71E-02	ILMN_1705735	C9orf131
					MuTHER Pilot	Twin2-S	2.91E-02	57	1.66E+00	ILMN_1717925	SIGMAR1
					MuTHER Pilot	Twin1-S	4.10E-02	57	2.34E-01	ILMN_2391345	C9orf23
					MuTHER Pilot	Twin2-A	4.31E-02	57	2.46E+00	ILMN_1651574	AQP3
					Geneva GenCord	GenCord-L	5.90E-03				IL10
					Geneva GenCord	GenCord-F	2.68E-02				CCR1
					Geneva GenCord	GenCord-F	5.00E-03				CCR3
	rs10758242	9	34146776	yes	HapMap3 (JPT)	Lymphoblastoid cell line	8.00E-04	53	4.24E-02	ILMN_1778347	NUDT2
					Geneva GenCord	GenCord-L	2.53E-02			ILMN_1752145	ERAP1
ELMO1	rs10259514	7	36829705	yes	MuTHER Pilot	Twin2-A	1.01E-02	11	1.11E-01	ILMN_1739645	ANLN
					MuTHER Pilot	Twin2-L	3.49E-02	11	3.84E-01	ILMN_1709820	AOAH
					MuTHER Pilot	Twin1-L	7.30E-03	11	8.03E-02	ILMN_1690484	KIAA0895
					Geneva GenCord	GenCord-L	5.90E-03			ILMN_1674167	IL10
					HapMap3 (CHB)	Lymphoblastoid cell line	3.90E-03			ILMN_1722991	KLRC4
ERAP1	rs17482078	5	96118866	yes	HapMap3 (JPT)	Lymphoblastoid cell line	4.96E-02	18	8.93E-01	ILMN_1768585	CAST
					HapMap3 (JPT)	Lymphoblastoid cell line	2.80E-03	18	5.04E-02	ILMN_1720934	LNPEP
					MuTHER Pilot	Twin1-A	1.13E-02	20	2.26E-01	ILMN_1744949	RHOBTB3
					MuTHER Pilot	Twin2-L	1.33E-02	20	2.66E-01	ILMN_2322806	CAST
					MuTHER Pilot	Twin1-A	1.60E-05	20	3.20E-04	ILMN_2336220	ERAP1
					MuTHER Pilot	Twin1-L	4.90E-06	20	9.80E-05	ILMN_2336220	ERAP1
					HapMap3 (CHB)	Lymphoblastoid cell line	1.76E-02			ILMN_1785202	STAT4
					MuTHER Pilot	Twin2-A	3.20E-07	20	6.40E-06	ILMN_2336220	ERAP1
					MuTHER Pilot	Twin2-L	4.50E-09	20	9.00E-08	ILMN_2336220	ERAP1
	rs2927615	5	96198202	yes	Geneva GenCord	GenCord-F	2.29E-02	14	3.21E-01	ILMN_1783627	CAST
					Geneva GenCord	GenCord-F	2.00E-04	14	2.80E-03	ILMN_2322806	CAST
					Geneva GenCord	GenCord-L	4.90E-07	14	6.86E-06	ILMN_1743145	ERAP1
					Geneva GenCord	GenCord-T	2.50E-07	14	3.50E-06	ILMN_1743145	ERAP1
					Geneva GenCord	GenCord-F	1.44E-02	14	2.02E-01	ILMN_1758661	LNPEP
					Geneva GenCord	GenCord-F	2.62E-02	14	3.67E-01	ILMN_1655329	RIOK2
					MuTHER Pilot	Twin1-A	2.80E-05	18	5.04E-04	ILMN_2336220	ERAP1
					MuTHER Pilot	Twin1-L	5.10E-06	18	9.18E-05	ILMN_2336220	ERAP1
					MuTHER Pilot	Twin2-A	7.60E-07	18	1.37E-05	ILMN_2336220	ERAP1
					MuTHER Pilot	Twin2-L	3.20E-07	18	5.76E-06	ILMN_2336220	ERAP1
					Geneva GenCord	GenCord-F	1.80E-03				IL10
					MuTHER Pilot	Twin2-A	1.00E-04	18	1.80E-03	ILMN_2336220	ERAP1
					MuTHER Pilot	Twin2-L	1.19E-02	18	2.14E-01	ILMN_2322806	CAST
					MuTHER Pilot	Twin1-L	4.05E-02	18	7.29E-01	ILMN_2123312	ERAP2
HLA-B	rs4959053	6	31099577	no	Geneva GenCord	GenCord-L	**1.98E-02**	121	2.40E+00	ILMN_1653591	TRIM40
					Geneva GenCord	GenCord-F	8.50E-03	121	1.03E+00	ILMN_1675282	
					Geneva GenCord	GenCord-F	4.68E-02	121	5.66E+00	ILMN_2413517	TRIM39
					Geneva GenCord	GenCord-F	1.27E-02	121	1.54E+00	ILMN_2186597	RPP21
					Geneva GenCord	GenCord-F	5.10E-03	121	6.17E-01	ILMN_2392674	RPR3
					Geneva GenCord	GenCord-F	1.04E-02	121	1.26E+00	ILMN_1763875	ABCF1
					Geneva GenCord	GenCord-F	8.10E-03	121	9.80E-01	ILMN_2392635	ABCF1
					Geneva GenCord	GenCord-F	4.70E-03	121	5.69E-01	ILMN_2121282	MRPS18B
					Geneva GenCord	GenCord-F	8.00E-04	121	9.68E-02	ILMN_1721337	MRPS18B
					Geneva GenCord	GenCord-F	8.30E-03	121	1.00E+00	ILMN_2101885	TUBB
					Geneva GenCord	GenCord-F	1.39E-02	121	1.68E+00	ILMN_1665583	TUBB
					Geneva GenCord	GenCord-F	1.00E-03	121	1.21E-01	ILMN_1800308	GTF2H4
					Geneva GenCord	GenCord-F	7.00E-03	121	8.47E-01	ILMN_1737585	VARS2
					Geneva GenCord	GenCord-F	1.34E-02	121	1.62E+00	ILMN_1708006	MICB
					Geneva GenCord	GenCord-F	5.00E-03	121	6.05E-01	ILMN_1800461	CSNK2B
					Geneva GenCord	GenCord-F	2.82E-02	121	3.41E+00	ILMN_1696295	LY6G6D
					Geneva GenCord	GenCord-F	2.00E-02	121	2.42E+00	ILMN_2307407	C6orf25
					Geneva GenCord	GenCord-F	4.75E-02	121	5.75E+00	ILMN_2325394	MSH5
					Geneva GenCord	GenCord-F	9.20E-03	121	1.11E+00	ILMN_1675708	MSH5
					Geneva GenCord	GenCord-F	1.24E-02	121	1.50E+00	ILMN_2134765	C6orf26
					Geneva GenCord	GenCord-F	1.99E-02	121	2.41E+00	ILMN_1660436	HSPA1B
					Geneva GenCord	GenCord-F	5.50E-03	121	6.66E-01	ILMN_1765532	RDBP
					Geneva GenCord	GenCord-F	3.34E-02	121	4.04E+00	ILMN_1700791	EHMT2
					Geneva GenCord	GenCord-F	4.65E-02	121	5.63E+00	ILMN_1730977	
					Geneva GenCord	GenCord-F	3.50E-03	121	4.24E-01	ILMN_2070300	LSM2
					Geneva GenCord	GenCord-F	7.80E-03	121	9.44E-01	ILMN_1696601	VARS2
					Geneva GenCord	GenCord-F	1.54E-02	121	1.86E+00	ILMN_1756982	CLIC1
					Geneva GenCord	GenCord-F	4.00E-02	121	4.84E+00	ILMN_1795286	C6orf47
					Geneva GenCord	GenCord-F	3.90E-03	121	4.72E-01	ILMN_2390416	BAT3
					Geneva GenCord	GenCord-F	1.51E-02	121	1.83E+00	ILMN_1705364	BAT3
					Geneva GenCord	GenCord-F	3.07E-02	121	3.71E+00	ILMN_1690576	BAT1
					Geneva GenCord	GenCord-F	2.01E-02	121	2.43E+00	ILMN_1686626	BAT1
					Geneva GenCord	GenCord-F	1.36E-02	121	1.65E+00	ILMN_2357976	BAT1
					Geneva GenCord	GenCord-L	3.24E-02	121	3.92E+00	ILMN_1653447	PSORS1C2

					Geneva GenCord	GenCord-F	4.60E-03	121	5.57E-01	ILMN_1682717	IER3
					Geneva GenCord	GenCord-F	1.84E-02	121	2.23E+00	ILMN_1674051	CCHCR1
					Geneva GenCord	GenCord-F	3.50E-03	121	4.24E-01	ILMN_1661439	FLOT1
					Geneva GenCord	GenCord-F	1.41E-02	121	1.71E+00	ILMN_1814122	MDC1
					Geneva GenCord	GenCord-F	3.52E-02	121	4.26E+00	ILMN_1732967	KIAA1949
					Geneva GenCord	GenCord-F	4.50E-03	121	5.45E-01	ILMN_1716922	DHX16
					Geneva GenCord	GenCord-F	8.40E-03	121	1.02E+00	ILMN_1789457	GNL1
					Geneva GenCord	GenCord-F	1.23E-02	121	1.49E+00	ILMN_1738704	TRIM26
					Geneva GenCord	GenCord-F	1.78E-02	121	2.15E+00	ILMN_1680993	TRIM10
					MuTHER Pilot	Twin2-S	4.57E-02	126	5.76E+00	ILMN_1660904	
					MuTHER Pilot	Twin2-L	4.20E-03	126	5.29E-01	ILMN_1730497	
					MuTHER Pilot	Twin1-A	4.49E-02	126	5.66E+00	ILMN_2280480	TCF19
					MuTHER Pilot	Twin2-S	1.74E-02	126	2.19E+00	ILMN_1682008	TCF19
					MuTHER Pilot	Twin2-L	4.81E-02	126	6.06E+00	ILMN_1803945	HCP5
					MuTHER Pilot	Twin2-L	2.36E-02	126	2.97E+00	ILMN_1731941	
					MuTHER Pilot	Twin2-L	4.04E-02	126	5.09E+00	ILMN_1709799	C6orf25
					MuTHER Pilot	Twin2-L	4.72E-02	126	5.95E+00	ILMN_2115350	TNXB
					MuTHER Pilot	Twin1-L	2.99E-02	126	3.77E+00	ILMN_1684614	TNXB
					MuTHER Pilot	Twin2-A	2.35E-02	126	2.96E+00	ILMN_1742782	
					MuTHER Pilot	Twin2-L	2.57E-02	126	3.24E+00	ILMN_1674778	
					MuTHER Pilot	Twin1-A	3.30E-03	126	4.16E-01	ILMN_1690576	
					MuTHER Pilot	Twin2-A	2.18E-02	126	2.75E+00	ILMN_1653447	
					MuTHER Pilot	Twin1-A	3.40E-02	126	4.28E+00	ILMN_1661439	FLOT1
					MuTHER Pilot	Twin1-L	4.47E-02	126	5.63E+00	ILMN_1814122	
					MuTHER Pilot	Twin1-L	4.91E-02	126	6.19E+00	ILMN_1694208	
					MuTHER Pilot	Twin1-A	4.91E-02	126	6.19E+00	ILMN_1680993	
					Geneva GenCord	GenCord-F	2.87E-02				CCR1
					Geneva GenCord	GenCord-L	1.32E-02			ILMN_1719093	KLRC4
IL10	rs1554286	1	206944233	yes	Geneva GenCord	GenCord-F	4.93E-02	47	2.32E+00	ILMN_2387224	CTSE
					Geneva GenCord	GenCord-L	5.00E-04	47	2.35E-02	ILMN_1656144	MAPKAPk2
					Geneva GenCord	GenCord-L	3.89E-02	47	1.83E+00	ILMN_1694588	C4BPB
					Geneva GenCord	GenCord-T	2.60E-02	47	1.22E+00	ILMN_1676725	C1orf116
					Geneva GenCord	GenCord-T	1.18E-02	47	5.55E-01	ILMN_1801743	FCAMR
					Geneva GenCord	GenCord-T	2.11E-02	47	9.92E-01	ILMN_2073307	IL10
					Geneva GenCord	GenCord-F	4.30E-03	47	2.02E-01	ILMN_1726081	SLC26A9
					MuTHER Pilot	Twin1-L	7.40E-03	53	3.92E-01	ILMN_1799887	CTSE
					MuTHER Pilot	Twin1-S	1.71E-02	53	9.06E-01	ILMN_1682592	Il19
					MuTHER Pilot	Twin2-L	2.18E-02	53	1.16E+00	ILMN_1799575	Il19
					MuTHER Pilot	Twin1-L	3.57E-02	53	1.89E+00	ILMN_1774685	IL24
					MuTHER Pilot	Twin1-L	2.32E-02	53	1.23E+00	ILMN_2385298	PFKFB2
					MuTHER Pilot	Twin1-A	4.09E-02	53	2.17E+00	ILMN_1723436	PFKFB2
					MuTHER Pilot	Twin1-L	4.23E-02	53	2.24E+00	ILMN_2347789	C4BPB
					MuTHER Pilot	Twin1-L	3.27E-02	53	1.73E+00	ILMN_1800540	CD55
					MuTHER Pilot	Twin2-A	1.82E-02	53	9.65E-01	ILMN_1676725	C1orf116
					MuTHER Pilot	Twin1-S	3.02E-02	53	1.60E+00	ILMN_1685387	PIGR
					MuTHER Pilot	Twin2-L	2.54E-02	53	1.35E+00	ILMN_1674167	IL10
					MuTHER Pilot	Twin2-L	1.33E-02	53	7.05E-01	ILMN_1726081	SLC26A9
					Geneva GenCord	GenCord-F	8.80E-03			ILMN_1799669	PSORS1C1
					Geneva GenCord	GenCord-F	1.69E-02			ILMN_1761921	IL12RB2
					Geneva GenCord	GenCord-F	4.63E-02			ILMN_1799669	IL10
NAALADL2	rs3914501	3	174564668	yes	HapMap3 (GIH)	Lymphoblastoid cell line	4.90E-03	2	9.80E-03	ILMN_1739521	
TGFBR3	rs284148	1	92277843	yes	HapMap3 (JPT)	Lymphoblastoid cell line	1.95E-02	19	3.71E-01	ILMN_1657665	ZNF644
	rs1805110	1	92327045	yes	HapMap3 (JPT)	Lymphoblastoid cell line	1.50E-02	22	3.30E-01	ILMN_1657665	ZNF644
					Geneva GenCord	CeneCord-F	2.23E-02	21	4.68E-01	ILMN_2247703	BRDT
					MuTHER Pilot	Twin1-A	3.61E-02	28	1.01E+00	ILMN_1667928	BRDT
					MuTHER Pilot	Twin1-L	4.11E-02	28	1.15E+00	ILMN_1796773	BTBD8
					MuTHER Pilot	Twin2-L	1.31E-02	28	3.67E-01	ILMN_2083742	C1orf146
					MuTHER Pilot	Twin2-S	7.50E-03	28	2.10E-01	ILMN_1784287	TGFBR3
					HapMap3 (CHB)	Lymphoblastoid cell line	4.48E-02			ILMN_1743455	IL12RB2
					Geneva GenCord	GenCord-L	3.61E-02			ILMN_1719093	KLRC4
YIPF7	rs6838327	4	44626846	yes	HapMap3 (JPT)	Lymphoblastoid cell line	4.82E-02	4	1.93E-01	ILMN_1754570	IL12RB2
					MuTHER Pilot	Twin1-A	3.00E-04	5	1.50E-03	ILMN_1696330	GUF1
					MuTHER Pilot	Twin1-L	4.00E-04	5	2.00E-03	ILMN_1696330	GUF1
					MuTHER Pilot	Twin1-A	2.10E-03	5	1.05E-02	ILMN_1696330	GUF1
					MuTHER Pilot	Twin1-L	9.00E-04	5	4.50E-03	ILMN_1696330	GUF1

[*] No. of cis-probes located ±1,000 kbp of each SNP obtained from HapMap3 (CEU, CHB, GIH, JPT, LWK, MEX, MKK, YRI) (Stranger et al., 2012), Geneva Gencord (Dimas et al., 2009), and MuTHER pilot (Nica et al., 2011) studies.

[¶] *P* Values obtained from correlation analysis between SNP genotypes and gene expression levels of cis-probes using Spearman's rank correlation coefficient (rho). Data were chosen with *P* Value less than 0.05

†*P* value obtained from correlation analysis after Bonferroni test based on number of cis-probes.

Yellow shows SNPs that passed the Bonferroni correction

Chr., chromosome; eQTL, expression quantitative trait loci; LD, linkage disequilibrium; Position, chromosome position (hg19); rSNP, regulatory SNP; F, fibroblast; T, T-cell; S, skin; F, fat; L, lymphoblastoid cell line.

Orange shows *P* value obtained from 10,000 permutations.

[¥] Genes affected by at least two unlinked trait-associated SNPs.

RegulomeDB analysis

Locus				regulatory SNP (from rSNPBase)				RegulomeDB						
Nearby Gene	SNP	Chr.	Position	LD-proxy of rSNP (r2>0.8)	Proximal regulati	Distal regulati	RNA binding protein mediated regulatio	Description	Regulome DB Sc	Metho	Bound protein (or motif)	Affected gene	Cell type	Reference
IL23R-IL12RB2	rs121191	1	###############	yes	no	no	no							
IL23R-IL12RB2	rs149596	1	###############	yes	no	no	no							
TGFBR3	rs178828	1	###############	yes	yes	no	yes	Likely to affect binding	2b	ChIP-seq	CEBPB		HeLa-S3, HepG2	
TGFBR3	rs284148	1	###############	yes	no	no	yes	No data						
TGFBR3	rs180511	1	###############	yes	no	no	yes	No data						
NHLH2	rs755658	1	###############	yes	yes	no	yes							
IL10	rs155428	1	###############	yes	no	no	yes	Likely to affect binding and linked to expression of a gene target	1f	ChIP-seq	CREBBP		Jurkat	PMID: 20019798
IL10	rs151811	1	###############	yes	yes	no	yes	Less likely to affect binding	3a	ChIP-seq	CREBBP		Jurkat	PMID: 20019798
IL10	rs180087	1	###############	yes	yes	no	no	minimal binding evidence	5	DNase-seq	Monocd14			
STAT4	rs757407	2	###############	yes	no	no	yes	Minimal binding evidence	6	eQTL		STAT4		PMID:18846210
CCR1-CCR3	rs574290	3	###############	yes	no	no	yes							
IQCJ-SCHIP1,SCHIP1	rs168305	3	###############	yes	no	no	yes	No data						
IQCJ-SCHIP1	rs168305	3	###############	yes	no	no	yes	No data						
NAALADL2	rs391450	3	###############	yes	no	no	no	No data						
NAALADL2	rs168481	3	###############	no	no	no	no	No data						
NAALADL2	rs986656	3	###############	no	no	no	no	Minimal binding evidence	6	PWM	Dobox4			ENCODE
DCAF12	rs183513	3	###############	no	no	no	no	minimal binding evidence	6	PWM	Spdef			ENCODE
YIPF7	rs201334	4	###############	no	no	no	yes							
YIPF7	rs683832	4	###############	yes	no	no	yes	Minimal binding evidence	6	eQTL		GUF1, FLJ13220	Lymphoblastoid, Monocytes	PMID:18846210, 20502693
YIPF7	rs192398	4	###############	no	no	no	yes	Minimal binding evidence	6	PWM	Sox4			PMID:19443739
YIPF7	rs374634	4	###############	no	no	no	no							
	rs170076	5	###############	yes	no	no	no							
CD180	rs32019	5	###############	yes	no	yes	no	No data						
ERAP1	rs174820	5	###############	yes	yes	yes	yes	minimal binding evidence	4	ChIP-seq	RFX3		K562	ENCODE
ERAP1-ERAP2	rs292761	5	###############	yes	no	no	no	Likely to affect binding and linked to expression of a gene target	1f	eQTL		ERAP1	Lymphoblastoid	PMID: 19644074
HLA-F-AS1–HLA-A, IFITM	rs471324	6	###############	no	no	no	no							
PSORS1C1	rs121917	6	###############	no	no	no	no							
PSORS1C1, CDSN	rs774203	6	###############	no	no	no	no							
PSORS1C1 (HLA-B)	rs495905	6	###############	no	no	no	no	minimal binding evidence	4	ChIP-seq	POU5F1			
PSORS1C1	rs125251	6	###############	no	no	no	no	No data						
	rs265565	6	###############	yes	no	no	no							
ELMO1	rs102595	7	###############	yes	no	no	no	No data						
DCAF12	rs104417	9	###############	yes	yes	no	no	minimal binding evidence	6	PWM	AP-3			ENCODE
DCAF12	rs150474	9	###############	no	no	no	yes	No data						
DCAF12	rs107582	9	###############	yes	no	no	no	minimal binding evidence	5	ChIP-seq	GATA3		T-47D	ENCODE
NRG3	rs111922	10	###############	yes	no	no	yes							
MIR548F5, NBEA	rs450059	13	###############	yes	no	no	yes							
	rs168159	14	###############	yes	no	yes	no							
CD300C/CD300LB	rs617301	17	###############	no	no	no	yes	minimal binding evidence						
LOC284395	rs724573	19	###############	no	no	no	no							
PLCB1	rs126248	20	###############	yes	no	no	yes							
PLCB1	rs608663	20	###############	yes	no	no	yes							
PLCB1	rs608663	20	###############	yes	no	yes	yes							
PLCB1	rs603930	20	###############	yes	no	no	yes							
PLCB1	rs608665	20	###############	yes	no	no	yes	No data						

*RegulomeDB score of less than 3, indicating a relatively high degree of evidence for potential regulatory function (''likely to affect binding'').

PWM: positional weight matrices

HaploReg analysis

Query SNP: rs32019 and variants with r^2 >= 0.8

chr	pos (hg19)	LD (r^2)	LD (D')	variant	Ref	Alt	AFR freq	AMR freq	ASN freq	EUR freq	SiPhy cons	Promoter histone marks	Enhancer histone marks	DNAse	Proteins bound	eQTL tissues	Motifs changed	GENCODE genes	dbSNP func annot
5	66684694	0.88	0.99	rs452189	G	A	0.8	0.59	0.57	0.53							BRCA1,Foxj2	RP11-434D9.1	
5	66684710	0.88	0.99	rs413765	C	A	0.19	0.44	0.57	0.45								RP11-434D9.1	
5	66684916	0.89	0.99	rs393411	T	C	0.18	0.44	0.57	0.45							Nkx3	RP11-434D9.1	
5	66685111	0.89	0.99	rs424331	G	A	0.81	0.58	0.57	0.52							6 altered motifs	RP11-434D9.1	
5	66685685	0.89	1	rs1592797	T	C	0.64	0.57	0.57	0.52							DMRT7,Gfi1,Mef2	RP11-434D9.1	
5	66686570	0.95	1	rs317969	T	C	0.28	0.45	0.55	0.45							5 altered motifs	RP11-434D9.1	
5	66686935	0.98	1	rs317970	A	T	0.2	0.43	0.55	0.45				4 cell types			Bcl6b,Pou2f2,STAT	RP11-434D9.1	
5	66688181	0.99	1	rs319249	C	G	0.63	0.56	0.55	0.52			NHEK				Smad	RP11-434D9.1	
5	66689922	0.9	1	rs319246	A	C	0.65	0.56	0.57	0.52			Huvec				6 altered motifs	RP11-434D9.1	
5	66690232	0.9	1	rs446231	A	C	0.28	0.45	0.57	0.45			Huvec				Pou5f1,Sox	RP11-434D9.1	
5	66691106	0.93	1	rs319245	C	T	0.64	0.56	0.56	0.52			Huvec				Pax-4,Pax-5,p300	RP11-434D9.1	
5	66691614	0.93	1	rs1428608	T	C	0.64	0.56	0.56	0.52			Huvec				RXRA	RP11-434D9.1	
5	66691700	0.93	1	rs1835046	G	T	0.64	0.56	0.56	0.52			Huvec	HMVEC-LLy	CTCF,RAD21		8 altered motifs	RP11-434D9.1	
5	66691710	0.93	1	rs1835045	C	T	0.63	0.56	0.56	0.52			Huvec	HMF,HMVEC-LLy	CTCF,RAD21		HDAC2,Irf,Pou2f2	RP11-434D9.1	
5	66691735	0.93	1	rs427861	A	G	0.65	0.56	0.56	0.52			Huvec	HMF,HMVEC-[illegible]	CTCF,RAD21		ZEB1	RP11-434D9.1	
5	66692401	0.99	1	rs407455	C	T	0.61	0.56	0.55	0.51				SK-N-MC				RP11-434D9.1	
5	66693056	0.91	1	rs2545140	T	G	0.65	0.57	0.57	0.54							CACD,CTCF,RREB-1	RP11-434D9.1	
5	66693157	0.94	1	rs627290	C	T	0.18	0.43	0.56	0.44								RP11-434D9.1	
5	66693907	0.93	1	rs389617	T	C	0.19	0.43	0.56	0.44							6 altered motifs	RP11-434D9.1	
5	66694983	0.93	0.98	rs655993	G	A	0.58	0.57	0.55	0.54							SETDB1,Zic	RP11-434D9.1	
5	66695077	0.97	1	rs570892	C	T	0.19	0.43	0.55	0.44							5 altered motifs	RP11-434D9.1	
5	66695433	0.97	1	rs2431236	T	C	0.78	0.6	0.55	0.54							EWSR1-FLI1,Pbx3,STAT	RP11-434D9.1	
5	66696246	0.97	1	rs2635679	A	C	0.26	0.44	0.55	0.44							Pou6f1	RP11-434D9.1	
5	66696747	0.97	1	rs160843	G	A	0.18	0.43	0.55	0.44				6 cell types			Foxd3	RP11-434D9.1	
5	66697639	0.97	1	rs32015	G	A	0.18	0.43	0.55	0.45							Foxp3,Pou3f2,Zfp187	RP11-434D9.1	
5	66699876	1	1	rs39919	C	A	0.74	0.56	0.54	0.51			Huvec, HMEC				10 altered motifs	RP11-434D9.1	
5	66700058	1	1	rs32016	C	T	0.6	0.54	0.54	0.51			HMEC, NHEK, Huvec				Pou2f2	RP11-434D9.1	
5	66700078	1	1	rs32017	C	A	0.73	0.56	0.54	0.51			HMEC, NHEK, Huvec				10 altered motifs	RP11-434D9.1	
5	66700677	1	1	rs151118863	ATTGT	A	0.22	0.43	0.54	0.44			4 cell types	38 cell types	CFOS,CJUN,STAT3		22 altered motifs	RP11-434D9.1	
5	66700683	0.84	1	rs5868487	TG	7-mer	0.2	0.38	0.5	0.41			4 cell types	38 cell types	STAT3			RP11-434D9.1	
5	66701037	1	1	rs32018	C	T	0.22	0.43	0.54	0.44			4 cell types					RP11-434D9.1	
5	66701568	0.97	1	rs648088	C	T	0.6	0.54	0.55	0.51			Huvec					RP11-434D9.1	
5	66701766	0.97	1	rs42275	G	A	0.22	0.43	0.55	0.44			Huvec					RP11-434D9.1	
5	66702373	1	1	rs32019	C	T	0.6	0.54	0.54	0.51							Mrg1::Hoxa9	RP11-434D9.1	
5	66702823	1	1	rs32020	G	A	0.22	0.43	0.54	0.44							BHLHE40,ELF1	RP11-434D9.1	
5	66703862	1	1	rs32021	C	T	0.63	0.54	0.54	0.51							Foxp1,Pax-3	RP11-434D9.1	
5	66704329	0.97	1	rs160975	G	C	0.6	0.57	0.55	0.55							6 altered motifs	RP11-434D9.1	
5	66704807	0.99	1	rs32022	G	A	0.55	0.55	0.54	0.52								RP11-434D9.1	
5	66704870	0.99	1	rs32023	C	T	0.55	0.55	0.54	0.52							4 altered motifs	RP11-434D9.1	
5	66705182	0.95	1	rs32024	T	C	0.58	0.55	0.56	0.52				HL-60,Monocytes-				RP11-434D9.1	
5	66707003	1	1	rs32026	G	A	0.2	0.44	0.54	0.45								RP11-434D9.1	
5	66707105	0.97	1	rs32027	T	A	0.2	0.44	0.53	0.45							10 altered motifs	RP11-434D9.1	
5	66708143	0.99	1	rs32028	G	A	0.56	0.54	0.54	0.52							AP-1,p53	RP11-434D9.1	
5	66708354	0.99	1	rs1611103	GACAA	G	0.2	0.44	0.54	0.45							13 altered motifs	RP11-434D9.1	
5	66708492	0.99	1	rs32029	A	T	0.55	0.57	0.55	0.53							9 altered motifs	RP11-434D9.1	
5	66711584	0.97	1	rs2371961	T	A	0.23	0.45	0.55	0.45			H1, GM12878				GR,NRSF	RP11-434D9.1	
5	66714011	0.94	0.99	rs2707772	A	C	0.23	0.44	0.55	0.45			H1				4 altered motifs	RP11-434D9.1	
5	66716144	0.95	0.99	rs160978	C	T	0.62	0.58	0.55	0.52							6 altered motifs	RP11-434D9.1	
5	66720725	0.9	0.99	rs255290	G	A	0.18	0.44	0.56	0.45							7 altered motifs	RP11-434D9.1	
5	66721085	0.87	0.99	rs173876	T	G	0.66	0.6	0.57	0.55							6 altered motifs	RP11-434D9.1	
5	66722075	0.91	0.99	rs160979	G	T	0.63	0.58	0.56	0.53							RXRA	RP11-434D9.1	
5	66722278	0.87	0.99	rs173875	T	C	0.18	0.44	0.57	0.46							4 altered motifs	RP11-434D9.1	
5	66722470	0.91	0.99	rs160980	C	T	0.63	0.58	0.56	0.53			HMEC				HNF4	RP11-434D9.1	
5	66722496	0.91	0.99	rs160981	A	G	0.2	0.51	0.56	0.53			HMEC				7 altered motifs	RP11-434D9.1	
5	66723345	0.91	0.99	rs160983	T	G	0.63	0.6	0.56	0.54				5 cell types			4 altered motifs	RP11-434D9.1	
5	66724526	0.91	0.99	rs160976	T	C	0.63	0.6	0.56	0.55							9 altered motifs	RP11-434D9.1	
5	66725048	0.88	0.97	rs32041	G	C	0.18	0.45	0.56	0.46							10 altered motifs	RP11-434D9.1	
5	66731693	0.85	0.98	rs42277	G	C	0.35	0.48	0.57	0.47							Ets,Foxq1	RP11-434D9.1	
5	66732702	0.83	0.98	rs10689791	A	ATC	0.72	0.62	0.58	0.54							GR,NERF1a	RP11-434D9.1	
5	66733238	0.88	0.98	rs191781	G	A	0.26	0.45	0.56	0.46								RP11-434D9.1	

5	66733417	0.91	0.98	rs32039	G	A	0.18	0.44	0.55	0.46							Evi-1,Pbx-1	RP11-434D9.1	
5	66734034	0.92	0.98	rs32038	G	A	0.5	0.49	0.55	0.46				HGF,SK-N-MC			Foxj2,HNF4,SRF	RP11-434D9.1	

Query SNP: rs10259514 and variants with r^2 >= 0.8

chr	pos (hg19)	LD (r²)	LD (D')	variant	Ref	Alt	AFR freq	AMR freq	ASN freq	EUR freq	SiPhy cons	Promoter histone marks	Enhancer histone marks	DNAse	Proteins bound	eQTL tissues	Motifs changed	GENCODE genes	dbSNP func annot
7	36829255	0.92	0.99	rs17170718	G	C	0.13	0.3	0.25	0.27			HMEC, NHEK				Nkx2	6.9kb 3' of AC007349.5	
7	36829705	1	1	rs10259514	A	G	0.13	0.3	0.26	0.27							Hltf,YY1	7.3kb 3' of AC007349.5	
7	36830027	0.93	1	rs79472482	T	C	0.03	0.19	0.24	0.14				HMVEC-dAd,HMVEC-dNeo	GATA2		RP58	7.6kb 3' of AC007349.5	
7	36831664	0.93	0.98	rs16879489	C	T	0.12	0.28	0.25	0.26							Zfp691	9.3kb 3' of AC007349.5	
7	36833400	0.91	0.98	rs10259131	T	C	0.03	0.19	0.25	0.14								11kb 3' of AC007349.5	
7	36834767	0.9	0.99	rs77575853	A	G	0.03	0.19	0.24	0.14			HMEC, NHEK, HepG2				4 altered motifs	12kb 3' of AC007349.5	
7	36834864	0.9	0.99	rs77923191	G	A	0.03	0.19	0.24	0.14			HMEC, NHEK, HepG2				HNF1,TCF11::MafG	12kb 3' of AC007349.5	
7	36838148	0.89	0.98	rs4723575	A	G	0.03	0.19	0.24	0.14							4 altered motifs	16kb 3' of AC007349.5	
7	36839372	0.88	0.97	rs4723576	A	T	0.05	0.18	0.24	0.14							7 altered motifs	17kb 3' of AC007349.5	
7	36841360	0.83	0.94	rs199934005	T	TG	0.05	0.19	0.25	0.16							5 altered motifs	19kb 3' of AC007349.5	
7	36848291	0.86	0.96	rs10277512	T	A	0.03	0.19	0.24	0.14							5 altered motifs	26kb 3' of AC007349.5	
7	36849138	0.86	0.96	rs76494870	T	C	0.07	0.19	0.24	0.14							NRSF,Sox	27kb 3' of AC007349.5	
7	36852128	0.88	0.98	rs28411180	A	G	0.1	0.19	0.24	0.14			HMEC	GM19239			Rad21	26kb 5' of AC007349.7	
7	36856630	0.81	0.93	rs28811959	A	G	0.08	0.19	0.24	0.14							Homez,Mef2	22kb 5' of AC007349.7	
7	36858203	0.81	0.93	rs10238019	T	C	0.08	0.19	0.24	0.14								20kb 5' of AC007349.7	
7	36865943	0.8	0.92	rs6462712	C	G	0.09	0.28	0.25	0.25							4 altered motifs	13kb 5' of AC007349.7	

Query SNP: rs17482078 and variants with r^2 >= 0.8

chr	pos (hg19)	LD (r²)	LD (D')	variant	Ref	Alt	AFR freq	AMR freq	ASN freq	EUR freq	SiPhy cons	Promoter histone marks	Enhancer histone marks	DNAse	Proteins bound	eQTL tissues	Motifs changed	GENCODE genes	dbSNP func annot
5	96118866	1	1	rs17482078	C	T	0.06	0.13	0.07	0.22				BE2_C				ERAP1	missense
5	96119339	1	1	rs2124921	G	T	0.08	0.13	0.07	0.22				HFF,HPAF			6 altered motifs	ERAP1	intronic
5	96121152	0.92	1	rs2032890	A	C	0.14	0.21	0.08	0.32								CTD-2260A17.1	intronic
5	96121184	1	1	rs13185488	G	A	0.05	0.13	0.07	0.22								CTD-2260A17.1	intronic
5	96121243	1	1	rs13186739	C	T	0.05	0.13	0.07	0.22							7 altered motifs	CTD-2260A17.1	intronic
5	96122210	1	1	rs10050860	C	T	0.05	0.14	0.07	0.23							GR,YY1	ERAP1	missense
5	96122458	1	1	rs13154629	C	T	0.05	0.13	0.07	0.22							HNF4,Sox	ERAP1	intronic
5	96123429	1	1	rs35983588	A	G	0.05	0.13	0.07	0.23							5 altered motifs	ERAP1	intronic
5	96125785	0.85	0.92	rs13170045	G	T	0.05	0.13	0.07	0.22				HPdLF			5 altered motifs	ERAP1	intronic
5	96126938	0.85	0.92	rs71630754	G	A	0.04	0.13	0.07	0.22			5 cell types	50 cell types	CFOS,CJUN		CEBPB,TAL1,p300	ERAP1	intronic
5	96129535	0.85	0.92	rs2287987	T	C	0.05	0.13	0.07	0.22							5 altered motifs	ERAP1	missense
5	96130189	0.85	0.92	rs13168673	C	T	0.06	0.13	0.07	0.22							4 altered motifs	ERAP1	intronic
5	96131483	0.85	0.92	rs13173167	G	T	0.05	0.13	0.07	0.22							Nkx2,Nkx3,TATA	ERAP1	intronic
5	96131599	0.8	0.92	rs13173351	G	C	0.05	0.13	0.07	0.22							6 altered motifs	ERAP1	intronic
5	96134175	0.85	0.92	rs2013717	T	G	0.07	0.14	0.07	0.22							6 altered motifs	ERAP1	intronic
5	96134283	0.85	0.92	rs2013671	T	C	0.05	0.13	0.07	0.22							5 altered motifs	ERAP1	intronic
5	96134306	0.85	0.92	rs2013633	T	C	0.05	0.14	0.07	0.22							GATA,Lmo2-complex,Mef2	ERAP1	intronic
5	96134915	0.85	0.92	rs113003291	GATTAA	G	0.05	0.13	0.07	0.23							15 altered motifs	ERAP1	intronic
5	96137140	0.85	0.92	rs10074694	G	A	0.05	0.13	0.07	0.22							BRCA1,EBF	ERAP1	intronic
5	96137204	0.85	0.92	rs10074708	G	A	0.05	0.13	0.07	0.22							5 altered motifs	ERAP1	intronic
5	96139066	0.85	0.92	rs10062964	C	T	0.05	0.13	0.07	0.22		GM12878					Egr-1	ERAP1	intronic
5	96139714	0.85	0.92	rs35307952	AG	A	0.05	0.13	0.07	0.22			GM12878				4 altered motifs	ERAP1	intronic
5	96139866	0.85	0.92	rs10043309	A	C	0.05	0.13	0.07	0.22		GM12878	K562				NF-Y,Pou2f2	ERAP1	intronic
5	96140740	0.85	0.92	rs10041240	C	T	0.05	0.13	0.07	0.22			GM12878	GM18507,CD34+_Mobilized Monocyt			Foxj1,GZF1	ERAP1	intronic
5	96141107	0.85	0.92	rs34387283	GA	G	0.05	0.13	0.07	0.22		GM12878	NHEK, HMEC				5 altered motifs	ERAP1	intronic

Query SNP: rs2927615 and variants with r^2 >= 0.8

chr	pos (hg19)	LD (r²)	LD (D')	variant	Ref	Alt	AFR freq	AMR freq	ASN freq	EUR freq	SiPhy cons	Promoter histone marks	Enhancer histone marks	DNAse	Proteins bound	eQTL tissues	Motifs changed	GENCODE genes	dbSNP func annot
5	96198202	1	1	rs2927615	G	A	0.09	0.15	0.05	0.27				HMEC				CTD-2260A17.2	
5	96199493	0.85	0.96	rs2927612	A	G	0.03	0.04	0.05	0.12							BDP1,GR,Pbx3	CTD-2260A17.2	
5	96200439	0.85	0.96	rs2927611	G	T	0.03	0.04	0.05	0.12							CCNT2,Hmbox1,RREB-1	CTD-2260A17.2	
5	96201583	0.85	0.96	rs2617443	T	G	0.01	0.04	0.05	0.12				CMK			CEBPB,Myc	CTD-2260A17.2	
5	96202155	0.85	0.96	rs2617444	A	G	0.01	0.04	0.05	0.12							Irx	CTD-2260A17.2	
5	96204413	0.85	0.96	rs35794116	CA	C	0.01	0.04	0.05	0.12							9 altered motifs	CTD-2260A17.2	
5	96204488	0.85	0.96	rs2617450	G	C	0.03	0.04	0.05	0.12							MZF1::1-4	CTD-2260A17.2	
5	96209820	0.85	0.96	rs142509426	GGAAA	G	0.01	0.04	0.05	0.12			K562				5 altered motifs	CTD-2260A17.2	

Query SNP: rs4959053 and variants with r^2 >= 0.8

chr	pos (hg19)	LD (r²)	LD (D')	variant	Ref	Alt	AFR freq	AMR freq	ASN freq	EUR freq	SiPhy cons	Promoter histone marks	Enhancer histone marks	DNAse	Proteins bound	eQTL tissues	Motifs changed	GENCODE genes	dbSNP func annot
6	31094971	0.98	1	rs10947137	A	C	0.01	0.08	0.1	0.08			HSMM, K562, HMEC				Rad21,SMC3	PSORS1C1	intronic
6	31099577	1	1	rs4959053	G	A	0.01	0.07	0.09	0.08			H1	H7-hESC	POU5F1			PSORS1C1	intronic
6	31099761	1	1	rs12525170	G	A	0.01	0.07	0.09	0.08							TBX5	PSORS1C1	intronic
6	31102273	0.8	1	rs75881311	T	A	0	0	0.08	0.01							SZF1-1,Spz1	PSORS1C1	intronic

Query SNP: rs1554286 and variants with r^2 >= 0.8

chr	pos (hg19)	LD (r²)	LD (D')	variant	Ref	Alt	AFR freq	AMR freq	ASN freq	EUR freq	SiPhy cons	Promoter histone marks	Enhancer histone marks	DNAse	Proteins bound	eQTL tissues	Motifs changed	GENCODE genes	dbSNP func annot
1	206944233	1	1	rs1554286	A	G	0.58	0.69	0.34	0.82		K562	4 cell types	Th1	POL2,TBP,POL24H8		GR	IL10	intronic
1	206944645	0.93	1	rs1518111	T	C	0.58	0.67	0.32	0.78		K562	GM12878, H1	14 cell types	PU1,POL2		Ets	IL10	intronic
1	206944861	0.9	0.97	rs1518110	A	C	0.58	0.67	0.33	0.78			GM12878	GM12891,Jurkat			9 altered motifs	IL10	intronic
1	206945311	0.91	0.98	rs3024490	A	C	0.58	0.67	0.33	0.77			GM12878				CEBPD,PLZF,STAT	IL10	intronic
1	206946407	0.94	1	rs1800872	T	G	0.58	0.67	0.32	0.77			GM12878		PU1		T3R	567bp 5' of IL10	
1	206946634	0.94	1	rs1800871	A	G	0.58	0.67	0.32	0.77			GM12878				5 altered motifs	794bp 5' of IL10	

Query SNP: rs1518111 and variants with r^2 >= 0.8

chr	pos (hg19)	LD (r²)	LD (D')	variant	Ref	Alt	AFR freq	AMR freq	ASN freq	EUR freq	SiPhy cons	Promoter histone marks	Enhancer histone marks	DNAse	Proteins bound	eQTL tissues	Motifs changed	GENCODE genes	dbSNP func annot
1	206944233	0.93	1	rs1554286	A	G	0.58	0.69	0.34	0.82		K562	4 cell types	Th1	POL2,TBP,POL24H8		GR	IL10	intronic
1	206944645	1	1	rs1518111	T	C	0.58	0.67	0.32	0.78		K562	GM12878, H1	14 cell types	PU1,POL2		Ets	IL10	intronic
1	206944861	0.97	1	rs1518110	A	C	0.58	0.67	0.33	0.78			GM12878	GM12891,Jurkat			9 altered motifs	IL10	intronic
1	206945311	0.98	1	rs3024490	A	C	0.58	0.67	0.33	0.77			GM12878				CEBPD,PLZF,STAT	IL10	intronic
1	206946407	0.99	1	rs1800872	T	G	0.58	0.67	0.32	0.77			GM12878		PU1		T3R	567bp 5' of IL10	
1	206946634	0.99	1	rs1800871	A	G	0.58	0.67	0.32	0.77			GM12878				5 altered motifs	794bp 5' of IL10	

Query SNP: rs1800871 and variants with r^2 >= 0.8

chr	pos (hg19)	LD (r²)	LD (D')	variant	Ref	Alt	AFR freq	AMR freq	ASN freq	EUR freq	SiPhy cons	Promoter histone marks	Enhancer histone marks	DNAse	Proteins bound	eQTL tissues	Motifs changed	GENCODE genes	dbSNP func annot
1	206944233	0.94	1	rs1554286	A	G	0.58	0.69	0.34	0.82		K562	4 cell types	Th1	POL2,TBP,POL24H8		GR	IL10	intronic
1	206944645	0.99	1	rs1518111	T	C	0.58	0.67	0.32	0.78		K562	GM12878, H1	14 cell types	PU1,POL2		Ets	IL10	intronic
1	206944861	0.96	0.99	rs1518110	A	C	0.58	0.67	0.33	0.78			GM12878	GM12891,Jurkat			9 altered motifs	IL10	intronic
1	206945311	0.97	0.99	rs3024490	A	C	0.58	0.67	0.33	0.77			GM12878				CEBPD,PLZF,STAT	IL10	intronic
1	206946407	1	1	rs1800872	T	G	0.58	0.67	0.32	0.77			GM12878		PU1		T3R	567bp 5' of IL10	
1	206946634	1	1	rs1800871	A	G	0.58	0.67	0.32	0.77			GM12878				5 altered motifs	794bp 5' of IL10	

Query SNP: rs3914501 and variants with r^2 >= 0.8

chr	pos (hg19)	LD (r²)	LD (D')	variant	Ref	Alt	AFR freq	AMR freq	ASN freq	EUR freq	SiPhy cons	Promoter histone marks	Enhancer histone marks	DNAse	Proteins bound	eQTL tissues	Motifs changed	GENCODE genes	dbSNP func annot
3	174546378	0.92	0.97	rs16864898	G	A	0.11	0.29	0.48	0.18							5 altered motifs	NAALADL2	
3	174555714	0.97	0.99	rs7628070	A	G	0.1	0.29	0.49	0.19							Foxa,HNF1	NAALADL2	
3	174556564	0.95	0.99	rs9836047	G	A	0.1	0.29	0.48	0.18							4 altered motifs	NAALADL2	
3	174558124	0.95	0.99	rs3849516	C	T	0.1	0.29	0.48	0.18							Hand1	NAALADL2	
3	174560915	0.81	1	rs7629700	T	C	0.62	0.5	0.54	0.42							4 altered motifs	NAALADL2	
3	174561109	0.99	1	rs7629896	T	A	0.19	0.29	0.49	0.18							5 altered motifs	NAALADL2	
3	174561585	0.83	1	rs7632512	T	A	0.68	0.51	0.53	0.42							5 altered motifs	NAALADL2	
3	174561904	0.84	1	rs3905971	T	C	0.47	0.4	0.53	0.3							HNF1	NAALADL2	
3	174561949	0.82	0.98	rs3905970	G	A	0.25	0.39	0.52	0.3							Irx,Pou2f2,Pou3f2	NAALADL2	
3	174562018	0.99	1	rs3905969	C	T	0.1	0.29	0.49	0.18							YY1	NAALADL2	
3	174562570	0.85	1	rs9868028	G	T	0.47	0.4	0.53	0.3								NAALADL2	
3	174562764	0.85	1	rs9868084	C	T	0.47	0.4	0.53	0.3				H1-hESC,H7-hESC,NT2-	RAD21		5 altered motifs	NAALADL2	
3	174562942	0.83	1	rs9834318	A	T	0.65	0.5	0.53	0.42							FXR	NAALADL2	
3	174563022	0.85	1	rs9872466	G	A	0.41	0.39	0.53	0.3							BDP1,DMRT1	NAALADL2	
3	174563402	0.84	0.99	rs7644152	G	A	0.32	0.39	0.53	0.3								NAALADL2	
3	174563554	0.83	1	rs7636520	A	G	0.62	0.51	0.53	0.42							RXRA	NAALADL2	
3	174563661	0.85	1	rs9873293	C	G	0.32	0.39	0.53	0.3							Tgif1	NAALADL2	
3	174564668	1	1	rs3914501	A	G	0.19	0.32	0.49	0.2							Cdx,TAL1	NAALADL2	
3	174565102	0.84	1	rs10222542	C	A	0.62	0.51	0.53	0.42							RREB-1,Zec,Zfp105	NAALADL2	
3	174565241	0.82	1	rs10222377	A	T	0.54	0.5	0.54	0.42							6 altered motifs	NAALADL2	
3	174565782	0.99	0.99	rs9818108	A	G	0.25	0.29	0.49	0.18							Mef2	NAALADL2	
3	174566513	0.81	0.98	rs3849517	C	T	0.25	0.39	0.53	0.3							Hoxa5,Pax-2,Pax-8	NAALADL2	
3	174566565	0.96	0.98	rs3849518	G	A	0.18	0.28	0.49	0.18							13 altered motifs	NAALADL2	
3	174566904	0.96	0.98	rs9865076	C	T	0.19	0.29	0.49	0.18							16 altered motifs	NAALADL2	
3	174567580	0.96	0.98	rs34933378	C	T	0.19	0.29	0.49	0.18							Egr-1,Hoxa5	NAALADL2	

3	174567744	0.96	0.98	rs9828728	A	G	0.19	0.29	0.49	0.18							Foxj2,NF-kappaB	NAALADL2	
3	174568066	0.81	0.98	rs9832908	A	C	0.47	0.4	0.53	0.31							Smad3	NAALADL2	
3	174568296	0.95	0.98	rs9833244	A	C	0.19	0.29	0.49	0.18							DMRT7	NAALADL2	
3	174568398	0.95	0.98	rs9871227	C	T	0.26	0.29	0.49	0.18							Nkx2,Nkx3	NAALADL2	
3	174569014	0.94	0.97	rs28832882	C	G	0.19	0.29	0.49	0.18							Foxm1	NAALADL2	
3	174569574	0.8	0.98	rs6804977	G	C	0.62	0.51	0.53	0.42				Osteobl			8 altered motifs	NAALADL2	
3	174570094	0.95	0.98	rs9826118	T	C	0.19	0.29	0.49	0.18							Mef2,Nr2f2	NAALADL2	
3	174571478	0.93	0.97	rs10454772	A	T	0.11	0.28	0.49	0.18							20 altered motifs	NAALADL2	
3	174571511	0.93	0.97	rs9831995	T	C	0.19	0.29	0.49	0.18							HNF4,PPAR	NAALADL2	
3	174571717	0.93	0.97	rs9849969	A	G	0.19	0.29	0.49	0.18							7 altered motifs	NAALADL2	
3	174572179	0.91	0.96	rs12185925	G	A	0.35	0.4	0.49	0.29							Hoxa5	NAALADL2	
3	174575665	0.83	0.95	rs9843028	A	C	0.47	0.41	0.47	0.3							Foxp1,Hoxd10,Maf	NAALADL2	
3	174575900	0.83	0.95	rs72242924	TAAA	T	0.38	0.4	0.47	0.3							12 altered motifs	NAALADL2	
3	174577881	0.83	0.95	rs1399810	T	C	0.21	0.39	0.47	0.3				MCF-7,HEEpiC	CEBPB,FOXA1,P300		RXRA	NAALADL2	intronic
3	174578686	0.83	0.95	rs9821396	C	T	0.2	0.39	0.47	0.3							PU.1,TATA	NAALADL2	intronic
3	174579111	0.83	0.95	rs1515625	G	A	0.25	0.4	0.47	0.31							9 altered motifs	NAALADL2	intronic
3	174580594	0.83	0.95	rs6801168	C	T	0.31	0.4	0.47	0.31			NHEK, HMEC	8 cell types			E2A,Smad	NAALADL2	intronic
3	174580752	0.83	0.95	rs6767809	T	C	0.31	0.4	0.47	0.3			HMEC, NHEK	Ishikawa,AG04449	GATA3		Smad4	NAALADL2	intronic
3	174580786	0.83	0.95	rs6801386	C	T	0.31	0.4	0.47	0.31			HMEC, NHEK	Ishikawa,AG04449	GATA3		6 altered motifs	NAALADL2	intronic
Query SNP: rs16848171 and variants with r^2 >= 0.8																			
chr	pos (hg19)	LD (r²)	LD (D')	variant	Ref	Alt	AFR freq	AMR freq	ASN freq	EUR freq	SiPhy cons	Promoter histone marks	Enhancer histone marks	DNAse	Proteins bound	eQTL tissues	Motifs changed	GENCODE genes	dbSNP func annot
3	175180353	1	1	rs10460885	T	C	0.36	0.1	0.26	0.03							GR	NAALADL2	intronic
3	175180518	1	1	rs10460799	T	C	0.17	0.08	0.26	0.03							p300	NAALADL2	intronic
3	175180883	1	1	rs57929088	T	A	0.17	0.08	0.26	0.03				SKMC,WI-38			Sox	NAALADL2	intronic
3	175180903	1	1	rs16825727	A	C	0.17	0.08	0.26	0.03				SKMC,WI-38			CDP	NAALADL2	intronic
3	175180938	1	1	rs73884418	T	C	0.17	0.08	0.26	0.03				SKMC,WI-38			6 altered motifs	NAALADL2	intronic
3	175180991	0.99	1	rs16825731	C	T	0.17	0.08	0.26	0.03				SKMC,WI-38			6 altered motifs	NAALADL2	intronic
3	175181067	1	1	rs16848171	T	C	0.17	0.08	0.26	0.03							Alx4,GR,Smad4	NAALADL2	intronic
Query SNP: rs12624809 and variants with r^2 >= 0.8																			
chr	pos (hg19)	LD (r²)	LD (D')	variant	Ref	Alt	AFR freq	AMR freq	ASN freq	EUR freq	SiPhy cons	Promoter histone marks	Enhancer histone marks	DNAse	Proteins bound	eQTL tissues	Motifs changed	GENCODE genes	dbSNP func annot
20	8808310	0.88	0.98	rs4816090	C	T	0.13	0.46	0.62	0.33							4 altered motifs	PLCB1	intronic
20	8808434	0.88	0.98	rs4816091	C	T	0.15	0.46	0.62	0.33							Foxj1	PLCB1	intronic
20	8810060	0.89	0.99	rs6077432	C	T	0.04	0.43	0.62	0.3				5 cell types	CTCF		Brachyury,Pou5f1	PLCB1	intronic
20	8810363	0.97	0.99	rs2223571	A	G	0.14	0.46	0.64	0.33							Bbx,HMG-IY,Nkx6-1	PLCB1	intronic
20	8814051	0.89	0.99	rs6140737	G	A	0.15	0.46	0.62	0.33							6 altered motifs	PLCB1	intronic
20	8816665	0.97	0.99	rs6086622	G	A	0.04	0.43	0.64	0.3			HSMM					PLCB1	intronic
20	8816728	0.98	0.99	rs6140738	G	T	0.04	0.43	0.64	0.3			HSMM					PLCB1	intronic
20	8817665	0.98	0.99	rs6077434	C	T	0.16	0.48	0.64	0.36			HSMM, NHLF					PLCB1	intronic
20	8818022	0.97	0.99	rs6086624	C	T	0.16	0.47	0.65	0.36			HSMM, NHLF				CEBPB	PLCB1	intronic
20	8818326	0.98	0.99	rs6086625	C	T	0.16	0.47	0.64	0.36			HSMM, NHLF	51 cell types			Pax-8	PLCB1	intronic
20	8818335	0.98	0.99	rs6086626	A	G	0.16	0.47	0.64	0.36			HSMM, NHLF	42 cell types			AP-1,ZID	PLCB1	intronic
20	8818667	0.98	0.99	rs6140739	T	A	0.16	0.47	0.64	0.36			HSMM				4 altered motifs	PLCB1	intronic
20	8818700	0.98	0.99	rs6140740	T	A	0.16	0.47	0.64	0.36			HSMM					PLCB1	intronic
20	8818864	0.98	0.99	rs6140741	T	C	0.16	0.47	0.64	0.36							Pou2f2	PLCB1	intronic
20	8819112	0.98	0.99	rs6140742	G	C	0.16	0.47	0.64	0.36							Foxa,Pou1f1,STAT	PLCB1	intronic
20	8819136	0.98	0.99	rs11698656	C	T	0.16	0.47	0.64	0.36							Evi-1,Osf2	PLCB1	intronic
20	8819200	0.98	0.99	rs11697064	T	C	0.16	0.47	0.64	0.36							DMRT1,DMRT4,Foxa	PLCB1	intronic
20	8819209	0.98	0.99	rs11697066	T	C	0.16	0.47	0.64	0.36							ERalpha-a,Foxa,Mef2	PLCB1	intronic
20	8819295	0.97	0.99	rs11698557	G	C	0.16	0.47	0.64	0.36								PLCB1	intronic
20	8819303	0.98	0.99	rs6077436	G	A	0.16	0.47	0.64	0.36							Irf,Osf2	PLCB1	intronic
20	8819311	0.98	0.99	rs11697086	T	C	0.04	0.43	0.64	0.3								PLCB1	intronic
20	8820056	0.97	0.99	rs6086629	T	A	0.16	0.47	0.65	0.36							Nkx2,STAT	PLCB1	intronic
20	8820280	1	1	rs2179475	C	G	0.16	0.47	0.64	0.36							Gfi1,HMG-IY	PLCB1	intronic
20	8820448	0.82	1	rs2143259	C	T	0.16	0.45	0.6	0.34							6 altered motifs	PLCB1	intronic
20	8820502	0.99	1	rs2143260	G	A	0.17	0.48	0.64	0.36							AP-1,ATF3	PLCB1	intronic
20	8820549	0.83	1	rs2143261	A	G	0.16	0.45	0.6	0.35							6 altered motifs	PLCB1	intronic
20	8820747	1	1	rs6086630	C	T	0.16	0.47	0.64	0.36							6 altered motifs	PLCB1	intronic
20	8821275	1	1	rs6140743	C	T	0.16	0.47	0.64	0.36							Irf,STAT,p300	PLCB1	intronic
20	8821334	1	1	rs6140744	C	T	0.16	0.47	0.64	0.36							Gfi1,HNF6,Sox	PLCB1	intronic

20	8821517	0.97	0.98	rs6140747	G	A	0.16	0.47	0.64	0.36							AP-4,HP1-site-factor,Zbtb3	PLCB1	intronic
20	8821577	0.98	1	rs6140748	C	T	0.16	0.48	0.65	0.36							4 altered motifs	PLCB1	intronic
20	8821621	0.98	1	rs6140750	A	G	0.16	0.47	0.65	0.35							5 altered motifs	PLCB1	intronic
20	8821695	0.8	0.99	rs6140751	A	G	0.15	0.43	0.59	0.33								PLCB1	intronic
20	8821846	1	1	rs2179476	T	A	0.16	0.47	0.64	0.36							6 altered motifs	PLCB1	intronic
20	8821969	1	1	rs2143262	T	C	0.16	0.47	0.64	0.36							Sox	PLCB1	intronic
20	8822431	1	1	rs12624809	G	C	0.16	0.47	0.64	0.36							E2F,Irf	PLCB1	intronic
20	8822723	1	1	rs6140753	G	A	0.16	0.47	0.64	0.36							10 altered motifs	PLCB1	intronic
20	8822931	1	1	rs6086632	T	C	0.16	0.47	0.64	0.36							Foxp1,HNF1	PLCB1	intronic
20	8823064	1	1	rs6086633	C	T	0.16	0.47	0.64	0.36							4 altered motifs	PLCB1	intronic
20	8823539	0.85	0.99	rs6140754	G	A	0.16	0.44	0.61	0.34							RXRA,ZEB1,p300	PLCB1	intronic
20	8824009	0.96	0.99	rs6133627	C	T	0.16	0.47	0.65	0.36							LBP-1	PLCB1	intronic
20	8824117	0.98	0.99	rs6140755	T	C	0.19	0.47	0.65	0.36								PLCB1	intronic
20	8824259	0.89	0.97	rs6140756	A	T	0.16	0.47	0.63	0.35							8 altered motifs	PLCB1	intronic
20	8824322	0.98	0.99	rs6086635	A	G	0.17	0.47	0.65	0.36							7 altered motifs	PLCB1	intronic
20	8824717	0.98	0.99	rs11087826	A	G	0.19	0.47	0.65	0.36			HepG2				HNF4,Mef2	PLCB1	intronic
20	8825193	0.98	0.99	rs6140758	C	T	0.04	0.43	0.65	0.3			HepG2				4 altered motifs	PLCB1	intronic
20	8825509	0.83	0.97	rs6140760	A	G	0.18	0.46	0.62	0.35							Ik-2,NRSF,Pitx2	PLCB1	intronic
20	8825748	0.97	0.98	rs6086640	A	G	0.19	0.47	0.64	0.35							Pax-5,ZID	PLCB1	intronic
20	8825793	0.97	0.98	rs6133628	C	T	0.15	0.47	0.64	0.35							4 altered motifs	PLCB1	intronic
20	8825801	0.97	0.98	rs6140761	T	C	0.18	0.47	0.64	0.35							HDAC2,Zbtb12	PLCB1	intronic
20	8826177	0.98	0.99	rs6077437	A	G	0.04	0.43	0.65	0.3							Pou5f1	PLCB1	intronic
20	8826360	0.98	0.99	rs6140764	G	A	0.19	0.47	0.65	0.36							Evi-1,Pou2f2	PLCB1	intronic
20	8826412	0.98	0.99	rs55875629	A	C	0.16	0.47	0.65	0.36							6 altered motifs	PLCB1	intronic
20	8826507	0.98	0.99	rs6077438	T	C	0.04	0.43	0.65	0.3							BRCA1,Cart1,Sox	PLCB1	intronic
20	8826512	0.98	0.99	rs6077439	A	G	0.16	0.47	0.65	0.35							Cart1,Nkx2,Sox	PLCB1	intronic
20	8826632	0.98	0.99	rs6077440	C	T	0.04	0.43	0.65	0.3							Hoxb13	PLCB1	intronic
20	8826987	0.98	0.99	rs6086641	A	T	0.16	0.47	0.65	0.36							HNF1,Pitx2	PLCB1	intronic
20	8827858	0.95	0.98	rs202130727	G	GAT	0.16	0.47	0.64	0.36							6 altered motifs	PLCB1	intronic
20	8827973	0.98	0.99	rs6086642	A	G	0.19	0.47	0.65	0.36							Hsf,Sin3Ak-20	PLCB1	intronic
20	8828487	0.96	0.98	rs6086643	G	T	0.04	0.43	0.64	0.3							4 altered motifs	PLCB1	intronic
20	8828492	0.96	0.98	rs6086644	G	A	0.06	0.43	0.64	0.3							6 altered motifs	PLCB1	intronic
20	8828502	0.97	0.98	rs6086645	T	C	0.19	0.47	0.64	0.35							HDAC2,Nanog,Sox	PLCB1	intronic
20	8828781	0.97	0.98	rs6140767	G	C	0.19	0.47	0.64	0.36				Melano			7 altered motifs	PLCB1	intronic
20	8829046	0.98	0.99	rs2876146	G	C	0.19	0.47	0.65	0.36							Nkx2	PLCB1	intronic
20	8829845	0.89	0.98	rs3902337	C	A	0.16	0.46	0.62	0.34							4 altered motifs	PLCB1	intronic
20	8829914	0.98	0.99	rs3902336	G	A	0.16	0.47	0.65	0.36								PLCB1	intronic
20	8830592	0.93	0.98	rs6140768	G	A	0.16	0.47	0.63	0.35							8 altered motifs	PLCB1	intronic
20	8831137	0.98	0.99	rs6039302	C	T	0.18	0.47	0.65	0.36							Foxp1	PLCB1	intronic
20	8831211	0.98	0.99	rs6056206	T	C	0.15	0.47	0.65	0.36							6 altered motifs	PLCB1	intronic
20	8831253	0.98	0.99	rs6039303	A	T	0.15	0.47	0.65	0.36							5 altered motifs	PLCB1	intronic
20	8831397	0.98	0.99	rs6056207	G	A	0.15	0.48	0.65	0.36							Hand1,NF-I,Smad3	PLCB1	intronic
20	8831501	0.98	0.99	rs4813873	G	A	0.16	0.47	0.65	0.36							Cdc5	PLCB1	intronic
20	8831744	0.98	0.99	rs4816093	A	T	0.16	0.48	0.65	0.36							7 altered motifs	PLCB1	intronic
20	8832767	0.98	0.99	rs6086647	C	T	0.19	0.47	0.65	0.36			HSMM, NHLF	5 cell types			4 altered motifs	PLCB1	intronic
20	8833132	0.98	0.99	rs4816094	G	A	0.18	0.47	0.65	0.36			HSMM				CDP	PLCB1	intronic
20	8833198	0.98	0.99	rs4816095	T	C	0.21	0.48	0.65	0.36			HSMM				GATA,Sox	PLCB1	intronic
20	8833231	0.98	0.99	rs4816096	T	C	0.18	0.47	0.65	0.36			HSMM				FXR,LXR	PLCB1	intronic
20	8833373	0.98	0.99	rs4816097	A	G	0.18	0.47	0.65	0.36			HSMM	Melano			Hoxa4	PLCB1	intronic
20	8833751	0.94	0.97	rs35030007	A	G	0.04	0.43	0.64	0.3			HSMM				4 altered motifs	PLCB1	intronic
20	8833755	0.95	0.98	rs11366851	CT	C	0.04	0.43	0.64	0.31			HSMM				6 altered motifs	PLCB1	intronic
20	8833905	0.97	0.98	rs4816098	G	A	0.17	0.47	0.64	0.36			HSMM				10 altered motifs	PLCB1	intronic
20	8834271	0.93	0.98	rs3926282	A	G	0.18	0.48	0.63	0.35			4 cell types				LBP-9	PLCB1	intronic
20	8834276	0.91	0.98	rs1967672	A	G	0.17	0.47	0.63	0.35			4 cell types				Hoxa3,Myc	PLCB1	intronic
20	8834367	0.95	0.98	rs4277595	A	G	0.2	0.47	0.64	0.35			4 cell types				4 altered motifs	PLCB1	intronic
20	8835779	0.96	0.98	rs6086649	A	T	0.04	0.41	0.64	0.29			HSMM, Huvec, NHLF				7 altered motifs	PLCB1	intronic
20	8837136	0.88	0.97	rs6133630	G	A	0.09	0.44	0.66	0.32			HSMM				6 altered motifs	PLCB1	intronic
20	8837162	0.88	0.97	rs6086650	G	A	0.09	0.44	0.66	0.32			HSMM				4 altered motifs	PLCB1	intronic
20	8837407	0.89	0.98	rs4426594	G	A	0.09	0.44	0.66	0.32							GR	PLCB1	intronic
20	8837909	0.87	0.95	rs6133632	G	T	0.04	0.41	0.65	0.3								PLCB1	intronic
20	8838220	0.89	0.98	rs6086652	C	T	0.1	0.44	0.66	0.32								PLCB1	intronic
20	8838343	0.89	0.98	rs6086653	A	G	0.04	0.41	0.66	0.3							Esr2,Spz1	PLCB1	intronic
20	8838376	0.89	0.98	rs6086654	C	T	0.09	0.44	0.66	0.32							ATF3,HEY1,Pdx1	PLCB1	intronic
20	8838914	0.89	0.98	rs6140771	C	T	0.09	0.44	0.66	0.32				8 cell types			CEBPA	PLCB1	intronic
20	8838963	0.89	0.98	rs6140772	A	C	0.1	0.44	0.66	0.32				6 cell types				PLCB1	intronic

20	8839222	0.89	0.98	rs11087827	A	T	0.1	0.44	0.66	0.32			HepG2, NHLF, HSMM	8 cell types	FOXA1,P300			PLCB1	intronic
20	8839408	0.89	0.98	rs13041928	G	A	0.09	0.44	0.66	0.32			NHLF, HSMM	HA-sp,RPTEC	FOXA1,FOXA2,P300			PLCB1	intronic
20	8839522	0.89	0.98	rs6140773	T	C	0.1	0.44	0.66	0.32			NHLF, HSMM					PLCB1	intronic
20	8839602	0.88	0.98	rs6077443	G	A	0.1	0.44	0.66	0.32			NHLF, HSMM				GR	PLCB1	intronic
20	8840064	0.89	0.98	rs6133634	C	G	0.1	0.44	0.66	0.32			HSMM				HMG-IY,Pou2f2,Pou5f1	PLCB1	intronic
20	8840154	0.89	0.98	rs6133635	C	A	0.09	0.44	0.66	0.32			HSMM	FibroP			Pax-6	PLCB1	intronic
20	8840293	0.89	0.98	rs6086657	T	C	0.09	0.44	0.66	0.32			HSMM				Hsf	PLCB1	intronic
20	8840670	0.89	0.98	rs4813874	A	T	0.11	0.44	0.66	0.32			HSMM, NHLF				7 altered motifs	PLCB1	intronic
20	8840702	0.89	0.98	rs4813875	A	C	0.1	0.44	0.66	0.32			HSMM, NHLF	AG04450			E2F	PLCB1	intronic
20	8840759	0.89	0.98	rs4813876	T	C	0.1	0.44	0.66	0.32			HSMM, NHLF	AG04450			Foxj2,Hoxa13,Maf	PLCB1	intronic
20	8840975	0.89	0.98	rs2066885	A	G	0.1	0.44	0.66	0.32			HSMM, NHLF	58 cell types	CFOS		5 altered motifs	PLCB1	intronic
20	8841005	0.89	0.98	rs2066884	A	G	0.1	0.44	0.66	0.32			HSMM, NHLF, HMEC	60 cell types	CFOS		5 altered motifs	PLCB1	intronic
20	8841105	0.89	0.98	rs2327117	G	A	0.09	0.44	0.66	0.32			HSMM, NHLF, HMEC	9 cell types	CFOS		5 altered motifs	PLCB1	intronic
20	8841183	0.89	0.98	rs2327118	G	A	0.1	0.44	0.66	0.32			HSMM, NHLF, HMEC					PLCB1	intronic
20	8842205	0.89	0.98	rs6133636	C	T	0.09	0.44	0.66	0.32							SZF1-1,Spz1,ZBTB33	PLCB1	intronic
20	8843256	0.89	0.98	rs6086660	T	G	0.1	0.44	0.66	0.32							4 altered motifs	PLCB1	intronic
20	8843306	0.89	0.98	rs6086661	C	T	0.1	0.45	0.66	0.32								PLCB1	intronic
20	8843353	0.89	0.98	rs6140778	A	G	0.1	0.44	0.66	0.32								PLCB1	intronic
20	8843845	0.87	0.96	rs6140779	A	G	0.1	0.44	0.66	0.32			H1				8 altered motifs	PLCB1	intronic
20	8844495	0.89	0.98	rs147271284	7-mer	A	0.14	0.44	0.66	0.32							8 altered motifs	PLCB1	intronic
20	8844694	0.89	0.98	rs6086662	T	C	0.15	0.44	0.66	0.32							DMRT2,Irf	PLCB1	intronic
20	8845519	0.89	0.98	rs6086664	T	A	0.08	0.44	0.66	0.32							LXR	PLCB1	intronic
20	8845933	0.85	0.94	rs6086665	G	A	0.09	0.44	0.65	0.32							12 altered motifs	PLCB1	intronic
20	8845937	0.85	0.94	rs6086666	G	A	0.16	0.44	0.65	0.31							Dmbx1	PLCB1	intronic
20	8846337	0.83	0.92	rs6140780	G	A	0.08	0.44	0.65	0.32							6 altered motifs	PLCB1	intronic
20	8846404	0.83	0.92	rs8182968	G	A	0.08	0.44	0.65	0.32							4 altered motifs	PLCB1	intronic
20	8846489	0.83	0.92	rs8182972	G	A	0.08	0.44	0.65	0.32							LBP-1	PLCB1	intronic
20	8846656	0.83	0.92	rs6086667	G	C	0.16	0.44	0.65	0.32							ATF3,STAT	PLCB1	intronic
20	8846739	0.83	0.92	rs6086668	A	G	0.16	0.44	0.65	0.32				Fibrobl			Dobox4,Mef2,STAT	PLCB1	intronic
20	8846835	0.83	0.92	rs6086669	G	T	0.16	0.44	0.65	0.32							7 altered motifs	PLCB1	intronic
20	8846925	0.83	0.92	rs6140781	G	A	0.16	0.44	0.65	0.31				FibroP				PLCB1	intronic
20	8846986	0.83	0.92	rs6086670	T	C	0.16	0.44	0.65	0.32				FibroP,HFF-Myc			CEBPB,Osf2	PLCB1	intronic
20	8847252	0.83	0.92	rs6077444	G	A	0.16	0.44	0.65	0.32							4 altered motifs	PLCB1	intronic
20	8847311	0.83	0.92	rs6086671	G	C	0.16	0.44	0.65	0.32							SEF-1	PLCB1	intronic
20	8847414	0.83	0.92	rs6086672	G	A	0.16	0.44	0.65	0.32			HSMM					PLCB1	intronic
20	8847727	0.83	0.92	rs3891453	A	G	0.16	0.44	0.65	0.32			HSMM					PLCB1	intronic
20	8848529	0.83	0.92	rs6077445	A	G	0.16	0.44	0.65	0.32							5 altered motifs	PLCB1	intronic
20	8848688	0.83	0.92	rs3915507	A	G	0.16	0.44	0.65	0.31							EBF	PLCB1	intronic
20	8848724	0.83	0.92	rs3848829	T	C	0.15	0.44	0.65	0.31							4 altered motifs	PLCB1	intronic
20	8848994	0.83	0.92	rs3848830	C	T	0.16	0.44	0.65	0.31							Sox	PLCB1	intronic
20	8849379	0.81	0.91	rs4816099	A	T	0.16	0.43	0.64	0.3							HNF4,Nkx2,TFE	PLCB1	intronic
20	8849390	0.83	0.92	rs4813878	C	T	0.16	0.44	0.65	0.3							SETDB1	PLCB1	intronic
20	8849910	0.83	0.92	rs2327119	C	A	0.16	0.44	0.65	0.31							Pou2f2,Zfp105	PLCB1	intronic
20	8851321	0.83	0.92	rs3848831	T	C	0.16	0.44	0.65	0.31							Foxc1,Maf,Pou2f2	PLCB1	intronic

Query SNP: rs6086632 and variants with r^2 >= 0.8

chr	pos (hg19)	LD (r^2)	LD (D')	variant	Ref	Alt	AFR freq	AMR freq	ASN freq	EUR freq	SiPhy cons	Promoter histone marks	Enhancer histone marks	DNAse	Proteins bound	eQTL tissues	Motifs changed	GENCODE genes	dbSNP func annot
20	8808310	0.88	0.98	rs4816090	C	T	0.13	0.46	0.62	0.33							4 altered motifs	PLCB1	intronic
20	8808434	0.88	0.98	rs4816091	C	T	0.15	0.46	0.62	0.33							Foxj1	PLCB1	intronic
20	8810060	0.89	0.99	rs6077432	C	T	0.04	0.43	0.62	0.3				5 cell types	CTCF		Brachyury,Pou5f1	PLCB1	intronic
20	8810363	0.97	0.99	rs2223571	A	G	0.14	0.46	0.64	0.33							Bbx,HMG-IY,Nkx6-1	PLCB1	intronic
20	8814051	0.89	0.99	rs6140737	G	A	0.15	0.46	0.62	0.33							6 altered motifs	PLCB1	intronic
20	8816665	0.97	0.99	rs6086622	G	A	0.04	0.43	0.64	0.3			HSMM					PLCB1	intronic
20	8816728	0.98	0.99	rs6140738	G	T	0.04	0.43	0.64	0.3			HSMM					PLCB1	intronic
20	8817665	0.98	0.99	rs6077434	C	T	0.16	0.48	0.64	0.36			HSMM, NHLF					PLCB1	intronic
20	8818022	0.97	0.99	rs6086624	C	T	0.16	0.47	0.65	0.36			HSMM, NHLF				CEBPB	PLCB1	intronic
20	8818326	0.98	0.99	rs6086625	C	T	0.16	0.47	0.64	0.36			HSMM, NHLF	51 cell types			Pax-8	PLCB1	intronic
20	8818335	0.98	0.99	rs6086626	A	G	0.16	0.47	0.64	0.36			HSMM, NHLF	42 cell types			AP-1,ZID	PLCB1	intronic
20	8818667	0.98	0.99	rs6140739	T	A	0.16	0.47	0.64	0.36			HSMM				4 altered motifs	PLCB1	intronic
20	8818700	0.98	0.99	rs6140740	T	A	0.16	0.47	0.64	0.36			HSMM					PLCB1	intronic
20	8818864	0.98	0.99	rs6140741	T	C	0.16	0.47	0.64	0.36							Pou2f2	PLCB1	intronic
20	8819112	0.98	0.99	rs6140742	G	C	0.16	0.47	0.64	0.36							Foxa,Pou1f1,STAT	PLCB1	intronic
20	8819136	0.98	0.99	rs11698656	C	T	0.16	0.47	0.64	0.36							Evi-1,Osf2	PLCB1	intronic
20	8819200	0.98	0.99	rs11697064	T	C	0.16	0.47	0.64	0.36							DMRT1,DMRT4,Foxa	PLCB1	intronic

20	8819209	0.98	0.99	rs11697066	T	C	0.16	0.47	0.64	0.36							ERalpha-a,Foxa,Mef2	PLCB1	intronic
20	8819295	0.97	0.99	rs11698557	G	C	0.16	0.47	0.64	0.36								PLCB1	intronic
20	8819303	0.98	0.99	rs6077436	G	A	0.16	0.47	0.64	0.36							Irf,Osf2	PLCB1	intronic
20	8819311	0.98	0.99	rs11697086	T	C	0.04	0.43	0.64	0.3								PLCB1	intronic
20	8820056	0.97	0.99	rs6086629	T	A	0.16	0.47	0.65	0.36							Nkx2,STAT	PLCB1	intronic
20	8820280	1	1	rs2179475	C	G	0.16	0.47	0.64	0.36							Gfi1,HMG-IY	PLCB1	intronic
20	8820448	0.82	1	rs2143259	C	T	0.16	0.45	0.6	0.34							6 altered motifs	PLCB1	intronic
20	8820502	0.99	1	rs2143260	G	A	0.17	0.48	0.64	0.36							AP-1,ATF3	PLCB1	intronic
20	8820549	0.83	1	rs2143261	A	G	0.16	0.45	0.6	0.35							6 altered motifs	PLCB1	intronic
20	8820747	1	1	rs6086630	C	T	0.16	0.47	0.64	0.36							6 altered motifs	PLCB1	intronic
20	8821275	1	1	rs6140743	C	T	0.16	0.47	0.64	0.36							Irf,STAT,p300	PLCB1	intronic
20	8821334	1	1	rs6140744	C	T	0.16	0.47	0.64	0.36							Gfi1,HNF6,Sox	PLCB1	intronic
20	8821517	0.97	0.98	rs6140747	G	A	0.16	0.47	0.64	0.36							AP-4,HP1-site-[illegible]	PLCB1	intronic
20	8821577	0.98	1	rs6140748	C	T	0.16	0.48	0.65	0.36							4 altered motifs	PLCB1	intronic
20	8821621	0.98	1	rs6140750	A	G	0.16	0.47	0.65	0.35							5 altered motifs	PLCB1	intronic
20	8821695	0.8	0.99	rs6140751	A	G	0.15	0.43	0.59	0.33								PLCB1	intronic
20	8821846	1	1	rs2179476	T	A	0.16	0.47	0.64	0.36							6 altered motifs	PLCB1	intronic
20	8821969	1	1	rs2143262	T	C	0.16	0.47	0.64	0.36							Sox	PLCB1	intronic
20	8822431	1	1	rs12624809	G	C	0.16	0.47	0.64	0.36							E2F,Irf	PLCB1	intronic
20	8822723	1	1	rs6140753	G	A	0.16	0.47	0.64	0.36							10 altered motifs	PLCB1	intronic
20	8822931	1	1	rs6086632	T	C	0.16	0.47	0.64	0.36							Foxp1,HNF1	PLCB1	intronic
20	8823064	1	1	rs6086633	C	T	0.16	0.47	0.64	0.36							4 altered motifs	PLCB1	intronic
20	8823539	0.85	0.99	rs6140754	G	A	0.16	0.44	0.61	0.34							RXRA,ZEB1,p300	PLCB1	intronic
20	8824009	0.96	0.99	rs6133627	C	T	0.16	0.47	0.65	0.36							LBP-1	PLCB1	intronic
20	8824117	0.98	0.99	rs6140755	T	C	0.19	0.47	0.65	0.36								PLCB1	intronic
20	8824259	0.89	0.97	rs6140756	A	T	0.16	0.47	0.63	0.35							8 altered motifs	PLCB1	intronic
20	8824322	0.98	0.99	rs6086635	A	G	0.17	0.47	0.65	0.36							7 altered motifs	PLCB1	intronic
20	8824717	0.98	0.99	rs11087826	A	G	0.19	0.47	0.65	0.36			HepG2				HNF4,Mef2	PLCB1	intronic
20	8825193	0.98	0.99	rs6140758	C	T	0.04	0.43	0.65	0.3			HepG2				4 altered motifs	PLCB1	intronic
20	8825509	0.83	0.97	rs6140760	A	G	0.18	0.46	0.62	0.35							Ik-2,NRSF,Pitx[illegible]	PLCB1	intronic
20	8825748	0.97	0.98	rs6086640	A	G	0.19	0.47	0.64	0.35							Pax-5,ZID	PLCB1	intronic
20	8825793	0.97	0.98	rs6133628	C	T	0.15	0.47	0.64	0.35							4 altered motifs	PLCB1	intronic
20	8825801	0.97	0.98	rs6140761	T	C	0.18	0.47	0.64	0.35							HDAC2,Zbtb12	PLCB1	intronic
20	8826177	0.98	0.99	rs6077437	A	G	0.04	0.43	0.65	0.3							Pou5f1	PLCB1	intronic
20	8826360	0.98	0.99	rs6140764	G	A	0.19	0.47	0.65	0.36							Evi-1,Pou2f2	PLCB1	intronic
20	8826412	0.98	0.99	rs55875629	A	C	0.16	0.47	0.65	0.36							6 altered motifs	PLCB1	intronic
20	8826507	0.98	0.99	rs6077438	T	C	0.04	0.43	0.65	0.3							BRCA1,Cart1,Sox	PLCB1	intronic
20	8826512	0.98	0.99	rs6077439	A	G	0.16	0.47	0.65	0.35							Cart1,Nkx2,Sox	PLCB1	intronic
20	8826632	0.98	0.99	rs6077440	C	T	0.04	0.43	0.65	0.3							Hoxb13	PLCB1	intronic
20	8826987	0.98	0.99	rs6086641	A	T	0.16	0.47	0.65	0.36							HNF1,Pitx2	PLCB1	intronic
20	8827858	0.95	0.98	rs202130727	G	GAT	0.16	0.47	0.64	0.36							6 altered motifs	PLCB1	intronic
20	8827973	0.98	0.99	rs6086642	A	G	0.19	0.47	0.65	0.36							Hsf,Sin3Ak-20	PLCB1	intronic
20	8828487	0.96	0.98	rs6086643	G	T	0.04	0.43	0.64	0.3							4 altered motifs	PLCB1	intronic
20	8828492	0.96	0.98	rs6086644	G	A	0.06	0.43	0.64	0.3							6 altered motifs	PLCB1	intronic
20	8828502	0.97	0.98	rs6086645	T	C	0.19	0.47	0.64	0.35							HDAC2,Nanog,Sox	PLCB1	intronic
20	8828781	0.97	0.98	rs6140767	G	C	0.19	0.47	0.64	0.36				Melano			7 altered motifs	PLCB1	intronic
20	8829046	0.98	0.99	rs2876146	G	C	0.19	0.47	0.65	0.36							Nkx2	PLCB1	intronic
20	8829845	0.89	0.98	rs3902337	C	A	0.16	0.46	0.62	0.34							4 altered motifs	PLCB1	intronic
20	8829914	0.98	0.99	rs3902336	G	A	0.16	0.47	0.65	0.36								PLCB1	intronic
20	8830592	0.93	0.98	rs6140768	G	A	0.16	0.47	0.63	0.35							8 altered motifs	PLCB1	intronic
20	8831137	0.98	0.99	rs6039302	C	T	0.18	0.47	0.65	0.36							Foxp1	PLCB1	intronic
20	8831211	0.98	0.99	rs6056206	T	C	0.15	0.47	0.65	0.36							6 altered motifs	PLCB1	intronic
20	8831253	0.98	0.99	rs6039303	A	T	0.15	0.47	0.65	0.36							5 altered motifs	PLCB1	intronic
20	8831397	0.98	0.99	rs6056207	G	A	0.15	0.48	0.65	0.36							Hand1,NF-I,Smad3	PLCB1	intronic
20	8831501	0.98	0.99	rs4813873	G	A	0.16	0.47	0.65	0.36							Cdc5	PLCB1	intronic
20	8831744	0.98	0.99	rs4816093	A	T	0.16	0.48	0.65	0.36							7 altered motifs	PLCB1	intronic
20	8832767	0.98	0.99	rs6086647	C	T	0.19	0.47	0.65	0.36			HSMM, NHLF	5 cell types			4 altered motifs	PLCB1	intronic
20	8833132	0.98	0.99	rs4816094	G	A	0.18	0.47	0.65	0.36			HSMM				CDP	PLCB1	intronic
20	8833198	0.98	0.99	rs4816095	T	C	0.21	0.48	0.65	0.36			HSMM				GATA,Sox	PLCB1	intronic
20	8833231	0.98	0.99	rs4816096	T	C	0.18	0.47	0.65	0.36			HSMM				FXR,LXR	PLCB1	intronic
20	8833373	0.98	0.99	rs4816097	A	G	0.18	0.47	0.65	0.36			HSMM	Melano			Hoxa4	PLCB1	intronic
20	8833751	0.94	0.97	rs35030007	A	G	0.04	0.43	0.64	0.3			HSMM				4 altered motifs	PLCB1	intronic
20	8833755	0.95	0.98	rs11366851	CT	C	0.04	0.43	0.64	0.31			HSMM				6 altered motifs	PLCB1	intronic
20	8833905	0.97	0.98	rs4816098	G	A	0.17	0.47	0.64	0.36			HSMM				10 altered motifs	PLCB1	intronic
20	8834271	0.93	0.98	rs3926282	A	G	0.18	0.48	0.63	0.35			4 cell types				LBP-9	PLCB1	intronic

20	8834276	0.91	0.98	rs1967672	A	G	0.17	0.47	0.63	0.35			4 cell types				Hoxa3,Myc	PLCB1	intronic
20	8834367	0.95	0.98	rs4277595	A	G	0.2	0.47	0.64	0.35			4 cell types				4 altered motifs	PLCB1	intronic
20	8835779	0.96	0.98	rs6086649	A	T	0.04	0.41	0.64	0.29			HSMM, Huvec, NHLF				7 altered motifs	PLCB1	intronic
20	8837136	0.88	0.97	rs6133630	G	A	0.09	0.44	0.66	0.32			HSMM				6 altered motifs	PLCB1	intronic
20	8837162	0.88	0.97	rs6086650	G	A	0.09	0.44	0.66	0.32			HSMM				4 altered motifs	PLCB1	intronic
20	8837407	0.89	0.98	rs4426594	G	A	0.09	0.44	0.66	0.32							GR	PLCB1	intronic
20	8837909	0.87	0.95	rs6133632	G	T	0.04	0.41	0.65	0.3								PLCB1	intronic
20	8838220	0.89	0.98	rs6086652	C	T	0.1	0.44	0.66	0.32								PLCB1	intronic
20	8838343	0.89	0.98	rs6086653	A	G	0.04	0.41	0.66	0.3							Esr2,Spz1	PLCB1	intronic
20	8838376	0.89	0.98	rs6086654	C	T	0.09	0.44	0.66	0.32							ATF3,HEY1, Pdx1	PLCB1	intronic
20	8838914	0.89	0.98	rs6140771	C	T	0.09	0.44	0.66	0.32				8 cell types			CEBPA	PLCB1	intronic
20	8838963	0.89	0.98	rs6140772	A	C	0.1	0.44	0.66	0.32				6 cell types				PLCB1	intronic
20	8839222	0.89	0.98	rs11087827	A	T	0.1	0.44	0.66	0.32			HepG2, NHLF, HSMM	8 cell types	FOXA1,P300			PLCB1	intronic
20	8839408	0.89	0.98	rs13041928	G	A	0.09	0.44	0.66	0.32			NHLF, HSMM	HA-sp,RPTEC	FOXA1,FOXA2,P300			PLCB1	intronic
20	8839522	0.89	0.98	rs6140773	T	C	0.1	0.44	0.66	0.32			NHLF, HSMM					PLCB1	intronic
20	8839602	0.88	0.98	rs6077443	G	A	0.1	0.44	0.66	0.32			NHLF, HSMM				GR	PLCB1	intronic
20	8840064	0.89	0.98	rs6133634	C	G	0.1	0.44	0.66	0.32			HSMM				HMG-IY,Pou2f2,Pou5f1	PLCB1	intronic
20	8840154	0.89	0.98	rs6133635	C	A	0.09	0.44	0.66	0.32			HSMM	FibroP			Pax-6	PLCB1	intronic
20	8840293	0.89	0.98	rs6086657	T	C	0.09	0.44	0.66	0.32			HSMM				Hsf	PLCB1	intronic
20	8840670	0.89	0.98	rs4813874	A	T	0.11	0.44	0.66	0.32			HSMM, NHLF				7 altered motifs	PLCB1	intronic
20	8840702	0.89	0.98	rs4813875	A	C	0.1	0.44	0.66	0.32			HSMM, NHLF	AG04450			E2F	PLCB1	intronic
20	8840759	0.89	0.98	rs4813876	T	C	0.1	0.44	0.66	0.32			HSMM, NHLF	AG04450			Foxj2,Hoxa13,Maf	PLCB1	intronic
20	8840975	0.89	0.98	rs2066885	A	G	0.1	0.44	0.66	0.32			HSMM, NHLF	58 cell types	CFOS		5 altered motifs	PLCB1	intronic
20	8841005	0.89	0.98	rs2066884	A	G	0.1	0.44	0.66	0.32			HSMM, NHLF, HMEC	60 cell types	CFOS		5 altered motifs	PLCB1	intronic
20	8841105	0.89	0.98	rs2327117	G	A	0.09	0.44	0.66	0.32			HSMM, NHLF, HMEC	9 cell types	CFOS		5 altered motifs	PLCB1	intronic
20	8841183	0.89	0.98	rs2327118	G	A	0.1	0.44	0.66	0.32			HSMM, NHLF, HMEC					PLCB1	intronic
20	8842205	0.89	0.98	rs6133636	C	T	0.09	0.44	0.66	0.32							SZF1-1,Spz1,ZBTB33	PLCB1	intronic
20	8843256	0.89	0.98	rs6086660	T	G	0.1	0.44	0.66	0.32							4 altered motifs	PLCB1	intronic
20	8843306	0.89	0.98	rs6086661	C	T	0.1	0.45	0.66	0.32								PLCB1	intronic
20	8843353	0.89	0.98	rs6140778	A	G	0.1	0.44	0.66	0.32								PLCB1	intronic
20	8843845	0.87	0.96	rs6140779	A	G	0.1	0.44	0.66	0.32			H1				8 altered motifs	PLCB1	intronic
20	8844495	0.89	0.98	rs147271284	7-mer	A	0.14	0.44	0.66	0.32							8 altered motifs	PLCB1	intronic
20	8844694	0.89	0.98	rs6086662	T	C	0.15	0.44	0.66	0.32							DMRT2,Irf	PLCB1	intronic
20	8845519	0.89	0.98	rs6086664	T	A	0.08	0.44	0.66	0.32							LXR	PLCB1	intronic
20	8845933	0.85	0.94	rs6086665	G	A	0.09	0.44	0.65	0.32							12 altered motifs	PLCB1	intronic
20	8845937	0.85	0.94	rs6086666	G	A	0.16	0.44	0.65	0.31							Dmbx1	PLCB1	intronic
20	8846337	0.83	0.92	rs6140780	G	A	0.08	0.44	0.65	0.32							6 altered motifs	PLCB1	intronic
20	8846404	0.83	0.92	rs8182968	G	A	0.08	0.44	0.65	0.32							4 altered motifs	PLCB1	intronic
20	8846489	0.83	0.92	rs8182972	G	A	0.08	0.44	0.65	0.32							LBP-1	PLCB1	intronic
20	8846656	0.83	0.92	rs6086667	G	C	0.16	0.44	0.65	0.32							ATF3,STAT	PLCB1	intronic
20	8846739	0.83	0.92	rs6086668	A	G	0.16	0.44	0.65	0.32				Fibrobl			Dobox4,Mef2,STAT	PLCB1	intronic
20	8846835	0.83	0.92	rs6086669	G	T	0.16	0.44	0.65	0.32							7 altered motifs	PLCB1	intronic
20	8846925	0.83	0.92	rs6140781	G	A	0.16	0.44	0.65	0.31				FibroP				PLCB1	intronic
20	8846986	0.83	0.92	rs6086670	T	C	0.16	0.44	0.65	0.32				FibroP,HFF-Myc			CEBPB,Osf2	PLCB1	intronic
20	8847252	0.83	0.92	rs6077444	G	A	0.16	0.44	0.65	0.32							4 altered motifs	PLCB1	intronic
20	8847311	0.83	0.92	rs6086671	G	C	0.16	0.44	0.65	0.32							SEF-1	PLCB1	intronic
20	8847414	0.83	0.92	rs6086672	G	A	0.16	0.44	0.65	0.32			HSMM					PLCB1	intronic
20	8847727	0.83	0.92	rs3891453	A	G	0.16	0.44	0.65	0.32			HSMM					PLCB1	intronic
20	8848529	0.83	0.92	rs6077445	A	G	0.16	0.44	0.65	0.32							5 altered motifs	PLCB1	intronic
20	8848688	0.83	0.92	rs3915507	A	G	0.16	0.44	0.65	0.31							EBF	PLCB1	intronic
20	8848724	0.83	0.92	rs3848829	T	C	0.15	0.44	0.65	0.31							4 altered motifs	PLCB1	intronic
20	8848994	0.83	0.92	rs3848830	C	T	0.16	0.44	0.65	0.31							Sox	PLCB1	intronic
20	8849379	0.81	0.91	rs4816099	A	T	0.16	0.43	0.64	0.3							HNF4,Nkx2, TFE	PLCB1	intronic
20	8849390	0.83	0.92	rs4813878	C	T	0.16	0.44	0.65	0.3							SETDB1	PLCB1	intronic
20	8849910	0.83	0.92	rs2327119	C	A	0.16	0.44	0.65	0.31							Pou2f2,Zfp105	PLCB1	intronic
20	8851321	0.83	0.92	rs3848831	T	C	0.16	0.44	0.65	0.31							Foxc1,Maf,Pou2f2	PLCB1	intronic

Query SNP: rs6086633 and variants with r^2 >= 0.8

chr	pos (hg19)	LD (r²)	LD (D')	variant	Ref	Alt	AFR freq	AMR freq	ASN freq	EUR freq	SiPhy cons	Promoter histone marks	Enhancer histone marks	DNAse	Proteins bound	eQTL tissues	Motifs changed	GENCODE genes	dbSNP func annot
20	8808310	0.88	0.98	rs4816090	C	T	0.13	0.46	0.62	0.33							4 altered motifs	PLCB1	intronic
20	8808434	0.88	0.98	rs4816091	C	T	0.15	0.46	0.62	0.33							Foxj1	PLCB1	intronic
20	8810060	0.89	0.99	rs6077432	C	T	0.04	0.43	0.62	0.3				5 cell types	CTCF		Brachyury,Pou5f1	PLCB1	intronic

20	8810363	0.97	0.99	rs2223571	A	G	0.14	0.46	0.64	0.33							Bbx,HMG-IY,Nkx6-1	PLCB1	intronic
20	8814051	0.89	0.99	rs6140737	G	A	0.15	0.46	0.62	0.33							6 altered motifs	PLCB1	intronic
20	8816665	0.97	0.99	rs6086622	G	A	0.04	0.43	0.64	0.3			HSMM					PLCB1	intronic
20	8816728	0.98	0.99	rs6140738	G	T	0.04	0.43	0.64	0.3			HSMM					PLCB1	intronic
20	8817665	0.98	0.99	rs6077434	C	T	0.16	0.48	0.64	0.36			HSMM, NHLF					PLCB1	intronic
20	8818022	0.97	0.99	rs6086624	C	T	0.16	0.47	0.65	0.36			HSMM, NHLF				CEBPB	PLCB1	intronic
20	8818326	0.98	0.99	rs6086625	C	T	0.16	0.47	0.64	0.36			HSMM, NHLF	51 cell types			Pax-8	PLCB1	intronic
20	8818335	0.98	0.99	rs6086626	A	G	0.16	0.47	0.64	0.36			HSMM, NHLF	42 cell types			AP-1,ZID	PLCB1	intronic
20	8818667	0.98	0.99	rs6140739	T	A	0.16	0.47	0.64	0.36			HSMM				4 altered motifs	PLCB1	intronic
20	8818700	0.98	0.99	rs6140740	T	A	0.16	0.47	0.64	0.36			HSMM					PLCB1	intronic
20	8818864	0.98	0.99	rs6140741	T	C	0.16	0.47	0.64	0.36							Pou2f2	PLCB1	intronic
20	8819112	0.98	0.99	rs6140742	G	C	0.16	0.47	0.64	0.36							Foxa,Pou1f1,STAT	PLCB1	intronic
20	8819136	0.98	0.99	rs11698656	C	T	0.16	0.47	0.64	0.36							Evi-1,Osf2	PLCB1	intronic
20	8819200	0.98	0.99	rs11697064	T	C	0.16	0.47	0.64	0.36							DMRT1,DMRT4,Foxa	PLCB1	intronic
20	8819209	0.98	0.99	rs11697066	T	C	0.16	0.47	0.64	0.36							ERalpha-a,Foxa,Mef2	PLCB1	intronic
20	8819295	0.97	0.99	rs11698557	G	C	0.16	0.47	0.64	0.36								PLCB1	intronic
20	8819303	0.98	0.99	rs6077436	G	A	0.16	0.47	0.64	0.36							Irf,Osf2	PLCB1	intronic
20	8819311	0.98	0.99	rs11697086	T	C	0.04	0.43	0.64	0.3								PLCB1	intronic
20	8820056	0.97	0.99	rs6086629	T	A	0.16	0.47	0.65	0.36							Nkx2,STAT	PLCB1	intronic
20	8820280	1	1	rs2179475	C	G	0.16	0.47	0.64	0.36							Gfi1,HMG-IY	PLCB1	intronic
20	8820448	0.82	1	rs2143259	C	T	0.16	0.45	0.6	0.34							6 altered motifs	PLCB1	intronic
20	8820502	0.99	1	rs2143260	G	A	0.17	0.48	0.64	0.36							AP-1,ATF3	PLCB1	intronic
20	8820549	0.83	1	rs2143261	A	G	0.16	0.45	0.6	0.35							6 altered motifs	PLCB1	intronic
20	8820747	1	1	rs6086630	C	T	0.16	0.47	0.64	0.36							6 altered motifs	PLCB1	intronic
20	8821275	1	1	rs6140743	C	T	0.16	0.47	0.64	0.36							Irf,STAT,p300	PLCB1	intronic
20	8821334	1	1	rs6140744	C	T	0.16	0.47	0.64	0.36							Gfi1,HNF6,Sox	PLCB1	intronic
20	8821517	0.97	0.98	rs6140747	G	A	0.16	0.47	0.64	0.36							AP-4,HP1-site-factor,Zbtb3	PLCB1	intronic
20	8821577	0.98	1	rs6140748	C	T	0.16	0.48	0.65	0.36							4 altered motifs	PLCB1	intronic
20	8821621	0.98	1	rs6140750	A	G	0.16	0.47	0.65	0.35							5 altered motifs	PLCB1	intronic
20	8821695	0.8	0.99	rs6140751	A	G	0.15	0.43	0.59	0.33								PLCB1	intronic
20	8821846	1	1	rs2179476	T	A	0.16	0.47	0.64	0.36							6 altered motifs	PLCB1	intronic
20	8821969	1	1	rs2143262	T	C	0.16	0.47	0.64	0.36							Sox	PLCB1	intronic
20	8822431	1	1	rs12624809	G	C	0.16	0.47	0.64	0.36							E2F,Irf	PLCB1	intronic
20	8822723	1	1	rs6140753	G	A	0.16	0.47	0.64	0.36							10 altered motifs	PLCB1	intronic
20	8822931	1	1	rs6086632	T	C	0.16	0.47	0.64	0.36							Foxp1,HNF1	PLCB1	intronic
20	8823064	1	1	rs6086633	C	T	0.16	0.47	0.64	0.36							4 altered motifs	PLCB1	intronic
20	8823539	0.85	0.99	rs6140754	G	A	0.16	0.44	0.61	0.34							RXRA,ZEB1,p300	PLCB1	intronic
20	8824009	0.96	0.99	rs6133627	C	T	0.16	0.47	0.65	0.36							LBP-1	PLCB1	intronic
20	8824117	0.98	0.99	rs6140755	T	C	0.19	0.47	0.65	0.36								PLCB1	intronic
20	8824259	0.89	0.97	rs6140756	A	T	0.16	0.47	0.63	0.35							8 altered motifs	PLCB1	intronic
20	8824322	0.98	0.99	rs6086635	A	G	0.17	0.47	0.65	0.36							7 altered motifs	PLCB1	intronic
20	8824717	0.98	0.99	rs11087826	A	G	0.19	0.47	0.65	0.36			HepG2				HNF4,Mef2	PLCB1	intronic
20	8825193	0.98	0.99	rs6140758	C	T	0.04	0.43	0.65	0.3			HepG2				4 altered motifs	PLCB1	intronic
20	8825509	0.83	0.97	rs6140760	A	G	0.18	0.46	0.62	0.35							Ik-2,NRSF,Pitx2	PLCB1	intronic
20	8825748	0.97	0.98	rs6086640	A	G	0.19	0.47	0.64	0.35							Pax-5,ZID	PLCB1	intronic
20	8825793	0.97	0.98	rs6133628	C	T	0.15	0.47	0.64	0.35							4 altered motifs	PLCB1	intronic
20	8825801	0.97	0.98	rs6140761	T	C	0.18	0.47	0.64	0.35							HDAC2,Zbtb12	PLCB1	intronic
20	8826177	0.98	0.99	rs6077437	A	G	0.04	0.43	0.65	0.3							Pou5f1	PLCB1	intronic
20	8826360	0.98	0.99	rs6140764	G	A	0.19	0.47	0.65	0.36							Evi-1,Pou2f2	PLCB1	intronic
20	8826412	0.98	0.99	rs55875629	A	C	0.16	0.47	0.65	0.36							6 altered motifs	PLCB1	intronic
20	8826507	0.98	0.99	rs6077438	T	C	0.04	0.43	0.65	0.3							BRCA1,Cart1,Sox	PLCB1	intronic
20	8826512	0.98	0.99	rs6077439	A	G	0.16	0.47	0.65	0.35							Cart1,Nkx2,Sox	PLCB1	intronic
20	8826632	0.98	0.99	rs6077440	C	T	0.04	0.43	0.65	0.3							Hoxb13	PLCB1	intronic
20	8826987	0.98	0.99	rs6086641	A	T	0.16	0.47	0.65	0.36							HNF1,Pitx2	PLCB1	intronic
20	8827858	0.95	0.98	rs202130727	G	GAT	0.16	0.47	0.64	0.36							6 altered motifs	PLCB1	intronic
20	8827973	0.98	0.99	rs6086642	A	G	0.19	0.47	0.65	0.36							Hsf,Sin3Ak-20	PLCB1	intronic
20	8828487	0.96	0.98	rs6086643	G	T	0.04	0.43	0.64	0.3							4 altered motifs	PLCB1	intronic
20	8828492	0.96	0.98	rs6086644	G	A	0.06	0.43	0.64	0.3							6 altered motifs	PLCB1	intronic
20	8828502	0.97	0.98	rs6086645	T	C	0.19	0.47	0.64	0.35							HDAC2,Nanog,Sox	PLCB1	intronic
20	8828781	0.97	0.98	rs6140767	G	C	0.19	0.47	0.64	0.36				Melano			7 altered motifs	PLCB1	intronic
20	8829046	0.98	0.99	rs2876146	G	C	0.19	0.47	0.65	0.36							Nkx2	PLCB1	intronic
20	8829845	0.89	0.98	rs3902337	C	A	0.16	0.46	0.62	0.34							4 altered motifs	PLCB1	intronic
20	8829914	0.98	0.99	rs3902336	G	A	0.16	0.47	0.65	0.36								PLCB1	intronic
20	8830592	0.93	0.98	rs6140768	G	A	0.16	0.47	0.63	0.35							8 altered motifs	PLCB1	intronic
20	8831137	0.98	0.99	rs6039302	C	T	0.18	0.47	0.65	0.36							Foxp1	PLCB1	intronic

20	8831211	0.98	0.99	rs6056206	T	C	0.15	0.47	0.65	0.36							6 altered motifs	PLCB1	intronic
20	8831253	0.98	0.99	rs6039303	A	T	0.15	0.47	0.65	0.36							5 altered motifs	PLCB1	intronic
20	8831397	0.98	0.99	rs6056207	G	A	0.15	0.48	0.65	0.36							Hand1,NF-I,Smad3	PLCB1	intronic
20	8831501	0.98	0.99	rs4813873	G	A	0.16	0.47	0.65	0.36							Cdc5	PLCB1	intronic
20	8831744	0.98	0.99	rs4816093	A	T	0.16	0.48	0.65	0.36							7 altered motifs	PLCB1	intronic
20	8832767	0.98	0.99	rs6086647	C	T	0.19	0.47	0.65	0.36			HSMM, NHLF	5 cell types			4 altered motifs	PLCB1	intronic
20	8833132	0.98	0.99	rs4816094	G	A	0.18	0.47	0.65	0.36			HSMM				CDP	PLCB1	intronic
20	8833198	0.98	0.99	rs4816095	T	C	0.21	0.48	0.65	0.36			HSMM				GATA,Sox	PLCB1	intronic
20	8833231	0.98	0.99	rs4816096	T	C	0.18	0.47	0.65	0.36			HSMM				FXR,LXR	PLCB1	intronic
20	8833373	0.98	0.99	rs4816097	A	G	0.18	0.47	0.65	0.36			HSMM	Melano			Hoxa4	PLCB1	intronic
20	8833751	0.94	0.97	rs35030007	A	G	0.04	0.43	0.64	0.3			HSMM				4 altered motifs	PLCB1	intronic
20	8833755	0.95	0.98	rs11366851	CT	C	0.04	0.43	0.64	0.31			HSMM				6 altered motifs	PLCB1	intronic
20	8833905	0.97	0.98	rs4816098	G	A	0.17	0.47	0.64	0.36			HSMM				10 altered motifs	PLCB1	intronic
20	8834271	0.93	0.98	rs3926282	A	G	0.18	0.48	0.63	0.35			4 cell types				LBP-9	PLCB1	intronic
20	8834276	0.91	0.98	rs1967672	A	G	0.17	0.47	0.63	0.35			4 cell types				Hoxa3,Myc	PLCB1	intronic
20	8834367	0.95	0.98	rs4277595	A	G	0.2	0.47	0.64	0.35			4 cell types				4 altered motifs	PLCB1	intronic
20	8835779	0.96	0.98	rs6086649	A	T	0.04	0.41	0.64	0.29			HSMM, Huvec, NHLF				7 altered motifs	PLCB1	intronic
20	8837136	0.88	0.97	rs6133630	G	A	0.09	0.44	0.66	0.32			HSMM				6 altered motifs	PLCB1	intronic
20	8837162	0.88	0.97	rs6086650	G	A	0.09	0.44	0.66	0.32			HSMM				4 altered motifs	PLCB1	intronic
20	8837407	0.89	0.98	rs4426594	G	A	0.09	0.44	0.66	0.32							GR	PLCB1	intronic
20	8837909	0.87	0.95	rs6133632	G	T	0.04	0.41	0.65	0.3								PLCB1	intronic
20	8838220	0.89	0.98	rs6086652	C	T	0.1	0.44	0.66	0.32								PLCB1	intronic
20	8838343	0.89	0.98	rs6086653	A	G	0.04	0.41	0.66	0.3							Esr2,Spz1	PLCB1	intronic
20	8838376	0.89	0.98	rs6086654	C	T	0.09	0.44	0.66	0.32							ATF3,HEY1,Pdx1	PLCB1	intronic
20	8838914	0.89	0.98	rs6140771	C	T	0.09	0.44	0.66	0.32				8 cell types			CEBPA	PLCB1	intronic
20	8838963	0.89	0.98	rs6140772	A	C	0.1	0.44	0.66	0.32				6 cell types				PLCB1	intronic
20	8839222	0.89	0.98	rs11087827	A	T	0.1	0.44	0.66	0.32			HepG2, NHLF, HSMM	8 cell types	FOXA1,P300			PLCB1	intronic
20	8839408	0.89	0.98	rs13041928	G	A	0.09	0.44	0.66	0.32			NHLF, HSMM	HA-sp,RPTEC	FOXA1,FOXA2,P300			PLCB1	intronic
20	8839522	0.89	0.98	rs6140773	T	C	0.1	0.44	0.66	0.32			NHLF, HSMM					PLCB1	intronic
20	8839602	0.88	0.98	rs6077443	G	A	0.1	0.44	0.66	0.32			NHLF, HSMM				GR	PLCB1	intronic
20	8840064	0.89	0.98	rs6133634	C	G	0.1	0.44	0.66	0.32			HSMM				HMG-IY,Pou2f2,Pou5f1	PLCB1	intronic
20	8840154	0.89	0.98	rs6133635	C	A	0.09	0.44	0.66	0.32			HSMM	FibroP			Pax-6	PLCB1	intronic
20	8840293	0.89	0.98	rs6086657	T	C	0.09	0.44	0.66	0.32			HSMM				Hsf	PLCB1	intronic
20	8840670	0.89	0.98	rs4813874	A	T	0.11	0.44	0.66	0.32			HSMM, NHLF				7 altered motifs	PLCB1	intronic
20	8840702	0.89	0.98	rs4813875	A	C	0.1	0.44	0.66	0.32			HSMM, NHLF	AG04450			E2F	PLCB1	intronic
20	8840759	0.89	0.98	rs4813876	T	C	0.1	0.44	0.66	0.32			HSMM, NHLF	AG04450			Foxj2,Hoxa13,Maf	PLCB1	intronic
20	8840975	0.89	0.98	rs2066885	A	G	0.1	0.44	0.66	0.32			HSMM, NHLF	58 cell types	CFOS		5 altered motifs	PLCB1	intronic
20	8841005	0.89	0.98	rs2066884	A	G	0.1	0.44	0.66	0.32			HSMM, NHLF, HMEC	60 cell types	CFOS		5 altered motifs	PLCB1	intronic
20	8841105	0.89	0.98	rs2327117	G	A	0.09	0.44	0.66	0.32			HSMM, NHLF, HMEC	9 cell types	CFOS		5 altered motifs	PLCB1	intronic
20	8841183	0.89	0.98	rs2327118	G	A	0.1	0.44	0.66	0.32			HSMM, NHLF, HMEC					PLCB1	intronic
20	8842205	0.89	0.98	rs6133636	C	T	0.09	0.44	0.66	0.32							SZF1-1,Spz1,ZBTB33	PLCB1	intronic
20	8843256	0.89	0.98	rs6086660	T	G	0.1	0.44	0.66	0.32							4 altered motifs	PLCB1	intronic
20	8843306	0.89	0.98	rs6086661	C	T	0.1	0.45	0.66	0.32								PLCB1	intronic
20	8843353	0.89	0.98	rs6140778	A	G	0.1	0.44	0.66	0.32								PLCB1	intronic
20	8843845	0.87	0.96	rs6140779	A	G	0.1	0.44	0.66	0.32			H1				8 altered motifs	PLCB1	intronic
20	8844495	0.89	0.98	rs147271284	7-mer	A	0.14	0.44	0.66	0.32							8 altered motifs	PLCB1	intronic
20	8844694	0.89	0.98	rs6086662	T	C	0.15	0.44	0.66	0.32							DMRT2,Irf	PLCB1	intronic
20	8845519	0.89	0.98	rs6086664	T	A	0.08	0.44	0.66	0.32							LXR	PLCB1	intronic
20	8845933	0.85	0.94	rs6086665	G	A	0.09	0.44	0.65	0.32							12 altered motifs	PLCB1	intronic
20	8845937	0.85	0.94	rs6086666	G	A	0.16	0.44	0.65	0.31							Dmbx1	PLCB1	intronic
20	8846337	0.83	0.92	rs6140780	G	A	0.08	0.44	0.65	0.32							6 altered motifs	PLCB1	intronic
20	8846404	0.83	0.92	rs8182968	G	A	0.08	0.44	0.65	0.32							4 altered motifs	PLCB1	intronic
20	8846489	0.83	0.92	rs8182972	G	A	0.08	0.44	0.65	0.32							LBP-1	PLCB1	intronic
20	8846656	0.83	0.92	rs6086667	G	C	0.16	0.44	0.65	0.32							ATF3,STAT	PLCB1	intronic
20	8846739	0.83	0.92	rs6086668	A	G	0.16	0.44	0.65	0.32				Fibrobl			Dobox4,Mef2,STAT	PLCB1	intronic
20	8846835	0.83	0.92	rs6086669	G	T	0.16	0.44	0.65	0.32							7 altered motifs	PLCB1	intronic
20	8846925	0.83	0.92	rs6140781	G	A	0.16	0.44	0.65	0.31				FibroP				PLCB1	intronic
20	8846986	0.83	0.92	rs6086670	T	C	0.16	0.44	0.65	0.32				FibroP,HFF-Myc			CEBPB,Osf2	PLCB1	intronic
20	8847252	0.83	0.92	rs6077444	G	A	0.16	0.44	0.65	0.32							4 altered motifs	PLCB1	intronic
20	8847311	0.83	0.92	rs6086671	G	C	0.16	0.44	0.65	0.32							SEF-1	PLCB1	intronic
20	8847414	0.83	0.92	rs6086672	G	A	0.16	0.44	0.65	0.32			HSMM					PLCB1	intronic
20	8847727	0.83	0.92	rs3891453	A	G	0.16	0.44	0.65	0.32			HSMM					PLCB1	intronic
20	8848529	0.83	0.92	rs6077445	A	G	0.16	0.44	0.65	0.32							5 altered motifs	PLCB1	intronic
20	8848688	0.83	0.92	rs3915507	A	G	0.16	0.44	0.65	0.31							EBF	PLCB1	intronic
20	8848724	0.83	0.92	rs3848829	T	C	0.15	0.44	0.65	0.31							4 altered motifs	PLCB1	intronic

20	8848994	0.83	0.92	rs3848830	C	T	0.16	0.44	0.65	0.31							Sox	PLCB1	intronic
20	8849379	0.81	0.91	rs4816099	A	T	0.16	0.43	0.64	0.3							HNF4,Nkx2,TFE	PLCB1	intronic
20	8849390	0.83	0.92	rs4813878	C	T	0.16	0.44	0.65	0.3							SETDB1	PLCB1	intronic
20	8849910	0.83	0.92	rs2327119	C	A	0.16	0.44	0.65	0.31							Pou2f2,Zfp105	PLCB1	intronic
20	8851321	0.83	0.92	rs3848831	T	C	0.16	0.44	0.65	0.31							Foxc1,Maf,Pou2f2	PLCB1	intronic
Query SNP: rs6039302 and variants with r^2 >= 0.8																			
**chr**	**pos (hg19)**	**LD (r^2)**	**LD (D')**	**variant**	**Ref**	**Alt**	**AFR freq**	**AMR freq**	**ASN freq**	**EUR freq**	**SiPhy cons**	**Promoter histone marks**	**Enhancer histone marks**	**DNAse**	**Proteins bound**	**eQTL tissues**	**Motifs changed**	**GENCODE genes**	**dbSNP func annot**
20	8808310	0.86	0.97	rs4816090	C	T	0.13	0.46	0.62	0.33							4 altered motifs	PLCB1	intronic
20	8808434	0.86	0.97	rs4816091	C	T	0.15	0.46	0.62	0.33							Foxj1	PLCB1	intronic
20	8810060	0.87	0.98	rs6077432	C	T	0.04	0.43	0.62	0.3				5 cell types	CTCF		Brachyury,Pou5f1	PLCB1	intronic
20	8810363	0.95	0.98	rs2223571	A	G	0.14	0.46	0.64	0.33							Bbx,HMG-IY,Nkx6-1	PLCB1	intronic
20	8814051	0.87	0.98	rs6140737	G	A	0.15	0.46	0.62	0.33							6 altered motifs	PLCB1	intronic
20	8816665	0.95	0.98	rs6086622	G	A	0.04	0.43	0.64	0.3			HSMM					PLCB1	intronic
20	8816728	0.96	0.98	rs6140738	G	T	0.04	0.43	0.64	0.3			HSMM					PLCB1	intronic
20	8817665	0.96	0.98	rs6077434	C	T	0.16	0.48	0.64	0.36			HSMM, NHLF					PLCB1	intronic
20	8818022	0.95	0.98	rs6086624	C	T	0.16	0.47	0.65	0.36			HSMM, NHLF				CEBPB	PLCB1	intronic
20	8818326	0.96	0.98	rs6086625	C	T	0.16	0.47	0.64	0.36			HSMM, NHLF	51 cell types			Pax-8	PLCB1	intronic
20	8818335	0.96	0.98	rs6086626	A	G	0.16	0.47	0.64	0.36			HSMM, NHLF	42 cell types			AP-1,ZID	PLCB1	intronic
20	8818667	0.96	0.98	rs6140739	T	A	0.16	0.47	0.64	0.36			HSMM				4 altered motifs	PLCB1	intronic
20	8818700	0.96	0.98	rs6140740	T	A	0.16	0.47	0.64	0.36			HSMM					PLCB1	intronic
20	8818864	0.96	0.98	rs6140741	T	C	0.16	0.47	0.64	0.36							Pou2f2	PLCB1	intronic
20	8819112	0.96	0.98	rs6140742	G	C	0.16	0.47	0.64	0.36							Foxa,Pou1f1,STAT	PLCB1	intronic
20	8819136	0.96	0.98	rs11698656	C	T	0.16	0.47	0.64	0.36							Evi-1,Osf2	PLCB1	intronic
20	8819200	0.96	0.98	rs11697064	T	C	0.16	0.47	0.64	0.36							DMRT1,DMRT4,Foxa	PLCB1	intronic
20	8819209	0.96	0.98	rs11697066	T	C	0.16	0.47	0.64	0.36							ERalpha-a,Foxa,Mef2	PLCB1	intronic
20	8819295	0.95	0.98	rs11698557	G	C	0.16	0.47	0.64	0.36								PLCB1	intronic
20	8819303	0.96	0.98	rs6077436	G	A	0.16	0.47	0.64	0.36							Irf,Osf2	PLCB1	intronic
20	8819311	0.96	0.98	rs11697086	T	C	0.04	0.43	0.64	0.3								PLCB1	intronic
20	8820056	0.95	0.98	rs6086629	T	A	0.16	0.47	0.65	0.36							Nkx2,STAT	PLCB1	intronic
20	8820280	0.98	0.99	rs2179475	C	G	0.16	0.47	0.64	0.36							Gfi1,HMG-IY	PLCB1	intronic
20	8820448	0.8	0.99	rs2143259	C	T	0.16	0.45	0.6	0.34							6 altered motifs	PLCB1	intronic
20	8820502	0.97	0.99	rs2143260	G	A	0.17	0.48	0.64	0.36							AP-1,ATF3	PLCB1	intronic
20	8820549	0.81	0.99	rs2143261	A	G	0.16	0.45	0.6	0.35							6 altered motifs	PLCB1	intronic
20	8820747	0.98	0.99	rs6086630	C	T	0.16	0.47	0.64	0.36							6 altered motifs	PLCB1	intronic
20	8821275	0.98	0.99	rs6140743	C	T	0.16	0.47	0.64	0.36							Irf,STAT,p300	PLCB1	intronic
20	8821334	0.98	0.99	rs6140744	C	T	0.16	0.47	0.64	0.36							Gfi1,HNF6,Sox	PLCB1	intronic
20	8821517	0.95	0.98	rs6140747	G	A	0.16	0.47	0.64	0.36							AP-4,HP1-site-factor,Zbtb3	PLCB1	intronic
20	8821577	0.96	0.98	rs6140748	C	T	0.16	0.48	0.65	0.36							4 altered motifs	PLCB1	intronic
20	8821621	0.96	0.98	rs6140750	A	G	0.16	0.47	0.65	0.35							5 altered motifs	PLCB1	intronic
20	8821846	0.98	0.99	rs2179476	T	A	0.16	0.47	0.64	0.36							6 altered motifs	PLCB1	intronic
20	8821969	0.98	0.99	rs2143262	T	C	0.16	0.47	0.64	0.36							Sox	PLCB1	intronic
20	8822431	0.98	0.99	rs12624809	G	C	0.16	0.47	0.64	0.36							E2F,Irf	PLCB1	intronic
20	8822723	0.98	0.99	rs6140753	G	A	0.16	0.47	0.64	0.36							10 altered motifs	PLCB1	intronic
20	8822931	0.98	0.99	rs6086632	T	C	0.16	0.47	0.64	0.36							Foxp1,HNF1	PLCB1	intronic
20	8823064	0.98	0.99	rs6086633	C	T	0.16	0.47	0.64	0.36							4 altered motifs	PLCB1	intronic
20	8823539	0.86	1	rs6140754	G	A	0.16	0.44	0.61	0.34							RXRA,ZEB1,p300	PLCB1	intronic
20	8824009	0.98	1	rs6133627	C	T	0.16	0.47	0.65	0.36							LBP-1	PLCB1	intronic
20	8824117	1	1	rs6140755	T	C	0.19	0.47	0.65	0.36								PLCB1	intronic
20	8824259	0.91	0.98	rs6140756	A	T	0.16	0.47	0.63	0.35							8 altered motifs	PLCB1	intronic
20	8824322	1	1	rs6086635	A	G	0.17	0.47	0.65	0.36							7 altered motifs	PLCB1	intronic
20	8824717	1	1	rs11087826	A	G	0.19	0.47	0.65	0.36			HepG2				HNF4,Mef2	PLCB1	intronic
20	8825193	1	1	rs6140758	C	T	0.04	0.43	0.65	0.3			HepG2				4 altered motifs	PLCB1	intronic
20	8825509	0.85	0.98	rs6140760	A	G	0.18	0.46	0.62	0.35							Ik-2,NRSF,Pitx2	PLCB1	intronic
20	8825748	0.99	1	rs6086640	A	G	0.19	0.47	0.64	0.35							Pax-5,ZID	PLCB1	intronic
20	8825793	0.99	1	rs6133628	C	T	0.15	0.47	0.64	0.35							4 altered motifs	PLCB1	intronic
20	8825801	0.99	1	rs6140761	T	C	0.18	0.47	0.64	0.35							HDAC2,Zbtb12	PLCB1	intronic
20	8826177	1	1	rs6077437	A	G	0.04	0.43	0.65	0.3							Pou5f1	PLCB1	intronic
20	8826360	1	1	rs6140764	G	A	0.19	0.47	0.65	0.36							Evi-1,Pou2f2	PLCB1	intronic
20	8826412	1	1	rs55875629	A	C	0.16	0.47	0.65	0.36							6 altered motifs	PLCB1	intronic
20	8826507	1	1	rs6077438	T	C	0.04	0.43	0.65	0.3							BRCA1,Cart1,Sox	PLCB1	intronic
20	8826512	1	1	rs6077439	A	G	0.16	0.47	0.65	0.35							Cart1,Nkx2,Sox	PLCB1	intronic
20	8826632	1	1	rs6077440	C	T	0.04	0.43	0.65	0.3							Hoxb13	PLCB1	intronic
20	8826987	1	1	rs6086641	A	T	0.16	0.47	0.65	0.36							HNF1,Pitx2	PLCB1	intronic

20	8827858	0.98	1	rs202130727	G	GAT	0.16	0.47	0.64	0.36							6 altered motifs	PLCB1	intronic
20	8827973	1	1	rs6086642	A	G	0.19	0.47	0.65	0.36							Hsf,Sin3Ak-20	PLCB1	intronic
20	8828487	0.98	1	rs6086643	G	T	0.04	0.43	0.64	0.3							4 altered motifs	PLCB1	intronic
20	8828492	0.98	1	rs6086644	G	A	0.06	0.43	0.64	0.3							6 altered motifs	PLCB1	intronic
20	8828502	0.99	1	rs6086645	T	C	0.19	0.47	0.64	0.35							HDAC2,Nanog,Sox	PLCB1	intronic
20	8828781	0.99	1	rs6140767	G	C	0.19	0.47	0.64	0.36				Melano			7 altered motifs	PLCB1	intronic
20	8829046	1	1	rs2876146	G	C	0.19	0.47	0.65	0.36							Nkx2	PLCB1	intronic
20	8829845	0.91	1	rs3902337	C	A	0.16	0.46	0.62	0.34							4 altered motifs	PLCB1	intronic
20	8829914	1	1	rs3902336	G	A	0.16	0.47	0.65	0.36								PLCB1	intronic
20	8830592	0.96	1	rs6140768	G	A	0.16	0.47	0.63	0.35							8 altered motifs	PLCB1	intronic
20	8831137	1	1	rs6039302	C	T	0.18	0.47	0.65	0.36							Foxp1	PLCB1	intronic
20	8831211	1	1	rs6056206	T	C	0.15	0.47	0.65	0.36							6 altered motifs	PLCB1	intronic
20	8831253	1	1	rs6039303	A	T	0.15	0.47	0.65	0.36							5 altered motifs	PLCB1	intronic
20	8831397	1	1	rs6056207	G	A	0.15	0.48	0.65	0.36							Hand1,NF-I,Smad3	PLCB1	intronic
20	8831501	1	1	rs4813873	G	A	0.16	0.47	0.65	0.36							Cdc5	PLCB1	intronic
20	8831744	1	1	rs4816093	A	T	0.16	0.48	0.65	0.36							7 altered motifs	PLCB1	intronic
20	8832767	1	1	rs6086647	C	T	0.19	0.47	0.65	0.36			HSMM, NHLF	5 cell types			4 altered motifs	PLCB1	intronic
20	8833132	1	1	rs4816094	G	A	0.18	0.47	0.65	0.36			HSMM				CDP	PLCB1	intronic
20	8833198	1	1	rs4816095	T	C	0.21	0.48	0.65	0.36			HSMM				GATA,Sox	PLCB1	intronic
20	8833231	1	1	rs4816096	T	C	0.18	0.47	0.65	0.36			HSMM				FXR,LXR	PLCB1	intronic
20	8833373	1	1	rs4816097	A	G	0.18	0.47	0.65	0.36			HSMM	Melano			Hoxa4	PLCB1	intronic
20	8833751	0.96	0.98	rs35030007	A	G	0.04	0.43	0.64	0.3			HSMM				4 altered motifs	PLCB1	intronic
20	8833755	0.98	1	rs11366851	CT	C	0.04	0.43	0.64	0.31			HSMM				6 altered motifs	PLCB1	intronic
20	8833905	0.99	1	rs4816098	G	A	0.17	0.47	0.64	0.36			HSMM				10 altered motifs	PLCB1	intronic
20	8834271	0.96	1	rs3926282	A	G	0.18	0.48	0.63	0.35			4 cell types				LBP-9	PLCB1	intronic
20	8834276	0.93	1	rs1967672	A	G	0.17	0.47	0.63	0.35			4 cell types				Hoxa3,Myc	PLCB1	intronic
20	8834367	0.98	1	rs4277595	A	G	0.2	0.47	0.64	0.35			4 cell types				4 altered motifs	PLCB1	intronic
20	8835779	0.98	1	rs6086649	A	T	0.04	0.41	0.64	0.29			HSMM, Huvec, NHLF				7 altered motifs	PLCB1	intronic
20	8837136	0.9	0.98	rs6133630	G	A	0.09	0.44	0.66	0.32			HSMM				6 altered motifs	PLCB1	intronic
20	8837162	0.9	0.98	rs6086650	G	A	0.09	0.44	0.66	0.32			HSMM				4 altered motifs	PLCB1	intronic
20	8837407	0.91	0.98	rs4426594	G	A	0.09	0.44	0.66	0.32							GR	PLCB1	intronic
20	8837909	0.89	0.96	rs6133632	G	T	0.04	0.41	0.65	0.3								PLCB1	intronic
20	8838220	0.91	0.98	rs6086652	C	T	0.1	0.44	0.66	0.32								PLCB1	intronic
20	8838343	0.91	0.98	rs6086653	A	G	0.04	0.41	0.66	0.3							Esr2,Spz1	PLCB1	intronic
20	8838376	0.91	0.98	rs6086654	C	T	0.09	0.44	0.66	0.32							ATF3,HEY1,Pdx1	PLCB1	intronic
20	8838914	0.91	0.98	rs6140771	C	T	0.09	0.44	0.66	0.32				8 cell types			CEBPA	PLCB1	intronic
20	8838963	0.91	0.98	rs6140772	A	C	0.1	0.44	0.66	0.32				6 cell types				PLCB1	intronic
20	8839222	0.91	0.98	rs11087827	A	T	0.1	0.44	0.66	0.32			HepG2, NHLF, HSMM	8 cell types	FOXA1,P300			PLCB1	intronic
20	8839408	0.91	0.98	rs13041928	G	A	0.09	0.44	0.66	0.32			NHLF, HSMM	HA-sp,RPTEC	FOXA1,FOXA2,P300			PLCB1	intronic
20	8839522	0.91	0.98	rs6140773	T	C	0.1	0.44	0.66	0.32			NHLF, HSMM					PLCB1	intronic
20	8839602	0.9	0.98	rs6077443	G	A	0.1	0.44	0.66	0.32			NHLF, HSMM				GR	PLCB1	intronic
20	8840064	0.91	0.98	rs6133634	C	G	0.1	0.44	0.66	0.32			HSMM				HMG-IY,Pou2f2,Pou5f1	PLCB1	intronic
20	8840154	0.91	0.98	rs6133635	C	A	0.09	0.44	0.66	0.32			HSMM	FibroP			Pax-6	PLCB1	intronic
20	8840293	0.91	0.98	rs6086657	T	C	0.09	0.44	0.66	0.32			HSMM				Hsf	PLCB1	intronic
20	8840670	0.91	0.98	rs4813874	A	T	0.11	0.44	0.66	0.32			HSMM, NHLF				7 altered motifs	PLCB1	intronic
20	8840702	0.91	0.98	rs4813875	A	C	0.1	0.44	0.66	0.32			HSMM, NHLF	AG04450			E2F	PLCB1	intronic
20	8840759	0.91	0.98	rs4813876	T	C	0.1	0.44	0.66	0.32			HSMM, NHLF	AG04450			Foxj2,Hoxa13,Maf	PLCB1	intronic
20	8840975	0.91	0.98	rs2066885	A	G	0.1	0.44	0.66	0.32			HSMM, NHLF	58 cell types	CFOS		5 altered motifs	PLCB1	intronic
20	8841005	0.91	0.98	rs2066884	A	G	0.1	0.44	0.66	0.32			HSMM, NHLF, HMEC	60 cell types	CFOS		5 altered motifs	PLCB1	intronic
20	8841105	0.91	0.98	rs2327117	G	A	0.09	0.44	0.66	0.32			HSMM, NHLF, HMEC	9 cell types	CFOS		5 altered motifs	PLCB1	intronic
20	8841183	0.91	0.98	rs2327118	G	A	0.1	0.44	0.66	0.32			HSMM, NHLF, HMEC					PLCB1	intronic
20	8842205	0.91	0.98	rs6133636	C	T	0.09	0.44	0.66	0.32							SZF1-1,Spz1,ZBTB33	PLCB1	intronic
20	8843256	0.91	0.98	rs6086660	T	G	0.1	0.44	0.66	0.32							4 altered motifs	PLCB1	intronic
20	8843306	0.91	0.98	rs6086661	C	T	0.1	0.45	0.66	0.32								PLCB1	intronic
20	8843353	0.91	0.98	rs6140778	A	G	0.1	0.44	0.66	0.32								PLCB1	intronic
20	8843845	0.9	0.97	rs6140779	A	G	0.1	0.44	0.66	0.32			H1				8 altered motifs	PLCB1	intronic
20	8844495	0.91	0.98	rs147271284	7-mer	A	0.14	0.44	0.66	0.32							8 altered motifs	PLCB1	intronic
20	8844694	0.91	0.98	rs6086662	T	C	0.15	0.44	0.66	0.32							DMRT2,Irf	PLCB1	intronic
20	8845050	0.81	0.98	rs6108204	A	G	0.15	0.44	0.69	0.32							4 altered motifs	PLCB1	intronic
20	8845067	0.81	0.98	rs6086663	G	T	0.14	0.44	0.69	0.32							6 altered motifs	PLCB1	intronic
20	8845519	0.91	0.98	rs6086664	T	A	0.08	0.44	0.66	0.32							LXR	PLCB1	intronic
20	8845933	0.87	0.95	rs6086665	G	A	0.09	0.44	0.65	0.32							12 altered motifs	PLCB1	intronic
20	8845937	0.87	0.95	rs6086666	G	A	0.16	0.44	0.65	0.31							Dmbx1	PLCB1	intronic
20	8846337	0.85	0.92	rs6140780	G	A	0.08	0.44	0.65	0.32							6 altered motifs	PLCB1	intronic
20	8846404	0.85	0.92	rs8182968	G	A	0.08	0.44	0.65	0.32							4 altered motifs	PLCB1	intronic

20	8846489	0.85	0.92	rs8182972	G	A	0.08	0.44	0.65	0.32							LBP-1	PLCB1	intronic
20	8846656	0.85	0.92	rs6086667	G	C	0.16	0.44	0.65	0.32							ATF3,STAT	PLCB1	intronic
20	8846739	0.85	0.92	rs6086668	A	G	0.16	0.44	0.65	0.32				Fibrobl			Dobox4,Mef2,STAT	PLCB1	intronic
20	8846835	0.85	0.92	rs6086669	G	T	0.16	0.44	0.65	0.32							7 altered motifs	PLCB1	intronic
20	8846925	0.85	0.92	rs6140781	G	A	0.16	0.44	0.65	0.31				FibroP				PLCB1	intronic
20	8846986	0.85	0.92	rs6086670	T	C	0.16	0.44	0.65	0.32				FibroP,HFF-Myc			CEBPB,Osf2	PLCB1	intronic
20	8847252	0.85	0.92	rs6077444	G	A	0.16	0.44	0.65	0.32							4 altered motifs	PLCB1	intronic
20	8847311	0.85	0.92	rs6086671	G	C	0.16	0.44	0.65	0.32							SEF-1	PLCB1	intronic
20	8847414	0.85	0.92	rs6086672	G	A	0.16	0.44	0.65	0.32			HSMM					PLCB1	intronic
20	8847727	0.85	0.92	rs3891453	A	G	0.16	0.44	0.65	0.32			HSMM					PLCB1	intronic
20	8848529	0.85	0.92	rs6077445	A	G	0.16	0.44	0.65	0.32							5 altered motifs	PLCB1	intronic
20	8848688	0.85	0.92	rs3915507	A	G	0.16	0.44	0.65	0.31							EBF	PLCB1	intronic
20	8848724	0.85	0.92	rs3848829	T	C	0.15	0.44	0.65	0.31							4 altered motifs	PLCB1	intronic
20	8848994	0.85	0.92	rs3848830	C	T	0.16	0.44	0.65	0.31							Sox	PLCB1	intronic
20	8849379	0.83	0.92	rs4816099	A	T	0.16	0.43	0.64	0.3							HNF4,Nkx2,TFE	PLCB1	intronic
20	8849390	0.85	0.92	rs4813878	C	T	0.16	0.44	0.65	0.3							SETDB1	PLCB1	intronic
20	8849910	0.85	0.92	rs2327119	C	A	0.16	0.44	0.65	0.31							Pou2f2,Zfp105	PLCB1	intronic
20	8851321	0.85	0.92	rs3848831	T	C	0.16	0.44	0.65	0.31							Foxc1,Maf,Pou2f2	PLCB1	intronic
Query SNP: rs6086653 and variants with r^2 >= 0.8																			
chr	pos (hg19)	LD (r²)	LD (D')	variant	Ref	Alt	AFR freq	AMR freq	ASN freq	EUR freq	SiPhy cons	Promoter histone marks	Enhancer histone marks	DNAse	Proteins bound	eQTL tissues	Motifs changed	GENCODE genes	dbSNP func annot
20	8810363	0.86	0.97	rs2223571	A	G	0.14	0.46	0.64	0.33							Bbx,HMG-IY,Nkx6-1	PLCB1	intronic
20	8816665	0.86	0.97	rs6086622	G	A	0.04	0.43	0.64	0.3			HSMM					PLCB1	intronic
20	8816728	0.87	0.97	rs6140738	G	T	0.04	0.43	0.64	0.3			HSMM					PLCB1	intronic
20	8817665	0.87	0.97	rs6077434	C	T	0.16	0.48	0.64	0.36			HSMM, NHLF					PLCB1	intronic
20	8818022	0.86	0.95	rs6086624	C	T	0.16	0.47	0.65	0.36			HSMM, NHLF				CEBPB	PLCB1	intronic
20	8818326	0.87	0.97	rs6086625	C	T	0.16	0.47	0.64	0.36			HSMM, NHLF	51 cell types			Pax-8	PLCB1	intronic
20	8818335	0.87	0.97	rs6086626	A	G	0.16	0.47	0.64	0.36			HSMM, NHLF	42 cell types			AP-1,ZID	PLCB1	intronic
20	8818667	0.87	0.97	rs6140739	T	A	0.16	0.47	0.64	0.36			HSMM				4 altered motifs	PLCB1	intronic
20	8818700	0.87	0.97	rs6140740	T	A	0.16	0.47	0.64	0.36			HSMM					PLCB1	intronic
20	8818864	0.87	0.97	rs6140741	T	C	0.16	0.47	0.64	0.36							Pou2f2	PLCB1	intronic
20	8819112	0.87	0.97	rs6140742	G	C	0.16	0.47	0.64	0.36							Foxa,Pou1f1,STAT	PLCB1	intronic
20	8819136	0.87	0.97	rs11698656	C	T	0.16	0.47	0.64	0.36							Evi-1,Osf2	PLCB1	intronic
20	8819200	0.87	0.97	rs11697064	T	C	0.16	0.47	0.64	0.36							DMRT1,DMRT4,Foxa	PLCB1	intronic
20	8819209	0.87	0.97	rs11697066	T	C	0.16	0.47	0.64	0.36							ERalpha-a,Foxa,Mef2	PLCB1	intronic
20	8819295	0.86	0.97	rs11698557	G	C	0.16	0.47	0.64	0.36								PLCB1	intronic
20	8819303	0.87	0.97	rs6077436	G	A	0.16	0.47	0.64	0.36							Irf,Osf2	PLCB1	intronic
20	8819311	0.87	0.97	rs11697086	T	C	0.04	0.43	0.64	0.3								PLCB1	intronic
20	8820056	0.89	0.97	rs6086629	T	A	0.16	0.47	0.65	0.36							Nkx2,STAT	PLCB1	intronic
20	8820280	0.89	0.98	rs2179475	C	G	0.16	0.47	0.64	0.36							Gfi1,HMG-IY	PLCB1	intronic
20	8820502	0.88	0.98	rs2143260	G	A	0.17	0.48	0.64	0.36							AP-1,ATF3	PLCB1	intronic
20	8820747	0.89	0.98	rs6086630	C	T	0.16	0.47	0.64	0.36							6 altered motifs	PLCB1	intronic
20	8821275	0.89	0.98	rs6140743	C	T	0.16	0.47	0.64	0.36							Irf,STAT,p300	PLCB1	intronic
20	8821334	0.89	0.98	rs6140744	C	T	0.16	0.47	0.64	0.36							Gfi1,HNF6,Sox	PLCB1	intronic
20	8821517	0.86	0.96	rs6140747	G	A	0.16	0.47	0.64	0.36							AP-4,HP1-site-factor Zbtb3	PLCB1	intronic
20	8821577	0.87	0.96	rs6140748	C	T	0.16	0.48	0.65	0.36							4 altered motifs	PLCB1	intronic
20	8821621	0.87	0.96	rs6140750	A	G	0.16	0.47	0.65	0.35							5 altered motifs	PLCB1	intronic
20	8821846	0.89	0.98	rs2179476	T	A	0.16	0.47	0.64	0.36							6 altered motifs	PLCB1	intronic
20	8821969	0.89	0.98	rs2143262	T	C	0.16	0.47	0.64	0.36							Sox	PLCB1	intronic
20	8822431	0.89	0.98	rs12624809	G	C	0.16	0.47	0.64	0.36							E2F,Irf	PLCB1	intronic
20	8822723	0.89	0.98	rs6140753	G	A	0.16	0.47	0.64	0.36							10 altered motifs	PLCB1	intronic
20	8822931	0.89	0.98	rs6086632	T	C	0.16	0.47	0.64	0.36							Foxp1,HNF1	PLCB1	intronic
20	8823064	0.89	0.98	rs6086633	C	T	0.16	0.47	0.64	0.36							4 altered motifs	PLCB1	intronic
20	8824009	0.9	0.97	rs6133627	C	T	0.16	0.47	0.65	0.36							LBP-1	PLCB1	intronic
20	8824117	0.91	0.98	rs6140755	T	C	0.19	0.47	0.65	0.36								PLCB1	intronic
20	8824259	0.86	0.98	rs6140756	A	T	0.16	0.47	0.63	0.35							8 altered motifs	PLCB1	intronic
20	8824322	0.91	0.98	rs6086635	A	G	0.17	0.47	0.65	0.36							7 altered motifs	PLCB1	intronic
20	8824717	0.91	0.98	rs11087826	A	G	0.19	0.47	0.65	0.36			HepG2				HNF4,Mef2	PLCB1	intronic
20	8825193	0.91	0.98	rs6140758	C	T	0.04	0.43	0.65	0.3			HepG2				4 altered motifs	PLCB1	intronic
20	8825509	0.8	0.98	rs6140760	A	G	0.18	0.46	0.62	0.35							Ik-2,NRSF,Pitx2	PLCB1	intronic
20	8825748	0.9	0.98	rs6086640	A	G	0.19	0.47	0.64	0.35							Pax-5,ZID	PLCB1	intronic
20	8825793	0.9	0.98	rs6133628	C	T	0.15	0.47	0.64	0.35							4 altered motifs	PLCB1	intronic
20	8825801	0.9	0.98	rs6140761	T	C	0.18	0.47	0.64	0.35							HDAC2,Zbtb12	PLCB1	intronic
20	8826177	0.91	0.98	rs6077437	A	G	0.04	0.43	0.65	0.3							Pou5f1	PLCB1	intronic

20	8826360	0.91	0.98	rs6140764	G	A	0.19	0.47	0.65	0.36							Evi-1,Pou2f2	PLCB1	intronic
20	8826412	0.91	0.98	rs55875629	A	C	0.16	0.47	0.65	0.36							6 altered motifs	PLCB1	intronic
20	8826507	0.91	0.98	rs6077438	T	C	0.04	0.43	0.65	0.3							BRCA1,Cart1,Sox	PLCB1	intronic
20	8826512	0.91	0.98	rs6077439	A	G	0.16	0.47	0.65	0.35							Cart1,Nkx2,Sox	PLCB1	intronic
20	8826632	0.91	0.98	rs6077440	C	T	0.04	0.43	0.65	0.3							Hoxb13	PLCB1	intronic
20	8826987	0.91	0.98	rs6086641	A	T	0.16	0.47	0.65	0.36							HNF1,Pitx2	PLCB1	intronic
20	8827858	0.89	0.98	rs202130727	G	GAT	0.16	0.47	0.64	0.36							6 altered motifs	PLCB1	intronic
20	8827973	0.91	0.98	rs6086642	A	G	0.19	0.47	0.65	0.36							Hsf,Sin3Ak-20	PLCB1	intronic
20	8828487	0.9	0.98	rs6086643	G	T	0.04	0.43	0.64	0.3							4 altered motifs	PLCB1	intronic
20	8828492	0.9	0.98	rs6086644	G	A	0.06	0.43	0.64	0.3							6 altered motifs	PLCB1	intronic
20	8828502	0.9	0.98	rs6086645	T	C	0.19	0.47	0.64	0.35							HDAC2,Nanog,Sox	PLCB1	intronic
20	8828781	0.9	0.98	rs6140767	G	C	0.19	0.47	0.64	0.36				Melano			7 altered motifs	PLCB1	intronic
20	8829046	0.91	0.98	rs2876146	G	C	0.19	0.47	0.65	0.36							Nkx2	PLCB1	intronic
20	8829845	0.83	0.98	rs3902337	C	A	0.16	0.46	0.62	0.34							4 altered motifs	PLCB1	intronic
20	8829914	0.91	0.98	rs3902336	G	A	0.16	0.47	0.65	0.36								PLCB1	intronic
20	8830592	0.87	0.98	rs6140768	G	A	0.16	0.47	0.63	0.35							8 altered motifs	PLCB1	intronic
20	8831137	0.91	0.98	rs6039302	C	T	0.18	0.47	0.65	0.36							Foxp1	PLCB1	intronic
20	8831211	0.91	0.98	rs6056206	T	C	0.15	0.47	0.65	0.36							6 altered motifs	PLCB1	intronic
20	8831253	0.91	0.98	rs6039303	A	T	0.15	0.47	0.65	0.36							5 altered motifs	PLCB1	intronic
20	8831397	0.91	0.98	rs6056207	G	A	0.15	0.48	0.65	0.36							Hand1,NF-I,Smad3	PLCB1	intronic
20	8831501	0.91	0.98	rs4813873	G	A	0.16	0.47	0.65	0.36							Cdc5	PLCB1	intronic
20	8831744	0.91	0.98	rs4816093	A	T	0.16	0.48	0.65	0.36							7 altered motifs	PLCB1	intronic
20	8832767	0.91	0.98	rs6086647	C	T	0.19	0.47	0.65	0.36			HSMM, NHLF	5 cell types			4 altered motifs	PLCB1	intronic
20	8833132	0.91	0.98	rs4816094	G	A	0.18	0.47	0.65	0.36			HSMM				CDP	PLCB1	intronic
20	8833198	0.91	0.98	rs4816095	T	C	0.21	0.48	0.65	0.36			HSMM				GATA,Sox	PLCB1	intronic
20	8833231	0.91	0.98	rs4816096	T	C	0.18	0.47	0.65	0.36			HSMM				FXR,LXR	PLCB1	intronic
20	8833373	0.91	0.98	rs4816097	A	G	0.18	0.47	0.65	0.36			HSMM	Melano			Hoxa4	PLCB1	intronic
20	8833751	0.89	0.98	rs35030007	A	G	0.04	0.43	0.64	0.3			HSMM				4 altered motifs	PLCB1	intronic
20	8833755	0.9	0.99	rs11366851	CT	C	0.04	0.43	0.64	0.31			HSMM				6 altered motifs	PLCB1	intronic
20	8833905	0.92	0.99	rs4816098	G	A	0.17	0.47	0.64	0.36			HSMM				10 altered motifs	PLCB1	intronic
20	8834271	0.88	0.99	rs3926282	A	G	0.18	0.48	0.63	0.35			4 cell types				LBP-9	PLCB1	intronic
20	8834276	0.86	0.99	rs1967672	A	G	0.17	0.47	0.63	0.35			4 cell types				Hoxa3,Myc	PLCB1	intronic
20	8834367	0.9	0.99	rs4277595	A	G	0.2	0.47	0.64	0.35			4 cell types				4 altered motifs	PLCB1	intronic
20	8835779	0.93	1	rs6086649	A	T	0.04	0.41	0.64	0.29			HSMM, Huvec, NHLF				7 altered motifs	PLCB1	intronic
20	8837136	0.99	1	rs6133630	G	A	0.09	0.44	0.66	0.32			HSMM				6 altered motifs	PLCB1	intronic
20	8837162	0.99	1	rs6086650	G	A	0.09	0.44	0.66	0.32			HSMM				4 altered motifs	PLCB1	intronic
20	8837407	1	1	rs4426594	G	A	0.09	0.44	0.66	0.32							GR	PLCB1	intronic
20	8837909	0.98	1	rs6133632	G	T	0.04	0.41	0.65	0.3								PLCB1	intronic
20	8838220	1	1	rs6086652	C	T	0.1	0.44	0.66	0.32								PLCB1	intronic
20	8838343	1	1	rs6086653	A	G	0.04	0.41	0.66	0.3							Esr2,Spz1	PLCB1	intronic
20	8838376	1	1	rs6086654	C	T	0.09	0.44	0.66	0.32							ATF3,HEY1,Pdx1	PLCB1	intronic
20	8838914	1	1	rs6140771	C	T	0.09	0.44	0.66	0.32				8 cell types			CEBPA	PLCB1	intronic
20	8838963	1	1	rs6140772	A	C	0.1	0.44	0.66	0.32				6 cell types				PLCB1	intronic
20	8839222	1	1	rs11087827	A	T	0.1	0.44	0.66	0.32			HepG2, NHLF, HSMM	8 cell types	FOXA1,P300			PLCB1	intronic
20	8839408	1	1	rs13041928	G	A	0.09	0.44	0.66	0.32			NHLF, HSMM	HA-sp,RPTEC	FOXA1,FOXA2,P300			PLCB1	intronic
20	8839522	1	1	rs6140773	T	C	0.1	0.44	0.66	0.32			NHLF, HSMM					PLCB1	intronic
20	8839602	0.99	1	rs6077443	G	A	0.1	0.44	0.66	0.32			NHLF, HSMM				GR	PLCB1	intronic
20	8840064	1	1	rs6133634	C	G	0.1	0.44	0.66	0.32			HSMM				HMG-IY,Pou2f2,Pou5f1	PLCB1	intronic
20	8840154	1	1	rs6133635	C	A	0.09	0.44	0.66	0.32			HSMM	FibroP			Pax-6	PLCB1	intronic
20	8840293	1	1	rs6086657	T	C	0.09	0.44	0.66	0.32			HSMM				Hsf	PLCB1	intronic
20	8840670	1	1	rs4813874	A	T	0.11	0.44	0.66	0.32			HSMM, NHLF				7 altered motifs	PLCB1	intronic
20	8840702	1	1	rs4813875	A	C	0.1	0.44	0.66	0.32			HSMM, NHLF	AG04450			E2F	PLCB1	intronic
20	8840759	1	1	rs4813876	T	C	0.1	0.44	0.66	0.32			HSMM, NHLF	AG04450			Foxj2,Hoxa13,Maf	PLCB1	intronic
20	8840975	1	1	rs2066885	A	G	0.1	0.44	0.66	0.32			HSMM, NHLF	58 cell types	CFOS		5 altered motifs	PLCB1	intronic
20	8841005	1	1	rs2066884	A	G	0.1	0.44	0.66	0.32			HSMM, NHLF, HMEC	60 cell types	CFOS		5 altered motifs	PLCB1	intronic
20	8841105	1	1	rs2327117	G	A	0.09	0.44	0.66	0.32			HSMM, NHLF, HMEC	9 cell types	CFOS		5 altered motifs	PLCB1	intronic
20	8841183	1	1	rs2327118	G	A	0.1	0.44	0.66	0.32			HSMM, NHLF, HMEC					PLCB1	intronic
20	8842205	1	1	rs6133636	C	T	0.09	0.44	0.66	0.32							SZF1-1,Spz1,ZBTB33	PLCB1	intronic
20	8842246	0.84	1	rs6086659	G	A	0.1	0.43	0.62	0.31							4 altered motifs	PLCB1	intronic
20	8843256	1	1	rs6086660	T	G	0.1	0.44	0.66	0.32							4 altered motifs	PLCB1	intronic
20	8843306	1	1	rs6086661	C	T	0.1	0.45	0.66	0.32								PLCB1	intronic
20	8843353	1	1	rs6140778	A	G	0.1	0.44	0.66	0.32								PLCB1	intronic
20	8843845	0.98	1	rs6140779	A	G	0.1	0.44	0.66	0.32			H1				8 altered motifs	PLCB1	intronic
20	8844495	1	1	rs147271284	7-mer	A	0.14	0.44	0.66	0.32							8 altered motifs	PLCB1	intronic
20	8844694	1	1	rs6086662	T	C	0.15	0.44	0.66	0.32							DMRT2,Irf	PLCB1	intronic

20	8845050	0.89	1	rs6108204	A	G	0.15	0.44	0.69	0.32							4 altered motifs	PLCB1	intronic
20	8845067	0.89	1	rs6086663	G	T	0.14	0.44	0.69	0.32							6 altered motifs	PLCB1	intronic
20	8845519	1	1	rs6086664	T	A	0.08	0.44	0.66	0.32							LXR	PLCB1	intronic
20	8845933	0.96	1	rs6086665	G	A	0.09	0.44	0.65	0.32							12 altered motifs	PLCB1	intronic
20	8845937	0.96	1	rs6086666	G	A	0.16	0.44	0.65	0.31							Dmbx1	PLCB1	intronic
20	8846337	0.94	1	rs6140780	G	A	0.08	0.44	0.65	0.32							6 altered motifs	PLCB1	intronic
20	8846404	0.94	1	rs8182968	G	A	0.08	0.44	0.65	0.32							4 altered motifs	PLCB1	intronic
20	8846489	0.94	1	rs8182972	G	A	0.08	0.44	0.65	0.32							LBP-1	PLCB1	intronic
20	8846656	0.94	1	rs6086667	G	C	0.16	0.44	0.65	0.32							ATF3,STAT	PLCB1	intronic
20	8846739	0.94	1	rs6086668	A	G	0.16	0.44	0.65	0.32				Fibrobl			Dobox4,Mef2,STAT	PLCB1	intronic
20	8846835	0.94	1	rs6086669	G	T	0.16	0.44	0.65	0.32							7 altered motifs	PLCB1	intronic
20	8846925	0.94	1	rs6140781	G	A	0.16	0.44	0.65	0.31				FibroP				PLCB1	intronic
20	8846986	0.94	1	rs6086670	T	C	0.16	0.44	0.65	0.32				FibroP,HFF-Myc			CEBPB,Osf2	PLCB1	intronic
20	8847252	0.94	1	rs6077444	G	A	0.16	0.44	0.65	0.32							4 altered motifs	PLCB1	intronic
20	8847311	0.94	1	rs6086671	G	C	0.16	0.44	0.65	0.32							SEF-1	PLCB1	intronic
20	8847414	0.94	1	rs6086672	G	A	0.16	0.44	0.65	0.32			HSMM					PLCB1	intronic
20	8847727	0.94	1	rs3891453	A	G	0.16	0.44	0.65	0.32			HSMM					PLCB1	intronic
20	8848529	0.94	1	rs6077445	A	G	0.16	0.44	0.65	0.32							5 altered motifs	PLCB1	intronic
20	8848688	0.94	1	rs3915507	A	G	0.16	0.44	0.65	0.31							EBF	PLCB1	intronic
20	8848724	0.94	1	rs3848829	T	C	0.15	0.44	0.65	0.31							4 altered motifs	PLCB1	intronic
20	8848994	0.94	1	rs3848830	C	T	0.16	0.44	0.65	0.31							Sox	PLCB1	intronic
20	8849379	0.91	1	rs4816099	A	T	0.16	0.43	0.64	0.3							HNF4,Nkx2,TFE	PLCB1	intronic
20	8849390	0.94	1	rs4813878	C	T	0.16	0.44	0.65	0.3							SETDB1	PLCB1	intronic
20	8849910	0.94	1	rs2327119	C	A	0.16	0.44	0.65	0.31							Pou2f2,Zfp105	PLCB1	intronic
20	8851321	0.93	0.99	rs3848831	T	C	0.16	0.44	0.65	0.31							Foxc1,Maf,Pou2f2	PLCB1	intronic

Query SNP: rs1957895 and variants with r^2 >= 0.8

chr	pos (hg19)	LD (r²)	LD (D')	variant	Ref	Alt	AFR freq	AMR freq	ASN freq	EUR freq	SiPhy cons	Promoter histone marks	Enhancer histone marks	DNAse	Proteins bound	eQTL tissues	Motifs changed	GENCODE genes	dbSNP func annot
14	61907407	0.94	0.99	rs4636825	C	G	0.27	0.89	0.64	0.91			Huvec, GM12878				GR,p300	PRKCH	intronic
14	61908076	0.99	1	rs1957893	T	C	0.23	0.89	0.63	0.92			GM12878				VDR	PRKCH	intronic
14	61908111	0.99	1	rs1957894	T	G	0.23	0.89	0.63	0.92			GM12878	Fibrobl			Mrg,Pbx3,Tgif1	PRKCH	intronic
14	61908332	1	1	rs1957895	G	T	0.22	0.89	0.63	0.92			GM12878				4 altered motifs	PRKCH	intronic
14	61909343	1	1	rs4899048	A	G	0.22	0.89	0.63	0.92			GM12878				Nanog,Pou2f2	PRKCH	intronic
14	61911157	1	1	rs10144353	T	C	0.2	0.88	0.63	0.92			GM12878	19 cell types	6 bound proteins		8 altered motifs	PRKCH	intronic
14	61912138	0.81	1	rs927686	C	T	0.18	0.88	0.58	0.92							Cdx2,Hoxb8	PRKCH	intronic

Query SNP: rs12525170 and variants with r^2 >= 0.8

chr	pos (hg19)	LD (r²)	LD (D')	variant	Ref	Alt	AFR freq	AMR freq	ASN freq	EUR freq	SiPhy cons	Promoter histone marks	Enhancer histone marks	DNAse	Proteins bound	eQTL tissues	Motifs changed	GENCODE genes	dbSNP func annot
6	31094971	0.98	1	rs10947137	A	C	0.01	0.08	0.1	0.08			HSMM, K562, HMEC				Rad21,SMC3	PSORS1C1	intronic
6	31099577	1	1	rs4959053	G	A	0.01	0.07	0.09	0.08			H1	H7-hESC	POU5F1			PSORS1C1	intronic
6	31099761	1	1	rs12525170	G	A	0.01	0.07	0.09	0.08							TBX5	PSORS1C1	intronic
6	31102273	0.8	1	rs75881311	T	A	0	0	0.08	0.01							SZF1-1,Spz1	PSORS1C1	intronic

Query SNP: rs7742033 and variants with r^2 >= 0.8

chr	pos (hg19)	LD (r²)	LD (D')	variant	Ref	Alt	AFR freq	AMR freq	ASN freq	EUR freq	SiPhy cons	Promoter histone marks	Enhancer histone marks	DNAse	Proteins bound	eQTL tissues	Motifs changed	GENCODE genes	dbSNP func annot
6	30837290	1	1	rs114059502	C	T	0.05	0.07	0	0.02				H7-hESC,SK-N-MC	SETDB1		Foxa,Smad	DDR1-AS1	
6	30837370	1	1	rs56051116	C	T	0	0.01	0	0				SK-N-MC	SETDB1		4 altered motifs	DDR1-AS1	
6	30838912	1	1	rs115921597	C	T	0	0.01	0	0							Egr-1	DDR1-AS1	
6	30840835	1	1	rs56377503	A	C	0.02	0.02	0	0.01			K562	14 cell types	13 bound proteins		18 altered motifs	DDR1-AS1	
6	30870904	1	1	rs116832402	A	G	0	0.01	0	0			GM12878				Ets,Ncx	3kb 3' of DDR1	
6	30905836	1	1	rs116363553	C	T	0.01	0.01	0	0				Fibrobl,H9ES			5 altered motifs	2.9kb 5' of DPCR1	
6	30933492	1	1	rs114000484	C	T	0.01	0.01	0	0			GM12878				4 altered motifs	11kb 5' of HCG21	
6	30933496	1	1	rs56804692	T	C	0.06	0.01	0	0.01			GM12878				4 altered motifs	11kb 5' of HCG21	
6	30935531	1	1	rs9468853	C	T	0.05	0.01	0	0.01							Foxp3,HNF4,Zbtb3	13kb 5' of HCG21	
6	30941209	1	1	rs115589984	G	A	0.05	0.02	0	0.01							6 altered motifs	10kb 5' of MUC21	
6	30952379	1	1	rs148257260	G	A	0.05	0.01	0	0.01							6 altered motifs	MUC21	intronic
6	30955717	1	1	rs45565239	G	A	0.07	0.01	0	0.01				HeLa-S3,Th1			Egr-1	MUC21	intronic
6	30959280	1	1	rs138480883	G	A	0.06	0.01	0	0.01							PLAG1,RXRA	1.6kb 3' of MUC21	
6	30959500	1	1	rs147171413	C	T	0.06	0.01	0	0.01							BATF,Irf	1.8kb 3' of MUC21	
6	30963143	1	1	rs9468857	A	C,G,T	0.06	0.01	0	0.01								5.5kb 3' of MUC21	
6	30965190	1	1	rs114891374	A	C	0.03	0	0	0.01				CD34+_Mobilized			6 altered motifs	7.5kb 3' of MUC21	
6	30966789	1	1	rs114982948	G	A	0.03	0	0	0.01			HMEC, HSMM	17 cell types	5 bound proteins		4 altered motifs	9.1kb 3' of MUC21	

chr	pos (hg19)	LD (r²)	LD (D')	variant	Ref	Alt	AFR freq	AMR freq	ASN freq	EUR freq	SiPhy cons	Promoter histone marks	Enhancer histone marks	DNAse	Proteins bound	eQTL tissues	Motifs changed	GENCODE genes	dbSNP func annot
6	30969347	1	1	rs138565013	C	A	0.02	0	0	0.01			K562		POL24H8		5 altered motifs	8.9kb 5' of MUC22	
6	30969781	1	1	rs201741822	CCA	C	0.02	0	0	0.01			K562		POL24H8		4 altered motifs	8.5kb 5' of MUC22	
6	30973650	1	1	rs56111713	G	T	0.03	0	0	0.01			GM12878	GM12864,GM12865	MEF2A		12 altered motifs	4.6kb 5' of MUC22	
6	30978328	1	1	rs56327229	C	T	0.03	0	0	0.01			NHEK, HMEC				Foxd1	MUC22	intronic
6	30983895	1	1	rs56183784	G	A	0.03	0	0	0.01				Osteobl			5 altered motifs	MUC22	intronic
6	30989791	1	1	rs144797308	C	T	0.03	0	0	0			K562	Jurkat	POL2,POL24H8		HDAC2,STAT,Sox	MUC22	intronic
6	30990631	1	1	rs115428009	G	A	0.03	0	0	0			K562				ERalpha-a	MUC22	intronic
6	30992353	1	1	rs147941707	C	T	0.04	0.01	0	0							Maf,THAP1	MUC22	intronic
6	31028132	1	1	rs147277589	T	G	0	0	0	0			HMEC	HPDE6-E6E7,HRE				464bp 3' of HCG22	
6	31085226	1	1	rs7742033	G	A	0.05	0.01	0	0		NHEK	4 cell types	Huh-7,PanIsletD,T-47D			4 altered motifs	CDSN	missense
6	31123723	1	1	rs56290415	T	C	0.01	0.01	0	0							20 altered motifs	CCHCR1	intronic
6	31323736	1	1	rs41552112	T	A	0.01	0.01	0	0		6 cell types	HMEC	K562			NF-kappaB,PU.1,STAT	HLA-B	intronic

Query SNP: rs7574070 and variants with r² >= 0.8

chr	pos (hg19)	LD (r²)	LD (D')	variant	Ref	Alt	AFR freq	AMR freq	ASN freq	EUR freq	SiPhy cons	Promoter histone marks	Enhancer histone marks	DNAse	Proteins bound	eQTL tissues	Motifs changed	GENCODE genes	dbSNP func annot
2	192010488	1	1	rs7574070	A	C	0.29	0.68	0.43	0.64			NHEK				DMRT1	STAT4	intronic
2	192015072	0.98	0.99	rs7572482	A	G	0.33	0.69	0.43	0.64		8 cell types		6 cell types	EBF1		E2F,Pou5f1,Sox	STAT4	intronic
2	192016879	0.97	0.99	rs11684030	G	A	0.23	0.68	0.43	0.63			GM12878				Mrg,Tgif1	556bp 5' of STAT4	
2	192017771	0.98	0.99	rs897200	T	C	0.3	0.68	0.43	0.63			5 cell types	63 cell types	PU1,NRSF		Foxc1,Maf,Nkx3	1.4kb 5' of STAT4	
2	192017989	0.98	0.99	rs897199	T	A	0.31	0.68	0.43	0.63				Hepatocytes			8 altered motifs	1.7kb 5' of STAT4	
2	192018007	0.98	0.99	rs897198	C	T	0.31	0.68	0.43	0.63				Hepatocytes			Foxp1,GR,Pitx2	1.7kb 5' of STAT4	
2	192018417	0.98	0.99	rs1598253	T	C	0.23	0.67	0.43	0.63							CEBPB,GATA,Irf	2.1kb 5' of STAT4	
2	192019119	0.98	0.99	rs55925192	G	A	0.35	0.68	0.43	0.63							Foxa,GR	2.8kb 5' of STAT4	
2	192019372	0.98	0.99	rs16833437	T	G	0.19	0.67	0.43	0.63								3kb 5' of STAT4	
2	192019546	0.97	0.99	rs7561569	C	A	0.19	0.67	0.43	0.63				Th1			9 altered motifs	3.2kb 5' of STAT4	
2	192020618	0.98	0.99	rs1031507	A	C	0.35	0.69	0.43	0.64				22 cell types	4 bound proteins		4 altered motifs	4.3kb 5' of STAT4	
2	192023568	0.85	0.98	rs75066818	TC	T	0.3	0.67	0.46	0.65			HSMM				9 altered motifs	7.2kb 5' of STAT4	
2	192023708	0.94	0.97	rs6736458	A	G	0.31	0.68	0.43	0.63			HSMM	11 cell types			Pou2f2,TCF12	7.4kb 5' of STAT4	
2	192024550	0.94	0.97	rs16833453	T	C	0.35	0.68	0.43	0.63							Rad21	8.2kb 5' of STAT4	
2	192024985	0.94	0.97	rs57081321	T	C	0.35	0.68	0.43	0.63								8.7kb 5' of STAT4	
2	192025764	0.94	0.97	rs10931485	T	C	0.35	0.68	0.43	0.63							Ets	9.4kb 5' of STAT4	
2	192025838	0.94	0.97	rs10931486	C	T	0.3	0.67	0.43	0.63							NRSF,TATA	9.5kb 5' of STAT4	
2	192026200	0.94	0.97	rs7567778	G	A	0.31	0.68	0.43	0.63							EBF	9.9kb 5' of STAT4	
2	192028387	0.88	0.97	rs36181338	G	A	0.34	0.67	0.42	0.6							GR,Myf	12kb 5' of STAT4	

Query SNP: rs17882828 and variants with r² >= 0.8

chr	pos (hg19)	LD (r²)	LD (D')	variant	Ref	Alt	AFR freq	AMR freq	ASN freq	EUR freq	SiPhy cons	Promoter histone marks	Enhancer histone marks	DNAse	Proteins bound	eQTL tissues	Motifs changed	GENCODE genes	dbSNP func annot
1	92163682	1	1	rs17882828	C	G	0	0.02	0.04	0			NHLF, GM12878, K562	24 cell types	CEBPB		Foxa,HNF4	TGFBR3	missense
1	92170554	0.96	1	rs3767572	A	G	0	0.02	0.04	0			K562				AFP1	TGFBR3	intronic

Query SNP: rs284148 and variants with r² >= 0.8

chr	pos (hg19)	LD (r²)	LD (D')	variant	Ref	Alt	AFR freq	AMR freq	ASN freq	EUR freq	SiPhy cons	Promoter histone marks	Enhancer histone marks	DNAse	Proteins bound	eQTL tissues	Motifs changed	GENCODE genes	dbSNP func annot
1	92271920	0.87	1	rs284157	C	T	0.23	0.44	0.42	0.4			NHLF, Huvec	9 cell types				TGFBR3	intronic
1	92273253	0.87	1	rs284156	C	G	0.23	0.44	0.42	0.4			4 cell types				5 altered motifs	TGFBR3	intronic
1	92275672	1	1	rs284151	A	G	0.09	0.11	0.39	0.09			NHLF					TGFBR3	intronic
1	92275741	1	1	rs284150	G	A	0.14	0.11	0.39	0.09			NHLF				Ik-1	TGFBR3	intronic
1	92277843	1	1	rs284148	C	T	0.15	0.11	0.39	0.09							PLAG1	TGFBR3	intronic
1	92279583	1	1	rs284146	T	C	0.16	0.11	0.39	0.09			Huvec, H1	H7-hESC,SK-N-MC			6 altered motifs	TGFBR3	intronic
1	92280928	1	1	rs2489178	G	A	0.16	0.11	0.39	0.09			Huvec	LNCaP			8 altered motifs	TGFBR3	intronic
1	92283077	1	1	rs449960	T	A	0.16	0.12	0.39	0.09			HepG2, NHLF				E2F,Foxk1,Msx-1	TGFBR3	intronic
1	92283899	0.89	-0.98	rs10874980	A	G	0.8	0.85	0.59	0.88			5 cell types	20 cell types	MAFF,MAFK		E2F,Maf,TCF12	TGFBR3	intronic
1	92284920	1	1	rs2489176	G	A	0.11	0.11	0.39	0.09			NHLF, HepG2				Foxm1,Ik-2,Smad	TGFBR3	intronic
1	92285839	1	1	rs2799539	A	G	0.13	0.11	0.39	0.09			HepG2	4 cell types	CEBPB,JUND		8 altered motifs	TGFBR3	intronic
1	92286940	0.99	1	rs1032255	C	T	0.1	0.11	0.39	0.09			K562				4 altered motifs	TGFBR3	intronic
1	92290111	1	1	rs2634036	T	C	0.11	0.11	0.39	0.09			HepG2				Hbp1	TGFBR3	intronic
1	92290285	0.96	1	rs2265863	G	A	0.1	0.11	0.38	0.1			HepG2				4 altered motifs	TGFBR3	intronic
1	92292407	1	-1	rs901917	C	T	0.83	0.89	0.61	0.91			HepG2, NHLF				9 altered motifs	TGFBR3	intronic
1	92292609	1	-1	rs901915	C	A	0.83	0.89	0.61	0.91			HepG2, NHLF		FOXA1,POL2		Cphx,Pbx-1	TGFBR3	intronic
1	92292629	1	-1	rs901914	G	C	0.83	0.89	0.61	0.91			HepG2, NHLF, NHEK		FOXA1,POL2		Maf,NF-AT	TGFBR3	intronic
1	92292765	1	-1	rs2391070	A	G	0.82	0.88	0.61	0.91			HepG2, NHLF, NHEK	19 cell types	10 bound proteins		Hic1,TCF4	TGFBR3	intronic
1	92293162	1	1	rs901913	G	T	0.13	0.11	0.39	0.09			4 cell types	A549,HepG2	5 bound proteins		Pou2f2	TGFBR3	intronic
1	92294660	0.99	1	rs2256471	C	T	0.09	0.11	0.39	0.09			HepG2, NHLF	PrEC			HES1,TATA	TGFBR3	intronic

1	92294703	0.91	1	rs2489169	T	C	0.13	0.1	0.37	0.09			HepG2, NHLF				6 altered motifs	TGFBR3	intronic
1	92295724	1	1	rs717923	A	G	0.14	0.11	0.39	0.09			4 cell types					TGFBR3	intronic
1	92297055	1	1	rs2489186	C	T	0.11	0.11	0.39	0.09			HepG2, NHLF				AIRE,Foxq1, RREB-1	TGFBR3	intronic
1	92297887	0.86	-0.99	rs11165578	C	A	0.77	0.56	0.58	0.6			NHLF, HepG2, NHEK				CDP,Sox	TGFBR3	intronic
1	92297986	0.94	0.99	rs2489185	T	A	0.09	0.11	0.38	0.09			NHLF, HepG2, NHEK	SAEC			4 altered motifs	TGFBR3	intronic
1	92298343	0.94	0.99	rs2489184	T	C	0.11	0.11	0.38	0.1			HepG2				8 altered motifs	TGFBR3	intronic
1	92300631	0.99	0.99	rs2634034	A	C	0.14	0.11	0.39	0.09			HepG2		HNF4G		5 altered motifs	TGFBR3	intronic
1	92301182	0.96	0.99	rs2810903	G	A	0.1	0.11	0.38	0.09			NHLF, HepG2				Nr2f2	TGFBR3	intronic
1	92301580	0.99	0.99	rs3103333	G	A	0.14	0.12	0.39	0.09			HepG2				LXR,Sp4	TGFBR3	intronic
1	92302296	0.97	0.99	rs3103332	T	C	0.11	0.11	0.39	0.09			HepG2				4 altered motifs	TGFBR3	intronic
1	92304823	0.96	0.98	rs2087300	A	G	0.1	0.11	0.39	0.09			HepG2				Klf4	TGFBR3	intronic
1	92305082	0.96	0.98	rs2129973	C	T	0.1	0.11	0.39	0.09			HepG2	8988T			ATF3	TGFBR3	intronic
1	92305250	0.96	0.98	rs2129972	C	T	0.15	0.11	0.39	0.09			HepG2	WI-38			4 altered motifs	TGFBR3	intronic
1	92305534	0.96	0.98	rs78781288	GT	G	0.16	0.11	0.39	0.09			HepG2				4 altered motifs	TGFBR3	intronic
1	92306976	0.96	0.98	rs2799522	C	G	0.22	0.11	0.39	0.09			NHEK, NHLF, K562	28 cell types			5 altered motifs	TGFBR3	intronic
1	92307362	0.85	0.96	rs2799521	A	G	0.11	0.11	0.37	0.09			NHLF				6 altered motifs	TGFBR3	intronic
1	92308567	0.96	0.98	rs2799547	C	T	0.19	0.11	0.39	0.09							7 altered motifs	TGFBR3	intronic
1	92309634	0.96	0.98	rs2634033	G	A	0.11	0.11	0.39	0.09			HepG2, NHEK, NHLF					TGFBR3	intronic
1	92309916	0.96	0.98	rs2799512	T	C	0.19	0.11	0.39	0.09			HepG2, NHEK, NHLF		SETDB1		Foxl1	TGFBR3	intronic
1	92312060	0.96	0.98	rs1352438	G	C	0.19	0.11	0.39	0.09			4 cell types					TGFBR3	intronic
1	92312595	0.96	0.98	rs2634032	A	G	0.19	0.11	0.39	0.09			NHLF, HepG2					TGFBR3	intronic
1	92312764	0.96	0.98	rs2799516	G	A	0.19	0.11	0.39	0.09			HepG2, NHLF				Foxl1,Nkx6-2,TATA	TGFBR3	intronic
1	92313391	0.96	0.98	rs2799517	C	T	0.1	0.11	0.39	0.09			HepG2				12 altered motifs	TGFBR3	intronic

Query SNP: rs1805110 and variants with r^2 >= 0.8

chr	pos (hg19)	LD (r^2)	LD (D')	variant	Ref	Alt	AFR freq	AMR freq	ASN freq	EUR freq	SiPhy cons	Promoter histone marks	Enhancer histone marks	DNAse	Proteins bound	eQTL tissues	Motifs changed	GENCODE genes	dbSNP func annot
1	92316682	0.91	0.98	rs2799523	C	T	0.16	0.1	0.41	0.09			5 cell types	HMVEC-LLy,HMVEC-dLy-Ad			4 altered motifs	TGFBR3	intronic
1	92317312	0.92	0.98	rs2799524	C	T	0.25	0.11	0.41	0.09			6 cell types	Osteobl,PANC-1	ZNF263,KAP1,SETDB1		7 altered motifs	TGFBR3	intronic
1	92317891	0.92	0.98	rs2634030	T	C	0.16	0.11	0.41	0.09			HepG2, NHLF	GM12892			CEBPG,Hsf, STAT	TGFBR3	intronic
1	92318550	0.92	0.98	rs140625227	GA	G	0.16	0.11	0.41	0.09							5 altered motifs	TGFBR3	intronic
1	92318581	0.92	0.98	rs2799525	C	T	0.16	0.11	0.41	0.09							4 altered motifs	TGFBR3	intronic
1	92318614	0.92	0.98	rs2634029	T	A	0.16	0.11	0.41	0.09							8 altered motifs	TGFBR3	intronic
1	92319577	0.92	-0.97	rs2799526	G	A	0.82	0.89	0.57	0.9							7 altered motifs	TGFBR3	intronic
1	92320257	0.97	0.99	rs2489171	A	G	0.16	0.11	0.42	0.09							Foxp1,GR	TGFBR3	intronic
1	92320260	0.97	0.99	rs2450927	T	C	0.16	0.11	0.42	0.09							ATF3,Foxp1, GR	TGFBR3	intronic
1	92320270	0.97	0.99	rs2489170	A	G	0.16	0.11	0.42	0.09							8 altered motifs	TGFBR3	intronic
1	92320853	0.97	0.99	rs2634028	G	T	0.16	0.11	0.42	0.09								TGFBR3	intronic
1	92321468	0.96	0.99	rs2634027	T	C	0.2	0.12	0.42	0.09							4 altered motifs	TGFBR3	intronic
1	92321512	0.96	0.99	rs2450926	A	G	0.33	0.12	0.42	0.09							GR	TGFBR3	intronic
1	92321813	0.98	0.99	rs2634026	C	T	0.19	0.12	0.42	0.09								TGFBR3	intronic
1	92321913	0.98	0.99	rs2799514	A	G	0.19	0.12	0.42	0.09							11 altered motifs	TGFBR3	intronic
1	92321974	0.99	0.99	rs2634025	C	T	0.19	0.12	0.42	0.09							4 altered motifs	TGFBR3	intronic
1	92322301	0.99	0.99	rs2799515	G	A	0.19	0.12	0.42	0.09							RXRA	TGFBR3	intronic
1	92323724	0.99	0.99	rs2634024	A	C	0.19	0.12	0.42	0.09							Pou2f2	TGFBR3	intronic
1	92323809	0.98	-0.99	rs11576557	T	C	0.46	0.83	0.58	0.89							AIRE,Znf143	TGFBR3	intronic
1	92324150	0.97	0.99	rs2634023	C	T	0.19	0.12	0.42	0.09							AP-1,GR	TGFBR3	intronic
1	92324968	0.91	0.96	rs113558784	CT	C	0.19	0.11	0.42	0.09		GM12878		GM19240			5 altered motifs	TGFBR3	intronic
1	92325802	1	1	rs2489192	A	G	0.19	0.11	0.42	0.09							Pou1f1,Pou2f2,Zfp105	TGFBR3	intronic
1	92326297	1	1	rs1906586	T	C	0.19	0.11	0.42	0.09							GCM	TGFBR3	intronic
1	92326636	1	1	rs2046737	C	T	0.19	0.11	0.42	0.09							Bcl6b,Foxd3, Maf	TGFBR3	intronic
1	92326781	1	1	rs2634022	A	G	0.19	0.11	0.42	0.09							GATA,NF-AT	TGFBR3	intronic
1	92327045	1	1	rs1805110	G	A	0.19	0.11	0.42	0.09							Osr	TGFBR3	missense
1	92327126	1	1	rs1805109	C	T	0.16	0.11	0.42	0.09								TGFBR3	5'-UTR
1	92327701	1	1	rs2634020	A	G	0.19	0.11	0.42	0.09							12 altered motifs	TGFBR3	intronic
1	92327877	1	1	rs2634019	A	T	0.19	0.11	0.42	0.09							FAC1,Gfi1b, RREB-1	TGFBR3	intronic
1	92331736	0.81	0.91	rs2489189	T	A	0.16	0.1	0.43	0.06							7 altered motifs	TGFBR3	intronic
1	92332527	0.8	0.91	rs1488692	C	T	0.16	0.1	0.43	0.06			NHEK	Medullo,NHDF-Ad,WERI			Smad4,Smad	TGFBR3	intronic

Query SNP: rs150474359 and variants with r^2 >= 0.8

chr	pos (hg19)	LD (r^2)	LD (D')	variant	Ref	Alt	AFR freq	AMR freq	ASN freq	EUR freq	SiPhy cons	Promoter histone marks	Enhancer histone marks	DNAse	Proteins bound	eQTL tissues	Motifs changed	GENCODE genes	dbSNP func annot
9	34107356	1	1	rs150474359	C	T											Hltf,Pax-4	DCAF12	splice acceptor

Query SNP: rs10758242 and variants with r^2 >= 0.8

chr	pos (hg19)	LD (r^2)	LD (D')	variant	Ref	Alt	AFR freq	AMR freq	ASN freq	EUR freq	SiPhy cons	Promoter histone marks	Enhancer histone marks	DNAse	Proteins bound	eQTL tissues	Motifs changed	GENCODE genes	dbSNP func annot
9	34081235	0.8	0.91	rs7034119	T	C	0.62	0.62	0.3	0.69			K562				5 altered motifs	3.1kb 5' of RP11-537H15.3	
9	34082144	0.8	0.91	rs10441723	T	C	0.57	0.61	0.3	0.69							AIRE,AP-3,Alx4	2.2kb 5' of RP11-537H15.3	
9	34084485	0.8	0.91	rs7031060	C	G	0.62	0.62	0.3	0.68			HSMM	Th1			GCM,GR	RP11-537H15.3	
9	34087360	0.8	0.91	rs1052656	T	C	0.58	0.61	0.3	0.68			HSMM				Myc	RP11-537H15.3	3'-UTR
9	34093090	0.81	0.92	rs10971909	A	G	0.57	0.7	0.3	0.75							ELF1,Myc,NRSF	RP11-537H15.3	intronic
9	34096597	0.81	0.92	rs7857551	G	A	0.58	0.7	0.3	0.75								RP11-537H15.3	intronic
9	34098110	0.81	0.92	rs10758240	T	C	0.68	0.71	0.3	0.75			GM12878	7 cell types	EBF1,NFKB			DCAF12	intronic
9	34100917	0.81	0.92	rs1980887	A	G	0.58	0.7	0.3	0.75							TBX5	DCAF12	intronic
9	34107232	0.81	0.92	rs10511914	T	C	0.58	0.7	0.3	0.75				Melano				DCAF12	intronic
9	34110846	0.84	0.93	rs10971920	C	G	0.58	0.7	0.3	0.75				Th1				DCAF12	intronic
9	34111261	0.84	0.93	rs12551419	T	C	0.63	0.71	0.3	0.75				Th1			E2A,TBX5,ZEB1	DCAF12	intronic
9	34113794	0.84	0.93	rs10971924	G	A	0.63	0.71	0.3	0.75							9 altered motifs	DCAF12	intronic
9	34115076	0.84	0.93	rs11788468	C	T	0.58	0.7	0.3	0.75							ERalpha-a	DCAF12	intronic
9	34124860	0.88	0.95	rs2275003	A	G	0.35	0.49	0.29	0.53		4 cell types	4 cell types				HDAC2,Ik-2	DCAF12	intronic
9	34129839	0.91	0.96	rs10814070	T	A	0.76	0.7	0.3	0.74							5 altered motifs	2.4kb 5' of DCAF12	
9	34146776	1	1	rs10758242	G	A	0.65	0.7	0.3	0.75					GATA3		AP-1,RP58	19kb 5' of DCAF12	
9	34147194	0.82	0.97	rs10971954	A	C	0.39	0.6	0.27	0.64							10 altered motifs	20kb 5' of DCAF12	
9	34148794	0.98	0.99	rs4879755	G	C	0.73	0.7	0.3	0.75			K562		CTCF		Nr2f2,Pax-3	21kb 5' of DCAF12	
9	34149119	0.98	0.99	rs10758243	G	C	0.65	0.7	0.3	0.75			K562				Foxo,Pax-4,RREB-1	22kb 5' of DCAF12	
Query SNP: rs16830589 and variants with r^2 >= 0.8																			
chr	pos (hg19)	LD (r^2)	LD (D')	variant	Ref	Alt	AFR freq	AMR freq	ASN freq	EUR freq	SiPhy cons	Promoter histone marks	Enhancer histone marks	DNAse	Proteins bound	eQTL tissues	Motifs changed	GENCODE genes	dbSNP func annot
3	159344907	0.83	0.95	rs4494974	G	C	0.61	0.05	0.17	0.01							5 altered motifs	IQCJ-SCHIP1	intronic
3	159347475	0.83	0.95	rs1437520	A	T	0.61	0.05	0.17	0.01			NHLF				6 altered motifs	IQCJ-SCHIP1	intronic
3	159347730	0.83	0.95	rs9882328	C	T	0.61	0.05	0.17	0.01			NHLF, HSMM				Nanog,Sox	IQCJ-SCHIP1	intronic
3	159348014	0.83	0.95	rs9848763	A	C	0.61	0.05	0.17	0.01				HNPCEpiC, HPdLF			8 altered motifs	IQCJ-SCHIP1	intronic
3	159348112	0.83	0.95	rs9848897	A	T	0.66	0.05	0.17	0.02				HNPCEpiC, HPdLF			7 altered motifs	IQCJ-SCHIP1	intronic
3	159349481	0.83	0.95	rs1816621	C	T	0.61	0.05	0.17	0.02			NHEK, HMEC				5 altered motifs	IQCJ-SCHIP1	intronic
3	159349702	0.83	0.95	rs1347671	A	G	0.48	0.04	0.17	0.01			HMEC				7 altered motifs	IQCJ-SCHIP1	intronic
3	159352025	0.91	0.96	rs78881494	A	G	0.42	0.04	0.16	0.02			NHLF, HSMM				5 altered motifs	IQCJ-SCHIP1	intronic
3	159352143	0.91	0.96	rs9832970	G	A	0.42	0.04	0.16	0.02			NHLF, HMEC, HSMM				DMRT2,Nanog,Pou2f2	IQCJ-SCHIP1	intronic
3	159352596	0.83	0.96	rs77954255	C	G	0.01	0.01	0.15	0.01			5 cell types	HA-sp	KAP1		7 altered motifs	IQCJ-SCHIP1	intronic
3	159353144	0.91	0.96	rs28434644	T	C	0.54	0.04	0.16	0.05			4 cell types		GR,POL2		Ets,Pax-5	IQCJ-SCHIP1	intronic
3	159354028	0.92	0.96	rs1962890	G	A	0.6	0.04	0.16	0.04							ZBTB33	IQCJ-SCHIP1	intronic
3	159355906	0.92	0.96	rs11921824	T	C	0.56	0.04	0.16	0.02			4 cell types	39 cell types	4 bound proteins		LF-A1	IQCJ-SCHIP1	intronic
3	159355918	0.92	0.96	rs11921827	T	C	0.48	0.04	0.16	0.02			4 cell types	25 cell types	4 bound proteins		AP-1,Gfi1	IQCJ-SCHIP1	intronic
3	159356482	0.85	0.96	rs9826417	C	G	0.45	0.04	0.15	0.02			HMEC, HSMM				Nkx3	IQCJ-SCHIP1	intronic
3	159357047	0.82	0.93	rs16830546	T	C	0.08	0.01	0.15	0.01								IQCJ-SCHIP1	intronic
3	159357525	0.92	0.96	rs9851700	T	C	0.48	0.04	0.16	0.02							LUN-1	IQCJ-SCHIP1	intronic
3	159358778	0.92	0.96	rs750688	C	T	0.5	0.04	0.16	0.02							5 altered motifs	IQCJ-SCHIP1	intronic
3	159359371	0.85	0.96	rs9842465	G	T	0.45	0.04	0.15	0.02							HMG-IY,Irf,p300	IQCJ-SCHIP1	intronic
3	159359429	0.92	0.96	rs9879792	A	C	0.5	0.04	0.16	0.02								IQCJ-SCHIP1	intronic
3	159359872	0.92	0.96	rs9809076	A	C	0.57	0.04	0.16	0.02							HNF1,OTX,Pou3f2	IQCJ-SCHIP1	intronic
3	159360233	0.92	0.96	rs9867212	T	C	0.5	0.04	0.16	0.02							AhR::Arnt,Arnt	IQCJ-SCHIP1	intronic
3	159361672	0.92	0.96	rs16830575	A	G	0.48	0.04	0.16	0.01							4 altered motifs	IQCJ-SCHIP1	intronic
3	159362244	0.93	0.97	rs16830579	G	A	0.48	0.04	0.16	0.02							GATA	IQCJ-SCHIP1	intronic
3	159362915	0.96	1	rs16830581	C	G	0.5	0.04	0.15	0.02							7 altered motifs	IQCJ-SCHIP1	intronic
3	159364557	1	1	rs9290046	T	C	0.52	0.04	0.16	0.02							SIX5	IQCJ-SCHIP1	intronic
3	159364915	1	1	rs9290047	C	T	0.52	0.04	0.16	0.02							En-1,RORalpha1	IQCJ-SCHIP1	intronic
3	159365432	1	1	rs16830589	T	C	0.52	0.04	0.16	0.02							GATA,Hoxa5	IQCJ-SCHIP1	intronic
3	159366426	0.99	1	rs77414618	C	G	0.52	0.04	0.16	0.02							Pax-4,RREB-1,Zfp740	IQCJ-SCHIP1	intronic
3	159367303	0.99	1	rs1347672	C	A	0.55	0.04	0.16	0.02							E2F,YY1,p300	IQCJ-SCHIP1	intronic
3	159367346	0.92	1	rs1816622	G	A	0.46	0.04	0.15	0.02							8 altered motifs	IQCJ-SCHIP1	intronic
3	159367925	0.91	1	rs74550897	A	T	0.46	0.04	0.15	0.02							Foxq1,TATA	IQCJ-SCHIP1	intronic
3	159368581	0.97	1	rs1978830	T	C	0.52	0.04	0.15	0.02			NHEK	4 cell types			Hoxa9,Hoxd10	IQCJ-SCHIP1	intronic
3	159368828	0.97	1	rs2886621	C	T	0.53	0.04	0.15	0.02				RWPE1,SAEC				IQCJ-SCHIP1	intronic
3	159369276	0.97	1	rs9829310	A	G	0.52	0.04	0.15	0.02							Foxa,GR	IQCJ-SCHIP1	intronic
3	159369874	0.91	1	rs79167961	C	T	0.09	0.01	0.15	0.01							4 altered motifs	IQCJ-SCHIP1	intronic
3	159371068	0.97	1	rs9877425	C	A	0.51	0.04	0.15	0.02								IQCJ-SCHIP1	intronic
3	159372107	0.97	1	rs6772245	A	T	0.59	0.04	0.15	0.02				5 cell types	4 bound proteins		4 altered motifs	IQCJ-SCHIP1	intronic
3	159372345	0.91	1	rs9883498	G	A	0.51	0.04	0.15	0.02								IQCJ-SCHIP1	intronic
3	159372637	0.91	1	rs13434009	A	C	0.51	0.04	0.15	0.02				SK-N-SH_RA			TAL1	IQCJ-SCHIP1	intronic

3	159373158	0.9	1	rs79347224	C	T	0.07	0.01	0.14	0.01							Sox	IQCJ-SCHIP1	intronic

Query SNP: rs16830581
and variants with r^2 >= 0.8

chr	pos (hg19)	LD (r²)	LD (D')	variant	Ref	Alt	AFR freq	AMR freq	ASN freq	EUR freq	SiPhy cons	Promoter histone marks	Enhancer histone marks	DNAse	Proteins bound	eQTL tissues	Motifs changed	GENCODE genes	dbSNP func annot
3	159332305	0.81	0.96	rs12107648	C	T	0.61	0.05	0.17	0.01							NRSF,Rhox11,Sin3Ak-20	IQCJ-SCHIP1	intronic
3	159333379	0.81	0.96	rs1343824	T	C	0.61	0.05	0.17	0.01							5 altered motifs	IQCJ-SCHIP1	intronic
3	159336072	0.82	0.96	rs9870872	T	A	0.61	0.05	0.17	0.01							GATA,Irf,Pou2f2	IQCJ-SCHIP1	intronic
3	159337480	0.82	0.96	rs9819080	A	G	0.65	0.05	0.17	0.02			NHLF, HSMM, Huvec	31 cell types	4 bound proteins		Foxj1,Zfp105	IQCJ-SCHIP1	intronic
3	159338382	0.81	0.96	rs9824363	A	G	0.61	0.05	0.17	0.01		HSMM	4 cell types					IQCJ-SCHIP1	intronic
3	159341404	0.81	0.96	rs61018732	G	C	0.6	0.05	0.17	0.01							Esr2,Smad	IQCJ-SCHIP1	intronic
3	159341465	0.81	0.96	rs61373569	T	C	0.66	0.05	0.17	0.02								IQCJ-SCHIP1	intronic
3	159342699	0.82	0.96	rs16830534	T	G	0.61	0.05	0.17	0.01			NHEK, HMEC				5 altered motifs	IQCJ-SCHIP1	intronic
3	159342865	0.82	0.96	rs73012253	C	G	0.61	0.05	0.17	0.01			NHEK, HMEC				Foxj2,Zfp105	IQCJ-SCHIP1	intronic
3	159343853	0.81	0.96	rs59556163	C	T	0.74	0.06	0.17	0.04							PPAR,PTF1-beta,STAT	IQCJ-SCHIP1	intronic
3	159344907	0.87	0.99	rs4494974	G	C	0.61	0.05	0.17	0.01							5 altered motifs	IQCJ-SCHIP1	intronic
3	159347475	0.87	0.99	rs1437520	A	T	0.61	0.05	0.17	0.01			NHLF				6 altered motifs	IQCJ-SCHIP1	intronic
3	159347730	0.87	0.99	rs9882328	C	T	0.61	0.05	0.17	0.01			NHLF, HSMM				Nanog,Sox	IQCJ-SCHIP1	intronic
3	159348014	0.87	0.99	rs9848763	A	C	0.61	0.05	0.17	0.01				HNPCEpiC, HPdLF			8 altered motifs	IQCJ-SCHIP1	intronic
3	159348112	0.87	0.99	rs9848897	A	T	0.66	0.05	0.17	0.02				HNPCEpiC, HPdLF			7 altered motifs	IQCJ-SCHIP1	intronic
3	159349481	0.87	0.99	rs1816621	C	T	0.61	0.05	0.17	0.02			NHEK, HMEC				5 altered motifs	IQCJ-SCHIP1	intronic
3	159349702	0.87	0.99	rs1347671	A	G	0.48	0.04	0.17	0.01			HMEC				7 altered motifs	IQCJ-SCHIP1	intronic
3	159352025	0.95	0.99	rs78881494	A	G	0.42	0.04	0.16	0.02			NHLF, HSMM				5 altered motifs	IQCJ-SCHIP1	intronic
3	159352143	0.95	0.99	rs9832970	G	A	0.42	0.04	0.16	0.02			NHLF, HMEC, HSMM				DMRT2,Nanog,Pou2f2	IQCJ-SCHIP1	intronic
3	159352596	0.87	0.96	rs77954255	C	G	0.01	0.01	0.15	0.01			5 cell types	HA-sp	KAP1		7 altered motifs	IQCJ-SCHIP1	intronic
3	159353144	0.95	0.99	rs28434644	T	C	0.54	0.04	0.16	0.05			4 cell types		GR,POL2		Ets,Pax-5	IQCJ-SCHIP1	intronic
3	159354028	0.96	1	rs1962890	G	A	0.6	0.04	0.16	0.04							ZBTB33	IQCJ-SCHIP1	intronic
3	159355906	0.96	1	rs11921824	T	C	0.56	0.04	0.16	0.02			4 cell types	39 cell types	4 bound proteins		LF-A1	IQCJ-SCHIP1	intronic
3	159355918	0.96	1	rs11921827	T	C	0.48	0.04	0.16	0.02			4 cell types	25 cell types	4 bound proteins		AP-1,Gfi1	IQCJ-SCHIP1	intronic
3	159356482	0.88	0.96	rs9826417	C	G	0.45	0.04	0.15	0.02			HMEC, HSMM				Nkx3	IQCJ-SCHIP1	intronic
3	159357047	0.86	0.93	rs16830546	T	C	0.08	0.01	0.15	0.01								IQCJ-SCHIP1	intronic
3	159357525	0.96	1	rs9851700	T	C	0.48	0.04	0.16	0.02							LUN-1	IQCJ-SCHIP1	intronic
3	159358778	0.96	1	rs750688	C	T	0.5	0.04	0.16	0.02							5 altered motifs	IQCJ-SCHIP1	intronic
3	159359371	0.88	0.96	rs9842465	G	T	0.45	0.04	0.15	0.02							HMG-IY,Irf,p300	IQCJ-SCHIP1	intronic
3	159359429	0.96	1	rs9879792	A	C	0.5	0.04	0.16	0.02								IQCJ-SCHIP1	intronic
3	159359872	0.96	1	rs9809076	A	C	0.57	0.04	0.16	0.02							HNF1,OTX,Pou3f2	IQCJ-SCHIP1	intronic
3	159360233	0.96	1	rs9867212	T	C	0.5	0.04	0.16	0.02							AhR::Arnt,Arnt	IQCJ-SCHIP1	intronic
3	159361672	0.96	1	rs16830575	A	G	0.48	0.04	0.16	0.01							4 altered motifs	IQCJ-SCHIP1	intronic
3	159362244	0.97	1	rs16830579	G	A	0.48	0.04	0.16	0.02							GATA	IQCJ-SCHIP1	intronic
3	159362915	1	1	rs16830581	C	G	0.5	0.04	0.15	0.02							7 altered motifs	IQCJ-SCHIP1	intronic
3	159364557	0.96	1	rs9290046	T	C	0.52	0.04	0.16	0.02							SIX5	IQCJ-SCHIP1	intronic
3	159364915	0.96	1	rs9290047	C	T	0.52	0.04	0.16	0.02							En-1,RORalpha1	IQCJ-SCHIP1	intronic
3	159365432	0.96	1	rs16830589	T	C	0.52	0.04	0.16	0.02							GATA,Hoxa5	IQCJ-SCHIP1	intronic
3	159366426	0.95	0.99	rs77414618	C	G	0.52	0.04	0.16	0.02							Pax-4,RREB-1,Zfp740	IQCJ-SCHIP1	intronic
3	159367303	0.95	0.99	rs1347672	C	A	0.55	0.04	0.16	0.02							E2F,YY1,p300	IQCJ-SCHIP1	intronic
3	159367346	0.88	0.96	rs1816622	G	A	0.46	0.04	0.15	0.02							8 altered motifs	IQCJ-SCHIP1	intronic
3	159367925	0.89	0.97	rs74550897	A	T	0.46	0.04	0.15	0.02							Foxq1,TATA	IQCJ-SCHIP1	intronic
3	159368581	0.96	0.99	rs1978830	T	C	0.52	0.04	0.15	0.02			NHEK	4 cell types			Hoxa9,Hoxd10	IQCJ-SCHIP1	intronic
3	159368828	0.96	0.99	rs2886621	C	T	0.53	0.04	0.15	0.02				RWPE1,SAEC				IQCJ-SCHIP1	intronic
3	159369276	0.96	0.99	rs9829310	A	G	0.52	0.04	0.15	0.02							Foxa,GR	IQCJ-SCHIP1	intronic
3	159369874	0.89	0.97	rs79167961	C	T	0.09	0.01	0.15	0.01							4 altered motifs	IQCJ-SCHIP1	intronic
3	159371068	0.96	0.99	rs9877425	C	A	0.51	0.04	0.15	0.02								IQCJ-SCHIP1	intronic
3	159372107	0.96	0.99	rs6772245	A	T	0.59	0.04	0.15	0.02				5 cell types	4 bound proteins		4 altered motifs	IQCJ-SCHIP1	intronic
3	159372345	0.89	0.97	rs9883498	G	A	0.51	0.04	0.15	0.02								IQCJ-SCHIP1	intronic
3	159372637	0.89	0.97	rs13434009	A	C	0.51	0.04	0.15	0.02				SK-N-SH_RA			TAL1	IQCJ-SCHIP1	intronic
3	159373158	0.88	0.97	rs79347224	C	T	0.07	0.01	0.14	0.01							Sox	IQCJ-SCHIP1	

Colored SNP numbers are linked to HaploReg pages detailing the information.

Supporting Information 5: Canonical pathway-charts

Categories	Diseases or Functions Annotation	*P*-Value	Molecules	# Molecules
Liver Cirrhosis	Cirrhosis	1.28E-06	CCR1,IL10,IL12RB2,IL23R	4
Liver Inflammation/Hepatitis	inflammation of liver	1.16E-05	IL10,IL12RB2,IL23R,STAT4	4
Increased Levels of Creatinine	increased quantity of creatinine	1.52E-04	IL10,STAT4	2
Liver Damage	injury of liver	1.68E-04	IL10,IL23R,STAT4	3
Renal Inflammation, Renal Nephritis	glomerulonephritis	1.77E-04	CCR1,IL10,IL12RB2	3
Liver Cirrhosis, Liver Failure	acute-on-chronic liver failure	9.84E-04	IL23R	1
Liver Damage	inflammatory injury of liver	9.84E-04	IL10	1
Liver Inflammation/Hepatitis	mild alcoholic hepatitis	9.84E-04	IL10	1
Liver Inflammation/Hepatitis	severe alcoholic hepatitis	9.84E-04	IL10	1
Liver Inflammation/Hepatitis	viral hepatitis	1.36E-03	IL10,IL23R	2
Liver Damage, Liver Inflammation/Hepatitis	chronic hepatitis	1.41E-03	IL10,IL23R	2
Liver Fibrosis	fibrosis of liver	1.71E-03	CCR1,IL10	2
Renal Damage	damage of kidney	2.02E-03	IL10,STAT4	2
Liver Cirrhosis	cirrhosis of liver	2.25E-03	IL12RB2,IL23R	2
Liver Inflammation/Hepatitis	experimental hepatitis	6.38E-03	IL10	1
Increased Levels of AST	increased localization of AST	6.87E-03	STAT4	1
Liver Inflammation/Hepatitis	acute hepatitis	7.36E-03	IL10	1
Increased Levels of Blood Urea Nitrogen	increased quantity of blood urea nitrogen	7.36E-03	STAT4	1
Glutathione Depletion In Liver	quantity of glutathione in liver	7.36E-03	IL10	1
Increased Levels of ALT	increased localization of ALT	9.31E-03	STAT4	1
Glomerular Injury	formation of glomerular crescent	1.03E-02	CCR1	1
Liver Necrosis/Cell Death	apoptosis of liver cell lines	1.52E-02	IL10	1
Liver Damage, Liver Inflammation/Hepatitis	chronic hepatitis B	1.61E-02	IL23R	1
Cardiac Inflammation	myocarditis	1.90E-02	STAT4	1
Liver Damage, Liver Inflammation/Hepatitis	chronic hepatitis C	2.77E-02	IL10	1
Liver Regeneration	regeneration of liver	3.92E-02	IL10	1
Hepatocellular Carcinoma, Liver Hyperplasia/Hyperproliferation	hepatocellular carcinoma	4.10E-02	IL10,PSORS1C1	2
Renal Damage	injury of kidney	4.15E-02	STAT4	1
Liver Cirrhosis	primary biliary cirrhosis	4.63E-02	IL12RB2	1
Increased Levels of Red Blood Cells	increased quantity of red blood cells	4.77E-02	IL10	1
Glomerular Injury	glomerulosclerosis	6.40E-02	CCR1	1
Liver Necrosis/Cell Death	apoptosis of hepatocytes	6.77E-02	IL10	1
Cardiac Infarction	myocardial infarction	1.02E-01	IL10	1
Liver Steatosis	hepatic steatosis	1.39E-01	IL10	1
Liver Hyperplasia/Hyperproliferation	liver cancer	1.57E-01	CCR1,ERAP1,IL10,IL23R,PSORS1C1	5

Supporting Information 6: BD genelists from GWAS catalog, IBD gene lists from GWAS

BD genelists from GWAS catalog
CCR1
ERAP1
FLT1P1
IL10
IL12A-AS1
IL12RB2
IL23R
KLRC4
KLRK1
LOC102724748
PSORS1C1
STAT4
TFCP2L1
TRNAI25

IBD gene lists from GWAS catalog
AAMP
ACSL6
ADA
ADAD1
ADCY7
AK3
AMIGO3
APEH
ARHGAP30
ARPC
ATG16L1
ATG16L2
BAT2
BCAR1
BRE
BSN
BTNL2
C10orf58
C1orf141
C1orf93
C2orf74
CARD11
CARD9
CCDC116
CCDC88B
CCL1
CCL11
CCL13
CCL7
CCL8
CCR6
CD19
CD48
CD6
CEP250
CNNM1
COX15
CPAMD8
CREB5
CTRB1/2
CTSW
CTSZ
CUTC
CXCL1
CXCL2
CXCL5
CXCL6
CYLD
DBP
DDR1
DGKD
DUSP5
EHBP1
EIF3C
ELF1
ENTPD7
EP300
ERAP1
ERFFI1
F11R
FAIM3
FASLG
FCGR2B
FCGR2C
FCGR3A
FCGR3B
FCHSD2
FCRLA
FGFR1OP
FIGNL1
FLOT1
FLRT1
FOSL1
FYN
GABBR1
GART
GMPPB

GOT1
GPR18
GPR65
GPX1
GSDMA
GSDML
GSMDL
GTF2H4
HLA-A
HLA-C
HLA-DOB
HLA-DQA1
HLA-DQB1
HLA-DRA
HLA-DRA
HLA-DRB1
HLA-DRB5
HLA-F
HLA-G
HLA-H
HMHA1
HORMAD2
HSPA1L
HSPA6
ICAM1
IFNAR1
IFNAR2
IKZF1
IKZF3
IL10
IL10RB
IL13
IL15RA
IL18R1
IL18RAP
IL19
IL1R1
IL1R2
IL1RL1
IL1RL2
IL21
IL22
IL23R
IL24
IL26
IL27
IL3
IL31RA
IL4
IL5
IL8
IL8RB
INPP5D
INSL4
IP6K1
IPMK
IRF1
IRF1
IRF5
IRGM
ITIH4
ITPR3
IZUMO1
JAK2
JRKL
KIAA1841
KPNA7
LAMB1
LAT
LILRB4
LNPEP
LOC645266
LPXN
LRAP
LRRK2
LSP1
LST1
LTA

LTB
MANBA
MAPK1
MAPKAPK2
MICB
microRNA2276
MIF1
MIR3939
MIR588
MLH3
MLN
MLX
MMP9
MOG
MSH5
MST1
MST1R
MSTO1
MTMR3
MUC1
NDFIP1
NDUFAF1
NFKBIL1
NKD1
NKX2-3
NKX3
NLRP2
NLRP7
NOS2
NOTCH4
NUPR1
NUSAP1
NXPE4
ORMDL3
OSM
OTUD3
P4HA2
PAPOLG
PDLIM4
PEX13
PF4
PF4V1
PIGR
PIM3
PLA2G2E
PLCH2
PLCL1
PLTP
PMPCA
PNMT
PRDX5
PRG2
PRG3
PROCR
PSMB9
PTGDR2
PTPN22
PUS10
RASGRP1
RASIP1
RASSF5
REL
REV3L
RIMBP3
RMI2
RNASE2
RNASET2
RP11-525A16.1
RTEL1
RXRA
SBNO2
SDCCAG3
SEC16A
SERBP1
SH2B1
SLAMF1
SLAMF7
SLC10A4

SLC11A1
SLC22A4
SLC22A5
SLC25A15
SLC26A3
SLC2A4RG
SLC35D1
SLC44A4
SNAPC4
SNX20
SNX32
SOCS1
SPHK2
STAT4
STAT5A
STAT5B
STMN3
SULT1A1
SULT1A2
TBC1D1
TEC
TMEM50B
TNF
TNFRSF14
TNFRSF6B
TNFRS-F6B
TNFRSF9
TNFSF15
TNFSF4
TNFSF8
TNPO3
TPPP
TRPT1
TTYH3
TUBD1
TYK2
UBE2L3
UBQLN4
UCN2
USF1
USP34
USP4
USP40
UTS2
VARS2
ZBTB46
ZGPAT
ZHP1
ZNF300P1
ZNRF1
ZPBP2
ADAM30
ADCY3
ADO
AHSA2
AIF1
APOBEC3G
ARPC2
ATF4
ATG16L1
ATG16L2
BACH2
BTNL2
C10orf67
C11orf30
C11orf9
C13orf31
C1orf106
C1orf53
C20orf70
C20orf71
C2orf74
C8orf84
CACNA2D1
CALM3
CAPZB
CARD15
CARD9

CCDC101
CCDC139
CCL2
CCNY
CCR6
CD226
CD244
CD40
CD5
CDH1
CDKAL1
CEBPB
CEBPG
CEP72
CFB
CIITA
CISD1
CLN3
CNTF
COL5A1
CPEB4
CREM
CRTC3
CSF2
CTDSP1
CXCL3
CXCR1
CXCR2
CXCR5
DAP
DCLRE1B
DENND1B
DLD
DNMT3A
DNMT3B
DOK3
EPO
ERAP2
ERG2
ESRRA
EXOC3
FADS1
FADS2
FCGR2A
FCHSD2
FGFR10P
FOS
FOSL2
FOXP2
FUT2
GALC
GCKR
GNA12
GPR183
GPR35
GPX4
GSDMB
GSDML
HCG26
HCG9
HCK
HDAC9
HLA
HLA-DQA1
HLA-DQA2
HLA-DQB1
HLA-DRA
HLA-DRB1
HNF4A
IBD5
ICAM3
ICOSLG
IER3
IFIH1
IFNG
IFNGR2
IKZF3
IL10

IL12B
IL12RB2
IL12RL2
IL17REL
IL18R1
IL1R2
IL2
IL20
IL23R
IL26
IL27
IL2RA
IL3
IL6ST
IL7R
IL8RA
INPP5D
INPP5E
INSL6
IRF8
IRGM
ITGAL
ITLN1
ITPKA
JAK2
JAZF1
K1F21B
KCNB2
KIAA1841
KIF21B
KIR2DL1
KLF3
LACC1
LAMB1
LDHD
LEMD2
LGALS9
LIF
LIF
LIME1
LITAF
LOC100129633
LOH12CR1
LSP1
MAML2
MAP3K7IP1
MAP3K8
MHC
MMEL1
MST1
MTMR3
MUC19
NDFIP1
NELL1
NFIL3
NFKB1
NKX2
NKX2-3
NOD2
NOTCH4
NR
NXPE1
ORMDL3
OTUD3
PARK7
PBX2
PDRM1
PFKFB4
PHACTR2
PLCL1
PNKD
PPAN
P2RY11
PRDM1
PRKCB
PRKCD
PSMB10
PSMG1

PSORS1C1
PSORS1C3
PTGER4
PTPN2
PTPN22
PTPRC
PUS10
RABEP2
RBM26
RBX1
RDH10
REL
REL
RELA
RFTN2
RIPK2
RIT1
RNASET2
RNF186
RORC
RPL7
RPS6KA2
RPS6KA4
RPS6KB1
RUNX3
SAG
SATB2
SCAMP3
SERINC3
SLC11A1
SLC25A28
SLC26A3
SLC2A4RG
SLC9A3
SLCO6A1
SMAD3
SMAD7
SMNDC1
SMURF1
SNAPC4
SOCS1
SOX11
SP140
SPRED1
SPRED2
SPRY4
STARD10
STAT1
STAT3
TAB1
TAGAP
TBC1D1
TCERG1L
TERF1
THADA
TMBIM1
TMEM17
TNC
TNFAIP3
TNFRSF18
TNFRSF4
TNFRSF6B
TNFRSF9
TNFSF11
TNFSF15
TNFSF18
TNFSF8
TNIP1
TNNI2
TNPO3
TRAF3IP2
TRIB1
TSPAN14
TSPAN33
TXK
UBA7
UBD
UBE2D1

UCN
UQCC
USP12
VAMP3
VDR
WBP4
YDJC
ZDHHC23
ZFP36L1
ZFP90
ZMIZ1
ZNF365
ZNF831
ZPBP
ZPBP2M
